# A clinical protocol for group-based ketamine-assisted therapy in a community of practice: the Roots To Thrive model

**DOI:** 10.3389/fpsyt.2025.1568017

**Published:** 2025-09-22

**Authors:** Shannon Dames, Pamela Kryskow, Vivian W. L. Tsang, Elena Argento

**Affiliations:** ^1^ Health and Human Services, Vancouver Island University, Nanaimo, BC, Canada; ^2^ Department of Family Medicine, University of British Columbia, Vancouver, BC, Canada; ^3^ Roots to Thrive for Psychedelic Therapy, Nanaimo, BC, Canada

**Keywords:** psychedelic, ketamine, community of practice (cop), group therapy, ketamine-assisted therapy, psychedelic-assisted therapy, protocol & guidelines

## Abstract

**Background:**

Ketamine-assisted therapy (KaT) has demonstrated therapeutic potential in treating depression, anxiety, and PTSD, driving interest in group-based models of care. Yet, few published protocols offer the comprehensive structure required for safe, scalable application in real-world clinical settings. The RTT-KaT model offers a resilience-informed, community-anchored framework that integrates trauma-aware care with a respectful and intentional weaving of Western and Indigenous knowledge systems. Initially launched as a quality improvement initiative through a partnership between a Canadian university and a regional health authority, RTT-KaT has since evolved into a non-profit clinical program. To date, it has supported over 750 participants through more than 2,000 KaT sessions and 700 Community of Practice groups. RTT-KaT is a culturally informed, resilience-focused model of group-based psychedelic-assisted therapy developed and refined since 2018. The model is rooted in the intentional weaving of Western clinical frameworks and Indigenous knowledge systems, grounded in principles of relational accountability, cultural humility, and trauma-informed care.

**Methods:**

This methods protocol describes the RTT-KaT model, including its medical, operational, and ceremonial components. Delivered over 12 weeks, the program embeds three intramuscular or sublingual ketamine sessions within a structured Community of Practice. Weekly large- and small-group sessions are grounded in somatic, relational, and culturally responsive principles. The program is co-facilitated by a multidisciplinary—and often multicultural—team including healthcare providers, therapists, and somatic energy practitioners. A structured, psychology- and resilience-informed curriculum cultivates core resilience factors—such as congruence and sense of coherence—through practices that foster awareness, meaning-making, somatic regulation, and alignment with one’s values and purpose.

**Results:**

Ketamine is positioned as an amplifier, not the primary driver of change. Ceremonial framing, intentional group process, and a relationally anchored curriculum serve as central mechanisms supporting meaningful change. Supplementary materials detail protocols for screening, dosing, consent, medical monitoring, and integration. While early outcome data have been published elsewhere, this article presents the methodology and protocol to support replication, adaptation, and ongoing evaluation.

**Discussion:**

RTT-KaT offers a structured, scalable, evidence-informed, and culturally responsive model that bridges clinical safety with both Western and Indigenous knowledge systems. A longitudinal follow-up study is currently underway to evaluate long-term impact and guide future implementations.

## 1 Introduction

The Roots to Thrive ketamine-assisted therapy (RTT-KaT) protocol offers a real-world model for delivering group-based psychedelic therapy within a resilience-informed framework. Developed in 2018 in British Columbia, Canada, through a collaboration between a university and a regional health authority, the program was initially launched as a quality improvement initiative to address treatment-resistant mental health conditions and caregiver burnout. The structured Community of Practice (CoP) served as the primary intervention, with psychedelic therapy provided as an adjunct. While not the only psychedelic explored (psilocybin and MDMA are also provided via special approvals), ketamine has been the primary medicine used—largely due to its accessibility (it can be prescribed without special permissions from Health Canada) and its compatibility with group-based therapeutic processes. Early findings demonstrated significant improvements in symptoms of generalized anxiety, PTSD, depression, and overall functioning (1–3). In 2021, the program transitioned into a non-profit clinical service operating within an ongoing quality improvement framework. To date, it has supported over 750 participants through more than 700 CoP sessions and 2,000 ketamine treatments.

The RTT-KaT model embeds three ketamine sessions within a structured 12-week CoP framework, delivered by a multidisciplinary team that includes healthcare providers, therapists, facilitators, and, where appropriate, Indigenous Elders. Grounded in trauma-informed, culturally responsive, and somatic-based practices (4), the program is guided by a resilience-focused curriculum designed to foster congruence and a sense of coherence, while leveraging the benefits of co-regulation and supporting the development of secure attachment. Within this context, ketamine serves as an adjunctive catalyst—facilitating openness, deepened insight, and engagement—rather than acting as the primary driver of therapeutic change.

The RTT-KaT protocol is grounded in several foundational theories, including Carl Rogers’ principles of congruence and unconditional positive regard (5, 6), and Antonovsky’s concept of sense of coherence (7). Its therapeutic foundation is further supported by a growing body of literature affirming the safety and efficacy of ketamine in psychiatric care (8–13), including its role in enhancing neuroplasticity and psychological flexibility (14–16). The model is further distinguished by its ceremonial framing, integration of Indigenous teachings, and the involvement of Elders and traditional knowledge keepers—components that foster a culturally grounded and relationally attuned approach to care (17). Emphasis is placed on therapeutic set and setting, including the program’s structured and predictable rhythm, its ceremonial context, and the intentional use of music as an active therapeutic agent (18).

Although not developed as a formal research intervention, RTT-KaT has been refined through an iterative quality improvement process, informed by ongoing participant feedback, facilitator reflection, and real-world application. This methods article presents, for the first time, the complete RTT-KaT protocol, detailing its clinical, operational, and ceremonial components. **Supplementary Materials** are also included to support safe, ethical, and culturally responsive implementation. Together, these tools offer a replicable foundation for clinical teams and community organizations seeking to deliver resilience-centered, group-based psychedelic-assisted therapy.

This model reflects a transformative arc that moves from knowing to being to doing - each phase supporting therapeutic integration. Knowing involves cognitive understanding of one’s patterns, values, or traumas; being marks the internalization of insights through felt awareness and presence; and doing signifies the alignment of behavior with insight - the translation of embodied insight into congruent action. This progression fosters sense of coherence, agency, and sustainable change - core goals of the therapeutic journey.

## 2 Personnel, materials, and equipment

The RTT-KaT protocol requires a multidisciplinary team, trauma-informed physical environment, and a set of structured participant-facing and clinical materials. This section outlines the essential components for safe and consistent delivery of the program.

### 2.1 Personnel and team composition

- Team members are selected based on their ability to embody the programs principles, scope of practice capacity, training in mental health and non-ordinary state support.
- Facilitation Team: Each small group is led by a dyad of trained facilitators, ideally with complementary backgrounds therapy, somatics, psychology, and cultural responsiveness. A ratio of one facilitator to every three to four participants is maintained.
- Medical Staff: A physician with experience in psychedelic-assisted therapy oversees the medical screening, ketamine dosing, and clinical safety. Registered nurses (RNs) conduct the initial screening, administer vital signs, and support session flow. At least one person on site must have up-to-date first aid and basic life support training.
- Elder or Cultural Guide: For cohorts with Indigenous participants or contextual partnerships, an Indigenous Elder may provide spiritual oversight, ceremonial context, and relational grounding.

### 2.2 Physical setting

- Ketamine sessions are conducted in a low-stimulus group setting designed to promote comfort and psychological safety. The space includes dim lighting, natural elements, comfortable furniture, and soft materials such as blankets and pillows. Participants are arranged in a circle on individual mats and are provided with reclining chairs for use during the verbal portions of the ceremony. Each participant has access to eye masks, weighted blankets, extra pillows, affirmation cards, and noise-canceling headsets—through which carefully curated music is played both in the room and directly to each individual. Paper bags are provided for tissues used during the session, which are later burned in a ceremonial practice rooted in Indigenous teachings gifted to the program. A detailed explanation of the space, supplies, and their intended purposes is included in the KaT facilitation script (**Supplementary File 8**).
- Environmental Features:
- Adjustable lighting.
- Soft surfaces for comfort.
- Soothing, curated music (delivered via high-quality sound system).
- Visual privacy and acoustic quiet.
- Nature incorporated through wall art and live plants.
- Medical and Emergency Supplies:
- Blood pressure monitor, pulse oximeter, thermometer (if illness is suspected.)
- Emergency and comfort medications, supplies and response protocols on site (all clinicians are required to review emergency protocols before KaT sessions).
- Emergency contact (and naming who will drive them home after the session) for each participant.
- Participant safety is prioritized through comprehensive screening, monitoring, and emergency preparedness protocols. Pre-session blood pressure must be below 150/90 mmHg to proceed, and vital signs are monitored before and after ketamine administration. In cases of elevated blood pressure or anxiety, rescue medications such as clonidine and captopril are available onsite, administered under physician guidance. Emergency response protocols are in place, including an on-site or on-call physician, and clearly assigned roles among the facilitation team. Safety procedures, including session-day documentation and post-KaT care instructions are provided in the **Supplementary Files**.

### 2.3 Participant-facing materials

The following materials are provided to support clarity, safety, and participant empowerment throughout the RTT-KaT program:

- Pre- and Post-Program Assessments: Participants complete a series of validated self-report measures at intake and discharge, including the *Generalized Anxiety Disorder-7 (GAD-7)*, *Patient Health Questionnaire-9 (PHQ-9)*, *PTSD Checklist for DSM-5 (PCL-5)*, and the *Brief Inventory of Psychosocial Functioning (B-IPF)*. An anonymous feedback link is also distributed periodically throughout the 12-week program, along with a final qualitative survey at discharge to inform ongoing quality improvement.
- Intake Forms, Consent, and Onboarding Agreements (**Supplementary File 1**): Includes referral criteria, informed consent documents, and a readiness checklist to assess alignment with program expectations.
- Participant Guide (**Supplementary File 2**): Outlines the program philosophy, weekly themes, consent process, and integration guidance to help participants prepare for and make meaning of their experiences.
- CoP Intentions and Agreements Template *(* **Supplementary File 3** *)*: A collaboratively developed set of community intentions that fosters psychological safety and relational accountability. During the first CoP session, participants review and refine the agreements together until consensus is reached.
- Post-KaT Safety Sheet *(* **Supplementary File 4** *):* Provides aftercare instructions, including driving restrictions and contact information for additional support following ketamine sessions.

### 2.4 Facilitator and clinical tools

The following resources support safe, consistent, and ethically grounded delivery of RTT-KaT:

- CoP Facilitation Guide *(* **Supplementary File 5** *):* Guides facilitation of weekly CoPs.
- KaT Medical Protocol (**Supplementary File 6**): Provides clinical guidance on eligibility screening, contraindications, dosing parameters, and medical oversight during ketamine sessions.
- Session Record Templates (**Supplementary Files 7A, B**): Standardized forms for documenting vital signs, medications, participant check-ins, and touch preferences, supporting both clinical tracking and continuity of care.
- KaT Ceremony Script (**Supplementary File 8**): While not intended for verbatim delivery, the script provides a consistent ceremonial framework that helps anchor participants amid uncertainty and supports psychological safety.
- Ketamine Administration Procedure (**Supplementary File 9**): Outlines the ritualized clinical workflow for ketamine delivery, promoting consistency, minimizing risk of error, and reinforcing therapeutic containment across sessions.

## 3 Methods

### 3.1 Program overview

**Figure 1:**
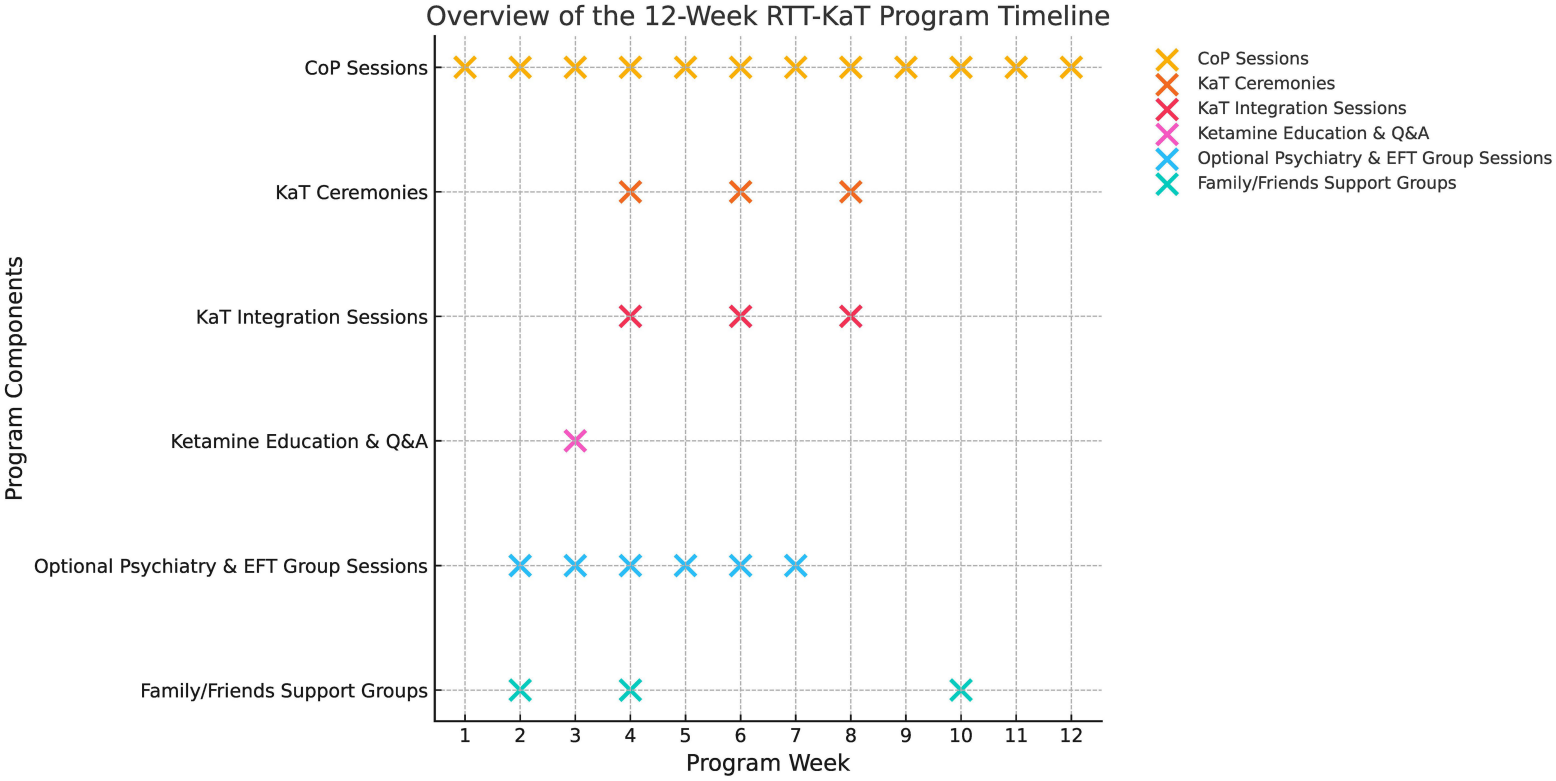
Overview of the 12-week RTT-KaT program timeline. The program consists of weekly CoP sessions, three structured KaT ceremonies and integrated preparation and integration components.

**Figure 1** provides a visual overview of the 12-week program, illustrating the timing of CoP sessions, ketamine ceremonies, preparatory and integration activities, and support offerings for both participants and their families.

Guided by a resilience-informed curriculum, each aspect of the program supports participants to develop the awareness and agency to move from knowing, to being, to doing - supporting intentional change that can be sustained in their day to day life. Weekly content follows a consistent structure (see weekly guide template in **Supplementary File 5**), emphasizing shared intentions, meaning making, emotional regulation, relational safety, and personal alignment—each contributing to the development of resilience through enhanced congruence and sense of coherence.

Additional support offerings include a mandatory ketamine education and Q&A session, optional weekly psychiatry groups and Emotional Freedom Technique (EFT) sessions. Family and friends support groups are offered at three key points to support the wider integration container. The program concludes with final CoP integration and discharge.

Rather than adhering to a manualized psychotherapy model, RTT-KaT draws from trauma-informed, somatic, and relational principles. Its primary therapeutic mechanism is rooted in the CoP environment, where shared intentions and relational agreements enable authenticity, co-regulation, and secure interpersonal connections. Each cohort includes 20–40 participants, subdivided into small groups of 6–9, and is supported by a multidisciplinary team of medical professionals, therapists, somatic practitioners, and cultural knowledge keepers.

All ketamine sessions are medically supervised by licensed prescribers and delivered in accordance with provincial and federal clinical governance standards. Weekly large- and small-group sessions integrate somatic practices, guided inquiry, and culturally responsive agreements to support psychological safety. Participants are expected to attend all 12 weekly sessions, with an option to join additional support sessions, and must complete all preparatory components before receiving their first ketamine treatment.

### 3.2 Participant screening and intake

Information sessions are offered for participants, friends, and family members before applying to the program, providing an overview and answering questions. To be eligible, they require a referral by licensed healthcare providers and undergo a multi-step screening process to assess readiness and suitability. The process includes:

- Intake agreements, outlining participant responsibilities, group participation expectations, confidentiality guidelines, consent for treatment, and safety commitments. These agreements are designed to establish mutual understanding, promote psychological safety, and ensure informed and accountable participation throughout the program.
- Mental health assessments are conducted virtually and include the following standardized tools: the Brief Inventory of Psychosocial Functioning (B-IPF), the Generalized Anxiety Disorder 7-item scale (GAD-7), the Patient Health Questionnaire-9 (PHQ-9), and the PTSD Checklist for DSM-5 (PCL-5).
- RN Screening: A 45–60 minute virtual interview to assess medical and psychological history, trauma-informed readiness, commitment to group participation, and ability to co-regulate in a community setting.
- MD Screening: A physician with experience in psychedelic therapy conducts a medical evaluation, including medication and allergy review, vital signs, past experiences in non-ordinary states, and informed consent confirmation.
- Psychiatric Evaluation: If indicated, participants are referred for additional psychological assessment.
- Inclusion and exclusion criteria are outlined in the RTT KaT Medical Protocol (**Supplementary File 6**), and all participants must acknowledge intake agreements (**Supplementary File 1**).

### 3.3 Weekly CoP format

Each weekly RTT session includes both a Large Group and a Small Group segment. Weekly curriculum themes are outlined in **Table 1**, and a detailed session guide is provided in **Supplementary File 5**.

**Table 1:** RTT-KaT 12-Week Curriculum Overview.

Week	Curriculum Topic
1	Foundations
2	Supporting the Body to Hold Compassionate Space for ‘What Is’
3	Mindful & Heartful Listening
4	Inner Healing Intelligence & RAIN
5	Liminal Spaces and the Window of Tolerance
6	Emotional Conditioning and Pathways of Expression
7	Letting Go and Awakening to Purpose
8	Relationships and Attachment Tendencies
9	My Journey to Self-Compassion
10	Acknowledging Our Interconnectedness – Co-Creating Community
11	Generativity – Your Way Forward
12	Locating Your North Star

**Large group segment (30–40 minutes):**

- The session opens with a grounding invitation—typically offered by a local Elder or through a reflective reading—to help participants arrive fully and intentionally.
- Facilitators then offer key logistical updates, including reminders about optional psychiatry and somatic regulation sessions, friends and family support groups, integration opportunities, and upcoming preparation requirements for ketamine sessions.
- The central component of the large group is the “Coming to Know” segment, which introduces weekly themes (**Table 1**) drawn from evidence-based practices, professional expertise, and Indigenous teachings.
- Participants engage in a brief somatic regulation practice to deepen body awareness, support nervous system regulation, and foster alignment with personal values and intentions.
- The large group concludes with a “pause” practice to support integration and prepare for the transition into small-group relational work.

**Small group segment (70–90 minutes):** These facilitated sessions, led by dyadic or triadic teams, focus on compassionate witnessing, embodied presence, and emergent sharing. Small groups maintain consistent membership throughout the program to support the development of relational trust and psychological safety.

- The initial small group meeting includes a formal intentions and agreements process (**Supplementary File 3**), which invites participants to co-create the group container, establish shared values, and promote relational accountability. Mixed-gender groups are supported by gender-representative facilitators when possible. Facilitators are trained to model vulnerability and move at the pace of trust development, guided by the principle of unconditional positive regard.
- Each session begins and ends with a brief check-in and check-out, during which participants are invited to share one to two sensations and one to two emotions without explanation or qualification. This somatic anchoring practice supports emotional awareness and nonverbal integration.
- Open-ended prompts aligned with the week’s *Coming to Know* theme encourage participants to explore emotional edges, practice vulnerability, and connect their inner experience with the collective learning process.
- All participants are invited to engage in the practice of compassionate witnessing, a relational skill that involves attuning to others with nonjudgmental presence and sharing one’s own felt sense—such as sensations or emotions—as a form of mirroring. This shared practice reinforces the program’s emphasis on co-regulation, emotional safety, and authentic connection.

Additional Sessions:

- Over the 12-week period, participants and their support networks are offered several additional small groups, beyond the weekly CoPs, KaT sessions, and post-KaT integration sessions. In addition to dedicated family and friends sessions designed to educate and prepare loved ones, all participants attend a mandatory ketamine information and Q&A session prior to their first medicine session. Additional optional support offerings include group psychiatry sessions (often including an Indigenous Elder as co-lead) and Emotional Freedom Technique (EFT) workshops, which provide additional tools for emotional regulation and integration.

### 3.4 Ketamine sessions

Participants receive three ketamine sessions spaced across the 12-week program, typically beginning between weeks 4 and 6. These sessions are held in a ceremonial group setting and follow a consistent, trauma-informed structure designed to foster emotional safety, predictability, and inner-directed presence.

#### 3.4.1 Preparation

Preparation for ketamine sessions includes the following components:

- Group education and consent review: A 1-hour group session introduces participants to the therapeutic rationale for ketamine use, expected psychedelic effects, safety procedures, and the session-day flow. A video of how touch and energy field support can be provided during their KaT is provided the week before the first session. Informed consent is revisited during this session to ensure participants remain empowered and informed throughout the process.
- Individual physician consultations: Each participant meets privately with a physician experienced in psychedelic-assisted therapy to confirm dosage, review medical readiness, and discuss any questions or concerns. An intentional and trauma-informed process is used to elicit participants’ touch preferences and boundaries, ensuring informed consent, trust and agency. To reinforce this process, a preparatory video outlining the use of therapeutic touch and energy field support is provided prior to dosing, deepening participants’ understanding and consent capacity.
- Environmental orientation: Participants are familiarized with the clinic environment, including pictures and a video, showing the medicine room layout and the music playlist that will accompany the session.

#### 3.4.2 Session day

On the day of the ketamine session, participants are welcomed into a quiet, low-stimulus room arranged in a circle, with dim lighting, soft furnishings, and blankets to support comfort and containment. Facilitators are already present in the space, modeling an inward focus and available to help participants settle. The ceremonial tone of the room invites a shift from outward social engagement (“up and out”) to inward exploration (“in and down”). A structured session record (**Supplementary File 7b**) is used to guide and document key safety steps. This includes:

- Collection of an emergency contact number for the participant’s driver,
- Completion of baseline vital signs and weight measurements,
- A brief private medical check-in to confirm readiness and finalize dosage,
- Distribution of the post-session safety sheet (**Supplementary File 4**).

The medicine ceremony is guided by a highly structured and scripted protocol, designed to ensure continuity, minimize ambiguity, and foster psychological and relational trust. Participants are encouraged to prepare their space with intention and to remain inwardly focused throughout the experience. To preserve the contemplative and ceremonial atmosphere, verbal communication is kept to a minimum. Touch preferences are reviewed during Week 3 medical check-ins and are formally confirmed on the day of the session using two methods: a dedicated touch consent form (**Supplementary File 7a**) and a clearly visible whiteboard in the medicine room that lists individual preferences for all team members to reference in real time.

In alignment with teachings from local Elders, ketamine is introduced into the room with ceremonial intention, emphasizing the participant’s relationship with the medicine as an ally in healing. This approach highlights the role of intention-setting and respect for ketamine as a therapeutic agent and ally. Ketamine is administered either via sublingual lozenges (100–300 mg) or intramuscular injection (0.5–1.5 mg/kg), based on clinical assessment and participant preference. For participants who are uncertain about starting with a higher dose, an initial lower dose is provided with the option of a top-up—ranging from 5–20 mg, administered 10–20 minutes into the session. This flexible dosing strategy supports individualized care while maintaining therapeutic efficacy. A curated music playlist (18) accompanies the session to act as an emotional anchor, guide somatic and psychological processing, and serve as a co-therapist throughout the journey.

Following the session, participants remain in the space until they feel steady and ready to transition home. Once ready, they are accompanied to their designated driver. A no-driving policy is enforced for 24 hours. Session documentation (**Supplementary File 7b**) includes observations, clinical notes, and safety measures.

### 3.5 Integration and post-program support

Integration is woven into both small group sessions (where they remain with familiar CoP members) and dedicated integration meetings (mixing CoP members) following each ketamine session. Participants are encouraged to explore the contrast between “being” and “doing,” and to cultivate insights from their experiences within the CoP.

Post-session integration is structured through small-group CoPs and individualized support where needed. Participants are encouraged to engage in self-reflection, journaling, and ongoing community of practice. Where available, referrals to local suppliers and longitudinal follow-up assessments are used to ensure continuity of care.

All processes described in this Methods section are detailed further in the **Supplementary Materials**, including intake documentation, medical protocols, safety procedures, and group facilitation tools. These resources are provided to support adaptation and replication of the RTT-KaT protocol in diverse therapeutic environments.

Following the final CoP session, participants are invited to join alumni programming, which includes ongoing access to CoP groups and optional KaT refresher sessions, as clinically indicated. At discharge, participants complete the same set of standardized mental health assessments administered at intake. These results are summarized in a consultation note that is shared with the referring healthcare provider to support continuity of care.

## 4 (Anticipated) Results

Although this article does not present new outcome data, the RTT-KaT protocol has been implemented with more than 750 participants across 14 cohorts between 2020 and 2024. Published program evaluations from the initial cohorts (1–3) demonstrated meaningful improvements in symptoms of depression, anxiety, and PTSD. For example, 92% of participants who reported impaired life-work functionality at baseline indicated improvement by program completion, and most participants screening positive for PTSD no longer met diagnostic thresholds at 12 weeks. These early results—derived from a real-world, community-delivered setting—provided the foundation for ongoing protocol refinement through continuous quality improvement.

A subsequent safety and tolerability analysis involving 128 participants and over 400 ketamine sessions found both sublingual (100–300 mg) and intramuscular (0.5–1.5 mg/kg) routes to be well tolerated, with only transient and manageable side effects (3). No serious adverse events have been reported across any of the RTT-KaT cohorts to date, contributing to the protocol’s strong safety profile and feasibility for wider clinical application.

Notably, approximately 10% of RTT-KaT participants have self-identified as Indigenous. In response, a targeted quality improvement initiative was undertaken to better understand Indigenous participant experiences and adapt the model accordingly (17). Feedback emphasized the need for relational authenticity, the presence of Indigenous team members, and the integration of traditional healing values and ceremonial elements. These findings directly informed the development of the culturally responsive framework presented in this protocol.

Together, these published findings support RTT-KaT as a safe, well-tolerated, and scalable group-based approach to psychedelic-assisted therapy. The model’s adaptability across clinical, academic, and community contexts—particularly when guided by local cultural knowledge—positions it as a promising option for expanding access to integrative mental health care.

## 5 Discussion

### 5.1 Real-world foundations and evolving implementation

While the RTT-KaT protocol has not been evaluated through randomized controlled trials, it is grounded in real-world data, published outcomes, and an iterative quality improvement process. Embedding protocol dissemination within methods articles—such as this one—helps bridge innovation and evaluation, offering transparency for replication and adaptation while contributing to the ethical evolution of group-based psychedelic care.

This article presents a structured clinical model for delivering KaT within a Community of Practice (CoP) framework. While the protocol originated in a specific cultural and geographic context (Vancouver Island, Canada), it has since been adapted and delivered in a range of clinical, academic, and community-based environments. These implementations underscore the model’s flexibility, though further research is needed to evaluate its feasibility and effectiveness across broader and more diverse populations.

### 5.2 Integration of Indigenous and Western paradigms

A central strength of RTT-KaT is its integration of Western therapeutic principles with Indigenous relational values. Developed in partnership with Indigenous Elders and knowledge keepers, the model incorporates ceremonial elements, collective witnessing, and teachings rooted in interconnection, reciprocity, and relational accountability. These components reflect a worldview often underrepresented in psychedelic-assisted therapy literature and offer an inclusive alternative to individually focused models of care.

Throughout its development, RTT-KaT has prioritized cultural humility and careful differentiation between cultural appreciation and appropriation. Teachings are shared with permission, grounded in longstanding relationships, and embedded within appropriate ceremonial and community contexts. Co-developing protocols with Indigenous collaborators and maintaining feedback loops ensures the integrity of sacred knowledge is respected and that cross-cultural integration is approached with consent, transparency, and relational accountability.

### 5.3 Community-based framework for resilience development

Rather than employing a manualized therapy approach (e.g., CBT or ACT), RTT-KaT centers healing within a Community of Practice, where shared intentions and relational safety agreements create the conditions for authenticity, vulnerability, somatic awareness, and secure attachment—each unfolding at the pace of trust. Therapeutic change emerges through co-regulation, compassionate witnessing, and connection to personal and collective meaning. Weekly group sessions foster psychological resilience by cultivating congruence, a sense of coherence, and trauma-informed self-leadership.

### 5.4 Ketamine as an amplifier and therapeutic catalyst

A defining feature of the RTT-KaT model is its ceremonial framing of ketamine as an adjunctive catalyst—one that amplifies rather than drives the healing process. Rather than serving as the central mechanism of change, ketamine supports and enhances the inner work that unfolds within the broader therapeutic container. The three intramuscular sessions are embedded within a structured 12-week curriculum that incorporates multiple forms of “medicine,” including relational safety, somatic regulation, emotional processing, values alignment, and community connection. Ritual, music, and embodied practices are woven throughout to foster psychological safety, deepen insight, and support transformative experiences.

### 5.5 Implementation tools and iterative development

To support implementation, seven **Supplementary Files** accompany this article, offering practical templates and tools used in clinical delivery. While other published protocols have helped establish clinical standards for psychedelic-assisted therapy (19), the RTT-KaT model is distinguished by its resilience-informed curriculum and Indigenous wisdom-informed framework, delivered within a structured CoP model.

The RTT-KaT program continues to evolve through an ongoing quality improvement process. Templates and practices are updated regularly in response to participant feedback, multidisciplinary team reflections, and shifting regulatory standards. This flexible, iterative approach enables contextual adaptation and fosters ethical responsiveness across diverse populations.

### 5.6 Limitations and future directions

Although earlier publications reported outcomes from more than 400 participants (1–3), additional cohorts have since participated in the program, further informing protocol refinements. While the current paper focuses on sharing the clinical protocol, limitations include the absence of randomized control comparisons, potential variability in facilitator implementation, and the intensive resources required for training and delivery. Additionally, while culturally informed, adaptations must be made thoughtfully when applying the protocol in other settings to preserve cultural congruence and community safety.

The program has been adapted to psilocybin and MDMA therapy and may be further adapted to other psychedelic medicines and clinical populations, as well as the integration of this work across broader systems of care. A longitudinal follow-up study is currently underway to evaluate long-term outcomes across psychological, relational, and spiritual domains, contributing to an evolving understanding of the therapeutic potential of group-based KaT. By contributing a real-world, culturally responsive, and resilience-informed model, this protocol aims to expand the clinical landscape for group-based KaT and provide a scalable alternative for integrative mental health care.

## Data Availability

The original contributions presented in the study are included in the article/**Supplementary Material**. Further inquiries can be directed to the corresponding author.
