# Supplementary material for: A clinical protocol for group-based ketamine-assisted therapy in a community of practice: the Roots To Thrive model
Source: Front Psychiatry. 2025 Sep 22;16:1568017. doi: 10.3389/fpsyt.2025.1568017 (PMC12498912; doi:10.3389/fpsyt.2025.1568017)
Supplement: Supplementary file 1 [file DataSheet1.pdf]

Roots to Thrive

Ketamine Preparation, Administration, Documentation, and Clinician Oversight

(Revised February 2025)

Purpose: to ensure consistency in procedure and to and promote safety and efficiency in the preparation and administration of ketamine to support ketamine-assisted therapy sessions.

Nurse/nurses take Ketamine out of the safe, open the med container with code # and do count at once before continuing.

A.) The patient arrives, and the nurse takes BP (systolic must be <150) and HR, documented on the patient's chart. If systolic is >150, use relaxation techniques to help lower, and consider using the automatic blood pressure cuff in a quiet space (takes 3 pressures on its own – recording the lowest of the 3). Antihypertensives are available if necessary.

The nurse keeps the chart and takes it to the office for further processing. Nurses collaboratively proceed to draw ketamine doses and double-check each other's draws to ensure accuracy and protocols. Each nurse signs off on their tasks ensuring a comprehensive and traceable record of the care administered.

B.) Some participants may ask for anti-nausea medication as a preventive strategy. We do not offer to all, unless they indicate a history of needing. If yes, the medication will be taken into to room to be administered by the nurse about 20 minutes before Ketamine is given. Take extra Ondansetron with you into the room for patients who may need it at the end of their sit.

Preparing Ketamine

If the doctor is on-site, the Session record with the Ketamine order is brought to the nursing room post-dosing session. If there is not a doctor on-site, there will be written/electronic orders provided by the prescribing physician on the relevant “Design Sheet.”

Once participants have all completed BPs, drawing to make sure that all charts are in nursing room to prepare draws (at times, participants take charts to room, causing potential for missed draws).

Nurse prepares medication distribution area, make sure you have all supplies necessary, sharp’s container, alcohol swaps, cotton balls, needles, both sizes, area specifically for the charts, make sure door is closed so you are not interrupted during preparation of the medicine.

- ❑ Note the size of the needle. 1” or 1 ½” (to ensure given in muscle). If the size of the needle is not shown on the session record, pl, please check with the MD before drawing up ketamine, refer to weight on the dosing sheet if the dosing was done ahead of time).
- ❑ Note dose of Ketamine ordered and calculate volume of dose (50mg/1 mg) to be given. Double-check amount with dose chart on desk. If volume of injection will be more than 2.5ml consider dividing the dose between two syringes: Larger volume to be given in DG and smaller volume to be given in deltoid on same side.
- ❑ Clean top of vial with alcohol swab.
- ❑ Draw air into the syringe to the volume of fluid required. Inject that air into vial and draw out same amount of Ketamine (dose and amount ordered). Ensure there are no bubbles.

- ❑ If another nurse is available, have them check the dose and volume (provide initials as documentation of double check on KaT administration record). If there is not a second nurse available, the drawing nurse will perform a second check and initial the KaT administration record.
- ❑ Replace with a fresh safety needle (1" or 1 ½") after the draw.
- ❑ On label include: First Name (highlight any name alerts with yellow highlighter) and dose in mg. Please use black sharpie if possible (easier to see in low light).

Name	___mg/___ml
Ketamine 50mg/ml	

- ❑ Put the label on the needle cap, not on the barrel of the syringe. That allows us to double check for volume and bubbles.
- ❑ Place the prepared syringe in a plastic med tray with an alcohol swab and cotton ball. If using two syringes, label both and place them together in a plastic med tray with 2 swabs and cotton balls.
- ❑ Place all plastic med trays on a large tray to go into the room.
- ❑ Document ketamine used on narcotic record.

Top-Ups

- ❑ If a **top-up dose** has been ordered, complete a top-up dose slip (on yellow paper) with name/dose/time to be given and any specific MD directions.
- ❑ The nurse doing the ketamine preparation will prepare the top-up, label it as a top-up dose, document it on a narcotic record, and place it in a separate medication tray along with the top-up dose on a yellow slip and an alcohol swab and cotton ball.
- ❑ When ordering top-ups, MDs will let people know that the main dose will be in the hip, but the top-up will be given in the shoulder (to decrease the need for the participant to move) and remind participants to ensure their shoulder is accessible for the potential injection. If the MD is not present on KaT session days, the drawing nurse needs to prepare participants, ensuring their shoulder is accessible once they are in the KaT session.

<u>Controlled Substances Documentation</u>

- ❑ A Narcotic Count will be done at the beginning and end of each clinical day.
    - Enter each dose drawn up in the Controlled Substances binder.
    - Sign with your regular signature and designation (no initials, except for wastage).
    - Calculate and document the amount of Ketamine remaining. When you have drawn all doses for a group, ensure you have entered all doses and double-check your amount remaining.
    - Ensure any expired or unused doses are properly wasted (with two clinician initials) on the controlled substance record.

  - Controlled substances and the record must be always stored in the safe (or if mobile, must be always locked in a smaller safe, and returned to the larger safe before end of day).
- ❑ On the session record, sign that you drew up the ketamine with your regular signature and designation. Look over each chart to see that it is complete to this point.
- ❑ **Expiration dates:** please ensure the ketamine being drawn up is within its expiry date (found on the vial). If it is beyond the expiry, it must be wasted with a second clinician present. If it is a multiuse vial, it is useable for 30 days once opened. Once 30 days passes, the vial must be wasted. If you open a vial, write the date it was opened on the vial (so we know when the 30-day expiry date is). If it is a single use vial, any fluid left at the end of the day will need to be wasted (with a second clinician present).
- ❑ **Wasting:** all wastage must be done by two clinician witnesses and discarded into the biohazard/sharp container. Once wasting is complete, the two clinicians then initial on the narcotic record that it was wasted (either on the participant line if it was meant for them but unused, or the count line if it is an expired product). If there is only one clinician onsite, the controlled substance must be left in the safe, labelled as waste, until two clinicians are available to waste.

Distributing Ketamine in Room

- ❑ Place all individual plastic trays on a large tray along with the charts and take into the session room when the team rings that they are ready for the medicine. Alternatively, the nurse preparing the medicine will take it into the room during the bio break, which happens right before medication administration.
- ❑ As scripted (and adaptions welcome), the nurse will read a blessing as they hold the tray of ketamine/medicine and invite the facilitators in the room to distribute to each participant.
- ❑ The nurse will call out each participant's name, and hand it to the facilitator to distribute (ensuring the right dose goes to the right person).
- ❑ As part of the script, participants are then invited to hold the medicine and bless it if they wish while naming their intentions and pillars of strength.

Administration of Ketamine

- ❑ As scripted, at a point close to administration, a team member will ring the call bell and any available nurses and physicians will go to the room to inject the Ketamine.
- ❑ Participants have been asked to lay on their sides, belts loosened as necessary, with their hips up where they want the injection to be given. They will also be asked to place their medicine tray beside the mat on the side where they want their injection. It is also helpful if their chart is on that side. (If this has not happened, gently remind people of this.) For participants with a top-up order, ensure clothing provides easy access to deltoid muscle –remove sweaters, etc.
- ❑ Starting with those who have top ups, and then women first (because they metabolize ketamine more slowly than men), nurses and physicians will go to participants, pick up the syringe, address the person with the name on the syringe and say “(Name) may I have permission to give you ketamine in your hip?”
- ❑ With consent, and letting the participant know they can focus on their outbreaths, and that you will provide two taps before the injection (to mitigate suspense), landmark the Dorso gluteal

muscle from the Posterior Superior Iliac Spine to the Greater Trochanter of the Femur and choose injection site above that "line" (See illustration in Supporting Documents).

- ❑ Gently clean the site with an alcohol swab. Let the person know you will tap twice at the site before injecting Ketamine.
- ❑ Tap twice at site. Inject the Ketamine and place a cotton ball at the site.
- ❑ Guide the person to place their hand over the injection site and to gently rub the medication in. If 2 syringes have been prepared, give smaller dose in the deltoid on the same side using the same technique.
- ❑ Wish the participant a "good journey".
- ❑ Other team members will come along and get them set up and tucked in for the session. Do not recap the needle.
- ❑ Place the medication tray with the used syringe at the foot of the mat to show the dose has been given to that person. The trays will be gathered when all injections are done.
- ❑ Chart time, dose given, and site. Sign with your full signature and professional status.

Top-Up Administration

- ❑ Unless otherwise arranged, the nurse who drew up the Ketamine for the group will stay in the room for the 20 minutes post injection to give top-ups as ordered.
- ❑ If a top-up has been ordered, the MD order will specify:
  - Dose
  - Whether we should: 1) Give top-up only if the participant asks for it up to 20 minutes after initial dose given. 2) Ask the participant at the time shown on the chart if they want a top-up.
- ❑ If the top-up dose is not taken, the clinician will return it to the nursing room, and waste it with a second clinician present (both initialing the narcotic record).

Occasionally we may ask MD (Pam or another MD if Pam is not available in person or by phone) for a top-up order around the 20-minute mark for someone who does not have one and who is not going into the medication very deeply.

If a top-up is wanted, give the prepared dose following the steps above. **Give all top-ups in the Deltoid.** Ensure that the top-up is documented on the chart ***and*** the controlled substances book. If top-up is not given, chart that as well. Return the top-up dose to the nursing room.

Final Ketamine Checks

- ❑ As you collect all the medication trays, ensure all injections, including top-ups, are administered (or set aside for wasting if not).
- ❑ If another healthcare professional is present, transport the tray to the office for disposal of sharps.
- ❑ Ensure all labels with patient names on them are either disposed of in the sharp's container (caps with names on them) or shredded (shredder in nursing office).
- ❑ Sign off on any waste if needed – with a second clinician (i.e. top ups that were not used).
- ❑ Clean trays thoroughly and ensure ketamine is securely stored.

Participant Assistance During KaT

- ❑ Return to the patient area to provide support and aid.
  - o Ensure easy access to emesis bags, mouthwash, and ondansetron.
  - o Aid participants to washroom as needed (2-person aid, with possibility for wheelchair if needed).
- ❑ Have crackers (including gluten free) available in individual bowls for participants as they emerge from the medicine. If not ready for food, offering salt is another way to help them ground in their bodies (small amount only). The facilitation lead will announce when it is time to offer these, but best to prepare the crackers during the KaT session, so they are ready (minimizes post session hustling).
- ❑ Ensure there is ondansetron, ibuprofen, and acetaminophen in the room, in case participants emerge with nausea or headaches.
  - o If nauseated, remind them to keep their heads in a low position (not to sit up), as nausea is often a sign that they are not ready to have their head up yet. If this does not resolve it, ondansetron is available.

Discharge

- ❑ After the participant debrief and ceremonial closing, all participants must have a blood pressure completed, with a systolic pressure <150. If higher, have them practice relaxation techniques, and consider moving to a quiet space. It is rare that antihypertensives are necessary, but this is available if needed (check in with MD on site or on call if it is still >150).
- ❑ On the KaT admin record, note if they were escorted to their driver, to the front door, or if they left on foot (important to make sure they are not driving post-KaT). Upon entry, they were asked who their driver was, and a phone number was provided (contact driver if needed).
