# Supplementary material for: A clinical protocol for group-based ketamine-assisted therapy in a community of practice: the Roots To Thrive model
Source: Front Psychiatry. 2025 Sep 22;16:1568017. doi: 10.3389/fpsyt.2025.1568017 (PMC12498912; doi:10.3389/fpsyt.2025.1568017)
Supplement: Supplementary file 2 [file DataSheet2.pdf]

Roots to Thrive KAT Session #1 Sequence – C14

Print in PDF format, so spacing enables flow while reading

ROOM SET UP

Facilitators and Somatic Energy Providers:

- For session 3, **have golden ropes and T-shirts in the room**
- **Mats** set up (one per participant if dense foam/2 if softer).
- **Stock Baskets:** headphones, emesis bag, eye masks (2 per basket), tea towel/bean bag, post-it or taped paper + sharpie for sitters.
- **Towels & cloths**: 5-6 of each for various uses.
- **Paper cups** available for water.
- **Tissue care**: Paper bags for ceremonial collection of tears.
- **Distributed items**: Tissues, sharps container, hand sanitizers.
- **Waste bins**: Check and empty as needed (including bathroom).
- **Comfort setup**: Pillows, mats, chairs for team. Ensure extra blankets & pillows for participants.
- **Clarity roles and Know the script well** so it flows naturally—avoiding monotone delivery and reliance on reading word-for-word.
- **Accessibility**: Ensure clear wheelchair path between mats (preferred for efficiency/safety).
- **Lighting**: Adjust as needed (Blue Room – switch #11 on breaker panel).
- **Painters tape** available to mark participant boundaries.
- **Wall Prep**:
    - **Paper** for "Dose time: _______"
    - **Whiteboard:** boundaries & support requests (post near door, marker attached).
    - **Flip chart** outlining session steps:
        - Overview
        - Opening circle & grounding
        - Check-in (internal sensations/emotions)
        - 2nd check-in (pillars/support requests/intentions)
        - Bio-break
        - Medicine administration
        - Transition circle sharing
        - Closing circle & grounding
        - Final vitals
- **Consider a stone to pass** between sharing transitions – multiple benefits.

How to Launch the Music:

- **Turn on the screen** – Press the button on the **top-right edge** of the device.
- **Unlock** – Swipe up and enter **8946** (code is on paper by the device).
- Spotify on computer/phone, connected to wired speakers/headphones
- **Select Playlist** – Choose from the **left-hand column** (title will appear if already selected).
- **Start Music** – Tap the **first song or album cover** (NOT green triangle, which may shuffle order).
- **Adjust Sound** –
  - **Tablet volume**: Set to **¾ full** for proper headphone control.
  - **Speaker volume**: Adjust separately (does not affect headphone level).

Nurse:

- **Medicine basket** (one per room) stocked with:
  - Extra face masks (x2), gloves (x4), emesis bags (x2), alcohol swabs
  - Cups, medicine cups, crackers, salt, Tums, ginger Gravol
  - Ibuprofen, acetaminophen, pulse oximeter
- **Ensure emergency medical equipment is functional** know location (nursing office)
- **Inform the team if there are resilience participants** (those not receiving ketamine).
- **Ensure crackers are stocked in rooms.**
- **Confirm enough BP cuffs** are available for team-assisted departure readings. Ensure team members know how to use them and signal you for sign-off when complete.
- **For those with needle phobias:**
  - Ask about support needs.
  - Offer a scarf to cover the tray (available in the nursing office).
  - Remind them of tapping as a support strategy.
  - Coordinate with somatic energy providers for additional support.

ARRIVAL

Breathe & ground yourself – Your calmness regulates the room. Facilitators stay inside; other team members handle external tasks.

Set the tone – Play calm music in the session room.

Welcome participants – Guide them to:

- **Remove shoes** (place by cubbies).
- **Keep only Ceremony/Sit materials bedside** (no cell phones).
- **Choose a spot**, unpack essentials, and store extra items (coats, bags, phones) in the designated corner.

Somatic Energy Providers:

- Explain **headphone use** (volume & channel switch).
- Invite them to write words for sitters (affirmations, reminders, pillars) and place them near their mat.
- Encourage placement of **personal objects** in the centerpiece with intention.

Transition to Ceremony Space: Ask for calm and silence -
"Please help us cultivate peace and focus by keeping conversations quiet and brief."

Team members and mentees: After logistics, settle into your space and ground yourself—**be the example**.

SESSION BEGINS – LOGISTICS - (20 MINUTES)

*Read **bold** sections as written, without additions or clarifications. With each session, the scripts are more succinct (repetition only where helpful).*

Turn off HEPA filter.

Take a Deep Breath (Project your voice)

Let's begin 😌.
I am (name) **and I…** (introduce yourself and your ancestry as you choose)**.**

We acknowledge our welcome on the unceded territory of the Snuneymuxw First Nation (Snu-Nay'-muwh) and hope today's healing supports reconciliation and the well-being of this land's first peoples.

To ensure you're familiar with everyone in the room, we'll introduce ourselves and let you know if anyone will be joining later (additional nurses, MD, etc.). **You can trust that only Roots to Thrive team members and mentees will be present today.**

We will be reading words co-created by the team and used across all KAT groups. Let the words provide a sense of comfort in their familiarity, helping us create predictability and trust in the process.

- **Please ensure your cell phone and any smart watches are turned off or switched to airplane mode – we want to ensure you are protected from any outside disturbances.**
- **The cedar and rosemary at the foot of your mat are gifts of nature to support you in this experience. Feel free to take them home with you.**
- **On the wall is the flow of the day - logistics - grounding and connection - check-in sharing – Bio break - medicine - integration sharing. The medicine administration will be about 45 minutes from now.**
- **You may notice paper bags around the room—these are for tissues used to collect your tears. Our Indigenous partners teach that tears of healing are sacred and should be honored. Rather than being discarded, these tissues can be burned in a fire with healing intentions. You are welcome to take them with you for this or leave them for us to handle with care.**

- Beside you is a basket—if you haven't already, go ahead and pick it up. Inside, you'll find headphones, a pen/marker, and various eye coverings - *Elder Geraldine has taught us that covering our eyes helps deepen our inward journey.*

Have you all been shown how to use the headphones? The color should be *Green/Blue/Red*. The volume dial is on the right.

- These headphones will help you go deeper with less external sound. If you hear sounds from other participants, please know that we are taking good care of them, and you can simply stay with your own experience - just redirect your attention inside and allow any external sound to exist in your peripheral awareness. You can turn up the volume on your headphones as well.
- The same music playing in the headphones will also be playing in the room should you choose to take them off.
- The music has been specially made for you and this journey. Let it carry you through the experience. If you do not like a song, just be curious about the experience, knowing that no matter what, the song will end and a new one will begin in a few minutes.
- If at any time we need to communicate with you we will gently squeeze your arm or shoulder, lift your headphones and whisper in your ear.
- After two hours, we will gently remove your headphones.
- If you come out of the medicine before this, we invite you to stay 'in and down' with your experience. As ordinary awareness returns, there's significant value in remaining present with insights, sensations and emotions. We will hold quiet space for others to complete any active experience before transitioning to group sharing.

At the end of your session, before we walk you out to meet your ride, we will need one final blood pressure for those receiving ketamine. Please help us ensure this is done.

If you need to go to the bathroom during your session, we will assist you to get there (*demonstrate bathroom technique*). We also have a wheelchair we will use if you aren't steady on your feet.

To help you stay internal and connected with your Inner Healer, we will only intervene when necessary and with your permission—unless needed for safety reasons. Permission will be confirmed in the check-in before medicine administration and can be requested with a hand wave during the medicine session.

While being careful to maximize time 'in and down', the support team will attend to subtle cues, including breath and body language to determine if touch may be helpful.

- Touch might include holding your hand, touching your shoulder or placing a hand on your forehead for calming assurance.
  - Or your ankles might be held to help you to settle.
  - Sometimes, we lightly hold points on the head, hands, or feet that connect with certain emotions. This can help you reconnect with feelings you've tucked away. Placing hands on the front or back of the chest can support the energy around your heart. We can also work in the space just above the body — this is called energy work and doesn't involve touch.
- Asking for physical support can help you feel safe, remind you that you are worthy of care, and gently expand your window of tolerance. Just as important, setting boundaries — like choosing not to be touched, saying where touch feels okay, or sharing your preferences around gender — can be equally empowering.
- Finally, please know that we will honour your requests and unless you have instructed no touch at all, you can ask for physical support at any time during the session.

Please join us for the Zoom integration session(s) Monday nights 6:00 – 8:00pm.

These integration sessions are a space to give and receive support about your experience today. Instead of your regular Tuesday night group, the groups will

be mixed — so you'll be with some familiar folks and some new ones, all moving together in the same stream of our shared healing.

Any questions about practicalities before we transition to our ceremony?

EXPECTATIONS (Keep projecting your voice)

Before we begin, we'd like to offer a few gentle reminders about expectations you may have brought with you today.

Please take a deep breath — and if it feels comfortable, soften your gaze or close your eyes.

- If you are holding ideas of what your experience is "supposed to be," may we invite you to let those go.
- You are unique. Your life is unique. Your experience is unique.
- You may have sadness. You may have joy.
- You may have darkness. You may have light, visuals & beauty.
- You may have silence. You may have insights.
- This experience may be like a nighttime dream, you might have an emotional experience, and... you will be safe.
- This medicine can help open the path between the mind and the heart. Whatever happens, you have what you need to take this journey.
- If you do not understand today, you will in the future. This is a time-release experience. More unfolds over time.
- Lean in - let go - be curious... if you see a door, open it – a staircase, explore it.
- Go gently, reach out and ask for the support you need to navigate through any uncomfortable parts of the journey.
- You've got this & we've got you. (Or whatever feels natural for you)

CLEARING/GROUNDING (Somatic Energy Provider)

SHORT CHECK-IN (Everyone)

Emotions & physical sensations: **Now we'll begin by checking in with emotions and physical sensations. Honoring the Coast Salish tradition, we will pass to the right. Who is feeling ready to start us off?**

LONG CHECK-IN (Participants Only): **(15 MINUTES)**

Now it is time to have each of you share:

- **Your pillars of strength,**
- **What kind of support and boundaries do you want from the team?**
    - Somatic energy provider will WRITE support requests and limitations on the whiteboard (*draw beds and note room markers, so it's intuitive*) for all sitters to be able to see and track (no need to record pillars or intentions), and ensure they are also charted on the touch consent record.
- **And your intention for this experience?**

(There won't be tagging, someone will start, and then again pass to the right)

VISUALIZING AND CONNECTING (5 MINUTES)

Before we begin the medicine session, let's take a moment to deepen our connection and strength as a group.

Take a deep breath in through your nose… and out through your mouth. If it feels comfortable, please close your eyes.

To create an energetic container for our work, place your hands over your heart. (Pause)

Now, imagine sending a taproot from the base of your spine down into the Earth. (Pause) With each breath, draw in its grounding, supportive energy. (Pause)

Next, picture branches extending from the crown of your head into the sky. (Pause) With each breath, invite light energy to flow down into you. (Pause)

Let these Earth and Sky energies meet and merge within you. Breathe in loving-kindness… and breathe out loving-kindness. (Pause)

Now, releasing your arms, allow this energy to flow to your right, around the group. (Pause) Feel yourself receiving from your left. (Pause) Keep this flow going, strengthening our connection.

As we close this visualization, let the sense of connection remain. Gently open your eyes and take a moment to look around at the group that will be with you on this journey.

BIO BREAK (5 MINUTES)

Before we settle in, this is your final opportunity to use the restroom. Please maintain an 'in and down' approach in this final break and let's keep voices down as we move around the building to support other groups in the process.

RN – please ensure that the Medicine is brought into the room during this break.

Once all have returned:

The RN brings the medicine tray forward, making eye contact with the group and offering a simple blessing. Please make sure it is authentic for you. A suggested blessing could be:

"This medicine, touched by many loving hands, may you receive it with grace, gratitude, and openness. May you feel the support of your allies, seen and unseen, and the safety among us. May these medicines serve your highest purpose."

The RN checks the label, names the recipient aloud, and hands it to the facilitator, who approaches the participant from the left side, offering it in sacred silence or with a few heartfelt words.

Now that you have received your medicine, I'd like to share a Nuu-Chah-Nulth teaching from our Uut Ustukyuu partners, Elders Dave Frank and JC Lucas—encouraging us to build a relationship with the medicine by introducing yourself and sharing your intentions.

(For those not receiving ketamine, you may do this with a meaningful object.)

Let's hold a few moments of silence to make space for this. (Pause for 1 min)

Let's begin our final preparation for the journey.   Please centre yourself, and direct your attention inward, as you listen to this poem by Anne Hillman:

We are **all** on a journey together
To the centre of the universe.
Look **deep** into yourself,
into another.
It is to a centre that is **everywhere**.

First you need only look:
Notice and honour, the radiance of
Everything about you.

Play in this universe.
Tend **all** these shining things around you:
The smallest plant, the creatures and
objects in your care.
Be **gentle** and **nurture**. Listen

As we experience and accept
All that we really are
We grow in care.
We grow to embrace **others** a**s** **ourselves**,
And learn to live as **one** among **many.**

Let's trust that whatever unfolds today for us as individuals and as a group will be supportive for all, meeting our personal and collective intentions for well-being. <u>We've got this.</u>

Please lay on your side, elevating the preferred hip you'd like to receive your injection.

Other team members are on their way to assist with dosing. Invite participants to lie down comfortably and prepare masks and headphones. Press the call bell

when the group is ready, and the medical team will enter to administer the medicine.

MEDICATION ADMINISTRATION

RN/MD:

- Begin with women.
- Confirm the hip ask for permission to proceed. Let them know you will tap twice right before you inject. In the meantime, tell them to focus on their breathing, and you will take care of the rest.
- Tap twice on the injection site before administering.
- Offer reassurance: *"Wishing you a beautiful journey – you are safe."*
- Place the sharp in the medicine tray (do not recap) at the foot of the mat once complete.

Facilitators:

- Follow behind RN/MD to assist with eyeshades, headphones, and tucking in.
- Offer simple cues: *"Go down and in," "Just ride the slide," "Breathe and allow."*
- Start Journey Music (ensure phone is on airplane mode).
- Press the first track (not the green triangle, which shuffles the playlist).
- Write Med Time on the wall.
- Turn on HEPA filter.

Room Setup:

- Use painter's tape to mark "no-go" zones requested by participants.
- Move decorative items off the floor for accessibility.
- **Once all are in and down and music is loud in room, nurse to prepare cracker bowls (you can set in basket or offer when session complete).**

Support Principles:

- Hold space with stillness, groundedness, and peace.
- Avoid standing over participants; distribute support evenly.
- Trust the process and your intuition.

- Respond to needs without rushing in.
- Prioritize non-verbal and energetic support unless verbal interaction is directly requested. Extract yourself once they are settled.
- Use the Call Bell at any time you need more team members to support the group. Ensure you have 2 RTT team members (not including practicum students/mentees) present in the room at all times.

TRANSITION

- At 2 hours, listen for the Tibetan Singing Bowl.
- The next track will be higher energy for awakening.
- Adjust timing based on group readiness:
  - Gently squeeze toes or shoulder before removing headphones.
  - Encourage participants to stay lying down and present.
  - Keep everyone in the room if possible; a bathroom break is coming soon.
  - Once all headphones are off, reduce music volume to zero for the poem.
  - Turn off HEPA filter.

**Keep everyone in the room if possible – bathroom break is happening very shortly.

POEM READ BY SAME PERSON WHO OFFERED THE POEM EARLIER

At this time, we gently draw together by hearing this poem shared at the beginning of the medicine session. Continue to rest or sit on your mats as we begin to move toward our transition circle.

We are **all** on a journey together
To the centre of the universe.
Look **deep** into yourself,
into another.

It is to a centre that is **everywhere**.
First you need only look:
Notice and honour, the radiance of
Everything about you.

Play in this universe.
Tend **all** these shining things around you:
The smallest plant, the creatures and
objects in your care.
Be **gentle** and **nurture**. Listen

As we experience and accept
All that we really are
We grow in care.
We grow to embrace **others a**s **ourselves**,
And learn to live as **one** among **many.**

TRANSITION SUPPORT
(brief pause before sharing circle – optionally turn music back on)

- Offer crackers or salt for grounding.

- Encourage everyone to use the bathroom NOW as the sharing circle is about to begin. If they prefer to wait, they can go afterward, but it's important to remain present during the integration sharing.

TRANSITION SHARING (60 MINUTES)

(Once everyone is settled, open the space for sharing)

Now we're going to open the space to share about your experience. After each share, compassionate witnessing is welcome - as always, please focus on listening rather than fixing, and we can also hold each other in appreciative silence.

Before we begin, please consider:

- **There's no obligation to share—passing is just as valid. If you pass, we'll check back later for another opportunity.**
- **Stay in your heart-body, focusing on the raw experience rather than searching for meaning. Meaning often unfolds naturally in the coming days and weeks.**
- **For example, if you felt deep compassion, love, or acceptance, focus on that felt sense—The images & ideas may fade but the felt sense is the path to cultivating that quality in daily life.**
- **If you catch yourself comparing your experience to others, gently return to curiosity about yourself—your experience is exactly right for you.**
- **Now, as you feel ready, share whatever you'd like us to know about your experience. Who would like to start us off?**

If no volunteers, ask whoever seems most out of the medicine if they are ready and wanting to share

- Compassionate witnessing after each
- Proceed around the circle to the right with those who want to share (if they are not ready – they can pass – then return and give them another opportunity in sequence)

- (Optional) group-level compassionate witnessing in brief to bring closure - remember, we do not want to engage DOing (cognitive processing)

INTEGRATION

As we prepare to step across the threshold into our daily lives, I'd like to offer a few words to support the next hours and days of integration:

- As you transition from your medicine journey, remain open and gentle with whatever arises. Release any urge to judge the experience or the emotions that may come. Remember that healing unfolds through the body's innate intelligence— let's honor its pace and process.
- Simply hold whatever arises in still awareness. There is no need to make sense of anything or fix anything.
- Trust that the cognitive mind is not needed here; in fact, it may only get in the way.
- Return to the breath and to the RAIN practice—recognizing and allowing whatever is present in the body. Stay curious and gently tend to what comes.
- Whatever is arising is necessary and right for you at this time. There are no good or bad emotions. Welcome and allow these waves of energy without resisting. Greet what comes with curiosity and compassion, developing a new relationship with memories and emotions.
- Whatever you choose to do with your integration time, be gentle with yourself. Resist the pressure to arrive anywhere. This waking-up is a process. Having that awareness, return to right here, right now. Still awareness in the body is the destination. And right here, right now, all is well.
- With that presence, bring your attention inward. Notice what is arising in your body right now. Lying or sitting on your mat, let's take a few moments to go in and notice, without a need to change anything, or make sense of what arises. (Allow 1 minute)

Now, with one long slow in-breath…and a long sounding out-breath... let's all bring our awareness back into the room.

CLOSING

For the last few hours, we've shared the gift of journeying together. Now, it's time to close our ceremony.

Please place your hands over your heart and take a deep breath. Again, picture sending a taproot from your spine into the Earth. With your breath, draw grounding energy up through this root into your body. (Pause)

Now, picture sending branches up into the sky through the crown of your head. With each breath, draw light energy down into you. Let these energies merge within you.

Offer these energies as loving-kindness to the group, flowing in a circle to your right. Feel yourself also receiving these energies from your left. As you feel into our connection, let's take a silent moment to send thanks and appreciation to each other. (Pause)

Whether your experience was subtle or profound, let's affirm that what unfolded exactly right to meet our individual and collective intentions for well-being.

Now, take a few cleansing breaths, and shake off anything that needs to be left here. Please hold onto what you want to take with you - you will know which is which.

While we end our time together, may this circle remain unbroken. We are connected in our shared intentions for healing and well-being. This golden thread of connection is always here and will support us as we move back into our daily lives. *(pause)*

FINAL BP

As we move into the final part of our time together, please sit back or lie down and relax while we take your final blood pressure

(IF some did not receive Ketamine: ***For those who did not receive ketamine, you are free to gently and quietly pack up your things and get ready to head home)***

When the RN or MD lets you know that your blood pressure is good to go, you can gently gather your things. Please be sure a Team member walks with you to your ride.

As you leave, keep your voices low to honour and protect the quiet space of other groups who may still be in ceremony.

If you have a bag of tissues you'd like us to burn, you're welcome to leave it with us.

Before we part, let's take a few 4-7-8 breaths together to help ground and settle.

Turn music on again (not the KaT playlist – use a relaxation playlist)

All team members can assist with final BP's. Please ensure the RN signs off on the chart.

RN - Manage headaches, dizziness, nausea, and vomiting as needed. Take these things into account when assessing the BP

Ensure each participant has an escort out - Ensure they are not driving. On the chart, escorting team member ensures final BP is complete and after escorting participant to their ride, **note the time and print your name** confirming tasks are complete.

****Core Support Team Completion of Ceremony**

Once all participants are escorted out, please circle up with the Co-facilitators, RN, and Energy Medicine worker (and include others on the support team when applicable). Take 5 or more minutes to intentionally close your ceremony of holding the participants with love and grace. Consider: silence…smudge…gratitude…check out…however spirit moves you to connect and complete.
