# Supplementary material for: A clinical protocol for group-based ketamine-assisted therapy in a community of practice: the Roots To Thrive model
Source: Front Psychiatry. 2025 Sep 22;16:1568017. doi: 10.3389/fpsyt.2025.1568017 (PMC12498912; doi:10.3389/fpsyt.2025.1568017)
Supplement: Supplementary file 3 [file DataSheet3.pdf]

Roots to Thrive
Ketamine-assisted Therapy (KAT): Medical Procedure

**Purpose:**	The administration of Ketamine to support ketamine-assisted therapy is an innovative and evidence-informed treatment option that combines ketamine with adjunctive psychotherapy. Considering that the field of mental health research has thus far uncovered very few effective pharmacological options, there remain several individuals for whom conventional therapies have failed. Ketamine-assisted therapy is considered a safe and effective treatment option for those who continue to suffer from treatment resistant mental and emotional health conditions.[23]
**Eligibility**	Inclusion Criteria<br>● Aged 19 years or older.<br>● Referral from a primary care provider (or provider with a longitudinal relationship with client) AND a diagnosis of a mental health disorder that has had a poor response to standard treatments, such as: depression (TRD)[1,5] , chronic anxiety[2] , obsessive-compulsive disorder (OCD)[3], suicidality[4], post-traumatic stress disorder (PTSD)[5], End-of-Life Distress, or Substance Use Disorder[6,7,]<br>● Have the willingness and capacity to be an engaged, open-hearted, and consistent participant in a weekly small group.<br>● If parenting, childcare is available for weekly meetings and in-person ketamine sessions.<br>● If self-medicating substances, the impact does not interfere with their capacity to be fully present.<br>● Have reliable internet for weekly virtual meetings via Zoom.<br>● Can attend in-person ketamine sessions on Vancouver Island, British Columbia.<br><br>Exclusion Criteria<br>● Hypersensitivity to ketamine.<br>● Presence of active psychotic symptoms.<br>● Diagnosis of dementia/delirium.<br>● High risk coronary artery disease.<br>● Uncontrolled cardiopulmonary disease/cardiovascular disease/hypertension.<br>● Aneurysm.<br>● History of intracerebral hemorrhage.<br>● Hepatic cirrhosis.<br>● Hepatorenal disease.<br>● Recent changes in medications related to mood disorders.<br>● Pregnant, or if breastfeeding within 11 hours of ketamine administration[29,35]
**Outcomes:**	Varies based on treatment indication. May improve social connections, enhance coping skills, reduce symptoms related to anxiety and depression, and improve emotional health endpoints and/or a return to baseline.

1.0 Required Equipment and Team Capacity

1.1 Equipment

- AED
- Ambu-bag for ventilation with oral airways in various sizes
- Automatic blood pressure cuff
- Pulse oximeter
- EpiPen
- Oxygen source with mask and/or nasal cannula
- Suction

1.2 Team Composition and Capacity Requirements

The continuous review, meticulous editing, and formal endorsement of team intentions and agreements by the RTT team stand as foundational safety measures, ensuring persistent alignment and agreement crucial for the comprehensive and secure care provided throughout RTT-KaT.

Within each small group/Community of Practice (CoP), the collaborative team of professional facilitators plays a pivotal role in establishing a secure, supportive, and trauma-informed environment for participants. Comprising one member with a medical background and another with a therapeutic orientation, this team serves as the cornerstone of the structured process. Their role ensures equitable opportunities for participants to share, fostering authenticity through a timed sharing process that actively encourages vulnerability—a practice modeled by facilitators. Notably, those serving as physicians and somatic energy practitioners in the KaT sessions will periodically float into the weekly CoP small groups to ensure familiarity.

Relational ruptures, whether between participants or team members, are viewed through a transformative lens, redefined as opportunities to navigate tension and consciously engage with attachment tendencies. This approach normalizes challenges, leveraging the opportunity to promote secure attachment through relationships that mirror unconditional positive regard (especially powerful during times of tension).

Facilitators prioritize cultivating secure relationships through energetic attunement, honesty, and authenticity, delivering support in an invitational manner that respects each participant's unique healing pace. The approach remains person-centered, recognizing the diverse factors shaping perceptions, meaning-making, and responses. Grounded in humility and curiosity, facilitators refrain from imposing personal or professional models, responding compassionately to aggressive or harmful behavior as an expression of unmet needs.

Therapeutic relationships are rooted in shared humanity, emphasizing BEing with participants rather than implementing fixes. The facilitation of somatic trauma recovery, wellness, and resiliency further amplifies the impact of these commitments. Emphasizing somatic approaches

and recognizing trauma within the body contribute to holistic healing. Honoring vulnerabilities and strengths is pivotal, fostering trust in inner healing intelligence. The gentle and gradual approach to trauma respects inner protective mechanisms, promoting curiosity and collaboration. Encouraging catharsis and emotional release without pressure respects individualized healing paces, collectively contributing to a holistic, effective therapeutic environment.

Additional medical training and support requirements include assurance that team members:

- Are adequately trained and competent for their roles during therapy sessions with formal training in psychedelic-assisted therapy,
- Have a minimum of 3 years of professional experience with acute mental health conditions,
- Be a licensed or registered healthcare provider or work under the supervision of one,
- Be a clinician with a scope of practice that includes medical monitoring or be paired with another healthcare provider who does,
- Have Basic Life Support (BLS) training, or ensure someone with this training is present during the session,
- Have training on appropriate forms of touch (and other somatic modalities) in a therapeutic environment, or the provider must not engage in touch and should be paired with another healthcare provider with formal training.
- Oversight: The oversight of a medical physician with access to a psychiatrist is required. While their physical onsite presence is not obligatory, their availability on-call is a prerequisite.
- Screening: Initial screening can be conducted by clinicians, including nurses and physicians, ensuring a thorough assessment before the therapy sessions.
- Monitoring: Qualified nurses and physicians can manage monitoring requirements and address any medical needs during KaT sessions. For ketamine administration, one RN or MD skilled in airway management must be present.
- Therapy: Allied healthcare providers with specialized training in KaT are qualified to support all aspects of the KaT experience.
- Somatic Energy Support: While somatic energy providers may not be universally available, their inclusion in the KaT support team is recommended as a holistic measure. With appropriate training, they can also function as therapists in the process.
- Patient Safety Measure: It is imperative to ensure the presence of qualified professionals throughout therapy sessions, avoiding leaving patients alone with a sole provider unless an alternative form of a third witness is arranged.
- Post-KaT: Prior to KaT, participants receive a post-session 'safety sheet' outlining clear guidelines and recommendations for the 24 hours following consumption. The safety sheet includes contact information for at least one team member available on-call for assistance during this post-session period.

This 'Great Tips for Great Sits' document provides general preparation advice for those supporting psychedelic-assisted therapy. Furthermore, before and after sessions, team members are encouraged to participate in clearing practices that protect them and mitigate transference between sessions.

2.0 Intake, Workup and Review, Education and Consent, Physical and Clinical Assessments

Intake: Eligibility Review - Medical Screen

The 'External Screening and Referral Form: Ketamine-assisted therapy for the Treatment of Mood Disorders' will be completed by the referring physician and sent to the KAT team for review. The screening form will include:

- Medical and Psychological Assessment
  - Inclusion criteria (ensuring no treatment exemptions)
  - Demographics
  - Family history
  - Psychiatric & substance use history
- Best Possible Medication History (BPMH)
  - Current medications and clinically relevant past medications (including prior ketamine use)
  - OTC/NHP/Supplements
  - Drug/non-drug allergies
- Baseline Vitals, Weight

Patient Preparation Visit

In addition to this medical preparation, patients must also meet with a KAP trained therapist prior to the first KAP appointment. For KAP therapy preparation requirements, refer to the Psychedelic-assisted Psychotherapy Guidelines.

- Medical history reviewed by a MD to assess suitability
  - Further medical assessments needed if conditions of concern arise (e.g. additional labs or ECG to be done)
- Education session with patient
  - Explanation of ketamine as off-label
  - Discuss expected psychedelic effects and other possible side effects
  - Discuss rationale for dosing
  - Discuss the mechanism of action and duration of action for ketamine
  - Discuss treatment preparation (taking or holding regular meds, eating prior, etc.)
  - Requirement of pre-arranged safe ride home
- Informed Consent for off-label use of ketamine [34]
- Repeat baseline vitals: BP, HR, RR, Oxygen Saturation
- General mental status (calm/anxious/agitated)

2.1 Ketamine Administration: Therapy session with Sublingual (SL) or Intramuscular (IM) ketamine

Patient Visit: First KAP appointment

- Patients should arrive at least 30 minutes prior to ketamine administration to ensure blood pressure is within normal range. If elevated, refer to the Therapeutic Guidelines for relaxation practices. If hypertension continues, pre-emptively treat (refer to PRNs below) and re-take vitals to ensure they are in normal range prior to ketamine administration.
- Recommended dosing: ketamine 100-300mg lozenge sublingual (SL) x1 dose* (dosing to be determined by patient and physician/therapist based on the patient's prior use history and comfort)
  - If initial dose was <300mg, at 12-20 minutes, an additional ketamine sublingual (SL) lozenge can be offered (up to 300mg) if the patient does not feel a noticeable therapeutic effect and BP remains <140/90 or, if baseline on the higher end, <150/95 is acceptable but should consider treating (refer to PRNs below).
  - Maximum first session dose: ketamine 300mg sublingual (SL)
- Alternatively, the first session may also be administered intramuscularly, using Ketamine HCL 0.5mg-1.0mg/kg IM_{30}*, depending on previous experience with ketamine and propensity for nausea.

Patient Visit: Second KAP appointment - Dose escalation/increase based on patient tolerability/sensitivity and clinician assessment from previous session.

- Patients should arrive at least 30 minutes prior to ketamine administration to ensure blood pressure is within normal range. If elevated, refer to the Therapeutic Guidelines for relaxation practices. If hypertension continues, pre-emptively treat (refer to PRNs below) and re-take vitals to ensure they are in normal range prior to ketamine administration.
- Recommended dosing: Ketamine HCl 0.50mg-1.0mg/kg IM. If tolerated well, may increase up to 1.5mg/kg for subsequent sessions.
- Doses may be administered as a single IM injection or as a divided administration with a larger upfront dose, *followed by a secondary top-up dose at 20 minutes. BP must be under <140/90 or, if baseline on the higher end, <150/95 is acceptable but should consider treating (refer to PRNs below).

Patient Visit: Third and Subsequent KAP appointments

- Patients should arrive at least 30 minutes prior to ketamine administration to ensure blood pressure is within normal range. If elevated, refer to the Therapeutic Guidelines for relaxation practices. If hypertension continues, pre-emptively treat (refer to PRNs below) and re-take vitals to ensure they are in normal range prior to ketamine administration.

- Recommended dosing: If ketamine HCl 0.50mg-1.5mg/kg IM.

Clinical monitoring during sessions

- Monitoring during sublingual (SL) ketamine session:
  - [**OPTIONAL] BP at 20 minutes and 60 minutes (pre-emptive of peak serum concentration* which varies based on amount swallowed[17,22])
  - [**OPTIONAL] Sedation Scale at 20 minutes and 60 minutes post-administration
- Monitoring during intramuscular (IM) ketamine session:
  - [**OPTIONAL] BP at 20 minutes (pre-emptive of peak serum concentration[18])
  - [**OPTIONAL] Sedation Scale at 20 minutes post-administration

Side effects: Historically, these transient and self-limiting[23, 27, 28] with treatment and/or interventions rarely required

- Common side effects: Light headedness, elevated blood pressure, elevated heart rate, nausea, agitation
  - PRN medications (below) for Anxiety, HTN, Nausea,
- Rare side effects: Hypotension, arrhythmias, bradycardia

Recommended PRN medications

- Anxiety: Lorazepam 0.5mg SL (rarely used: thorough pre-session education often reduces the need for anxiolytics)
- Hypertension: Refer to the Therapeutic Guidelines for relaxation practices. If hypertension continues, try captopril 3.125-12.5mg PO/SL (can repeat 1-2 hours), or clonidine 0.05-0.2mg PO (can repeat in 1-2hours)[31]
- Nausea: Ginger tablets or Ondansetron 4-16mg PO/IM.
- Headaches: Tylenol 500mg and/or Ibuprofen 200-400mg.

2.2 Post-Procedure: Physical and Clinical Assessment, Workup and Review, Referrals, Discharge

The swallowing of ketamine from SL administration may result in a remote onset of effect in the range of 45–75 minutes later[23]

- BP, HR
- General Mental Status (calm/anxious/agitated)
- Following completion of treatment series, send a consult note to referring care provider (as necessary)
- Complete integration session with therapist (or in group therapy environment) within 48 hours of KAT session

3.0. Definition and Mechanism

- Ketamine-assisted therapy (KAT) is a treatment modality that uses ketamine "off-label" combined with psychotherapy for various chronic and treatment resistant mental health conditions. The current understanding of how ketamine works as an adjunct to therapy relates to its mechanism as an NMDA antagonist, a modulator of the glutamate neurotransmitter system. Ketamine is classified as a dissociative agent, which promotes non-attachment and an expanded awareness of one's ordinary reality.

4.0 Related Standards

- Participant Onboarding Agreements, RTT Team Guidelines, CoP Intentions and Agreements

5.0. Resources

- Informed Consent for Off-Label Ketamine Use
- External Screening and Referral Form: Ketamine-assisted therapy for the Treatment of Mood Disorders

**Dosing for sublingual (SL) ketamine is derived from intranasal dosing as it is considered a comparable route. Intranasal (IN) dosing utilizes up to 84 mg per session[26] —with an approximate 50% absorption, this delivers around 42 mg ketamine systemically. For an equivalent SL dose, PK data gives a bioavailability range as low as 15-25%[23] translating to a SL dose of 175-300mg. This equates to up to 75mg, which falls within 1mg/kg range. The higher % dose would be given first, followed by a second dose as top-up if needed. Note that peak serum concentrations vary from 30-76min based on quantity absorbed sublingually vs swallowed. The bioavailability of IM ketamine is similar to IV ketamine at 93–95%[18,19] SL ketamine absorption is 15-25%[21,23].* Larger upfront doses are chosen to ensure less agitation, which are thought to result from insufficient, sub-therapeutic doses. *Based on a summary of clinical and observational data and expert opinion, the most common adverse drug events in the use of ketamine for mood disorders given IM, IN, & SL were limited to nausea, vomiting and agitation[23,27,28]. Cardiac events (such as arrhythmias) requiring interventions were not reported. Practice standards within anesthesia and analgesia stipulating ECG monitoring are not reflective of the monitoring standards required for non-IV sub-anesthetic doses in the context of mental health treatment. Furthermore, analgesia and anesthesia infusion protocols[16] may include IV lidocaine, which is the key driver for the continuous telemetry requirement. In the absence of lidocaine, ketamine use alone does not warrant such ECG monitoring [23,27,28,29]*
***Based on safety data (Tsang et al, 2023 - awaiting publication) gathered between 2020-2022 in the Roots to Thrive program, it was established that the blood pressure measure taken 20 minutes after administration was no longer necessary.*

6.0 References

1. Chilukuri, H., Reddy, N. P., Pathapati, R. M., Manu, A. N., Jollu, S., & Shaik, A. B. (2014). Acute antidepressant effects of intramuscular versus intravenous ketamine. *Indian journal of psychological medicine*, *36*(1), 71–76. https://doi.org/10.4103/0253-7176.127258
2. Taylor, J., Landeros-Weisenberger, A., Coughlin, C. *et al.* Ketamine for Social Anxiety Disorder: A Randomized, Placebo-Controlled Crossover Trial. *Neuropsychopharmacol.* 43, 325–333 (2018). https://doi.org/10.1038/npp.2017.194
3. Bloch, M. H., Wasylink, S., Landeros-Weisenberger, A., Panza, K. E., Billingslea, E., Leckman, J. F., Krystal, J. H., Bhagwagar, Z., Sanacora, G., & Pittenger, C. (2012). Effects of ketamine in treatment-refractory obsessive-compulsive disorder. *Biological psychiatry,* 72(11), 964–970. https://doi.org/10.1016/j.biopsych.2012.05.028
4. Wilkinson, S. T., Ballard, E. D., Bloch, M. H., Mathew, S. J., Murrough, J. W., Feder, A., Sos, P., Wang, G., Zarate, C. A., Jr, & Sanacora, G. (2018). The Effect of a Single Dose of Intravenous Ketamine on Suicidal Ideation: A Systematic Review and Individual Participant Data Meta-Analysis. *The American journal of psychiatry*, *175*(2), 150–158. https://doi.org/10.1176/appi.ajp.2017.17040472
5. Feder A, Parides MK, Murrough JW, et al. Efficacy of Intravenous Ketamine for Treatment of Chronic Posttraumatic Stress Disorder: A Randomized Clinical Trial. *JAMA Psychiatry.* 2014;71(6):681–688. doi:10.1001/jamapsychiatry.2014.62
6. Ivan Ezquerra-Romano, I., Lawn, W., Krupitsky, E., & Morgan, C. J. A. (2018). Ketamine for the treatment of addiction: Evidence and potential mechanisms. Neuropharmacology, 142, 72-82. doi:10.1016/j.neuropharm.2018.01.017
7. Jones, J. L., Mateus, C. F., Malcolm, R. J., Brady, K. T., & Back, S. E. (2018). Efficacy of Ketamine in the Treatment of Substance Use Disorders: A Systematic Review. *Frontiers in psychiatry*, *9*, 277. https://doi.org/10.3389/fpsyt.2018.00277
8. Loftus RW, Yeager MP, Clark JA, et al. (2010). Intraoperative ketamine reduces perioperative opiate consumption in opiate-dependent patients with chronic back pain undergoing back surgery. *Anesthesiology, 113*: 639–646.

9. Subramaniam K, Akhouri V, Glazer PA, et al. (2011). Intra- and postoperative very low dose intravenous ketamine infusion does not increase pain relief after major spine surgery in patients with preoperative narcotic analgesic intake. *Pain Med,* 12:1276–1283.
10. Williams, J. B. W.; Kobak, K. A. (2008). Development and reliability of a structured interview guide for the Montgomery-Asberg Depression Rating Scale (SIGMA). *The British Journal of Psychiatry*. 192 (1): 52–58. doi:10.1192/bjp.bp.106.032532.
11. Weathers, F. W., Bovin, M. J., Lee, D. J., Sloan, D. M., Schnurr, P. P., Kaloupek, D. G., . . . & Marx, B. P. (2018). The Clinician-Administered PTSD Scale for DSM-5 (CAPS-5): Development and initial psychometric evaluation in military Veterans. *Psychological Assessment, 30*, 383-395. doi:10.1037/pas0000486
12. Blake, D. D., Weathers, F. W., Nagy, L. M., Kaloupek, D. G., Gusman, F. D., Charney, D. S., & Keane, T. M. (1995). The development of a clinician-administered PTSD scale. (PDF) *Journal of Traumatic Stress, 8*, 75-90. doi: 10.1002/jts.2490080106
13. Maier W, Buller R, Philipp M, Heuser I. (1988). The Hamilton Anxiety Scale: reliability, validity and sensitivity to change in anxiety and depressive disorders. *J Affect Disorders, 14*(1):61–8.
14. Kroenke, K., Spitzer, R. L., & Williams, J. B. (2001). The PHQ-9: validity of a brief depression severity measure. *Journal of general internal medicine*, *16*(9), 606–613. https://doi.org/10.1046/j.1525-1497.2001.016009606.x
15. Felitti, V. J., Anda, R. F., Nordenberg, D., Williamson, D. F., Spitz, A. M., Edwards, V., Koss, M. P., & Marks, J. S. (1998). Relationship of childhood abuse and household dysfunction to many of the leading causes of death in adults: the Adverse Childhood Experiences (ACE) study. *American Journal of Preventive Medicine, 14,* 245-258.
16. Island Health. (2014). VIHA IV Monograph: Ketamine.
17. Dore, J., Turnipseed, B., Dwyer, S., Turnipseed, A., Andries, J., Ascani, G., . . . Wolfson, P. (2019).Ketamine assisted psychotherapy (KAP): Patient demographics, clinical data and outcomes in three large practices administering ketamine with psychotherapy. Journal of Psychoactive Drugs: 50th Anniversary: *Psychedelic Research Today*, 51(2), 189-198. doi:10.1080/02791072.2019.1587556Rolan, P., Lim, S., Sunderland, V., Liu, Y., & Molnar, V. (2014). The absolute bioavailability of racemic ketamine from a novel sublingual formulation. *British journal of clinical pharmacology*, *77*(6), 1011–1016. https://doi.org/10.1111/bcp.12264
18. Clements JA, Nimmo WS, Grant IS. (1982). Bioavailability, pharmacokinetics, and analgesic activity of ketamine in humans. *J Pharm Sci.* 71:539-42.
19. Yanagihara Y, Ohtani M, Kariya S, Uchino K, Hiraishi T, Ashizawa N, et al. (2003). Plasma concentration profiles of ketamine and norketamine after administration of various ketamine preparations to healthy Japanese volunteers. *Biopharm Drug Dispos.* 24:37-43.
20. Peltoniemi, M. A., Hagelberg, N. M., Olkkola, K. T., & Saari, T. I. (2016). Ketamine: A review of clinical pharmacokinetics and pharmacodynamics in anesthesia and pain therapy. Clinical Pharmacokinetics, 55(9), 1059-1077. doi:10.1007/s40262-016-0383-6
21. Chong, C., Schug, S. A., Page-Sharp, M., Jenkins, B., & Ilett, K. F. (2009). Development of a Sublingual/Oral formulation of ketamine for use in neuropathic pain: Preliminary findings from a three-way randomized, crossover study. *Clinical Drug Investigation, 29*(5), 317-324. doi:10.2165/00044011-200929050-00004
22. Chong, C., Schug, S., Page-Sharp, M., & Ilett, K. (2006). Bioavailability of ketamine after oral or sublingual administration. *Pain Medicine*, 7(5), 469-469. doi:10.1111/j.1526-4637.2006.00208_8.x
23. Dore, J., Turnipseed, B., Dwyer, S., Turnipseed, A., Andries, J., Ascani, G., . . . Wolfson, P. (2019). Ketamine assisted psychotherapy (KAP): Patient demographics, clinical data and outcomes in three large practices administering ketamine with psychotherapy. Journal of Psychoactive Drugs: 50th Anniversary: *Psychedelic Research Today*, 51(2), 189-198. doi:10.1080/02791072.2019.1587556
24. Bouso, J. C., Pedrero-Pérez, E. J., Gandy, S., & Alcázar-Córcoles, M. Á. (2016). Measuring the subjective: Revisiting the psychometric properties of three rating scales that assess the acute effects of hallucinogens. *Human Psychopharmacology: Clinical and Experimental, 31*(5), 356-372. doi:10.1002/hup.2545
25. Nour, M. M., L. Evans, D. Nutt, and R. L. Carhart-Harris. 2016, June 14. Ego-dissolution and psychedelics: Validation of the Ego-Dissolution Inventory (EDI). *Frontiers in Human Neuroscience* 10:269. doi: 10.3389/fnhum.2016.00269.eCollection2016.
26. Spravato Monograph. (2019). Retrieved from https://www.accessdata.fda.gov/drugsatfda_docs/label/2019/211243lbl.pdf
27. Loo, C. K., Gálvez, V., O'Keefe, E., Mitchell, P. B., Hadzi-Pavlovic, D., Leyden, J., . . . Glue, P. (2016). Placebo-controlled pilot trial testing dose titration and intravenous, intramuscular and subcutaneous routes for ketamine in depression. *Acta Psychiatrica Scandinavica,* 134(1), 48-56. doi:10.1111/acps.12572
28. Collins, K., J. Murrough, A. Perez, D. Reich, D. Charney, and S. Mathew. (2010). Safety and efficacy of repeated-dose intravenous ketamine for treatment-resistant depression. *Biological Psychiatry* 67 (2):139–45. doi:10.1016/j.biopsych.2009.08.038.
29. KAP BC. Ketamine Clinic for Mood Disorders. (2020). Under Review by KAP BC.
30. Kheirkhah, F., Tayyebi, G., Rabiee, S. M., Moghadamnia, A. A., & Bijani, A. (2018). Efficacy of different doses of ketamine as a bolus in major depressive disorder. *Caspian journal of internal medicine*, *9*(3), 220–227. https://doi.org/10.22088/cjim.9.3.220
31. Krahn, K. (2016). Hypertenstion Urgency: Consideration for Management. *RX Files.* Retrieved from: https://www.rxfiles.ca/rxfiles/uploads/documents/Hypertensive-Urgency-Management.pdf
32. ASA House of Delegates (2019). ASA Physical Status Classification System. Retrieved from https://www.asahq.org/standards-and-guidelines/asa-physical-status-classification-system
33. Spitzer RL, Kroenke K, Williams JBW, Löwe B. A Brief Measure for Assessing Generalized Anxiety Disorder: The GAD-7. *Arch Intern Med.* 2006;166(10):1092–1097. doi:10.1001/archinte.166.10.1092
34. The Standing Senate committee on Social Affairs, Science and Technology Senate (2014). Prescription Pharmaceuticals in Canada: Off-label Use. Retrieved from https://sencanada.ca/content/sen/committee/412/soci/rep/rep05jan14-e.pdf
35. Briggs, G. G., Freeman, R. K., & Yaffe, S. J. (2016). Drugs in pregnancy and lactation: A reference guide to fetal and neonatal risk. Place of publication not identified: Wolters Kluwer Health.
