# Supplementary material for: A clinical protocol for group-based ketamine-assisted therapy in a community of practice: the Roots To Thrive model
Source: Front Psychiatry. 2025 Sep 22;16:1568017. doi: 10.3389/fpsyt.2025.1568017 (PMC12498912; doi:10.3389/fpsyt.2025.1568017)
Supplement: Supplementary file 4 [file DataSheet4.pdf]

Week One

Foundations and Intentions

Week 1 is delivered in large group format only (small groups begin in Week 2) and is designed to promote a sense of coherence through:

- Facilitating meaning-making and participant buy-in by explaining the purpose and structure of each component of the 12-week program.
- Introducing core concepts of resilience—what it is, the personal qualities that contribute to it, and how the program is designed to cultivate those qualities.
- Helping participants understand how mental health challenges often arise as adaptive responses to disconnection and maladaptive environments, thereby normalizing their experiences.
- Instilling confidence in their innate capacity for healing and in the supportive design of the program, including the role of mindfulness, somatic regulation practices, community framework, and access to a multidisciplinary team.

<table>
<tr><th>Section</th><th>Purpose</th><th>Key Actions</th></tr>
<tr><td>Welcome & Opening
5 min</td><td colspan="2">Ceremonially open the 12-week journey and create a grounded, collective healing environment. Elder Geraldine Manson opens the space, inviting participants into presence and calling in seen and unseen supports for individual and collective healing.</td></tr>
<tr><td>Logistics
5 min</td><td colspan="2">Team introductions to promote familiarity and trust. Share agenda and program structure. Introduce shared intentions and agreements. Mention access to session recordings and informational video outlining program structure.</td></tr>
<tr><td>Coming to Know
140min</td><td colspan="2">Introduce the foundational concepts of the program to foster understanding, meaning-making, and participant engagement.

Wisdom teaching by Elder Geraldine Manson. Dr. Shannon introduces resilience theory and research. Dr. Crosbie contextualizes psychiatric patterns as adaptive. Phil provides a detailed overview of CoP structure and milestones. Conclude with Q&A.</td></tr>
<tr><td>Pause Practice
5 min</td><td colspan="2">Support nervous system regulation and introduce participants to somatic grounding techniques.</td></tr>
</table>

Week Two

Supporting the Body to Hold Compassionate Space for 'what is'
EFT and Somatic Energy Modalities

Week 2 curriculum is focused on somatic awareness and energy regulation—paired with the initiation of small group sessions, fosters congruence by helping participants align their internal experiences with external expression. Through intentional agreements and authentic sharing, participants begin to cultivate a sense of coherence by finding meaning, manageability, and connection within a relational and intentional structure.

Large Group (Opening Session)

Section	Purpose	Key Actions
**Welcome & Opening** 5 min	Create an inclusive and welcoming atmosphere by sharing an inspirational reading or prayer to invite the group into presence and our shared intentions.	
**Logistics** 5 min	Share details to make the process more predictable. Address anonymous questions and feedback relevant to the larger group from week 1 (and encourage ongoing use of anonymous feedback link). Promote buy-in for shared intentions and agreements to unfold in small groups.	
**Coming to Know** 15 min	Dr. Pam introduces the therapeutic role of KaT, outlines the required ketamine preparation session, and bridges into the value of somatic energy practices in preparing for non-ordinary states.<br><br>Helen and Darlus provide an overview of somatic regulation techniques, guiding participants through experiential practices such as Emotional Freedom Technique (EFT) to support grounding and nervous system regulation.<br><br>*KaT basics and Energy Medicine. Put this in the chat box to help with buy-in (research informed!): EFT Research: The Science of Tapping (https://www.efttappingtraining.com/eft-research/)*	
**Pause Practice** 5 min	Cultivate somatic awareness by shifting from cognitive processing to embodied presence—practicing self-regulation within the safety of group co-regulation, while expanding participants' repertoire of nervous system regulation tools.	

Small Group (Breakout Rooms)

Section	Purpose	Key Actions
**Facilitator Opening**<br><br>2 minutes	Reinforce shared intentions, including inclusion and honoring of diversity, flowing from unconditional positive regard (UPR).<br><br>Reinforce the purpose of shared agreements (to be co-created in this session)	Facilitators set the tone, welcoming participants with warmth and authenticity.<br><br>Reinforce unconditional positive regard (UPR) and safety.<br><br>You are welcome to reinforce your version of the antiracism statement (template provided below) if you are feeling called to do so.

<table>
<tr><th>Section</th><th>Purpose</th><th>Key Actions</th></tr>
<tr><td>Short Check-In
15 minutes</td><td>Build connection and self-awareness by speaking from the body. Facilitators share first to model.
Keep it concise (30–60 seconds/person).</td><td>Turn your focus to your body.
Name 1-2 physical sensations, 1-2 emotions – practicing naming without feeling the need to explain.
How I came to be on this land/place OR a place I like to be/sit in silence.
Tag another.</td></tr>
<tr><td>Logistical Round
2-3 minutes</td><td colspan="2">Speak to medicine of unconditional positive regard as a core medicine to promote resilience, which is reflected in our capacity to show up authentically. We can't do that unless our bodies believe they are worthy of it. We learn that by receiving it from others. Let's become mirrors for this medicine. Review and adopt group intentions and agreements and address and make any changes the group agrees to (template below)

Emphasize the importance of attending all four initial CoP sessions to build group relationships, trust, and security for healing. Missing sessions may impact participation in KaT Session 1, as safety requires connection and familiarity.
If not covered in large group logistics: A follow-up email will include videos on energy work, touch during Ketamine sessions, and Pam's Ketamine presentation. Please review these in preparation for the KaT Q&A session prior to Week 4's CoP.</td></tr>
<tr><td>Long Check-In
3–4 min/participant</td><td>Remind the group to stay present and avoid historical storytelling. Focus on the current moment (e.g., "What am I experiencing right now as I remember X?"). Emphasize listening and speaking from the body.

Facilitators share first to set the tone.
Co-facilitators - take turns offering BRIEF compassionate witness after each sharing - Tell the group what you are doing and that they will begin to participate similarly next week.
The timekeeper will need to keep everyone on track of time, as it can go by fast this round.</td><td>Before you share, please take one NOTICING BREATH (where you notice what is happening in your body)

What in your life called you to this journey?
Or what is your intention (hope to move toward) for this journey?
What are your fears and hopes about participating in this program? What physical sensations come into your awareness as you feel these fears and hopes?

OR - Share what you want the group to hear about where you are right now.
Tag another.</td></tr>
<tr><td>Integration Practice</td><td colspan="2">Making the conscious unconscious through deliberate practice. By trying new tools, we develop a bigger tool belt.</td></tr>
</table>

Section	Purpose	Key Actions
5–10 minutes	Practice listening to the body as you explore new ways of connecting with and comforting the body.<br>Establish Buddy Systems to expand opportunities to practice CoP skills outside the weekly sessions.<br>Provide clear instructions for when and how participants can seek support between sessions.	
**Check-Out**<br>5–10 minutes	Close the session with reflection and intentional release.<br>*Facilitators share first to set the tone.*<br>*Keep it concise (30–60 seconds/person).*	**Feel and name 1-2 physical sensations.**<br>**Feel and name 1-2 emotions.**<br>**How will you use the tools offered thus far, such as EFT, 4/7/8 (or other breath technique), etc.**<br>Tag another.

Notes to the facilitator(s)

Model vulnerability and self-compassionate boundaries to support authenticity within the group. Express your emotions openly while making it clear you have external resources and do not need caretaking. Encourage participants to engage with vulnerability gradually, listening to their bodies. This process may initially feel messy but offers valuable practice for the group.

Encourage the group to slow down, reflect, and feel their responses before sharing. A deep breath can help, and participants should aim to push gently beyond surface-level answers, staying attuned to their comfort and body signals.

Normalize post-group feelings of vulnerability, such as shame, regret, or fear, as well as the impact of witnessing others (e.g., feeling triggered or lingering emotions). Continue to remind about and encourage self-care practices/rituals to calm, release, close / transition to sleep.

Anti-Racism Statement:

(This is not intended as a script to be read, but rather principles to draw from. Please make it your own)

At Roots to Thrive, we are committed to addressing systemic racism. While we work toward equality and anti-racism, we acknowledge we work within health and education systems reflect aspects of systemic racism. These systems are actively addressing racism, as we are. We welcome feedback on anything that feels discriminatory, marginalizing, or oppressive. Your input helps us improve cultural safety and humility in our program.

You may hear us acknowledge colonization, racism, and the traditional, unceded territories of Indigenous peoples. We honor the Truth and Reconciliation Commission Calls to Action and respect the original stewards of this land. This is not to create guilt or shame, or a sense of otherwise less-than, but to inspire healing and understanding for everyone, grounded in the history of the land we share.

Your feedback is welcome in any form, including an anonymous feedback form that will be provided soon.

Roots to Thrive Community of Practice (CoP)

Intentions and Agreements

Each group should adapt these intentions and agreements to ensure collective buy-in and co-create a space that feels safe, inclusive, and supportive for all. This document will be developed during the first 2-3 meetings and reviewed mid-way through the program.

Intentions of Our Roots to Thrive Circle:

1. To co-create a community that practices compassion, caring and unconditional positive regard for ourselves and each other.
2. To practice expressing ourselves authentically and honestly, rather than saying what we think others want to hear.
3. To develop our ability to comfort and calm ourselves in moments of suffering (stress).
4. To contribute to feelings of safety by honoring vulnerability.
5. To gently step beyond our comfort zones.
6. To experience authentic connection to ourselves and to one another.

Agreements for participation in our Roots to Thrive Circle: Each member of our Circle is asked to commit to these agreements.

1. I agree to protect the privacy (confidentiality and anonymity) of group members. Outside of this circle I will only speak about my own experience, and not that of others. When possible, I will use a private space for the group Zoom meetings; if not I'll use headphones, and ensure my screen isn't visible to others, so that only I see and hear the group.
2. I agree to co-create a safe 'container,' where we practice giving and receiving unconditional positive regard and honour diversity.
3. I agree to practice listening compassionately, and to share from a place of "I want to hear" rather than "I want to fix".
4. I agree to refrain from analyzing or redirecting focus from someone's share or healing process to myself, ensuring the space remains centered, supportive, and respectful of the speaker's experience.
5. I agree to not interrupt or cross-talk, so everyone feels free to share without judgment.
6. I agree to be on time for meetings. If I need to be late, I will text or call one person to let them know approximately when I will arrive (no need to explain why).
7. I agree to attend the first 4 meetings, and at least 3 out of every 4 meetings thereafter.
8. If unable to attend a meeting, I agree to email the group with a check in as to how my week has been, just as I would during a meeting (e.g. practicing vulnerability, responding to that week's reflection questions).
9. If I use substances, I agree to not use an amount or at a time that would interfere with my ability to participate in group.
10. If I am aware of being at risk of harming myself or others, I will seek help.
11. I agree that if I have a safety concern for a group member or a group member shares a concern for their own safety or the safety of another with me, I will bring this information to a facilitator or team member for help. This is necessary to enable each of us to remain focused on our healing, to prevent moral distress, and for those of us in the helping professions, to satisfy any legal reporting requirements.
12. I understand that this community of practice is intended as a support but is not a replacement for any other therapy or healing work I will continue doing.
13. Because feelings of intimacy, warmth and affection can happen in these groups, I agree to keep all relationships within the group platonic and not pursue any romantic feelings. This is important to protect the sense of safety for everyone in the group.
14. I understand that I am not to ask other members of the group to hold any secrets.

15. If I leave the group, I will attend one final meeting to let everyone know that I will be leaving, allowing for good-byes and closure.
