# Supplementary material for: A clinical protocol for group-based ketamine-assisted therapy in a community of practice: the Roots To Thrive model
Source: Front Psychiatry. 2025 Sep 22;16:1568017. doi: 10.3389/fpsyt.2025.1568017 (PMC12498912; doi:10.3389/fpsyt.2025.1568017)
Supplement: Supplementary file 5 [file DataSheet5.pdf]

Roots to Thrive

Activating our innate, individual & collective human capacity for healing and resilience

Core Curriculum: Living Version 14

A Note about Sharing

Dear readers, the knowledge shared here reflects a joint endeavor with many contributors, guided by Dr. Shannon Dames as managing author. Our commitment lies in upholding relational knowledge, which entails recognizing the source from which it emerged, embracing its dynamic nature, and the spirit of reciprocity. Feel free to adopt teachings that resonate with you, weaving them personally as you see fit. To maintain knowledge integrity and honor sources, we kindly ask that you seek formal permission before distributing any written material.

Practical Aspects

The guide starts by helping by explaining the program's theory and research. For some of us, having this information prepares us to engage fully - mind, body, spirit. And we recognize that for others, this content may not resonate and may in fact limit be a distraction. As with everything - take what you need, and leave what you don't; feel free to skip past, skim over, or dive in. The guide moves from research and theory to practical application, with exercises throughout that aim to help you integrate and embody.

A Living Curriculum. The Roots to Thrive curriculum provides of way of BEing in the world. It's designed to help our collective healing journey, reminding us of what it means to be human in a complex and colonized world. Drawing from Western medicine, Indigenous knowledge, holistic practitioners, and participants, the content here continues to evolve. Our aim is for this resource to stay relevant amidst the changing times we're in.

Acknowledging ingrained racism and moving toward equity. As we advance towards genuine equality and anti-racism, we acknowledge the pervasive systemic racism ingrained within the institutions of healthcare and education. Recognizing our own upbringing within these systems, we admit to the presence of biases. Collectively, we're committed to uncovering and addressing these blind spots, actively inviting diverse perspectives to help us counteract discriminatory and marginalizing practices. This commitment aligns with our ethical standards and the pursuit of best practices. We genuinely welcome and value feedback to help us in this important endeavor.

Reading approach. You can read the guide at your own pace, from start to finish. Alternatively, if you are following the 12-week Roots to Thrive curriculum, you can navigate through the content by clicking on the designated links associated with each week in the content legend provided below the table of contents.

Reading efficiencies. The reference to Roots to Thrive is used interchangeably with RTT. Additionally, to make reading smoother, references will be provided within the text the first time they're used, removing the necessity for repeated citations. Also, important terms will be marked in bold when they're introduced initially, followed by their definition.

Optional Pre-Program/Reading Preparation

If you find yourself reading this, you have already taken the initial step on your journey, and preparation is underway. In the days and weeks leading up to the program, it can be helpful to consider the qualities essential to you, separate from the conditioning that occurs as we grow up in a colonized world. The following suggestions provide examples for those who feel inspired and safe to explore them (all are optional).

Awareness of the body. Pay mindful attention to sensations and emotions, allowing them without judgment or the need for immediate change.

Awareness of our essence. If you have access to childhood photos, see if you can find some that represent a time when you can see more light in your eyes. Is there an age where the spark seems harder to

notice? When does the smile change from one that feels authentic to one that feels 'put on'? Alternatively, ask someone who knew you in childhood about the qualities that stood out to them compared to other kids. What did you love to do? When were you most joyful?

Awareness of what inspires and irritates. If looking back feels unsafe, consider those in your current life whom you deeply admire. Those we admire often embody qualities within us waiting to manifest. Conversely, pay attention to who irritates you; these individuals may reflect parts of yourself that were unconsciously disowned in response to past experiences, where the fear of rejection became too overwhelming to let them surface. We all have these disowned parts of ourselves.

As we remember who we are, and as our sense of connection and security grows, that which we are not will gradually fall away. We will move at the pace of trust, developed as we learn to walk each other home—each step forward is an act of reconciliation with our bodies, with our communities, and the Spirit that connects us to all that is.

Table of Contents

Legend: Bridging 12-Week Program with Guide Content *7*

PART 1: THEORETICAL FOUNDATIONS *10*

Why Develop Your Roots to Thrive (RTT)? **10**

The Roots to Thrive Journey **10**

The Roots of Roots **13**

Congruence **13**

Sense of Coherence **16**

How Sense of Coherence and Congruence Inform our Ability to Thrive **17**

The Theory of Unmet Needs 19

Intertwining Root Systems: Nutrition for our Nervous Systems **19**

Breaking Free of Patterns **22**

Slow and Steady with an Inside Approach **23**

PART 2: DEVELOPING ROOTS TO THRIVE: A RECONCILIATION PROCESS *25*

Meaning Making: Reconciling with the Mind *26*

Understanding the Roots of Stress **26**

Pause to Reflect: What is stress to you? 27

Pause to Practice: Interrupting the Stress Response 28

Managing Stress: The Role of Co-Regulation and Somatic Reminders **28**

Incorporating R.A.I.N.(S) **29**

Pause to Reflect: Applying R.A.I.N.(S) 30

Pause to Practice: Cultivating Presence with Breath Awareness 30

Present Day Stress can be the Voice of Past Trauma: What are you Hearing? **32**

Pause to Practice: Compassion for the Nervous System 33

Pause to Practice: Clearing Stuck Energy through Expressive Writing 34

Managing “Weather” with Secure and Healthy “Roots” **35**

Pause to practice: Storm Watching 35

Securing our Roots in Community: Reconciling with Others *38*

Relational Attachment and Coping Styles **40**

Pause to Reflect: What are your attachment tendencies? 41

Attachment Antidotes: Navigating Relationships with Choice **42**

Pause to Practice: Reflecting on Your Attachment Antidotes 42

Pause to Strengthen: Cultivating Secure Attachment 43

Healing in a Community of Practice Structure **43**

Pause to Reflect: Who do you feel emotionally safe with? 45

Pausing to Strengthen: The Check-In 46

Pausing to Strengthen: The Buddy System 46

Re-friending the Body: Reconciling with Emotional Messengers......48

Differentiating Signal from Noise......48
Pause to Calm the Noise: The 4/7/8 Breath......49

Sensations and Emotions of the Body......50
Pause to Reflect: The Guest House......50

Non-Attachment......51
Pause to Reflect: What would it look like to Welcome and Respond to Cues from the Body?......52
Pause to Strengthen: Sensations as Messengers......53

Emotional Rhythm and Emotional Regulation......53

Emotions as Phone Calls......53
Pause to Reflect: How do you respond to emotional guests?......54

Expanding Beyond the Physical: Energy Medicine......55
Pausing to Strengthen: Emotional Freedom Technique......55

Unconditional Positive Regard (UPR)......57
Pause to Reflect: How do we Embody UPR?......57

Self-Compassion: UPR Directed Inwardly......58
Pause to Reflect: What story of self are you living by?......60
Pause to Reflect: How would you treat a dear friend?......61
Pause to Strengthen: Transitioning from Identifying with to Caring for......62
Pause to Strengthen: Self-Holding with the Butterfly Hug......64

Compassionate Witnessing: Outward UPR......64
Pause to Practice: From Fixing to Deep Listening......67

Fueling our Spiritual Pilot Light......68
Ritual: Bridging DOing and BEing......68

Inner Healing Intelligence......69
Pause to Reflect: Coming into Relationship with your Inner Healer......69
Pause to Practice: Inner Healing Intelligence and Parts of the Self......69

Liminal Space and the Window of Tolerance......70
Pause to Reflect: Recognizing Liminal Spaces......73

Soothing the Body......74
Soothing in the Gap Between Awareness and Embodiment......75
Pause to Strengthen: Soothing with Breath......75
Soothing with Touch (Somatic resourcing)......75

Clearing out the Sticky Bits......76
Transmuting 'Stuckness' with Gratitude......76
Pause to Strengthen: Transmuting with Gratitude......78
Letting Go: *For*-Giving: What are you *Giving* your Freedom *For*?......79
Pause to Reflect: What are you giving your freedom for?......80
Pause to Strengthen: Transmuting with Forgiveness......80

Significant Thing(s) and Optimistic Reframes......82
Pause to Reflect: What does Purposeful Action Look Like?......83
Pause to Reflect: Transmuting with an Optimism (Empowering!) Reframe......85

The Power of Humor in Coping and Resilience......86
Pause to Practice: A letter to myself......86

Generativity: Your Way Forward ***86***
Pause to Practice: Calling in my Resources 87
Living Your Calling: The North Star Within ***91***
Pause to Practice: Calling Statement – A Bridge to your North Star 91
Appendix A: Emotional Descriptors ***94***
Appendix B: Psychedelic Medicines as Catalysts for Healing ***95***
Appendix C: Intention Setting and Identifying Your Pillars of Strength ***98***
Appendix D: Ketamine-assisted Therapy ***100***
Appendix E: Psilocybin-assisted Therapy ***109***
Appendix F: Therapeutic Touch and Energy Medicine During Your Medicine Session ***113***
Appendix G: Music as Medicine ***114***
References ***117***

Legend: Bridging 12-Week Program with Guide Content

This curriculum guide can be read at your own pace, from start to finish. Alternatively, if you are following the 12-week RTT curriculum, you can navigate through the content by clicking on the designated links associated with each week, enabling you to follow along with the 'coming to know' provided at the beginning of each community of practice.

**Week / Content**	**Helpful Readings Reference**<br>*Click on the title below to go directly to the content area*
**Week One:** Foundations and Intentions	***PART 1: THEORETICAL FOUNDATIONS****Error! Reference source not found.*<br>***Understanding the Roots of Stress***<br>***Managing Stress: The Role of Co-Regulation and Somatic Reminders***<br>***Healing in a Community of Practice Structure***<br>***Pausing to Strengthen: The Check-In***<br>Pause to Practice: Cultivating Presence with Breath Awareness<br>**Pause to Calm the Noise: The 4/7/8 Breath**
**Week Two:**<br>Supporting the Body to Hold<br>Compassionate Space for 'what is'	**Expanding Beyond the Physical: Energy Medicine**<br>**Sensations and Emotions of the Body**<br>Appendix A: Emotional Descriptors<br>Present Day Stress can be the Voice of Past Trauma: What are you Hearing?<br>Pause to Practice: Compassion for the Nervous System<br>Pausing to Strengthen: Emotional Freedom Technique<br>Pause to Practice: Clearing Stuck Energy through Expressive Writing<br>Appendix A: Emotional Descriptors
**Week Three:**<br>Mindful & Heartful Listening	**Unconditional Positive Regard (UPR)**<br>Compassionate Witnessing: Outward UPR<br>**Pause to Practice: From Fixing to Deep Listening**<br>**Pause to Strengthen: Self-Holding with the Butterfly Hug**
**Psychedelic-assisted Therapy Preparation**<br>Appendix B: Psychedelic Medicines as Catalysts for Healing<br>Appendix C: Intention Setting and Identifying Your Pillars of Strength<br>Appendix D: Ketamine-assisted Therapy<br>Appendix E: Psilocybin-assisted Therapy<br>Appendix F: Therapeutic Touch and Energy Medicine During Your Medicine Session<br>Appendix G: Music as Medicine	
**Week Four:**<br>Inner Healing Intelligence & RAIN(S)	**Incorporating R.A.I.N.(S)**<br>Pause to Reflect: Applying R.A.I.N<br>Pause to Practice: Cultivating Presence with Breath Awareness<br>Differentiating Signal from Noise

	Inner Healing Intelligence<br>Pause to Reflect: Coming into Relationship with your Inner Healer<br>Pause to Practice: Inner Healing Intelligence and Parts of the Self
**Week Five:**<br>Liminal Spaces and the Window of Tolerance	**Liminal Space and the Window of Tolerance**<br>Pause to Reflect: Recognizing Liminal Spaces<br>**Managing "Weather" with Secure and Healthy "Roots"**<br>**Pause to practice: Storm Watching**<br>**Soothing the Body**<br>Soothing in the Gap Between Awareness and Embodiment<br>**Pause to Strengthen: Soothing with Breath**<br>**Soothing with Touch (Somatic resourcing)**
**Week Six:**<br>Emotional conditioning and pathways of expression (significant things)	***Appendix A: Emotional Descriptors***<br>Sensations and Emotions of the Body<br>Pause to Reflect: The Guest House<br>Emotional Rhythm and Emotional Regulation<br>***Emotions as Phone Calls***<br>***Pause to Reflect: How do you respond to emotional guests?***
**Week Seven:**<br>Letting Go and Awakening to Purpose	**Non-Attachment**<br>Pause to Reflect: What would it look like to Welcome and Respond to Cues from the Body?<br>Pause to Strengthen: Sensations as Messengers<br>Letting Go: *For-Giving*: What are you *Giving* your Freedom *For*?<br>Pause to Reflect: What are you giving your freedom for?
**Week Eight:**<br>Relationships and attachment tendencies	**Securing our Roots in Community: Reconciling with Others**<br>**Relational Attachment and Coping Styles**<br>**Pause to Reflect: What are your attachment tendencies?**<br>Attachment Antidotes: Navigating Relationships with Choice<br>Pause to Practice: Reflecting on Your Attachment Antidotes<br>Pause to Strengthen: Cultivating Secure Attachment
**Week Nine:**<br>My Journey to Self-Compassion	**Self-Compassion: UPR**<br>Error! Reference source not found.<br>Error! Reference source not found.<br>**Significant Thing(s) and Optimistic Reframes**<br>**Pause to Reflect: What does Purposeful Action Look Like?**<br>Pause to Reflect: Transmuting with an Optimism (Empowering!) Reframe<br>The Power of Humor in Coping and Resilience

**Week Ten:**<br>Acknowledging our interconnectedness - co-creating community	**Intertwining Root Systems: Nutrition for our Nervous Systems**Error! Reference source not found.<br>**Fueling our Spiritual Pilot LightFueling our Spiritual Pilot Light**<br>Ritual: Bridging DOing and BEing<br><br>Pause to Practice: A letter to myself
**Week Eleven:**<br>Generativity - Your Way Forward	**Clearing out the Sticky Bits**<br>Pause to Strengthen: Transmuting with Gratitude<br>Pause to Practice: Calling in my Resources<br>Generativity: Your Way Forward
**Week Twelve:**<br>Locating your North Star	**Living Your Calling: The North Star Within**<br>Pause to Practice: Calling Statement – A Bridge to your North Star

PART 1: THEORETICAL FOUNDATIONS

Why Develop Your Roots to Thrive (RTT)?

Distinct from the traditional hierarchical model, where the expert is responsible for the cure, our view is that persons are inherently whole, with an innate capacity for healing. We are born whole, as thriving beings.

The role of RTT facilitators, who have come through the program as participants themselves, are to assist in bringing awareness to patterns that create suffering. Symptoms that may be labelled a 'disorder', are often natural and logical consequences of upstream mediators (i.e. cultural conditioning, generational trauma, and our relational context).

Many of us learn early on that we are not good enough as we are and come to believe this is true. We are told repeatedly that the things we DO are what makes us whole, and worthy of love and belonging.

DOing = worth.

To get the love and belonging we need, we look around us to find out how to act, and how to present ourselves in the world. After trading in enough pieces of our real selves to attain the approval of others, we eventually lose touch with who we are - our very spirit or BEing - that fuels our passion for life. Our body then sends out distress signals, alerting us that we are out of tune with our real selves. These signals often manifest as anxiety and depression.

Geraldine Manson, an honored Roots to Thrive Elder, refers to our life force, the spark of life within, as our **pilot light**. To come back to and tend our pilot light we must be rooted deeply in the belief that we are indeed worthy just as we are. As such, the Roots the Thrive journey is not to learn to be human, it is remembering our inherent wholeness. Once we feel into this wholeness, we come to remember who we authentically are.

The Roots to Thrive Journey

The Roots to Thrive journey is full of grace. It's a process of remembering who we are, surrounding ourselves with people who can remind us of our inherent worth when we forget. It's about finding our way home, as beautifully conveyed by Toko-pa below.

> *Remembering Ourselves Home*
> *by Toko-pa Turner*
>
> *For the rebels and the misfits, the black sheep and the outsiders. For the refugees, the orphans, the scapegoats, and the weirdos. For the uprooted, the abandoned, the shunned and invisible ones.*
> *May you recognize with increasing vividness that you know what you know.*
> *May you give up your allegiances to self-doubt, meekness, and hesitation.*
> *May you be willing to be unlikeable, and in the process be utterly loved.*
> *May you be impervious to the wrongful projections of others, and may you deliver your disagreements with precision and grace.*
> *May you see, with the consummate clarity of nature moving through you, that your voice is not only necessary, but desperately needed to sing us out of this muddle.*
> *May you feel shored up, supported, entwined, and reassured as you offer yourself and your gifts to the world.*
> *May you know for certain that even as you stand by yourself, you are not alone.*

Moving at the speed of trust, we engage in a process of reconciliation, where the mind, body, and spirit come back into harmony. From this place of wholeness, we realign with our desires, values, and passions. We move from DOing in order to feel accepted and valued, to feeling safe enough to spontaneously BE our true, whole selves in the world.

BEing > DOing

As we securely attach to our ‘real’ selves, we become unburdened by the self-conscious (and often critical) mind. This enables a sense of compassion with our authentic selves. We naturally behave in ways that are consistent with who we are and what really matters to us, navigating our roles and relationships while still firmly grounded in our authentic self.

"You start at your darkness, and work your way back to your own light," (patient partner, cohort 5)

The primary medicine on the Roots to Thrive journey is **unconditional positive regard**, a term coined by Carl Rogers, an influential psychologist (Rogers, 1959). It means simply to accept another person for who they are. Positive regard is not withdrawn if the person makes a mistake or shows up in an irritating way. It means respecting each other as human beings with free will, and operating under the assumption that we are all doing the best that we can with the tools we have. Unconditional positive regard involves knowing we all carry varying resources and capacities that inform the conscious and unconscious choices we make.

Unconditional positive regard recognizes that we all have unique resources and abilities which impact our ability to consciously and with intention *respond* to life’s challenges, rather than being at the mercy of automatic, unconscious *reactions.* When we automatically react - our preschool child starts drawing on the wall and we, without thinking, yell at them to stop - we often experience feelings of shame, which further disconnects us from ourselves and others. When there is no pause in life, no chance to take a moment to think through how we would like to respond, it can feel like we have no choice; like we are at the mercy of our immediate reactions. Genuine choice can only come from our conscious awareness of what is happening. This understanding helps us approach each other with empathy, fostering connection and growth on our collective journey.

Fostering trust in ourselves and in the world: We don’t live in a society where we are taught how to receive or give unconditional positive regard, and very few of us grew up in an environment where this was demonstrated. This has significant consequences because unconditional positive regard is required to experience **secure attachment**. Secure attachment is described in detail later, but briefly stated it represents our sense of security in the world. Those who are more securely attached tend to have higher self-esteem and navigate the world with more confidence and less fear. Secure attachment, in turn, shows up as a wide range of personal resilience factors (Dames, 2022).

Secure attachment, closely tied to a core Roots to Thrive principle known as **sense of coherence** (Antonovsky, 1979), describes our sense of belonging, safety, security, and resourcefulness. With secure attachment, we navigate the world more confidently and with reduced fear. Constructing secure attachment involves relationships mirroring unconditional positive regard, fostering trust in ourselves and the world.

A relational journey: We do this work together. Just as many of our wounds are formed in relationship, they are also most often healed in relationship—through connection, shared presence, and the rewriting of stories that may have once defined or limited us.

To take on a more secure and integrated way of being, we practice embodying and mirroring unconditional positive regard within a safe and supportive community. Over time, the compassion we offer to others becomes something we learn to turn inward—this is the essence of self-compassion.

In this space, we learn to ask for what we need and offer support when we're able. We come to trust ourselves again—our voice, our timing, our needs—and to recognize the inner and outer resources available to us. Healing unfolds as we root ourselves in relationships that reflect back our wholeness and help us reconnect to our mind, body, and spirit.

This is where transformation begins: in a community of like-intentioned others, where we rediscover our life force and remember that we were never meant to do this alone.

Your journey: While it's natural to seek approval and validation from others, this journey invites you to return to your own inner compass. You are in the driver's seat now—guided not by external expectations, but by the quiet wisdom within you.

We strengthen this inner voice through intentional practices, which serve as steady guideposts along the way. As you move through the program, we encourage you to take what resonates, build on it, and let go of what doesn't—trusting that anything you release will return if and when it's needed.

This is your journey, in your timing. We're simply here to walk alongside you.

On this journey, there's no one-size-fits-all solution. We need a big and diverse toolbox to navigate the path ahead.

The Roots of Roots

The initial seed of *Roots to Thrive* was planted in 2018, when a group of healthcare providers with lived experience of burnout came together to respond to the growing mental health crisis on Vancouver Island. The initiative was led by Dr. Shannon Dames, a mother of two and nursing professor with a background in public health and a doctorate in education specializing in resilience. Shannon initiated the project not only to support others navigating burnout, but also from a deeply personal place—a desire to explore whether meaningful, lasting healing was truly possible for herself, as much as for others.

What began as a grassroots response has since evolved into a deeply collaborative program, shaped by the lived wisdom of heart-led professionals, Indigenous knowledge keepers, students, and patient partners. Together, they've co-created a model grounded in cultural humility, relational connection, and collective care.

This work has been made possible through generous support from the Michael Smith Foundation for Health Research, Island Health Authority, Vancouver Island University, philanthropic partners, and a range of government-supported grants.

The resilience-informed curriculum draws on a wide range of teachings, including psychiatry, psychology, somatic and energy-based modalities, functional and lifestyle medicine, and the lived experiences of hundreds of patient partners who have walked this path with us over the years. Guided by ongoing research and shaped by the wisdom of health experts, Elders, and Indigenous Knowledge Holders, the program's core concepts and practices are continuously evolving.

While the community of practice remains the primary medicine, additional supports have been introduced for individuals working through more rigid or deeply embedded patterns. In 2020, ketamine was integrated as an optional therapeutic tool, followed by the careful inclusion of psilocybin in 2021 and MDMA in 2024.

The theoretical foundation of Roots to Thrive draws on over five decades of Western-informed research, highlighting congruence (Rogers, 1959) and sense of coherence (Antonovsky, 1979) as core factors.

Congruence

Congruence represents our connection to our authentic self, as opposed to the 'idealized' self-prescribed to us by others. Our authentic self is the essence of who we are, the unchangeable elements that we were born into the world with, such as our unique desires, passions and talents. The idealized self refers to what we believe others want us to be; the masks we put on because we believe we need them to be accepted. It boils down to which self we show up to the world as - the genuine self or the socially prescribed self. The greater the disparity between these two selves, the more incongruent we feel, and as illustrated in Figure 1, the more shame we carry because of compromising our integrity (a violation of Self).

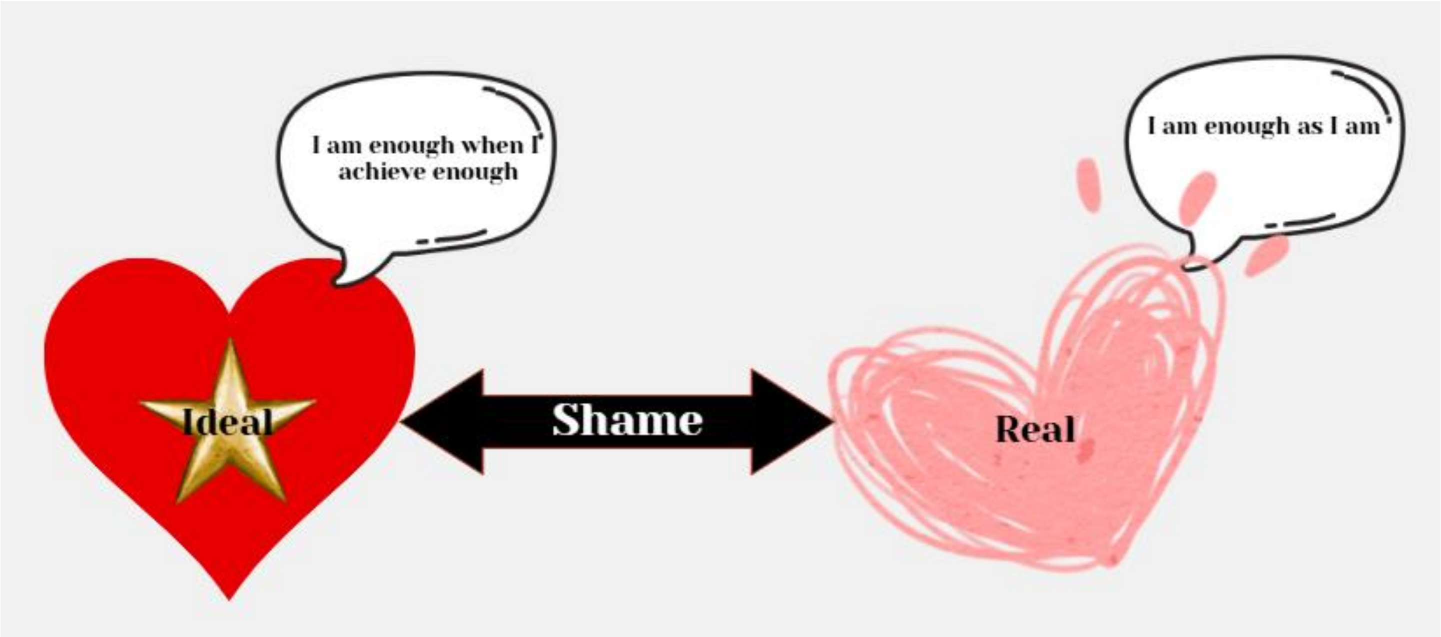

Figure 1. Congruence = Orientation to self, based on one's alignment between the 'real' and 'ideal' self. The wider the space between, the more shame we carry.

Calling in Reconciliation (explored further in Part 2):
Whenever we compromise our integrity, it's like a fracture within ourselves. Just as we would seek to repair a rift in a relationship with a friend, we also need to tend to the part of ourselves we've turned away from. When we suppress an aspect of who we are to fit in or avoid discomfort, we create distance from our authentic self. Reconciliation means gently returning to those parts with understanding and care—offering the same compassion we would extend to someone we love.

Essentially, our level of congruence reflects how connected we are to our real self. We act in congruence when we say "yes" because it feels true—not just because we think we *should*. When we agree to something out of obligation or fear of disappointing others, we place their needs or approval above our own. In doing so, we may quietly betray a part of ourselves. Over time, these small betrayals can pull us out of alignment with who we really are, weakening the relationship we have with ourselves.

In many cultures, the pressure to conform creates a sense of sameness, which can help us feel like we belong. This is partly because our brains are wired for connection. Mirror neurons—special brain cells that activate both when we do something and when we observe someone else doing it—help us learn through others and feel what others feel (Bonini et al., 2022). While this ability supports empathy and bonding, it also means we're highly influenced by the behaviors and expectations we see around us, especially when we're young.

Over time, we may begin to *mirror* those expectations, shaping our behavior to match what others approve of—even when it doesn't reflect who we really are. This helps us understand why congruence, or living from our authentic self, can be so difficult and yet so vital to healing. Expressing our uniqueness, like our passions and talents, can feel risky if they don't fit the cultural norms around us. Deviating from these expectations can invite social shaming, which we instinctively want to avoid. Because acceptance is a basic human need (Maslow, 1943), we often choose belonging over authenticity. Imagine a child who proudly calls herself an artist, and early on receives smiles and encouragement. But as she grows, the praise fades. She starts hearing more questions about practical careers and less support for her art. Slowly, she learns that being accepted may require her to push away a core part of herself. Even though creating still brings her joy, the fear of

rejection grows stronger. The *outcome* of her doing—how others respond—becomes more important than the *being* that inspires her creativity.

There are cultures who operate differently, celebrating the diverse qualities of their members. Sobonfu Some, an activist and spiritual teacher from Burkina Faso describes a different cultural experience:

> *I am from the Dagara tribe, and in my tradition it is customary for pregnant women to go through a hearing ritual. The purpose of a hearing ritual is to listen to the incoming baby; to find out who it is; why it's coming at this time; what it's purpose is; what it likes or dislikes; and what the living can do to prepare space for this person. The child's name is then given based on that information. Four weeks after the birth the naming for a baby girl takes place, and three weeks after the birth, a baby boy is named. In the Dagara tradition, you own your name up until the age of five. After the age of five, your name owns you. Your name is an energy; your name has a life force. It creates an umbrella under which you live. That is why it is important to hear the child before they giving him or her the name, because the name must match the purpose. My name, Sobonfu, means "keeper of rituals." (Some, 2010, para 2)*

Over time, the more we hide or reshape parts of ourselves to gain approval, the more distant we can feel from our authentic self. But incongruence doesn't only come from how we behave—it also shows up in how we relate to our emotions. When we push away uncomfortable feelings, we reject the part of ourselves that's trying to speak through those emotions. Instead of turning toward what feels threatening inside us, we often look—sometimes without realizing it—for ways to numb or distract ourselves.

This avoidance can take many forms: staying overly busy, scrolling on screens, reaching for food or substances, taking on everyone else's problems, or blaming others to avoid our own discomfort. These coping strategies are understandable—our nervous system is trying to protect us. But when we don't allow space for the full range of our emotions, we reinforce the message that some parts of us are unacceptable. Over time, this deepens the disconnection from who we truly are.

We'll explore this emotional avoidance and its impact more fully in the next chapter, as we begin to unpack how to turn toward our feelings instead of away from them.

In Robert Bly's *Long Bag*, he describes how incongruence develops:

> *When we were one or two years old, we had what we might visualize as a 360-degree personality. Energy radiated out from all parts of our body and all parts of our psyche. A child running is a living globe of energy. We had a ball of energy, all right; but one day we noticed that our parents didn't like certain parts of that ball. They said things like: "Can't you be still?" Or "It isn't nice to try and kill your brother." Behind us we have an invisible bag, and the part of us our parents don't like, we, to keep our parents' love, put in the bag. By the time we go to school our bag is quite large. Then our teachers have their say: "Good children don't get angry over such little things." So we take our anger and put it in the bag." … Our bags were already a mile long.*
> *Then we do a lot of bag-stuffing in high school. This time it's no longer the evil grownups that pressure us, but people our own age…Different cultures fill the bag with different contents.*
> *We spend our life until we're twenty deciding what parts of our self to put into the bag, and we spend the rest of our lives trying to get them out again. (Bly, 1989)*

Take a moment and look into your "long bag." What parts of your authentic self do you see in there?

Sense of Coherence

Throughout life, our sense of coherence fluctuates based on our ability to feel meaning and purpose in our lives, and a sense of confidence that we have what we need to face challenges along the way.

Sense of Coherence, a concept introduced by Antonovsky in 1979, refers to our overarching worldview—how we make sense of life's events and the confidence we feel in our ability to manage them. In his research, including studies of Holocaust survivors, Antonovsky identified a striking pattern: even among individuals exposed to similar extreme circumstances, some managed to thrive while others experienced profound dis-ease. What made the difference were three foundational elements (see Figure 2):

- **Meaning-making**: The sense that our actions matter, fueling motivation and purpose. Captured in the phrase: *"What I am doing matters."*
- **Comprehensibility**: The extent to which we perceive life as coherent and understandable—where events seem orderly and predictable. Summarized as: *"Life makes sense."*
- **Manageability**: A belief in our own capacity to meet life's demands, supported by the tools and resources we have. Expressed as: *"I have what it takes to make it through this."*

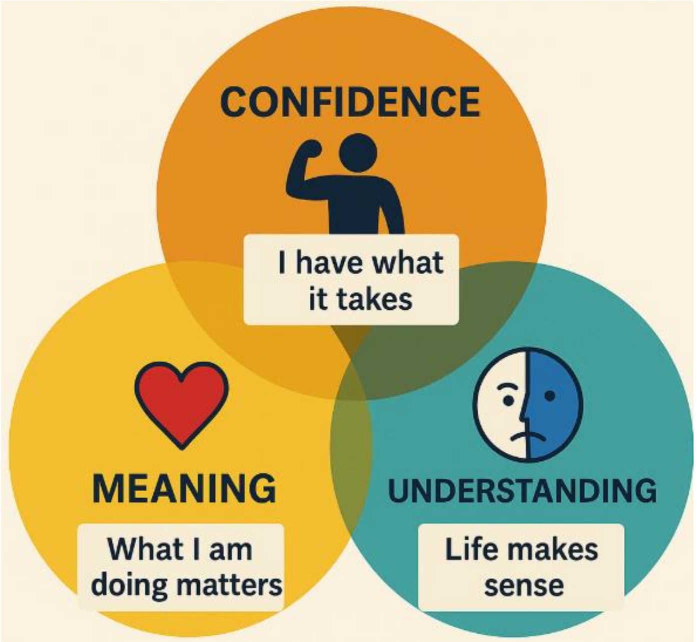

Figure 2. Sense of Coherence = our orientation to world, based on one's sense of capability, meaning, and understanding in the world.

These qualities—optimism, purpose, and confidence—give us the strength to take conscious, intentional action in the face of challenge, rather than falling into automatic or reactive responses. When we have a strong sense of coherence, our connection to meaning and purpose outweighs fear, allowing us to move through difficulties with both courage and compassion. We may even begin to view unexpected events as opportunities for growth, while those with a weaker sense of coherence are more likely to perceive such events as threats—activating the stress response and narrowing their options.

When we feel disconnected from the resources within and around us—when we lack trust in our own capacity and in the support available—our nervous system often shifts into survival mode. In this state, impulsive reactions take over, and we may start to believe the false story that we are not enough. Healing this

misunderstanding begins with reconnecting—to our inner wisdom, to the relationships that ground us, and to the greater abundance that reminds us of our worth and resilience.

Neuroscience supports this idea. A part of the brain called the anterior cingulate cortex (ACC) plays a key role in building a strong sense of coherence—the feeling that life is manageable, meaningful, and makes sense. What's unique about the ACC is that it doesn't activate automatically. It switches on when we choose to do something challenging—something we may not feel like doing, but we do it anyway because it aligns with what matters to us (Touroutoglou et al., 2020).

This type of effortful engagement, especially when rooted in personal meaning, helps us develop the inner grit we need to move forward with intention. It strengthens our belief that we can handle life's difficulties and find value in them. In this way, the ACC supports that quiet but powerful feeling of *"I've got this"*—the confidence that emerges when we stay committed to what matters, even when it's hard.

How Sense of Coherence and Congruence Inform our Ability to Thrive

Our congruence and sense of coherence shape our outlook on life, much like the tree shown in Figure 3. They determine the strength of our roots, which, in turn, influences whether we see events as threatening, manageable, or even beneficial. Just as a tree with deep roots is less troubled by passing windstorms (and may even find them refreshing), shallow roots can make us vulnerable to such winds, potentially posing a threat to our well-being.

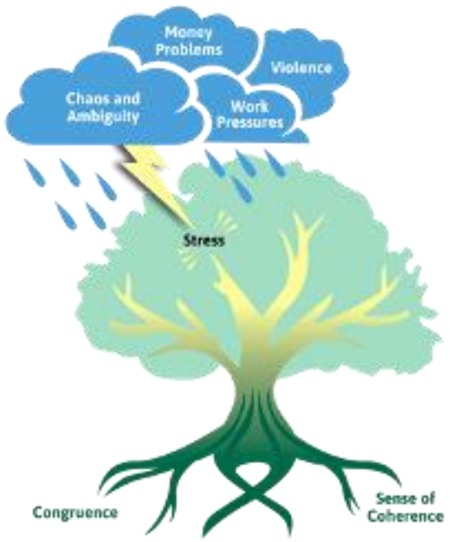

Figure 3. Congruence = Orientation to self, based on one's alignment between the 'real' and 'ideal' self. Sense of Coherence (SOC) = Orientation to world, based on one's sense of capability, meaning, and understanding in the world.

Sense of coherence is about feeling in control and finding meaning, making significant changes like starting a new job or becoming a parent an opportunity to learn and grow. We face challenges with confidence because we know we can navigate them. We look for the meaning in challenges because we know there is a valuable teaching within the experience. Conversely, when we lack power and purpose, the same events may feel like threats. Without recognizing our inner and outer strengths, challenges can overwhelm us, seeming impossible to overcome.

Congruence helps us avoid getting caught up in fleeting emotions and events. Strong emotions don't frighten us; instead, they give us insight into what truly matters. Feedback from others is less intimidating because our self-worth isn't tied to the opinions of others. Even when we feel threatened, we can step back from reactive impulses and approach it with curiosity.

When we don't resolve *perceived* threats, they become chronic stressors (elaborated on further in Part 2). Many of us navigate high-stimulation and unpredictable environments daily, which can feel threatening at times. If the sense of threat remains, the unease and uncertainty will linger, leading to constant vigilance and automatic reactions. This overwhelm hampers our ability to make thoughtful choices, pushing us into reactive and often habitual patterns that are rooted in learned patterns and past wounds. Many behaviors that lead to shame stem from this state of diminished choice. However, when we recognize the limitation of our choices, it allows us to view the bodies reactive behaviours with compassion. This concept will be delved into more deeply in Part 2.

How We Build Resilience: Brain Pathways for Congruence and Coherence

To truly thrive, we need more than just good intentions—we need environments where we feel safe enough to show up as our real selves. When we trust that we'll be met with acceptance instead of judgment, meaningful change becomes possible.

Figure 4 below shows how supportive relationships—like those in Communities of Practice—can activate two key parts of the brain: mirror neurons, which help us connect and feel with others, and the anterior cingulate cortex (ACC), which supports emotional regulation and intentional decision-making. Together, these experiences help us feel more aligned with our true self (congruence) and strengthen our sense of meaning, agency, and connection (sense of coherence).

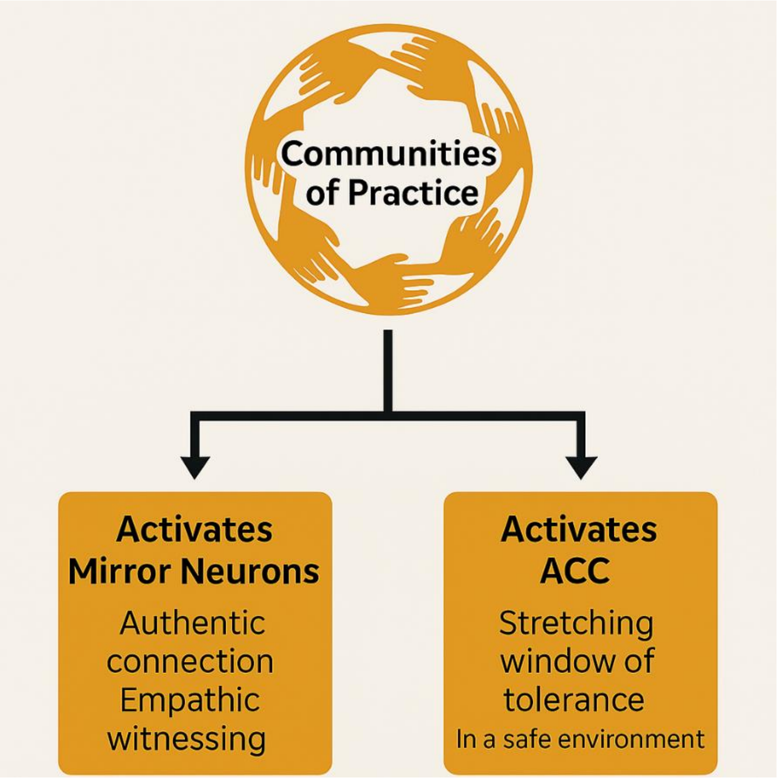

Figure 4. Communities of Practice create safe environments where people can experience empathic witnessing, authentic connection, and trust building. These experiences activate two key brain systems: mirror neurons, which support empathy and emotional resonance, and the anterior cingulate cortex (ACC), which helps us stay present with discomfort and make intentional, values-based choices. Together, these pathways support the development of congruence (authenticity and alignment with self) and sense of coherence (confidence, meaning, and clarity in life).

The Theory of Unmet Needs

While the Roots to Thrive theory is anchored in sense of coherence and congruence, it also draws from Maslow's Theory of Unmet Needs (1943) and co-regulation theories. As illustrated in Figure 5, Maslow's theory describes how unmet needs activate the stress response, disrupting our ability to engage in thriving. Once our basic physical needs are met, we become able to shift our focus to emotional safety, belonging, and esteem. However, if the need remains unmet (our bodies are cold, hungry, feeling unsafe, etc.), we will continue to make physical survival the priority, preventing us from addressing needs that feel less pressing. Conversely, if our physical needs are met, we become able to shift our focus to other needs, such as tending to our emotions. Becoming able to express ourselves authentically, marks a shift from surviving to thriving.

As each need is met, we progress toward **self-actualization,** where we can authentically engage with the world. While self-actualization involves becoming the best version of oneself and fulfilling one's potential, **transcendence** takes it a step further. It's about moving beyond the individual self, experiencing a greater purpose and connection to something larger than our self. In this state, we develop a deeper desire to be of service to others, recognizing that our fulfillment (and the strength of our 'roots') is intertwined with the well-being of the broader community.

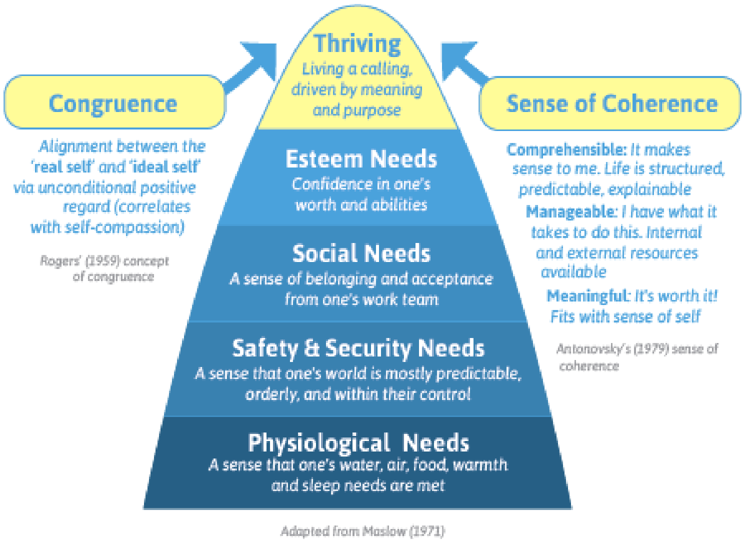

Figure 5: The Theory of Unmet Needs weaves together with congruence and sense of coherence. All three concepts suggest that basic human requirements must be met to engage in thriving.

Intertwining Root Systems: Nutrition for our Nervous Systems

The RTT theoretical framework, highlighting co-regulation theory (Figure 6), illustrates how connecting with a secure "other" enhances our personal sense of security. Imagine it as an inner vibration or frequency we carry within us. When we feel insecure, that inner frequency resonates with anxiety. However, by attuning to the

secure vibration of another, we can harmonize and mirror that same sense of safety within ourselves. This secure "tune" is like a radio signal we can retune to, grounding us in stability.

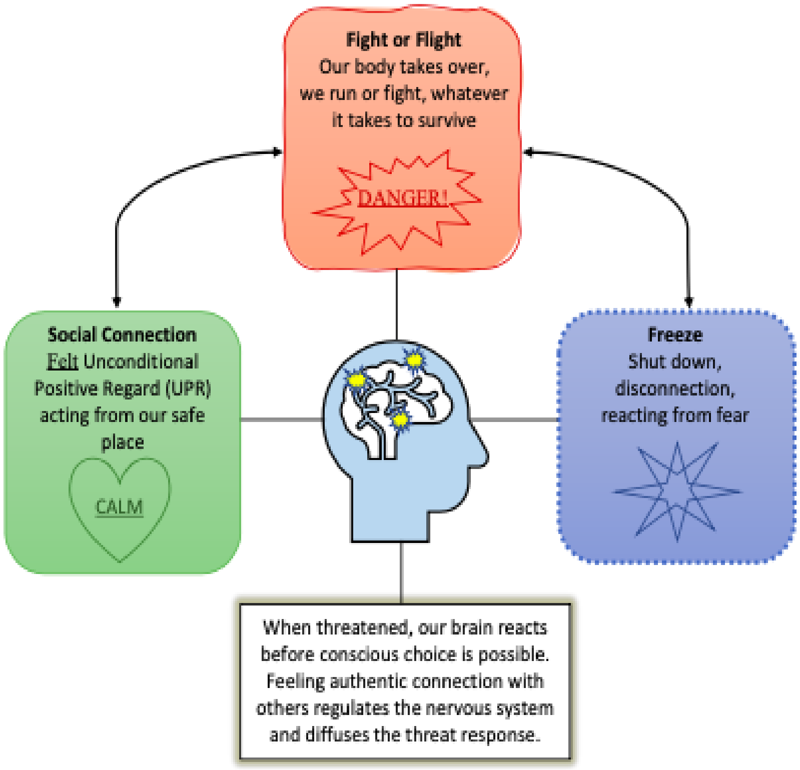

Figure 6: Regulating through Connection with Others. Simply BEing in secure relationships can diffuse the stress response, enabling us to navigate challenges from the mind-body-spirit interface.

Through our years of facilitating intentional communities of practice, we have discovered that being part of a community that prioritizes unconditional positive regard and collective security naturally helps regulate our nervous systems. This creates new pathways that foster a general sense of security in the world—a familiar tune we can reconnect with when we feel lost. This self-regulation calms our bodies, allowing us to engage with trauma without becoming overwhelmed or overly identified with it. When our nervous systems are balanced, we are more equipped to tackle challenges creatively, confident that we have the inner resources to address the unmet needs at the core of those challenges.

As we experience a growing sense of belonging and connection to others, self-consciousness fades, enabling us to show up authentically rather than conforming to external expectations. Authentic relationships serve as mirrors of unconditional positive regard, and over time, we learn to reflect that same compassion inward. This self-compassion helps soothe our bodies in moments of stress and serves as a guiding light through life's darker moments. By healing and reintegrating previously disowned parts of ourselves, we come into wholeness, becoming vessels of service to others—emitting a secure vibration that others can attune to.

A Note on Service: True inspiration to serve springs from the heart, not the intellect. When gratitude for what we have surpasses our fear of scarcity, service becomes a natural extension of our inner abundance. Like an overflowing cup, service pours out effortlessly when we feel full within.

Creating a safe community is much like baking a cake. With the right ingredients and a consistent process, the outcomes are reliable. This time-tested "recipe" includes:

- Shared Intentions: These serve as the community's collective "North Star," guiding everyone toward a common goal. One person's intention is powerful, but when an entire community holds shared intentions, the effect becomes exponentially stronger.
- Agreements: These form the framework that holds the collective together, ensuring resilience during challenges. Agreements act as guideposts, steering the community toward shared goals while promoting safety, cohesion, and collective momentum.
- Transparency and Predictability: Open communication and clear expectations foster trust, a crucial element in gaining agreement and buy-in from each member. Transparency and predictability reduce anxiety and instill confidence, contributing to a sense of coherence—an essential factor in resilience, both individually and as a community.
- Alignment: When members align with shared intentions and agreements, it fosters harmony. Misalignment, like an out-of-tune instrument, creates discord and can lead to dis-ease within the larger community.

Community is the ultimate nourishment for our nervous systems — Todd Haspect

Breaking Free of Patterns

By Crosbie Watler

Bringing awareness to patterns that create suffering

Much like inclement weather, challenges come and go, often unexpected and often outside our control. Challenges can activate reactive patterns of judgment and resistance, where our true nature is eclipsed by the mind field. The mind's sole purpose is to think, to turn. It is a good servant, but a poor master. We are called to bring discernment to our thought patterns, reflecting on the acronym WAIT–Why Am I thinking? Is it serving any purpose, or simply crowding space?

Our true nature is simply awareness without thought. True nature resides in the space between our thoughts. Thoughts come and go, but I am not the thinker, I am the witness of thought. Exploding the myth of "I think therefore I am", to simply: I am, no condition. No one and nothing out there is required to complete me. When we look externally for our worth, the doings are never enough, and we are never enough. Our thoughts and emotions often create an escalating body-mind activation, keeping us stuck in self-defeating patterns. Our thoughts and emotions are literal gas guzzlers, consuming all our attention, and eclipsing the still awareness of true nature, the being self. Metaphorically, the clouds have obscured the sun. We then believe that we are the clouds, the veneer, not the essence of the thing. Yet, the sun has never gone away, always present, patiently waiting for a crack in the facade to reveal herself. Even on the darkest day.

We are called to notice when the mind is driving the bus, and the distress that usually ensues: Am I using my mind, or is my mind using me? Our bodies will tell us– constriction, tension, heaviness. No need to name the emotion, or the story connected to it. No need to push it away. Rather, we cultivate a practice of noticing when we lose it to the stickiness of the mind field, and return to true nature.

We connect with our true nature, being self, not by resisting thought, but by stepping out of thought. Here we can trust the truth that awareness can only be in one place at a time. Anchoring 100% of our attention to the flow of breath in and down, and up and out, bringing awareness to the flow of breath and the space it creates in our bodies. Bringing our awareness from above the neck, to below the neck, using breath as our trustworthy ally.

To ward off insecurity and driven by a need to know, the mind/ego will put everything into little boxes, manufacturing "truths" that ain't necessarily so. Here are some familiar themes:

"I am not enough, I am a failure".

That narrative lands hard and we've all been there, needing something out there to complete us. A success, a validation, a relationship, seeking to arrive at some future moment that's better than the present moment. When we succeed at any given enterprise, our happiness is transient and conditional. In truth, we have outsourced our well being to the weather of our lives.

I shame or blame myself, or to ward off awareness of any chinks in my own facade, I project anger or judgment towards another…again, who is angry with who? My insecure toddler is in conflict with your insecure toddler and if we relate from there, we both lose.

What if we bring curiosity to our stuck thought patterns: Who is not enough, who is the *I* that's a failure?

Our doing selves will never be enough. The doing self is a perpetual toddler, doing the best he or she can with our awareness and tools at any point in time. We do not shame a toddler for her missteps. We understand that the toddler is doing the best she can with her awareness and tools. The toddler can simply do no better, it's not a choice and there is no shame in it.

When the mind addiction takes us up and out, we are called to notice. How grounded am I? Our bodies will tell us when we've lost it, as we will. The goal is not to never lose it, but to return to still awareness, shifting our attention in and down, from up and out, even though the latter is far more seductive (Elder Duncan Grady). Breath is one tool that can tether us to true nature and the wisdom that resides in the space between our thoughts.

The deepest thinkers think very little, they cultivate still awareness, bringing wisdom and discernment to the task at hand. My highest purpose is no longer what I ***do***, it is noticing when my mind is stuck in the habitual field that creates suffering, redirecting to the creative field of still awareness.

In that space there is no judgment of self or others, no scarcity of attachment. Only gratitude and compassion. We don't choose our patterns, and we all have patterns that don't serve. Our doing/toddler self is doing the best he or she can. Others too. True nature can leave the light on, holding space for compassion and unconditional positive regard.

Despite my lying eyes telling me I'm all grown up, I've surrendered to the fact that my doing self is a perpetual toddler, and that it's not a matter of if, but a matter of when, I'll fall on my ass. At those times, I am called to notice, to align with true nature, bringing awareness to missteps and patterns that do not serve.

With time, we come to know, come to trust the healing and wisdom that greets us in the space between our thoughts. It is, after all, our birthright, the clouds just got in the way.

Slow and Steady with an Inside Approach

This program honors all forms of reconciliation, beginning with the relationship we build with the messengers inside our bodies. Emotions and sensations are not problems to fix, but *benevolent guides*—offering insight into areas of imbalance, unmet needs, or perceived threats. While cultural reconciliation helps bridge divisions between communities, true healing often begins within—by restoring harmony between our mind, body, and spirit. As we tend to these core parts of ourselves, we open the door to deeper, more meaningful relationships with others and the world around us.

Let your body be your guide. Being true to yourself can stir up fear—especially the fear of being rejected or misunderstood. When we don't feel safe in our relationships, we might shut down emotionally. Vulnerability can feel overwhelming, leading us to retreat into overthinking instead of staying with the discomfort. That's why we begin with trauma-informed practices. Many of us carry the belief that the world isn't safe, and that belief shapes how we show up. When safety is uncertain, our nervous system shifts into protection mode, and our ability to handle emotional stress becomes limited. But when we feel safe, we soften. We can show up as our whole selves—without the need for masks or performance.

Healing happens at the *speed of trust*. Sometimes we'll stretch outside our comfort zones—and sometimes we'll need to pull back. That rhythm is part of the process. These moments teach us where our boundaries lie,

how to be gentle with ourselves, and how to build resilience through self-awareness. As we learn to navigate our edges, we gain the courage to ask for help, receive support, and move forward with greater strength.

PART 2: DEVELOPING ROOTS TO THRIVE: A RECONCILIATION PROCESS

In the upcoming sections, we explore opportunities and practices that help us grow sturdy roots, and guide us in rediscovering and reconciling with our whole selves. To do this, we focus on four core practices that foster the growth of congruence and sense of coherence.

Each of the core practices (Figure 7 below), are woven throughout the text, transitioning us from theory to practice. Please find a link attached to each, providing a video that explains the basics of each practice:

- Awareness of our authentic selves, and the abundance of resources within and around us.
- Regulating and soothing our nervous systems. This includes both self-regulation and co-regulation—aligning with the rhythms of a supportive community, syncing with our emotional landscape, and engaging in meaningful action that expands our sense of agency and possibility.
- Heartfulness/Compassion - Offering unconditional positive regard to others and ourselves. This outward mirroring and inward self-compassion builds a foundation of love and acceptance that strengthens our relationships and our sense of belonging.
- Alignment/Living a calling. Discovering and trusting our inner compass, and embracing the integrity needed to live a meaningful, purpose-driven life.

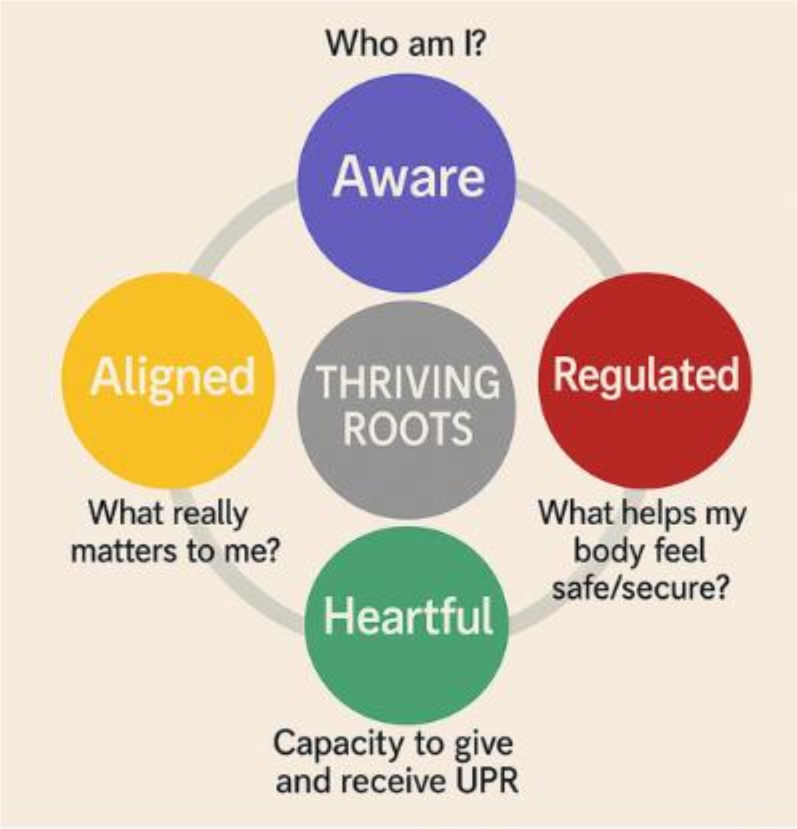

Figure 7: By integrating these four core practices into our daily lives, we strengthen our connection to who we truly are and learn to trust in the abundance that surrounds us. While separation from self, others, and Spirit is a common consequence of colonized cultural systems, this curriculum offers a pathway back—a process of reconciliation with ourselves, with one another, and with all that is.

Meaning Making: Reconciling with the Mind

Understanding the Roots of Stress

Imagine an ocean with towering waves. When equipped with a surfboard and the skills to navigate these waves, the experience can be exciting. In contrast, lacking the knowledge to handle the surfboard or expecting to be overwhelmed by the waves results in stress. The situation may be the same, but our frame of mind makes all the difference.

To thrive amid life's big waves, two essential conditions must be met:

- Sense of coherence: Trust in the external world (physical and relational surroundings) to fulfill our basic human survival needs, and a general sense of confidence that everything will work out okay. From this place, we can make meaning amid life's challenges, and welcome the lessons learned along the way. Confidence in our internal and external resources improves our capacity to respond thoughtfully - providing an opportunity to choose a course of action from a more objective and secure place. This confidence, even amidst challenges, helps us avoid succumbing to unconscious (choiceless) reactions.
- Congruence: The security, and thereby confidence to engage with and express our emotions, trusting we will be met with unconditional positive regard as we do. This empowers us to heed our emotional messengers, recognizing them as benevolent allies that are leading us back home to wholeness.

Stress emerges when our nervous system detects a potential threat to our essential needs. Once we perceive a threat (whether or not it's an actual threat), our brain releases chemicals that signal our nervous system (as illustrated in Figure 8 below). Subsequently, our nervous system activates a full body response to protect us. This entire process happens in an instant, leaving us no time to assess the accuracy of our perceived threat. From the perspective of our brain, it's more effective to react immediately to any potential danger rather than delaying for evaluation – just as we instinctively flinch when we touch something hot. This rapid response to perceived threats defines what we call a **stress response.**

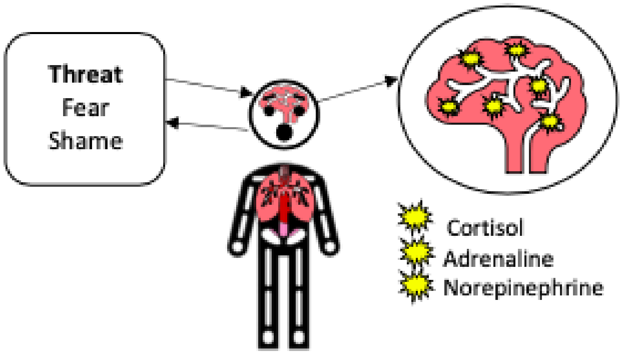

Figure 8: Neurochemicals (i.e. cortisol & adrenaline) are released when we believe we are threatened, which activates the biological stress response. The stress response mediates how we react to the event. When this happens, conscious choice is at least hampered, if not impossible

The stress response serves a protective function, urging us to be attentive and vigilant. Increased awareness enables us to focus on the potential threat and find a way to resolve it. We tend to respond by:

- Fighting – exhibiting anxiety or hostility
- Fleeing – manifesting as depression, distraction, or numbing
- Freezing – involving disconnection and dissociation
- Fawning – prioritizing others' approval over our own well-being.

The primary role of the nervous system is to react to threats by getting the body ready to react. When the stress response is activated, survival becomes top priority, often leading to the suppression of emotions that might distract us. Imagine driving home from a funeral and suddenly a car runs a stop sign, forcing you to slam on the brakes to avoid a collision. The sadness you were experiencing instantly disappears – because sadness isn't useful at that moment. What matters is quick reflexes and heightened alertness for hitting the brakes and swerving if necessary. Your nervous system takes over, ensuring control. So, there are instances where the stress response is vital, even life-saving.

However, this same stress response can happen when an emotion *feels* threatening. Your body gears up to confront the perceived threat, which happens to be your own emotional state at the time, and the emotion gets pushed aside. Until the danger subsides, our body's defense mechanisms – reactive behaviors driven by fear, aiming to protect us – remain in charge. In this reactive state, we are prone to act in ways that are incongruent with our values, leading to shame. It can be a difficult cycle, where the shame from reactions lead to further reactions, leading to further shame. Eventually, this shame fills us to the point that it spills onto others. This entire cycle is unconscious, and yet, we often hold ourselves and others accountable for choices that were never choices in the first place.

As mentioned earlier, whether an event is seen as a threat depends on how similar events were interpreted by our brains in the past. For example, if you were chased and bitten by a dog as a child, you may have developed a fear of dogs. If so, this fear would arise whenever you see a dog, regardless of its behavior, whether friendly or aggressive. Your brain would see a dog and immediately sense a threat, without hesitation.

When the perceived threat is intense or imminent, there's little room for choice or willpower. The nervous system acts protectively and preemptively, often immobilizing the body before we can consciously engage. Thus, our behaviors in such situations reflect the state of our nervous system, rather than our desire for change. Blaming ourselves or others for lacking willpower only fosters shame, making us feel disempowered and limiting our choices.

The fear-driven stress response can cloud our ability to make objective decisions until we learn to distinguish between an actual threat and one that's perceived or linked to past experiences. This involves recognizing the difference between the emerging threat we feel and the echoes of unresolved past wounds (aka trauma). To achieve this clarity, it requires is to step back (non-attachment is elaborated on later), so we can assess whether the stressor is an actual threat. If real, we can take the necessary steps to address it, calling on external resources as we need to. If rooted in the past, we have the chance to heal an old wound, working with relationships and resources available in the present. When viewed in this way, it can help us reframe a stressful moment into an opportunity to heal an old wound that we weren’t resourced to heal when it first occurred.

Pause to Reflect: What is stress to you?

What are some things that activate your stress response?

How does stress feel in your body?

Pause to Practice: Interrupting the Stress Response

Throughout the program, we'll explore and practice many methods to reduce stress and calm the nervous system. It's important to note that there are lots of stress-reduction tools out there, and we are sharing a sliver of them. The ones we chose are supported by research, frequently used by team members, and have been well received by past participants.

While there is no obligation to adopt them, we hope you will add them to your toolbox so you can call on them when you feel inspired to do so. Remember, it's not about how many tools you possess, but rather how inspired (motivated from within, not based on guilt and obligation) you feel to use the ones you have. Keep experimenting until you discover something that brings you fulfillment. If one technique becomes cumbersome, feel free to explore other options that pique your curiosity. Trust your instincts – if something doesn't resonate with you, there's no need to push it.

Start by taking a moment to reflect: What are your current go-to methods when you're feeling stressed? Avoid categorizing or judging them as 'good' or 'bad' behaviors; just honestly list them.

Managing Stress: The Role of Co-Regulation and Somatic Reminders

To heal from past trauma, it's important to establish a sense of security that allows us to confront and express previously overwhelming emotions. Building this security hinges on developing trusting relationships - characterized by unconditional positive regard. When we do so, a protective space forms between perceived threats and automatic reactions. This newfound security, akin to the deep roots of a tree (Figure 3), shields us from feeling threatened by passing emotions. Instead, emotions become informative rather than threatening, enabling us to reinterpret traumatic events through a more empowered lens. This process underpins the rewriting of old narratives and the release of emotions that are stuck to old wounds.

Co-regulation centres on the transformative power of authentic relationships that mirror unconditional positive regard, providing a sense of safety for our vulnerability. These relationships expand our ability to allow intense emotions that were once too overwhelming to sit with. As our emotional capacity expands, our **inner healing intelligence** kicks in, and suppressed emotions from the past gradually begin to surface in our daily lives. This is a promising sign, although it might not always feel that way. Our body seizes the first opportunity to mend wounds, as it's naturally inclined to do.

Furthermore, as part of our journey, we incorporate somatic practices aimed at listening to the body, soothing the fears that present, and as a result, calming the nervous system. These practices serve as reassuring reminders to the aspects of ourselves that can be perceived as threats, assuring us of our safety and well-being.

The below are some common somatic practices that we work with to cultivate awareness, self-regulation, and compassion:

- R.A.I.N.(S): A mindfulness meditation method that was adapted from Tara Brach's work (2019), elaborated upon in Chapter 4.
- The 4-7-8 Breath: Also discussed in Chapter 4, this technique helps regulate breathing patterns.
- Harmonizing the Body's Energy Field: Utilizing various forms of comforting touch to work with the body's energy.
- The Butterfly Hug: A technique designed to provide comfort and calmness.
- Emotional Freedom Technique (EFT): Further detailed in Chapter 4, this method aids in emotional release and balance.

These practices complement the community of practice, promoting self and co-regulation, and ultimately, improving our resilience and well-being.

Incorporating R.A.I.N.(S)

Trauma isn't defined by how much we've been through—it's shaped by how safe we feel to be with what we're feeling. Healing begins when we have the space and support to recognize, allow, investigate, and nurture our emotional experiences, and the confidence to take a significant step forward when the time is right.

The R.A.I.N.S. technique, inspired by the work of Tara Brach (2019), is a foundational practice in the *Roots to Thrive* program. The first four steps—Recognize, Allow, Investigate, and Nurture—support us in reconnecting with ourselves and building greater congruence between what we feel and how we live.

At first, this inner work can feel a bit awkward—maybe even *cringy* or *cheesy*—especially if we're not used to paying attention to what's happening inside. It can feel like trying to reconnect with someone you've been distant from for a long time. But with time, patience, and self-compassion, that relationship with ourselves begins to soften. The sensations, emotions, and patterns we once avoided start to feel like familiar messengers, not threats.

We add one more step to this process: the **Significant Thing**. This represents a conscious choice—a meaningful action or intentional pause—that comes after we've met our inner experience with honesty and care. It's not about pushing ourselves or rushing to fix something. Instead, it's about *intentional forward momentum*—stretching ourselves just enough to align our actions with what really matters.

This final step builds **agency**. It's like climbing onto a surfboard in the face of a towering wave. It might feel uncertain or effortful, but the act of choosing—even in the smallest way—reminds us that we're not powerless. That we can ride the wave instead of being pulled under by it. The Significant Thing becomes an anchor—a reminder that we can meet life's challenges without abandoning ourselves.

To explore this technique further, visit Tara Brach's website, www.tarabrach.com, which offers free guided meditations guiding you through the four steps of R.A.I.N. and helping you start this practice. The concept of the 'significant thing' is elaborated on later in the guide.

Breakdown of R.A.I.N.S:

- **Recognize:** Begin by noticing what's happening within you. Gently bring awareness to your thoughts, emotions, and body sensations—without labeling them as good or bad. Simply observe what's there. This is you, providing a compassionate witness to yourself.
- **Allow:** Give yourself permission to feel what you're feeling. Let the experience be as it is, without trying to fix, change, or push it away. This is about making space for the truth of the moment.
- **Investigate:** With curiosity and care, explore your inner experience more deeply. What might this emotion be pointing to? What need, wound, or value is trying to speak through it?
- **Nurture:** Offer yourself the same compassion you would extend to someone you deeply care about. Imagine how you would comfort a friend in pain—and let that same warmth turn inward. Our brains are wired to connect this way; when we reflect loving attention back toward ourselves, it activates the same pathways that support empathy and connection with others. Remind yourself that what you're feeling is human—and that many others have felt this way too. You are not alone.
- **Significant Thing:** When you're ready, take a meaningful step—a small but intentional action inspired by your inner healing intelligence. This isn't about forcing change or reacting quickly. It's about choosing to act—or consciously choosing *not* to act—in a way that aligns with your values, even when it feels uncomfortable or effortful. Sometimes, the most powerful choice we can make is to simply stay with what is—to pause in the discomfort, trusting that clarity will come. These moments of intentional stretch rewire our nervous system, strengthening the pathways that support courage, clarity, and resilience. Over time, they help us build the confidence to move through life with greater presence and purpose.

Pause to Reflect: Applying R.A.I.N.(S)

As an example of applying R.A.I.N.S., picture being in a highly stimulating environment—kids playing energetically, phones ringing, call bells chiming—and feeling a loss of control. In such a scenario, you can move through the R.A.I.N.S steps. Recognize the factors activating your nervous system, allow the anxiety to stay a while, investigate the layers of the experience to comprehend how to navigate it, and nurture your body by acknowledging and reassuring it. The significant thing might involve removing yourself from the environment or using your voice to establish a boundary. This practice helps you take control and remain centered amidst chaos.

Think about a recent challenging situation you encountered. After moving through the first four steps of R.A.I.N.S, can you identify what significant action (or inaction) was being called for in that moment?

Pause to Practice: Cultivating Presence with Breath Awareness

by Crosbie Watler MD, FRCPC

Presence could be defined as bringing still awareness to whatever is arising, right here, right now. It is experiencing this moment, free of any judgment, or clutter from the mind field. There are two truths that are helpful for cultivating presence, building our capacity to use our attention with intention:

Attention is finite, metaphorically, we have $100 of attention and should reflect on whether we are investing our attention wisely. Are my thoughts serving any purpose, or simply cluttering space, creating suffering. The acronym WAIT? is a great reminder: Why am I thinking? Is it serving any purpose? At these times, we are called to step out of thought and into presence, awareness without thought.

Attention can only be in one place at a time. When we believe that we are multitasking, in truth, our attention shifts back and forth. What we attend to grows. Attention is like fertilizer. Like a discerning gardener, we are called to notice whether we're feeding the veggies, or the weeds. Without this discernment, the mind will run the show, perpetuating patterns that do not serve us, or those around us.

When we lose presence to the stickiness of the mind, our bodies can remind us that we've lost it. Presence has been usurped by thought addiction, a challenge we all share, but can manage, often by noticing what's arising in the body. What do you feel in your body when anxious, afraid, sad, angry? Whatever the distressing emotion, it lands as a felt sense in the body. This can serve as a reminder to step out of thought, coming to know, coming to trust the wisdom and intuition that lives in the space between our thoughts.

Our reliable and omnipresent ally is awareness of breath and the space it creates in the body. We shift our attention with intention, bringing our awareness from above the neck (mind field) to below the neck (the body). Following our breath in and down, pause, then up and out, with 100% of our attention on the flow of breath and the space it creates in the body. This practice builds on the two points above–if our attention is fully grounded in awareness of breath, there is simply no attention or bandwidth left for the turning mind.

Imagine a light switch on your sternum, your breastbone: is my attention above the neck, or below the neck? Think when it serves, notice (in the body) when it does not. When our thoughts create distress, rather than judge or resist thought, we can simply step out of thought, light switch down, shifting awareness to breath and inner space. What you experience in that still space between your thoughts is your true nature, free of the stickiness of ego and attachment. Pilot light, true nature, being self, consciousness, all attempts to describe something that cannot be named, but is felt. Whatever you experience there is your truth, your inner divinity.

> *Breath awareness anchors our awareness in the present moment, aligning us with what is, instead of what we wish it to be. It is a resource that is always available. Practice these two breathing techniques, noticing their effects on your body. To better understand how your body responds, notice how settled you feel, both before and after the practice.*
>
> *4-2-4 breath: Breathe in for 4, pause for 2, then out for 4. No need to count the seconds, just an approximation. 4-2-4 breath is a versatile ally, as we can practice it when in doing mode–conversations, activities. Before we speak, we need to inhale. Before rushing to speak, pause briefly to bring awareness to space in the body, then speak from there. This is aligned with the practice of yoga, connection with breath and inner intelligence (more on this later in the program) before doing anything.*
>
> *4-7-8 breath is another technique: in for 4, pause for 7, out for 8. Experiment with both, as they have a different feel and different applications. Consider 4-2-4 breath as the foundation for heartfelt conversations and 4-7-8 breath for settling activation when there is the luxury of nothing to do.*

We have thousands of waking breaths per day, and each is an opportunity to build a relationship with the wisdom and well-being that greets us in the space between our thoughts. Don't wait for a crisis, rather, maintain an ongoing curiosity: *How grounded am I?* Build the connection, the relationship with presence, notice when you lose it, as we will, and simply return to it.

Set an intention this week to try speaking from the pause between breaths. Listen from there as well. I've been told that it's not the notes in music that draws the listener in, it is the space between the notes.

Present Day Stress can be the Voice of Past Trauma: What are you Hearing?

Traumatic events are a hard and inevitable part of life. In Western culture, experiencing difficult things can bring up feelings of shame, as though some personal flaw or weakness is responsible for what has happened to us. This is reinforced by cultural beliefs such as *'good things come to those who wait'* or the idea that hard work is rewarded by success (so if you aren't successful, you just aren't working hard enough). The reality is, the current conditions in which many of us now live are inherently traumatizing. Trauma is a normal response to toxic cultures and colonizing systems.

"Trauma is not what happens to us, but what we hold inside in the absence of an empathetic [compassionate] witness." Peter Levigne

If we feel too unsafe to express our feelings around a traumatic event, these feelings will be pushed away, hidden from us in a sense. The emotions don't go away though, however much we may wish that they would. The feelings, and the energy attached to these feelings, get 'stuck' in our body. It remains a stuck energy that the body carries forward. The more stuck emotional energy we carry, the more likely we are to consider future events as threatening, whether or not they actually are.

In this way, trauma can be thought of as *stuck* emotional energy; the emotions are there, somewhere, but we haven't yet acknowledged them, felt them, or released them. Trauma is stored in our body, not in our mind. This means we can work to feel and release the emotions related to a traumatic event, without having to re-visit the event itself. Furthermore, our body knows intuitively how to heal itself. When we have a cut, we ensure it's clean and protected, and our body does the rest, providing it is in a relatively healthy state. The term **inner healing intelligence** is the idea that our body similarly knows how to heal our inner wounds.

Whenever something reminds our nervous system of a past traumatic event, our stress response kicks in. The emotions we couldn't fully process back then often resurface. This can create confusion between the emotions from the past and the present situation. This is called an **emotional projection** – emotions from a previous event projecting onto something happening now.

It's important to remember that unresolved emotions from the past can feel threatening when they come up in the present. That's because these emotions initially formed in a threatening environment. In real life, this could mean overreacting to a comment or situation. Instead of feeling ashamed of such reactions, try being curious about what other emotions might be behind them.

We can only recognize, feel, and express past emotional pain when we feel safe and confident enough to work with it. Otherwise, we'll keep pushing these emotions away every time they come up, usually subconsciously. These stuck emotions will keep resurfacing until we're ready and able to feel and express them. These

emotional projections provide a way for our inner healing intelligence to help us feel and heal unresolved trauma. Here's an example of how an emotional projection might play out:

> *A well meaning co-worker is giving me constructive feedback about a task I completed. I can't see it as such - to me it immediately feels deeply threatening, and I can only see it as criticism. I become angry and defensive, and storm off. When I am able to get myself more calm, I recognize a feeling of unworthiness. I reflect on the fact that I grew up with an extremely critical parent; nothing I did was ever good enough. I see how - without even thinking, or having a chance to think - I responded defensively based on past hurts.*

Recognizing these projections helps us step back and understand where the intense emotions are coming from. It gives our body a chance to recognize that old hurt and maybe release some of the emotional intensity. In the example above, spending time exploring the feelings of inadequacy from childhood and validating them might help. We'll experience these projections until we feel secure enough to work through the intensity of the emotions that need to be felt.

Pause to Practice: Compassion for the Nervous System

Compassion for our bodies enables us to hold awareness from a place of abundance, enabling us to direct unconditional positive regard inwardly. As a result, we come to believe we are *good enough.*

Awareness without compassion often leads to perfectionism, fixing our eyes on what is wrong, fueling the shame that follows when we cannot reach a self-ascribed ideal. As a result, we come to believe we *not good enough.*

Understanding that our nervous system is here to protect us and always has our best interests at heart, we can start learning to see our stress responses with kindness, without judgment or self-blame. This skill gets better with practice. One way to do this is by talking to these responses like you would to a close and loyal friend – like a 'dear other.' This method helps us develop a caring inner voice.

Though it's important to use your own words for a sincere connection, here are a few examples of how you might have a 'dear other' conversation with your nervous system:

Talking to your nervous system:
"I really value how you protected me in the past."
"You were there for me when I couldn't protect myself. I'm safe now. I can handle things."
"I understand how much you've done to keep me safe. You've been a true friend."
"We're not on our own anymore. We're in a safe place now."

Addressing the part of yourself that still carries fear from past wounds:
"I recognize your pain. I feel it too."
"I'm so sorry for what you went through."
"I've got your back. I'm looking out for you."
"Let's face this together."

Imagine someone you deeply care about in your life – it could be a child, grandparent, partner, friend, or parent. Picture them feeling vulnerable, scared, and isolated. How would you show them kindness and support? Use this as a guide to show yourself the same compassion.

Continue to play with different phrases that resonate for you. Developing a self-compassionate inner voice is an important part of the process. In doing so, by compassionately witnessing what *is*, especially when it is different from what we *want* it to be, we are turning unconditional positive regard inward.

Pause to Practice: Clearing Stuck Energy through Expressive Writing

Expressive writing serves to metabolize (digest) our emotions without becoming overwhelmed by the process. Research indicates that this practice can alleviate symptoms of conditions like PTSD, anxiety, and depression (Smith et al., 2018). Furthermore, it also enhances brain function, promotes positive behaviors, reduces pain and fatigue, and enhances overall well-being (Tonarelli et al., 2018). By articulating our emotions in words, we forge a connection between the heart and the mind. Another perspective is that it acts as a conduit for the thoughts and emotions rattling around within us, transferring them onto paper. This process helps us distance ourselves from these thoughts and emotions, making them feel less threatening and, consequently, easier to process and integrate.

It's like talking out loud to stop repetitive thoughts – writing does a similar thing for thoughts causing emotional distress. To make writing fulfilling, find ways to enjoy it (what makes it fun?). If avoiding feelings is a challenge, writing might be tougher (Sabo Mordechay, Nir, & Eviatar, 2019). If it's frustrating, practice self-kindness. Adjust the process to create a safe and open space, increasing your ability to handle your feelings. Some might easily connect with their feelings, while others need more effort. The goal is to find what suits you and keeps you connected to your feelings.

Here's how it works:

1. Set Aside Time: Find a quiet space. Set a timer for 10 to 15 minutes.
2. Notice how you are feeling before you begin.
3. No Judgment: There's no right or wrong. Just express yourself honestly.
4. Choose a Topic: Start with a specific emotion or let your thoughts flow freely.
5. Write Freely without worrying about grammar or structure.
6. Stay Present: Focus on the present moment and your feelings.
7. Let It Flow: Write continuously. If stuck, keep the pen moving.
8. Stay Open: Explore deeper emotions and unexpected insights.
9. End When Time's Up: Write until you have nothing else to say, or until the timer goes off.
10. Burn it: As you watch the fire transform it, imagine you are transforming with it, releasing something old to birth something new. With the release of something old, breathe in something new. Breathing out the old, breathing in ________ (what are you wanting more of?).
11. Notice how you feel now.

Remember, the goal is to express and process your emotions, not create a polished piece of writing. You can use this practice regularly to explore feelings, gain insights, and connect with your thoughts. If helpful, you can even burn or shred the writing after, emphasizing that what's important is the process, not the outcome.

Managing "Weather" with Secure and Healthy "Roots"

Referring back to the tree analogy provided earlier (Figure 3), those more congruent, and who have a greater sense of coherence, have stronger, more enduring roots, giving them more stability even amid tumultuous conditions. An event acts like the changing weather that gets our attention. It might stem from our thoughts, emotions, bodily sensations, relationships with others, or interactions with the world. For example, consider the thought of an upcoming important exam. Although the exam isn't happening at that instant, mentally, we gear up as if it were. If we lack the preparation and resources needed, the notion of a potential threat can activate a stress response similar to having already failed the exam. On the flip side, this same thought of an approaching exam could serve as a helpful prompt, nudging us to get ready for it and offering a chance to demonstrate our knowledge and skills. It hinges on whether we perceive the event as threatening our basic needs. What might excite one person could terrify another, much like how a thunderstorm can evoke different feelings in different individuals.

When we possess confidence in our resources, weather-like events can energize and boost our confidence, proving that we can effectively navigate life's challenges. Conversely, when insecurity overwhelms us, we doubt our capacity to weather the storm. Consequently, our secure self-retreats, leaving the nervous system in charge. This activates our stress response, leading to reactions like fighting, fleeing, freezing, or fawning.

Much like trees taking root in soil, the conditions we grow in impact our ability to thrive. Toxic elements exist within the cultures and systems where we take root. For instance, experiencing systemic oppression, rooted in colonial beliefs that see diverse 'ways of being' as threats. These perceived threats encompass deviations from the dominant norm, covering biological, cultural, and personal distinctions – such as gender, skin color, ethnicity, and varying physical abilities.

Both overt and subtle forms of racism and discrimination can be ongoing threatening conditions. The solution often involves a combination of empowered actions to improve our environment and building the skills needed to navigate unchangeable factors. It's sometimes difficult to distinguish between the two! Imagine living in an environment where you are seen as a visible minority; altering people's perceptions and behavior toward you might not be realistic. Yet, fostering connections with those who genuinely understand you and cultivating the self-compassion to seek support when feeling unsafe can help you cope with the effects of discrimination. This helps bolster our resources and support network, empowering us to confront discrimination and racism from a more secure and grounded vantage point.

With deep-rooted strength, we're better equipped to detect and address common threats like uncertainty, relational tension, and the unpredictable dynamics in our personal and professional lives, thus preventing them from becoming chronic stressors. Ultimately, in settings where our basic human needs constantly face unchecked threats, our capacity to thrive becomes compromised, distracting us from our potential.

Pause to practice: Storm Watching

by Crosbie Watler MD, FRCPC

Challenges are objective events in the external domain. Many challenges are outside our ability to directly predict or control outcomes. We create a problem for ourselves when we fixate on outcomes, when all we control is process. The student can worry about the outcome of an exam or commit to a process of study.

Many of life's challenges are process challenges—health, relationships, financial planning. With each challenge, we need to identify whether we control the outcome directly, or process only. We can then commit fully to the process, detaching from outcomes we do not directly control.

This is the foundation of sport psychology, where the elite athlete commits to a process of training and preparation. On game day, the athlete steps on the field of play, grounded in present moment awareness, and away from any attachment to outcome. In so doing, she preserves her finite capacity for attention for actually doing better.

External challenges come and go like weather, but we are not the weather. We are the observer, the witness of the weather. Metaphorically, we can storm watch from a safe, secure place, there is space. Whatever happens in the weather of your life, at your essence you are no more than, or no less than.

Pause for a moment and contemplate the contrasting experiences of witnessing passing weather events as an observer, versus feeling adrift and vulnerable amidst them.

To adopt the perspective of an observer, engage in mindfulness, an intentional focus on the present moment with a sense of detachment. Connect to who is paying attention (this is you, as the observer)? Who is the witness of this moment, who am I? Our enduring and essential quality is awareness without thought. Some call it consciousness, the non-verbal stillness of being, nothing else required. We are already whole. In that space, I don't mind what happens and challenges become opportunities for noticing when we lose it and are called to return to true nature.

Breath can guide us there, shifting our attention, on purpose, from above the neck, to below the neck. Following our attention in and down and noticing the space it creates in the body. When we lose it, what have we lost? Simply space. That grounded felt sense of the space between our thoughts, where who we are is not hard to find, but impossible to avoid.

You can apply the R.A.I.N.S. acronym here, with a second "N" standing for **Non-Attachment**, which is explored further in a later section. In brief, non-attachment is about **leaning back into what *is***—rather than grasping for how we wish things would be. It creates space for clarity, discernment, and wise action. Without the stickiness of attachment, we tend to respond with greater ease and perspective.

RECOGNIZE: Recognize the external challenge in front of you. Acknowledge that some things may be beyond your direct control. Sense into your body—are you safe in this moment? Picture the challenge like passing weather: **you are the observer, not the storm**.

ALLOW: Allow emotions to rise, without trying to change or fix them. Commit to the process of feeling, while gently letting go of control over the outcome. Emotions are part of the weather too—they'll pass through.

INVESTIGATE: Turn inward with curiosity. What is arising within? Notice any judgments, urges to react, or stories you're telling yourself. Invite your awareness to return to the quiet stillness beneath it all.

NURTURE: Offer care to the emotions that are surfacing. Speak to yourself as you would to someone you love—a Dear Other. Reassure your nervous system that it doesn't need to fight or flee right now.

NON-ATTACHMENT: Lean back. Ask yourself: *Do I truly control the outcome, or can I only influence the process?* This step isn't about giving up—it's about releasing the grip. From this space, we can respond with more grounded discernment.

SIGNIFICANT THING: From that place of steadiness, choose your next step. Take a meaningful action—or an intentional pause—that helps resolve, reframe, or relate differently to the challenge. This is where clarity meets movement.

Securing our Roots in Community: Reconciling with Others

Community is not a place, a group, or theory. Community is a feeling in the body.

Community isn't just a group of people—it's a feeling that lives within us. It's the sense of being seen and welcomed as we are, held in unconditional positive regard, and supported by a relational space that affirms our belonging, safety, and worth. For this feeling to arise, mutual intention and trust are essential. Without them, the idea of community remains just that—an idea.

Drawing from the tree analogy introduced earlier (Figure 3), we can consider how trees thrive in a collective environment we call a forest. In healthy forests, trees share resources freely, giving and receiving as needed. A tree that requires support is not seen as a burden—it simply receives what's needed, without shame or hierarchy. Resources flow where they're most required, and when one tree thrives, the whole forest benefits. In the same way, when we cultivate community through mutual care, everyone has a better chance to grow and flourish.

Yet, many of us have been shaped by cultures that prioritize self-reliance, where we're taught to "go it alone" and prove our worth through performance. We are often rewarded for *doing*, but rarely for simply *being*. Competition becomes the norm, and we're told—directly or indirectly—that resources are limited. This belief in scarcity drives many of us to accumulate, outpace, and perform, even in the presence of abundance. It isolates us. It disconnects us.

In this narrative, vulnerability is framed as weakness—something that undermines our ability to compete. We're taught to hide it in order to survive. But this contradicts our deep human longing for authenticity, connection, and collaboration. Our DNA is wired for togetherness, yet we're often pulled into self-protection, leading to a deep incongruence between how we're wired to live and how we've been taught to survive.

This incongruence is contagious. The way we carry ourselves—the "energetic tune" we emit—can influence those around us. When surrounded by people stuck in survival mode, we often mirror that same guarded energy. But the opposite is also true: abundance, trust, and positive regard are just as contagious. When we're immersed in that kind of energy, we begin to soften. We begin to heal.

Unconditional positive regard can act as a kind of security medicine. When we're met with warmth, consistency, and care, we slowly begin to trust—not just others, but ourselves. As we learn to soak in relationships that reflect this kind of love and acceptance, we internalize the tune. With trust, we invite more parts of ourselves into the light. We learn to pace ourselves with care, allowing the body to lead and heal on its own terms.

Vulnerability becomes the bridge that allows others to meet our wounded selves—and in doing so, we gain the strength to reconnect our past, fragmented self with the present. This is how wholeness begins to return.

Our inner healing intelligence brings forward the parts of us that have remained untethered—guiding them home.

As Elder Geraldine Manson reminds us, reconnection with our mind, body, and spirit is what fuels our pilot light—a symbol of our whole, authentic self. True thriving is not about avoiding challenge, but about being able to stay present and in the driver's seat, even when things are hard. When we don't trust our ability to navigate life's challenges—when we lack the inner or outer safety to do so—our nervous system steps in as

protector. But in genuine relationships, where we are surrounded by the nourishing “tune” of community, we can begin to trust again.

From this place of safety and belonging, we become able to regulate, reconnect, and ultimately, become facilitators of healing—for ourselves and for others.

Relational Attachment and Coping Styles

During childhood, we learn how to connect with others based on how our parents connect with themselves and with us (Bowlby, 2012). For instance, if a parent struggles to connect with us or provide a sense of safety, we might interpret it as rejection. This can lead us to seek connection in other relationships, sometimes appearing clingy or needy. On the other hand, if a parent is overly clingy and needy, we might develop a tendency to avoid attaching to others, which can come across as emotionally distant.

Attachment styles represent how we naturally connect or attach to those around us. Recognizing these attachment tendencies allows us to interrupt old, unhelpful patterns. When our attachment patterns are out of sync, they mix with the noise of the stress response and defensive behaviors. Secure attachment (congruence), however, is linked to our level of self-compassion and our overall alignment at the mind-body-spirit level. From this point, self-consciousness fades, and our actions flow authentically from our being. Returning to this centered state empowers us to find meaning in challenges and approach them creatively and resourcefully (sense of coherence).

As illustrated in Figure 9, as adults, we tend to fall into one of three attachment categories, and the strength of our self-grounding influences the intensity of our tendencies in relationships. These categories include:

- Avoidant
- Secure
- Anxious

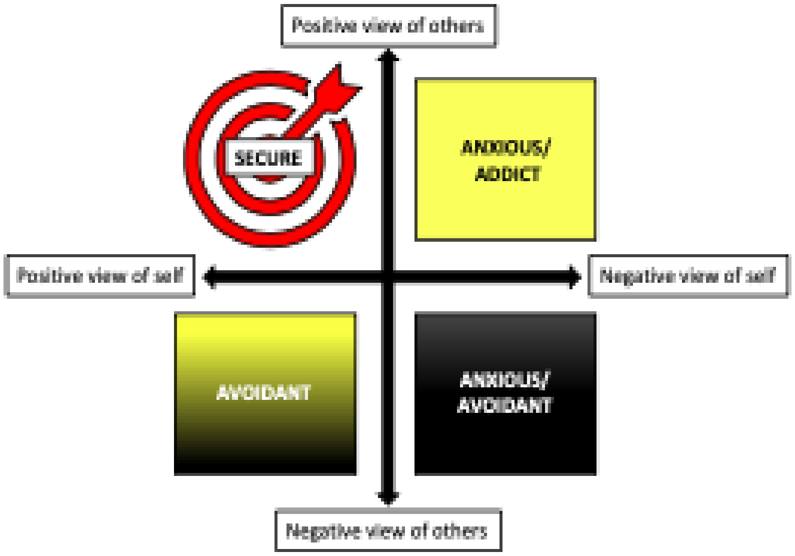

Figure 9: Attachment tendencies in relation to one's view of self and others.

Attachment tendencies aren't strictly black and white; they are general patterns with many exceptions. Most of us fall somewhere along the attachment spectrum, leaning towards either avoiding intimacy or anxiously seeking attachment to others. Those who naturally form secure attachments are in the minority. Keep in mind that these labels should be taken lightly. They're not meant to confine you, but to encourage awareness, curiosity, and objectivity. These traits are essential for working consciously with defensive behaviors that may not be beneficial to you.

The encouraging news is that attachment styles can change. Once we recognize our attachment style and the unhelpful reactions it activates, we gain the chance to heal the underlying distortions. Many of us haven't experienced secure attachments with others, nor do we feel securely attached to ourselves. Learning what secure attachment feels like can start by receiving it from others. Once we've experienced it externally, we can internalize it. By providing ourselves with this secure attachment, we can then extend it outward to others, including our children. This way, secure attachment can spread and reshape generational patterns.

Below is an adapted version of Vogel's Experience in Close Relationships Scale (Wei et al., 2007). Remember, these are tendencies, not fixed traits. Depending on the relationship and our mindset at a given moment, we can find ourselves on either end of the spectrum.

Anxious tendencies:

- I need people to frequently reassure me.
- I seem to want to be closer with people than they do.
- Sometimes my desire to be close to people scares them away.
- I fear I'll be abandoned.
- I seem to care more about other people than they care about me.
- I feel angry when my partner isn't available when I need them to be.
- When it's time to say goodbye, I draw it out, afraid to let go.

Avoidant tendencies:

- I rarely turn to others, especially to those close to me, in times of need.
- I long for close relationships, but I instinctually pull back.
- I don't rely on others for reassurance.
- I keep one foot outside the door of relationships most of the time.
- I avoid getting too close to others, it makes me nervous.
- I relish the intensity of a new relationship but typically withdraw from it after a little while.
- I don't usually bring up my 'real' problems with others.
- I don't like goodbyes or anything that's drawn out. I'd rather transitions happen quickly.

Secure tendencies (Brown & Elliott, 2016):

- I am safe and protected.
- I am supported to be my best self.
- I am seen, known, and understood.
- When hurt, I am soothed / comforted.
- Just by being myself, I am delightful.

Pause to Reflect: What are your attachment tendencies?

Which descriptors are you most resonating with? Write them below or mark the ones above that stand out for you. Considering past and/or current relationships - note if your tendencies change with different relationships.

Attachment Antidotes: Navigating Relationships with Choice

In this context, antidotes serve as remedies to address old attachment patterns. They are also the ‘significant things’, previously described as the final step in R.A.I.N.S. By consciously applying these antidotes, we break free from automatic reactions that no longer benefit us, empowering us to make deliberate choices. Working with attachment antidotes enhances our capacity to form secure connections with ourselves and others. This process nurtures congruence and a 'sense of coherence,' fostering the self-compassion needed for setting honest boundaries and feeling secure in our relationships.

For those prone to anxious attachment, building self-compassion leads to a secure self-attachment. This lessens the need to depend on others for reassurance.

Yearning for self-trust (anxious)? Antidote = REACH IN!

Individuals with anxious attachment patterns often focus their energy on others—monitoring relationships, seeking reassurance, and anticipating disconnection. One of the most powerful antidotes is to build self-trust. This means learning to securely attach to your own inner experience—your feelings, sensations, and needs.

By gently tuning into subtle emotional cues, you begin to meet them with curiosity rather than fear. Instead of letting them overwhelm you, you can recognize these sensations as messengers offering important insights. This process takes practice. (See the previous chapter for guidance on how to nurture this inner connection.)

It’s also helpful to cultivate diversity in your relationships, so that your emotional needs are spread across a broader support network—rather than relying too heavily on just one or two people.

For those with more avoidant attachment patterns, healing looks a little different. When avoidant individuals take the risk to set boundaries, name their needs, and express themselves more openly, they begin to foster trust with others. These small acts of vulnerability are essential building blocks for secure, reciprocal connection.

Lacking trust in others (avoidant)? Antidote = REACH OUT!

To build trust with others, honestly share your feelings and needs. Prioritize self-expression, even when it feels scary. This is a vital aspect of boundary-setting, especially for those uncomfortable with close relationships. By testing the boundaries of relationships through open communication, you establish a secure foundation.

Pause to Practice: Reflecting on Your Attachment Antidotes

Let's reflect on how you can practically apply these ideas to your own experiences. Think about the tendencies you recognized earlier, and consider how you might try new strategies in a time of conflict. Recall a past conflict that was stressful for you. How could you approach it differently? How might you apply an antidote described above to the situation?

It's important to proceed with patience. Avoid rushing, as pushing too quickly can take you beyond your comfort zone. Embracing new ways of behaving requires both self-compassion and a patient approach.

Pause to Strengthen: Cultivating Secure Attachment

Here, we offer affirmations embodying the five beliefs characteristic of a secure child and adult (Brown & Elliott, 2016). Speaking these affirmations with your own voice, addressing yourself as you would a cherished "other," can enhance their authenticity. Imagine the warmth and protection you'd extend to this "other" and speak the affirmations from that tender place.

Affirmations for Secure Attachment:

- I am secure and protected.
- I am encouraged to be my authentic self.
- I am deserving of being seen, known, and understood.
- When hurting, I am worthy of comfort and solace.
- Just as I am, I am wonderful.

How did this exercise feel for you? As you recited these affirmations, what sensations did you notice in your body?

Healing in a Community of Practice Structure

A compassionate community gives us the grace to grow—even when our growth is still messy. It offers space to become skillful in unskillful ways.

In *Roots to Thrive*, unconditional positive regard is our primary medicine, and Communities of Practice (CoPs) are the essential structure where that medicine is absorbed, integrated, and embodied. A Community of Practice refers to a group of individuals who share a common purpose and come together regularly to grow in that shared aim (Wenger-Trayner & Wenger-Trayner, 2015).

In our context, this shared purpose includes cultivating unconditional positive regard, expanding awareness, managing stress more effectively, deepening connection, and aligning with our inner calling. To support this, we rely on a structured framework, shared intentions, and mutual agreements—all designed to create the conditions for congruence (living authentically) and a strong sense of coherence (feeling life is manageable, meaningful, and understandable).

Structure helps us feel safe. It brings order to what can feel like chaos, reducing the anxiety that often accompanies uncertainty. Within this structure, the Community of Practice becomes a safe and supportive space to rediscover ourselves and each other. We do this by practicing authentic expression, and by compassionately witnessing one another as we speak from the heart.

From a neurological perspective, these practices activate key systems in the brain that support empathy, emotional regulation, and resilience. When we witness each other with presence and care, mirror neurons help us attune to the emotions of others, building trust and deepening connection (Bonini et al., 2022). Repeated experiences of being seen and accepted also engage the anterior cingulate cortex (ACC), which plays a vital role in helping us stay with discomfort, regulate our emotions, and take intentional action aligned with our values (Touroutoglou et al., 2020). Over time, this relational safety supports the nervous system in shifting out of stress states and into parasympathetic regulation, allowing us to feel grounded and connected.

A core method we use to build this foundation is the check-in process, which begins each group meeting (see "Pause to Practice" below). Through this simple but powerful ritual, we hold space for one another with presence and respect. We become mirrors for each other, reflecting the strengths, courage, and truth we perceive—often before we're able to see it in ourselves.

This mutual witnessing helps us soften and release old narratives and patterns that no longer serve us. As we feel safer, our emotional security increases, giving us the capacity to stay present even when we feel vulnerable. What once felt effortful—speaking our truth, staying with discomfort, offering compassion—begins to come with ease.

As we establish secure, meaningful connections with ourselves and others, the four core practices of resilience begin to unfold naturally:

- We become aware of our resources, both internal and external.
- We learn to co-regulate with the collective calm around us.
- We access compassion for ourselves and others.
- And we align with our calling, guided by meaning and purpose.

In this way, the Community of Practice becomes more than a structure—it becomes a living, breathing space for healing, where we remember who we are, and how to be together in a new way.

Intentional Communities of Practice: Working with our Narrative/Story
by Todd Haspect

One of the general themes we collectively work with in your community of practice is the concept of staying out of ‘story’ (or ‘narrative’ if that lands better for you). Our stories matter, but their usefulness in healing is limited. “Story” typically keeps us in our heads and focused on the past.

Staying present and dropping our focus into the body are two key practices we work on in our limited time together. These practices are meant to guide our check-ins, check-outs, and compassionate witnessing (you will learn more about these things in our first week)

I am inviting us all to lean into this. Whether we are responding to a weekly question or offering compassionate witnessing, it is normal to be pulled into our familiar stories. This is often connected to the patterns we want to shift in ourselves and so is valuable to notice. When we find stories popping up as we listen or respond, let’s pay attention to what our bodies tell us about this.

This takes practice. As you develop in your process, a Community of Practice (CoP) facilitator may offer a prompt in this regard. An example may look like, “I could hear you being pulled into story. This seems important. As you were speaking, what did you notice ‘below the neck’?”

Pause to Reflect: Who do you feel emotionally safe with?

Reflect on your current relationships and how they make you feel. It's possible that you don't have many relationships where you feel truly safe and accepted. This exercise aims to help you tune into your body's responses and identify individuals in your life who exhibit unconditional positive regard. These could be people you're not well-acquainted with or even individuals from your past. To begin, consider the following questions:

Where and with whom do you find yourself being the most self-conscious?

On the other hand, where and with whom do you feel the least self-conscious?

Who can you be completely honest with, without the fear of being rejected? For instance, who can you express disagreement to and still believe they'll accept you?

By participating in this relational space, supported by shared intentions and agreements that protect our emotional well-being, we nurture the trust necessary to cultivate a feeling of security and community. This offers us the chance to move beyond theoretical understanding and put our knowledge into action in our daily lives. It involves gently expanding our comfort zone in relationships to challenge deep-rooted patterns and beliefs that discourage vulnerability due to the fear of rejection (you can find more about this in the 'Window of Tolerance' section).

Through this process, we move from grasping concepts intellectually to gradually integrating them into our way of living, both within the community of practice and with the assistance of an assigned 'buddy'. Each of these practice aspects are outlined below and further explained in the Appendix.

Pausing to Strengthen: The Check-In

In the Roots to Thrive Program, we begin each weekly small group session with a 'check-in.' This straightforward yet impactful practice encourages us to connect with our bodies and engage authentically with others. Your task is to share 1 or 2 sensations you're experiencing in your body and 1 or 2 emotions. It's that simple. Some might find it challenging not to dive into the reasons behind their feelings. The point is to acknowledge your feelings as they are, without analyzing, judging, or providing explanations. This exercise fosters honest self-expression. For instance, if your back aches from gardening or you're tired after a night with your kids, instead of just saying "sore" or "tired," you might describe the sensation more specifically, such as "I feel a dull, burning sensation in my right leg." This doesn't disregard the importance of reasons; it's about honoring your feelings as valid, regardless of the causes. Similarly, regarding your emotions – whether it's sadness, anxiety, confusion, anger, disappointment, or anything else – just name the emotion and let it stand. No need to analyze or qualify.

Pausing to Strengthen: The Buddy System

In the first week, your facilitator will randomly pair you with a 'buddy' from your small group. You'll be encouraged to check in with each other once a week using a communication method you both agree on – whether it's Zoom, a phone call, text, email, or meeting in person. This touchpoint offers a chance to practice giving and receiving unconditional positive regard and also to express your needs and preferences.

The buddy system offers a brief but meaningful opportunity—just 5 to 15 minutes each week—to bring your authentic self into practice beyond the Community of Practice setting. Much like the vulnerability we explore within our small groups, connecting with a designated *buddy* allows us to continue that work in a more personal, one-on-one space. This relationship becomes a safe container for practicing real-world authenticity—showing up as you are, speaking from the heart, and receiving another with curiosity and compassion. It's an opportunity to be witnessed and to witness, grounded in unconditional positive regard, trust, and mutual respect.

Having a structured approach for these buddy check-ins, especially initially, is essential. It brings predictability and clear expectations, which in turn anchors the relational interaction, reducing the unease that comes with uncertainty or discomfort. Here's a format that past participants have found effective, which you can tailor to your preferences and needs, including the check-in duration:

1.) Quick check-in: Share 1-2 physical sensations and 1-2 emotions you're experiencing, providing detailed descriptions of bodily sensations, like "I sense a tingling feeling in my upper chest." When expressing an emotion, simply state it, such as "I feel joy." No need to add context.

2.) Deeper check-in: Each person delves into a deeper level, focusing more on emerging bodily sensations (often linked to recent experiences) rather than the specifics of the situation.

3.) Check-out: Once again, identify 1-2 physical sensations and 1-2 emotions without delving into explanations. Take note if there have been any changes since your initial check-in.

Re-friending the Body: Reconciling with Emotional Messengers

Elder Geraldine Manson refers to this inner signal as our pilot light. Often, the *noise* we are hearing is a combination of stuck energies in our body and **emotional transference** (when we feel others' emotions like they are our own) from others. The degree to which we can tune into our pilot light or signal and the authentic values, desires, and sense of meaning we find there, the less threatening the *noise* will be. When we address it from a place of strength, we are more likely to keep it in perspective so we can tend to compassionately as a dear "other", and when ready, clear it out. If we cannot clear out the noise, at the very least, we can turn the volume down.

Differentiating Signal from Noise

It used to be noise,
but now it is a signal
and I love the sound.
By Megan Mclaren, a fellow traveller

Some of us remember the days we listened to the radio with analogue dials. We turned the dial to get to our favorite radio station, and heard static as we moved the dial in between stations. The static was the noise, making it hard to hear the clear signal of the radio station. The closer we got to the station, the less noise. As illustrated in Figure 10, the 'signal' of who we are can easily be muffled by the 'noise' of who we are not (opinions of others, cultural conditioning, etc.). Consider our mental chatter as the noise and our unchangeable and inherently worthy essence as the signal of our BEing.

"The signal is the truth. The noise is what distracts us from the truth" (Silver, 2012, p.17)

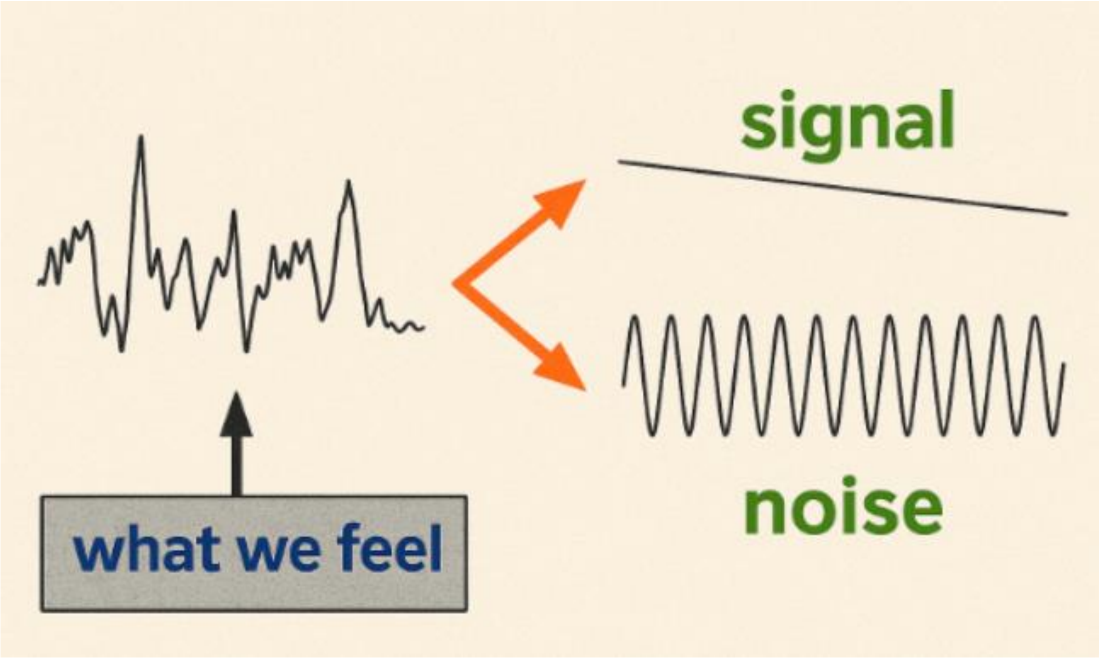

Figure 10: The "signal" of who we are at our core, versus the passing "noise" that swirls within and around us.

When the noise becomes overwhelming and distorts our sense of self, trapping us in overthinking, it's crucial to take a step back and expand our awareness of the present moment. For example, if thoughts about the future impacts of climate change fill our mind, we might begin to feel anxious and helpless, fixating on potential challenges that could arise in the next century. While addressing climate change is undeniably vital, dwelling excessively on worst-case scenarios can immobilize us, hindering our ability to contribute effectively to solutions.

To regain a more balanced perspective, we can engage in practices that help regulate our nervous systems, guiding us back into harmony with our authentic self. If you already possess methods that work well for you in this regard, that's fantastic! If not, you can explore several of the practices listed below and throughout this guide, noticing which ones resonate with your body and inner being.

Pause to Calm the Noise: The 4/7/8 Breath

"It's about learning to hear the whispers amid the screams" (Crystal Fee, a fellow traveller)

One of many ways to interrupt the stress response is with the 4-7-8 breath. It's simply inhaling for four seconds through the nose, holding for seven seconds, and exhaling through your mouth for eight seconds. It is normal to feel mildly lightheaded, so don't try this while driving.

When settled in a safe place (not while driving), begin by noting how anxious you are feeling on a scale from 1-10. What number would you assign? Then:

- Start by fully emptying your lungs with a long exhaling through your mouth.
- Inhale for four seconds through your nose (rest your tongue on your upper palate while you inhale).
- Hold the air in your lungs for seven seconds.
- Making a whoosh sound, exhale through your mouth for a full eight seconds.

Practice three complete rounds of this practice.

Now check in again - on a scale of 1-10, how anxious are you now? _____

Three rounds of this breathing are often enough to relax the nervous system. Adapt the practice to suit you. Continue until you've reached a state of calm, at least temporarily resolving the felt threat.

While this practice does not remove the event that felt threatening, it may provide the space necessary to step back from it so you can navigate the challenge more objectively and self-compassionately.

Sensations and Emotions of the Body

A key distinguishing characteristic of humans compared to animals is our heightened level of self-awareness, allowing us to differentiate between our physical bodies and the inner essence or consciousness within us. Understanding the emotional messages arising in the body improves what has been referred to as somatic intelligence, which links to our ability to connect securely with ourselves and others. As illustrated in Figure 11, Nummenmaa et al.(2018) demonstrates how typical emotions tend to be felt in the body, reminding us of our common humanity, and helping us to normalize how emotions and bodily sensations interact.

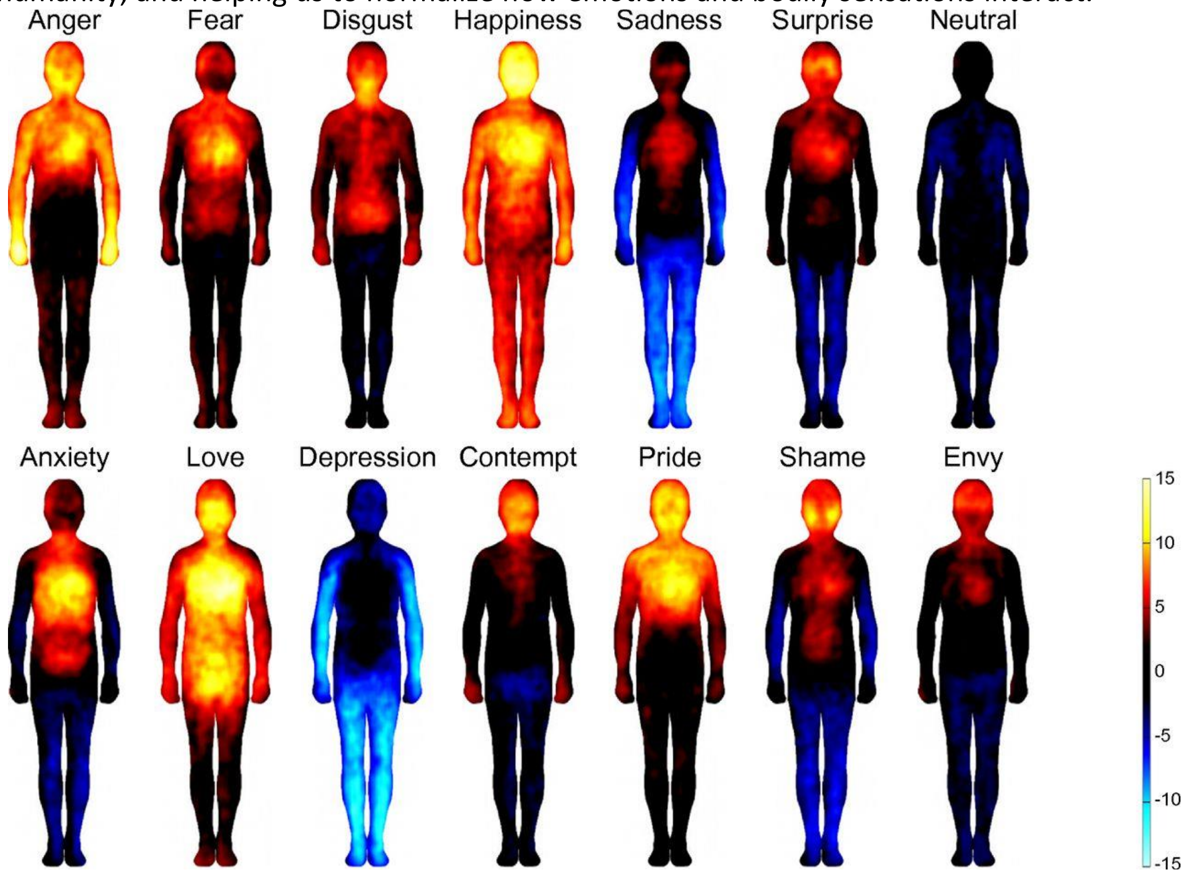

Figure 11. Typical sensations in the body. In >1000 people studied (Nummenmaa, 2018). The body maps show regions whose activation increased (warm colors) or decreased (cool colors) when feeling each emotion

Pause to Reflect: The Guest House

By Jelaluddin Rumi, translation by Coleman Barks (1997)

When we contemplate our practice of R.A.I.N.S, this poem by Rumi beautifully encapsulates the idea of welcoming visitors that arrive in our inner sanctuary, letting them bring the messages they are here to convey.

This being human is a guest house.
Every morning a new arrival.
A joy, a depression, a meanness,
some momentary awareness comes
as an unexpected visitor.
Welcome and entertain them all!
Even if they are a crowd of sorrows,
who violently sweep your house
empty of its furniture,
still, treat each guest honorably.
He may be clearing you out
for some new delight.
The dark thought, the shame, the malice.
meet them at the door laughing and invite them in.
Be grateful for whatever comes.
because each has been sent
as a guide from beyond.

Non-Attachment

A helpful strategy to develop self-compassion is to adopt a third-person perspective. This approach allows us to step back from intense emotional experiences, preventing them from overwhelming us. Imagine treating the body's sensations like cherished visitors. These sensations don't define us or reflect our worth; they serve as allies, conveying vital messages about the incongruities circulating within and around us. Paying attention to the body's cues—its sensations and emotions—fosters trust, reconciling our mind, Spirit and body. This encourages better communication, fostering a deeper connection and understanding within our whole selves.

Non-attachment comes into play when we step back (Figure 12), perceiving emotions as separate from ourselves, preventing over identification with them. As a result, we are less likely to perceive emotions as threatening, preventing us from getting lost in the stress response. This approach creates the inner space required to allow creative inspiration to flow in - fueling our pilot light and giving us access to the resources we need to metabolize strong emotions.

Non-attachment helps us welcome emotions, rather than feeling threatened by them, enabling the compassion required to metabolize them.

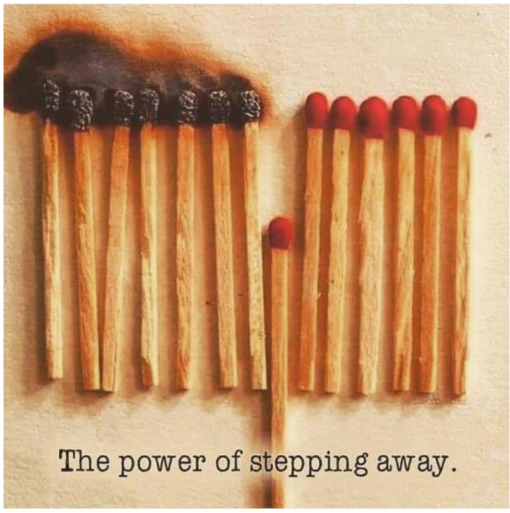

Figure 12: The power of stepping away from the stress response cascade (non-attachment).

Free from the noise of the nervous system, we become more curious to investigate our emotions. This curiosity enables a greater ability to allow and nurture - toward resolution or reorientation if needed. Thus, curiosity nurtures non-attachment, enabling us to view emotions as intriguing aspects rather than threats to ourselves. This compassionate perspective welcomes even uncomfortable emotions and sensations. We start seeing emotions as cues to pay attention and reminders to engage rather than cues to avoid them.

Pause to Reflect: What would it look like to Welcome and Respond to Cues from the Body?

These examples demonstrate how harmonizing with the body leads to more authentic, compassionate responses in everyday situations.

Example 1: Workplace Stress: At work, the fast-paced environment activates tension and a racing heart. Instead of dismissing these signals, I practice RAIN: Recognizing them as messages and pause. I Allow the sensations to exist without judgment. Investigating further, I realize they signal overwhelm. I take a short break, breathe deeply, and prioritize my well-being, Nurturing trust between my body and spirit. This self-compassionate approach helps me manage stress more effectively.

Example 2: Difficult Conversation: Before a tough talk with a friend, I notice a tight stomach and anxiety. Instead of ignoring these sensations, I see them as messengers. Allowing them without judgment, I detach by adopting a third-person view. I recognize these feelings as allies, not tied to my self-worth. Investigating deeper, I understand it's about potential conflict. With self-compassion, I reassure myself and approach the conversation mindfully. I might start by practicing RAIN, Recognizing and Allowing my emotions, investigating them further by fostering an open dialogue with them, nurturing them as I would a dear other.

Think about a recent instance when you noticed your body sending you signals or sensations. How did you initially respond to these messages? Now, consider how viewing these sensations as "dear guests" might have influenced your reaction. How could treating them with the same care and attention as welcome visitors have changed your experience in that moment? Practice this approach next time you encounter similar bodily cues and observe any shifts in your connection with yourself and your inner wisdom.

Pause to Strengthen: Sensations as Messengers

Take a moment to pause and tune into any sensations your body is sending you, acting as messengers. Cultivate curiosity by posing questions. For instance, what does your body need or want? If you sense fear, what is it afraid of? Does it have a name, like fear, sadness, or grief? Utilize the emotional descriptors provided in the Appendix if it helps.

Consider how it feels when you label the sensation with a descriptor. Does the label fit accurately, or does it need adjustment?

Approaching this way helps you step back, preventing over identification with emotional or physical discomfort. With this more objective perspective, create space for the sensation, like how you would hold space for a friend sharing something important.

Emotional Rhythm and Emotional Regulation

To allow for the unhindered flow of emotional expression, it's imperative to regulate the nervous system. When the nervous system is in an activated state, it tends to prioritize survival above all else, potentially stifling any room for emotional expression.

Emotional rhythm refers to the natural ebb and flow of our emotional experiences over time. Just as our bodies have physical rhythms like heartbeats and breathing patterns, our emotions also follow a rhythm. This rhythm encompasses the patterns and cycles of emotions that we go through, ranging from moments of heightened intensity to periods of calmness. Being in tune with our emotional rhythm allows us to navigate life with greater emotional intelligence and self-awareness. **Emotion regulation,** on the other hand, refers to the conscious strategies we employ to manage our emotions in response to various situations. It involves our ability to influence the intensity, duration, and expression of our emotions, ensuring that they are appropriate and adaptive to the context.

Both emotional rhythm and regulation are crucial for promoting emotional well-being and fostering a healthy relationship with our feelings. Often, the initial step involves emotional regulation, which sets the foundation for us to discover and align with our innate emotional rhythm.

Emotions as Phone Calls

Imagine your emotions as phone calls, relaying insights about your state of BEing, and conveying our inner state. Ideally, we answer these messengers, processing emotions fully. This aids in quieting inner chaos and reconnecting with authenticity. Yet, noisy external environments often hinder emotional attention, leading to frustration (fight), avoidance (flee), or suppression (freeze).
Just as with incoming calls, answering these emotional messengers yields relevant signals. Yet, ignoring them activates the stress response. How does your body respond when your emotional "phone" rings? To explore a range of emotions, consult Appendix A.

Chronically ignoring messages intensifies stress until addressed. Additionally, bypassing embedded signals hinders the healing of underlying wounds, prolongs suffering and heightens chronic dis-ease risks (O'Malley et al, 2015). Rather than shame, it's about embracing self-compassion. This stance offers the grace and space to heed the emotional "phone" when it rings.

Pause to Reflect: How do you respond to emotional guests?

Reflecting on Figure 13, consider your tendencies and circle the icon below that best describes how you tend to receive uncomfortable emotions.

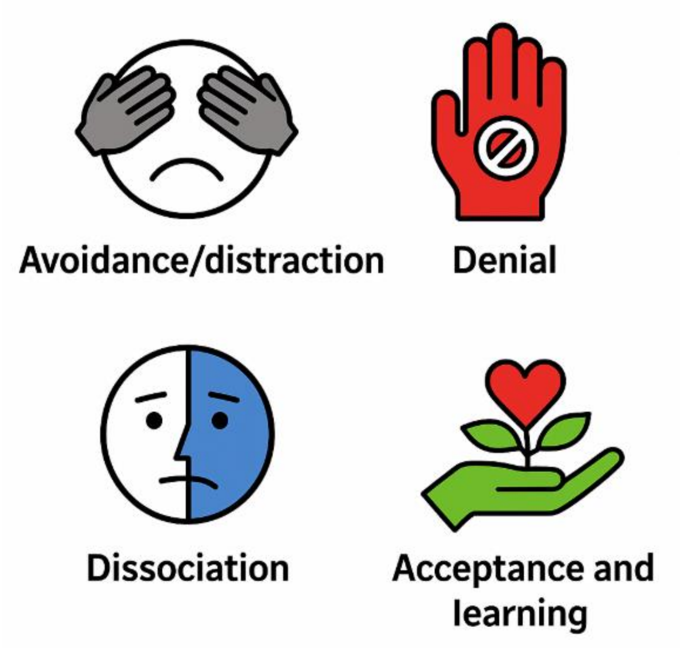

Figure 13: Typical reactions to emotions

Expanding Beyond the Physical: Energy Medicine

By Helen Watler

Physicists have corroborated what indigenous people have known since time immemorial: everything is energy and there are unseen realities we simply cannot sense. We can, however, be in relationship with unseen energies (Oschman, 2000).

Science tells us that 5 percent of the universe is visible, observable matter. Within this small fraction, the human eye can only perceive matter that emits light within a certain frequency. While birds can perceive magnetic fields and snakes can see in the infrared, we detect only visible light.

We can't see our energy systems, but they have been mapped by ancient cultures the world over. For example, we can learn about the meridian system from Chinese Medicine and chakras from Ayurvedic traditions. Indigenous cultures have extended this knowledge of an energetic presence to the land and all it contains. A key tenet is to look after the land, a responsibility passed down for thousands of years.

When we know about our energy system, we can learn ways to interact with it and help ourselves when life's inevitable challenges arise.

Energy exercises have been developed to help our energy systems re-pattern to remain free flowing, grounded and balanced. There are simple, easy practices that can help shift our nervous systems from fight/flight/freeze back to rest/digest mode.

The *Daily Energy Routine* series of exercises is a tool that helps to promote balance, health and vitality. It takes just a few minutes a day to complete the routine and will be reviewed in week 2 and a video link will be provided. If you are interested, you can view a video of the routine here https://www.youtube.com/watch?v=Di5Ua44iuXc&app=desktop.

Emotional Freedom Techniques (EFT) or 'tapping' is another effective tool and is explained briefly below. It will also be reviewed in week 2.

Pausing to Strengthen: Emotional Freedom Technique (EFT or "Tapping")

Emotional Freedom Techniques (EFT), also called "tapping," is a simple mind-body practice used to help regulate stress, emotions, and nervous system responses. It has been applied to everything from chronic paing, to PTSD symptoms, anxiety, and to reduce cravings to food and substances.

Is It Evidence-Based?

Yes. EFT has been shown in dozens of clinical studies to help reduce symptoms of anxiety, depression, PTSD, and chronic stress. A 2022 review of more than 40 clinical trials found that EFT meets accepted standards as an evidence-based treatment, with positive effects on both psychological and physical health. Reported benefits include reduced cortisol (stress hormone), improved heart rate variability, and lasting improvements

in mood and trauma symptoms (Church et al., 2022). Another 2024 meta-analysis of 18 randomized trials found that EFT significantly reduces depressive symptoms, especially for those in group settings or with moderate to severe depression (Seok & Kim, 2024).

When to Use It

You can use EFT when you're feeling anxious, overwhelmed, stuck, or emotionally activated. It's also a helpful practice before or after therapy, medicine sessions, or challenging conversations. You can tap quietly with a few words, or speak freely while tapping—whatever helps you stay present and connected with what's coming up.

How to Use It

It involves tapping with your fingertips on specific acupressure points while bringing gentle awareness to difficult thoughts or feelings. Tapping can reduce emotional intensity, calm the body, and create space for new insights or shifts.

Tapping can be practiced in different ways depending on your needs. In one approach, you say a setup phrase such as:
"Even though I feel [emotion], I deeply and completely accept myself." and repeat a few reminder words while tapping on a sequence of 8–10 points on the body.

EFT can also be more flexible and expressive. Many people tap while saying what they are honestly feeling in the moment—this could sound like venting, ranting, or naming thoughts that are hard to say out loud (e.g., "This isn't fair," "I'm scared they won't understand me," "I feel so tired of this," etc.). Both the structured and expressive forms help the body discharge stress and support emotional release.

The video below briefly explains the basic premise of EFT. The tapping points can be seen in the illustration. While the video is focused on reducing food and drink cravings, it contains a brief, helpful description of how 'tapping' can calm our nervous system and promote feelings of wellness.
https://www.youtube.com/watch?v=VpAICLoh8yg

EFT is a versatile tool. There are ways to use it on your own and ways it can be used with the help of a practitioner. You'll learn 4 easy techniques to use EFT on your own in the Community of Practice. You'll have a chance to sign up to work with a practitioner in a group setting to get a feel for how that works.

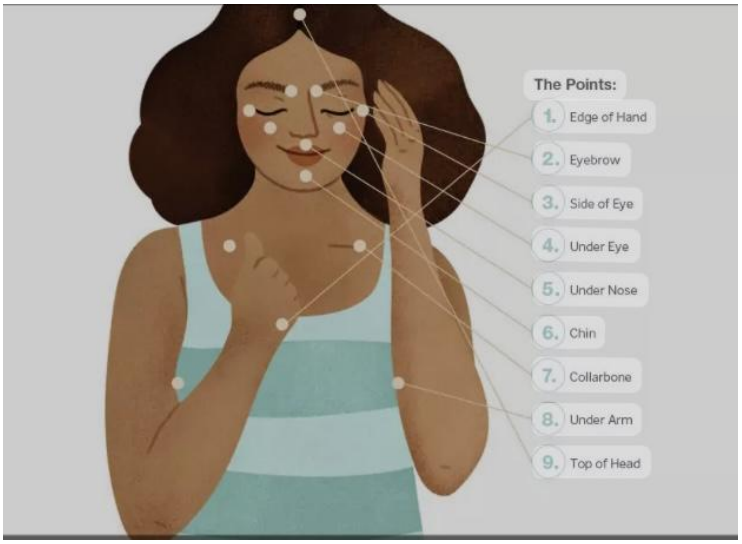

Unconditional Positive Regard (UPR)

Unconditional positive regard (UPR) involves accepting someone exactly as they are, without judgment or withdrawal of approval, even when mistakes are made, or they behave in an irritating manner. It's about recognizing each other's humanity, respecting our individual autonomy, and understanding that we are all doing our best with the resources available to us.

Most of us find it easier to give unconditional positive regard before we're ready to receive it ourselves. Often, we're more capable of offering to others what we desire for ourselves. As we engage in relationships where we're genuinely seen with unconditional positive regard, we gradually internalize the belief that we are genuinely accepted without any conditions. As previously mentioned, this internalization is akin to learning a new 'tune' within us. However, mastering this tune to the point where it becomes an inherent part of us is a process that takes time.

When we can receive this unconditional positive regard, we realize our inherent worthiness, separate from our actions or accomplishments. We also feel comfortable being truthful and setting boundaries that honor our needs and desires. This prevents the buildup of frustration or resentment, as we communicate clearly about our preferences and avoid saying 'yes' when we mean 'no'. Within this space of authentic expression, we can experiment with various ways of being and be vulnerable without fearing judgment or rejection.

Over time, as we become receptive to unconditional positive regard from others (as we start believing it might indeed be true), we acquire the ability to turn this same compassion inward, fostering self-compassion.

Pause to Reflect: How do we Embody UPR?

What's necessary for us to genuinely believe in unconditional positive regard?

- A conviction that we're accepted as we are, including our quirks and wounds.
- A belief that our worthiness is inherent, unrelated to our actions. This applies to everyone else as well.
- A belief that we can openly express our wants and needs.

What does it look like when we truly believe in unconditional positive regard?

- We feel free to express ourselves without fearing missteps.
- We're not afraid of rejection if we make mistakes.
- We celebrate differences in ourselves and others, instead of seeing them as threats.
- We can respectfully disagree and view conflict as a natural aspect of genuine relationships.
- We prioritize progress and the journey itself, valuing the process over the outcome.
- We recognize vulnerability as essential for both inner and outer connections.
- We form secure attachments with ourselves and with others.

Factors that encourage belief in unconditional positive regard:

A sense of connection with a higher power (or higher self) that we believe unconditionally regards us.
- The belief that we and others are doing our best given our current resources.
- Room for learning and growth when we make errors.
- Stepping back and investigating what is activating the stress response in us. We ask if the belief remains true now, even if it was valid in the past.
- Saying 'yes' when we genuinely want to and 'no' when we genuinely want to decline.
- Recognizing emotional projections creates space for introspection when we experience emotional pain, rather than blaming others. It also prevents us from taking others' behaviors personally.

"People are just as wonderful as sunsets if you let them be. When I look at a sunset, I don't find myself saying, 'Soften the orange a bit on the right-hand corner.' I don't try to control a sunset. I watch with awe as it unfolds." — Carl R. Rogers

What does conditional regard look like for you? How does it feel?

What would it feel like to genuinely believe you were unconditionally positively regarded?

It's crucial that we surround ourselves with individuals who see us as we really are; who encourage authentic expression; who can remind us of our essence (the *signal* within us), when we get lost in the *noise* of the world around us (or the nervous system within us).

Take a moment to consider who you feel truly seen by and who you feel safe expressing emotions with. Taking it a step further, when you react in ways you aren't proud of, whose unconditional positive regard remains steadfast?

Pay attention to your body. Notice and gravitate to the people in your lives who remind you of who you are when you forget, discerning the signal of your essence amidst the surrounding noise.

Self-Compassion: UPR Directed Inwardly

Many of us are familiar with the idea of self-compassion, but fully embracing it in our lives is a different story. The more we experience unconditional positive regard from others, the more we begin to believe in its truth.

It is like a radio frequency that we learn to embody through connections with others. Once we trust it and believe in it, we begin to recognize the feeling of self-compassion within us.When this belief becomes a part of who we are, self-compassion organically emerges. Self-compassion is a requirement of congruence. It provides the meaning required to overcome the fear of rejection, enabling the courage to be authentic.

Self-compassion serves as an anchor, connecting us to our inner selves and our inner strengths. It helps us perceive events and people without perceiving them as threats. As a result, we are better equipped to face challenges without activating our stress response. Imagine a skilled surfer facing big waves without anxiety. Similarly, high levels of self-compassion leads to reduced chronic anxiety and depression, increased joy, contentment, and confidence in navigating life's trials (Bluth et al., 2017; Dames, 2018; Gunnell et al., 2017; Homan & Sirois, 2017; Hwang et al., 2016; Kelly et al., 2014; Neff & Germer, 2018).

However, if we offer compassion primarily to "fix" a situation, make discomfort vanish, or artificially change our perspective to avoid unpleasant feelings, we're practicing *conditional* self-regard. This means we're attaching conditions on our worth, and on our ability to extend kindness to ourselves, leading to a lack of belief in our unconditional positive regard, both from ourselves and from others. In turn, we cannot truly practice self-compassion. The same principle applies when we try to extend compassion to others, which is hampered when we require them to meet specific conditions first

Without self-compassion, we stop BEing ourselves to start DOing what we feel is necessary to gain the acceptance we long for. We become perfectionists, fixated on what's wrong, or the conditions that appear to be a barrier to our worthiness. Perfectionism forces us to chase unrealistic standards to prove our worth. This leads to a lack of authenticity. In contrast, self-compassion counters shame and fuels self-acceptance.

When stuck in imperfection, we carry the tune of "I am not enough."

Self-compassion empowers us to view our awareness from a place of abundance, extending unconditional positive regard to ourselves.

When engaging in self-compassion, we carry the tune of "I am good enough."

Without any conscious choice involved, perfectionism can lead individuals to expect others to meet similarly unreachable standards. When we frequently feel disappointed by others (often spilling shame onto them), it could indicate a lack of compassion for ourselves. Conversely, by cultivating self-compassion for our bodies, we naturally begin to extend it to other bodies as well. This allows us to offer understanding and grace for their imperfections and honors that they too are becoming more skillful by practicing in unskillful ways.

Self-compassion creates an inner environment where we honor our priorities and needs as we do other's priorities and needs.

Our lived experiences and societal influences can lead us astray from our authentic selves, fueling a belief that our identity is tethered to our story. Consequently, we feel compelled to constantly validate ourselves, tirelessly efforting, but never satisfied. This perpetual cycle breeds insecurity, creating a disconnect between our inner and outer worlds. When our inner foundation is shaky, we instinctively seek external validation, be it through others' approval, substances, busyness or distractions. In doing so, we relinquish our autonomy, drowning out the distress signals that signify internal discord. Thus, shame inevitably arises because of this incongruence, serving as a reminder of our misalignment with our authentic selves.

Coping mechanisms can be viewed as *the* problem, with each maladaptive behaviour often labeled as a *disorder*, or we can view them all–people pleasing, food, sex, substances, screen time, shopping–as flowing from the same source: the trauma-disconnection interface that is becoming the norm modern society, with predictable downstream symptom clusters. Our overarching goal is to assess and remediate these symptom clusters at source, where persons can heal from the inside out.

Based on Neff's seminal research (2018), self-compassion has three core aspects:

1. Inward Unconditional Positive Regard: Treating ourselves as we would a close friend.
2. Common Humanity: Recognizing that everyone experiences difficulties and mistakes. Normalizing our own experiences.
3. Discomfort Acceptance: Stepping back to allow discomfort instead of suppressing it.

Ways to Cultivate Self-Compassion

- Seek Supportive Relationships: Surround yourself with people who offer unconditional positive regard. As you begin to show up more honestly and authentically in these relationships, you can test and begin to trust the acceptance they offer. Over time, this external validation helps you internalize a deeper sense of self-worth.
- Speak to Yourself with Kindness: Practice using gentle, nurturing self-talk—even if it feels unnatural at first. You don't have to feel deep self-love to begin speaking lovingly to yourself. With repetition, this practice becomes less effortful and more natural.
- Welcome Emotions as Visitors: Try to view emotions as temporary guests rather than fixed parts of your identity. When we see them this way, they become less threatening—and it becomes easier to feel, soothe, and release them without fear or avoidance.
- Challenge Limiting Beliefs: Gently investigate the old stories and inherited beliefs that block self-compassion. As you bring awareness to these patterns, space opens up for new ways of thinking, feeling, and relating to yourself.

Self-compassion, as you develop and embody it, becomes a guiding light for authentic living, resilience, and inner peace.

Pause to Reflect: What story of self are you living by?

The following questions can be helpful here. Take a moment to listen in, and reflect on:

- *How do you feel about yourself as a person?*
- *What academic grade–from A to F–would you give yourself for self-love, self-compassion?*
- *From strongly agree, to strongly disagree, what is your agreement with the following statements: I am a failure? I am enough?*

Pause to Reflect: How would you treat a dear friend?

Similar to a previous exercise where we focused on tending to our nervous system as a "Dear Other," consider a dear friend or family member. Imagine that they are turning to you during a challenging time. What words would you offer to comfort and encourage them?

Your response might go something like this: "Dear [Friend's Name], I can see how tough this situation is for you. It's truly challenging, and I understand why you're feeling this way. Remember, you're doing your best with the tools you have at this moment. Just the act of acknowledging these emotions is a great first step. Tell me about the sensations in your body right now?"

Take a moment to write out your imagined response.

Next time you find yourself in a place of vulnerability, find a quiet space to drop in for this important conversation.

BEing Self-Compassionate vs. DOing Self-Care

Self-care means different things to different people. For some, it offers permission to be kind to themselves. For others, it can feel like a chore or even selfish. But the moment self-care becomes a task we *should* do—driven by obligation rather than desire—it stops being an act of self-compassion.

Self-compassionate care is guided by what feels nurturing, not by what looks good from the outside. It's less about ticking off a to-do list and more about tuning into the needs of the body, heart, and spirit. That *wanting* we sometimes dismiss as selfish is often our body whispering what it needs to feel whole.
When we listen to those signals and respond with care, we build trust with ourselves. And when we tend to ourselves first, we don't take away from others—we actually have more to give. A full cup spills over naturally.

Re-Parenting and Re-Partnering Our Dislocated Parts

People who grow up surrounded by unconditional positive regard often carry a strong sense of self-compassion into adulthood. But for many, early environments were lacking in this kind of acceptance—and the result is a struggle to feel safe turning inward.

When we didn't receive the attuned care we needed, parts of us can remain *dislocated*—stuck in protective patterns and still scanning for safety. These wounds don't vanish with time; instead, they surface again and again, asking to be seen and healed. Often, our inner healing intelligence brings these parts forward during moments of stress or vulnerability—not to cause distress, but to invite integration.

These dislocated parts tend to view the world as unsafe and see themselves as a barrier to being loved or accepted. To truly heal, we need to call them home—not with force, but with compassion. This is where the work of re-parenting or re-partnering begins.

As the more resourced adult we are now, we can offer the care we may not have received in the past. We can return to these inner wounds with softness and the presence of someone who finally knows how to listen.

Through re-parenting and re-partnering, we begin to:

- Acknowledge these parts as unhealed wounds—not threats or flaws.
- Feel them fully, once we've created enough distance to know they are *part* of us, but not the whole.
- Nurture them with unconditional positive regard, offering what was missing.

With each encounter, we reclaim a piece of our true self—a part that once felt unsafe, unwanted, or unlovable. Every time an intense emotion rises, we're being offered an invitation: to sit with our younger selves, to listen, to hold them with care, and to remind them they are no longer alone.
This is how we bring all of ourselves home.

Pause to Strengthen: Transitioning from Identifying with to Caring for

Let's begin this practice with a clear intention: to receive whatever sensations and emotions arise within us as transient visitors in our inner sanctuary, each carrying an important message. It's crucial to understand that these messages don't define us, nor do they form our identity. They're fleeting messengers, aiding us in navigating challenges, reminding us of our values, and serving as protective guides.

Non-attachment: Our emotions can overwhelm us when we closely tie our identity to them. Instead of immersing ourselves in an emotional narrative (like saying "I'm sad"), let's delve into the multi-faceted layers of our feelings. Shift from stating "I am sad" to "I am noticing sadness in the body" (notice the absence of possessive pronouns). This shift helps alleviate the feeling of threat often associated with strong identification with emotions. It empowers you to regard emotions as transient guests with messages. This perspective fuels curiosity too: How does the sadness materialize within me? Where does it originate? How do I experience it? Does it ebb and flow?

With a newfound readiness to welcome our emotional guests, recall a situation that led to a sense of unease in your body. This event doesn't need to be monumental; simply choose a memory that comes to you. Allow the memory to unfold until you reconnect with the underlying feelings of stress.

Now, measure your current level of stress or anxiety on a scale of 1 to 10. How anxious do you feel right now? ________

Continue to observe this anxiety and any other emotions that arise. Allow them to surface without judgment.

Picture yourself engaging in a conversation with your emotions, treating them as you would a cherished friend who has arrived to share something significant – always with your best interests at heart. It might be helpful to put your response in writing, assisting you in forming new patterns that may initially feel challenging.

Your dialogue should ideally encompass these aspects:

Recognize the emotion. Literally name it. You can even say it aloud. "I am observing a sense of sadness in my body right now."

Normalize the experience of suffering – address your body directly: “I can sense that this is difficult for you. I'm here with you. You're not alone in this” (“I” represents your conscious, nurturing, wise self – the part that cares for your body).

Extend loving-kindness through nurturing words that foster acceptance, expression, and compassion for the emotion. “I deeply empathize with what you've been through. I'm sorry you didn't receive the support you needed when you needed it. It's comforting to know that you can now express this emotion in a safe and loving space.”

As you conclude this exercise, reflect on your emotions once again. On a scale of 1 to 10, how anxious do you feel now? _______.

Upon completing this dialogue, take a moment to reflect on your emotions once again. On a scale of 1 to 10, how anxious do you feel now? _______.

Pause to Strengthen: Self-Holding with the Butterfly Hug

(Artigas, Jarero, Mauer, López,Alcalá, 2000), adapted by Marcia McMillan

Read through it entirely, and then see if you can guide yourself through it. You may find it helpful to record yourself reading this out loud on your phone. You can then play it back and guide yourself through this activity.

The Butterfly Hug was developed by a Mexican psychologist named Lucina Artigas (Artigas et al., 2000), as she was delivering care to groups of children who had been traumatized by a hurricane in Acapulco in 1998. It's been around for over 20 years and it's effective with all age groups.

The goal of this technique is calming the nervous system. You can experience some wonderful effects with regular practice, which include befriending the body and even opening the heart.

So, let us begin.

Take two deep breaths, as you do feel your feet on the floor, your bottom on your seat and let your shoulders drop.

Cross your arms over your chest, with the middle finger of each hand just below the collar bone.
Hook your thumbs together - in the middle.

Try to keep your hands more upright, so they point towards the neck, rather than the upper arms, but don't strain.

Now imagine a beautiful butterfly is resting on your chest and flapping its wings, as you begin to tap with me: L and R, L and R, L and R.

Use a little pressure so you can feel your flesh give a bit under the touch.

Just tap and breath normally or you can do deeper breaths - whatever feels right for you.
That's right, just continue at whatever speed feels right for you, with eyes either closed or unfocused.

If things pass through your mind and body such as thoughts, images, sounds, feelings, maybe physical sensations. Just notice without the need to change, judge or push anything away. You can pretend what you are noticing is like clouds drifting by.

We will be ending soon, when you are ready, let your hands be still and rest on your chest, and open your eyes.

I close my practice with a little self-hug, like a pat or rub on my arms. Of course, this is completely optional and you may be curious to try it. If not, just put your hands down and take a deep cleansing breath in and out.

One last thought, this is something you can share with your children if you have them. Kids really love it! Or even with the child inside of you if that feels right.

Compassionate Witnessing: Outward UPR

by Wes Taylor

One powerful way we learn to practice giving unconditional positive regard is through cultivating mindful and heartful listening. We can listen at greater depth, using our own hearts and bodies to find greater attunement with others. When we can attune with one another, our nervous system can relax and find greater resilience through this interpersonal coherence and BEing. If we can learn to listen to others in a mindful-heartful way, we can then learn to listen to ourselves with kindness.

A concrete way we practice mindful-heartful listening is through **compassionate witnessing**. Compassionate witnessing is listening for another person's heart and spirit with our bodies and emotions, far more than simply listening with our minds. We are used to listening only with our minds - often preparing something meaningful to say in response, bringing in our similar experience, or advising them on best solutions. Compassionate witnessing encourages us to use our bodies as a resource to recognize what is alive within us as we listen, and to sense what is alive or awake in others as they describe their experiences. Being compassionately witnessed offers people the experience of being more fully seen and heard, and to know how their sharing has touched, moved, or resonated with us as listeners.

🧠 Neuroscience helps us understand why this kind of presence is so powerful. When we deeply attune to someone—really feel what they're feeling—our brains activate in similar ways through what's called the *mirror neuron system*. These special brain cells allow us to "mirror" another person's experience, helping us connect through shared emotion and resonance. When someone feels seen and heard at this level, it supports healing not just emotionally, but neurologically (Bonini et al., 2022). The beautiful part? This connection is mutual—it calms the nervous system of the one witnessing *and* the one being witnessed.

Confused? That's ok. You will learn this through experiencing it; being compassionately witnessed by your group facilitators starting in week 1. In week 3 you will receive teaching on it, and then we will encourage you to start practicing compassionate witnessing in your small group. It may feel awkward and clunky. That is ok, and to be expected.

There are so many habitual, conditioned patterns in our verbal responses to hearing another person's struggles and pain. These typically miss the mark. The need to be deeply understood, so important for a healing experience, is stepped over when the responses go in this direction. Here is a list of some of the more common ones:

- Consoling - trying to make the person feel better, reassuring, encouraging
- Fixing/Advising - offering your ideas about how to solve the challenge
- Sympathizing - focusing on how you are feeling in response to what they have shared *"I've been there too" or "I can't believe it! I'm so angry for you - that is so unfair…"*
- Educating - trying to get them to see how they can change their thinking or actions to make things better
- 'One-upping' - telling a story of a worse situation you have endured
- Correcting - showing them how they must have misinterpreted intentions or events
- Interrogating - asking lots of questions "to help them explore" the issue
- Analyzing - explaining to them the dynamics going on in them or in the situation
- Interpreting - diagnosing the issue

All of us will have responded in some, or all, of these ways and will continue to do so. We may have even been professionally trained to listen in some of these ways. While some responses have their usefulness in certain situations, we encourage you to lean away from these habitual replies and lean into compassionate witnessing.

When we step away from listening with the typical intent to fix/advise/console/ etc., we are inviting our fellow group members into presence as they tune into their own inner pilot light. Our attention on their heart and spirit (instead of the story) can guide their attention to these deeper qualities in themselves and result in greater self-connection. We are also demonstrating that we believe they are capable - they do not require fixing, because they are not broken - there is ample healing intelligence within them. It is a small but powerful way that we can say *“I hold you completely able and whole.”*

Furthermore, responding with compassionate witnessing can be powerful medicine for the witness*er* - especially those of us conditioned to ‘fix’ others. The witnesser is now in the practice of attending to our essence and away from the DOing mind. Being in this practice for others will strengthen our ability to attend to the same qualities within ourselves. When we realize we don’t have to ‘DO’ anything in these moments other than to be present and listen with our hearts, our own nervous systems can settle. We don’t have to ‘fix’ anyone (again, because none of us is broken).

To start compassionate witnessing, consider the following:

- Being fully present (returning your attention to what is happening right now)
- Listen deeply for the essence of their internal experience
- Writing down words that resonate (if helpful)
- Recognize what the feeling is that may not be articulated or spoken
- Recognize what the core need or value is that may not be articulated or spoken
- Reflect to the speaker the deeper meaning you have heard that may be their heart’s or spirit’s longing (e.g. the desire for peace; relief from suffering; mutual respect, etc.)

(adapted from Rosenberg, 2015 and Weingarten, 2003)

It’s not expected that you provide compassionate witnessing for each person who shares. As you provide compassionate witnessing, you may include some of the following:

• *When I heard you speaking of*… (you can use their own words here)
• *I felt*… (physical sensation and emotion)
• *I sensed that* …… (deep value / core need / longing) is important to you.

It could sound like this:

“John, when you said that you feel as much anger as you do, I felt tightness in my chest and felt nervous. I got the sense that you just want justice and fairness in this situation - it really matters to you”.

This is not a formula. Authentic expression of compassion is the main idea. The message, however it sounds, needs to be focused on these three sentiments:

I see you.

You matter.

I'm with you.

There will be times where you will want so badly to advise, 'cheerlead', or reassure. That is ok. We default to these responses because they make us feel we are DOing something. It is very challenging to sit with the distress of others. It can make us feel helpless, and when we are accustomed to DOing to prove our worth, this can become very painful.

So, when you inadvertently return to the familiar pattern of advising, identifying, trying to make each other feel better, etc., our facilitators will gently guide you back toward just BEing a compassionate witness.

We are - all of us - learning to be skillful, gently moving beyond our un-skillful ways.

Pause to Practice: From Fixing to Deep Listening

To practice transitioning from fixing to deep listening, you can:

1. Listen for the typical responses of fixing, advising, educating, analyzing, etc. in the dialogues you overhear from others in public, or in your novels or TV shows. You will be amazed at how consistent these patterns are in our media and typical social situations. Make it a game - see if you can identify the variety of non-empathic responses using the list above - like a bingo game!
2. Put your attention on the heart and spirit of others in the dialogues you overhear in public, or in your novels or TV shows. See if you can guess at some of the deeper emotions and spiritual longings being expressed by what is being said (e.g. Dorothy in the Wizard of Oz, saying, "There's no place like home" is longing for safety, comfort, and familiarity; Chief Brody in Jaws saying," You're gonna need a bigger boat!" is feeling fear and wanting to be safe.)
3. Recall some upset or complaint that a family member was expressing recently:
    a. Recall your response - did you offer responses that fit the fix-it, correcting, analyzing or any of the other typical patterns listed above? Which one(s)?
    b. Write down your best guess at what might have been the deeper emotions and spiritual longings they were communicating? See if you can craft a verbal response including those guesses that you could imagine offering.

Fueling our Spiritual Pilot Light

"Each person is born with an unencumbered spot, free of expectation and regret, free of ambition and embarrassment, free of fear and worry; an umbilical spot of grace where we were each first touched by God. It is this spot of grace that issues peace. Psychologists call this spot the psyche, theologians call it the soul, Jung calls it the seat of the unconscious, Hindu masters call it atman, Buddhists call it dharma, Rilke calls it inwardness, Sufis call it qalb, and Jesus calls it the Center of our Love". (Nepo, 2006)

The word spirituality can mean very different things to different people. Despite being commonly lumped in with religion, they are distinct concepts. A definition we have found useful comes from the Maori Program: *Just Therapy*

> *"Instead of the traditional European worldview that separates physical and spiritual values, we learned to respect the sacredness of all life. Spirituality for us is not centered on organized religion, but on the essential quality of relationship between people and their environment, people and other people, people and their heritage, and people and the numinous." (Waldegrave, 1990 p. 46)*

Spirituality not only serves as a source of hope but has also been associated with improvements in both mental and physical health (Wachholtz et al., 2017). Reclaiming spirituality involves shifting focus from physical characteristics, material possessions, and societal checkboxes toward a deeper connection with the inner spirit. This redirection is a challenging task, particularly in the dominant North American culture where external factors like appearance, possessions, and achievements often take precedence.

In understanding the whole self, regardless of individual belief systems, common functions unite us. We all possess a unique mind capable of higher thinking, a body that gathers information through the senses, keeping us safe, and providing us with the intuition and discernment necessary to remain congruent with our 'real' selves, and in alignment with our unique purpose and calling. Finally, we all have a Spirit within, which Elder Geraldine Manson refers to as the Pilot Light, serving as the conduit for ancestorial wisdom and our source of connection to all that is. For those who do not subscribe to the notion of a benevolent spiritual entity, the concept of the Pilot Light or inner Spirit may feel more accessible.

Operating from a state of BEing involves allowing our inner pilot light (Spirit within) to be our guide. Trust and confidence naturally develop as we experience the benefits of listening to its prompts, often communicated through our intuition. This gentle whisper of the Spirit, later described as our signal amid life's noise, becomes a guiding force. As trust deepens, our actions (DOing) effortlessly flow from our essence (BEing), empowering us to live out our calling with ease. Rituals play a crucial role as bridges, connecting our BEing with our DOing.

Ritual: Bridging DOing and BEing

Rituals serve as the DOings that remind us of our BEing.

Serving as sacred bridges between our daily actions (DOing) and the core of our being (BEing), rituals establish a rhythm that calls us into the present moment, quieting the distractions of life, so we can hear the gentle prompts of the spirit within. In this way, rituals become anchors, grounding us in the present and guiding our actions from a place of centered BEing.

Unconscious rituals, born from survival mode, can serve as reactive coping mechanisms, offering a break from overwhelming situations or temporarily numbing us. Yet, with conscious awareness, these rituals can shift us into abundance, reconnecting us with internal and external resources. Intentional rituals, cultivated through practice, evolve into habits that effortlessly anchor us in our state of BEing, amidst a world often consumed by constant DOing. Purposeful rituals, like mindful daily tasks or reflecting on food's origin, infuse meaning into actions, guiding us from conscious effort to habitual ease. They act as a catalyst for sustaining positive change, aligning our habits with our calling and bridging the ordinary to the spiritual.

Inner Healing Intelligence

Inner healing intelligence is woven throughout this curriculum. In essence, it centres on the belief that each of us possesses an intrinsic capacity for healing. Just as our body instinctively heals a cut, drawing on its inherent ability, we can tap into our emotional, spiritual, and mental healing potential. It points to the wisdom and untapped potential residing within us, guiding us toward holistic well-being. Some may call it Spirit, inner healer, innate wisdom, or deep insight. Just as a seed carries the intelligence to burgeon into a thriving plant, our inner healing intelligence steers us toward our optimal state.

Our context matters. Just as a plant displays signs of ill health when deprived of crucial components, our well-being may falter in an unsupportive environment. Comparable to a greenhouse providing the ideal conditions for plant growth, we can foster our inner healing intelligence with a positive and receptive internal atmosphere. Accessing this inner wisdom requires us to turn toward this inner signal, listening through the noise of mental clutter and self-criticism, and aligning with clarity.

Pause to Reflect: Coming into Relationship with your Inner Healer

Consider the analogy of a seed maturing into a plant; how you can create a nurturing environment for your own inner healer to thrive. What daily practices or habits could you engage in to foster the conditions necessary for your personal growth and well-being? Take a moment to jot down one action you can commit to, showing reverence for and cultivating your inner healing intelligence.

Pause to Practice: Inner Healing Intelligence and Parts of the Self

Visualize a challenging situation in your life, like an argument with a close friend. Connect with your inner healer by seeking guidance from a place of calm within yourself. Ask yourself: What really matters to you in this situation? How can you respond in a way that aligns with your well-being and values?

When we experience pain in a past relationship, it is common for present day events that remind us (even in the most subtle ways) of that painful time, to land us right back in what can feel like threatening territory. This happens because our inner healing intelligence is always trying to heal areas of stuckness (where past emotions are stuck in the body). If we are aware of this happening, we will be in a better position to turn toward the emotion, seeing the opportunity to heal a past wound in the present moment.

If you find yourself over-identifying with the emotion you are feeling, you might assign a name to the part of self that is coming forward. Doing so can help you distinguish the anxious or protective part from the inner healing intelligence that emerges. Just as we talked about before with the "signal" and the "noise," the anxious part often takes the forefront. This is why it's important to consciously tune in to the "signal" of your inner healing intelligence. This practice empowers you to confront challenges with a clear mind and authenticity, allowing you to make deliberate choices instead of impulsive reactions.

Let's put this into action. Think back to the last time your nervous system was activated, which may have led to a reaction you're not proud of. What questions could you have asked your inner healer in that moment?

Consider the different parts of yourself that surfaced during this process. What words could you offer to these parts to let them know they've been heard and that you'll take care of them? This exercise can help you build a deeper connection with your inner healing intelligence and foster a more compassionate relationship with yourself.

Liminal Space and the Window of Tolerance

The word "**liminal**," derived from the Latin term 'limen' meaning threshold, describes a space between familiar and unknown, between 'what was' and 'what comes next'. Think of it as a door between transition, waiting, and uncertainty. For instance, it's the period following a significant breakup or just before starting a new job. Author and Franciscan friar Richard Rohr eloquently characterizes this space as

> *"where we are betwixt and between the familiar and the completely unknown. There alone is our old world left behind, while we are not yet sure of the new existence. That's a good space where genuine newness can begin. Get there often and stay as long as you can by whatever means possible…This is the sacred space where the old world is able to fall apart, and a bigger world is revealed. If we don't encounter liminal space in our lives, we start idealizing normalcy." (Rohr, 1999)*

Transitions are a natural and recurring thread in all of our lives—and you may find yourself in one right now. This is the space between the old and the new, the familiar and the unknown. It can feel

disorienting, even uncomfortable. And yet, why welcome it? Because it's often within this uncertain, in-between space that breakthroughs happen.

These are the moments when something shifts—a limiting belief, a worn-out pattern, or energy that’s been stuck for years. These moments don’t usually come with clarity right away, but they pave the way for healing and growth.

Though the liminal space may feel uneasy, it is also rich with potential. It asks us to slow down, trust the process, and remain curious. This in-between is not a detour; it’s part of the path. An essential part of the journey where transformation quietly begins.

As illustrated in Figure 14, navigating this liminal space is aided by understanding your "**window of tolerance**," a concept that can help manage discomfort and foster an appreciation for these transitional periods. In simple terms, the window of tolerance refers to the range of arousal or stimulation that allows us to function optimally (Siegel, 1999). Think of hyperarousal as being like a car speeding too fast. This is linked to the fight or flight response and might manifest as trouble focusing, irritability, anger, panic, anxiety, and self-destructive behavior. On the flip side, hypoarousal is linked to the freeze response and can appear as exhaustion, depression, numbness, and feeling disconnected – this is the state of dissociation. People often move between these states. Trauma or extreme stress can increase the likelihood of becoming hyper- or hypo-aroused.

Each person's window of tolerance is unique, shaped by factors like neurobiology, early experiences, support networks, coping strategies, and environment. A wider window allows for better management of intense emotions and situations. However, those with a narrower window may find their emotions overwhelming. They might avoid new situations or people to stay within their comfort zone. Yet, continually limiting ourselves makes coping more difficult. Those with a broader window can handle intense emotions and situations without it significantly affecting their functioning.

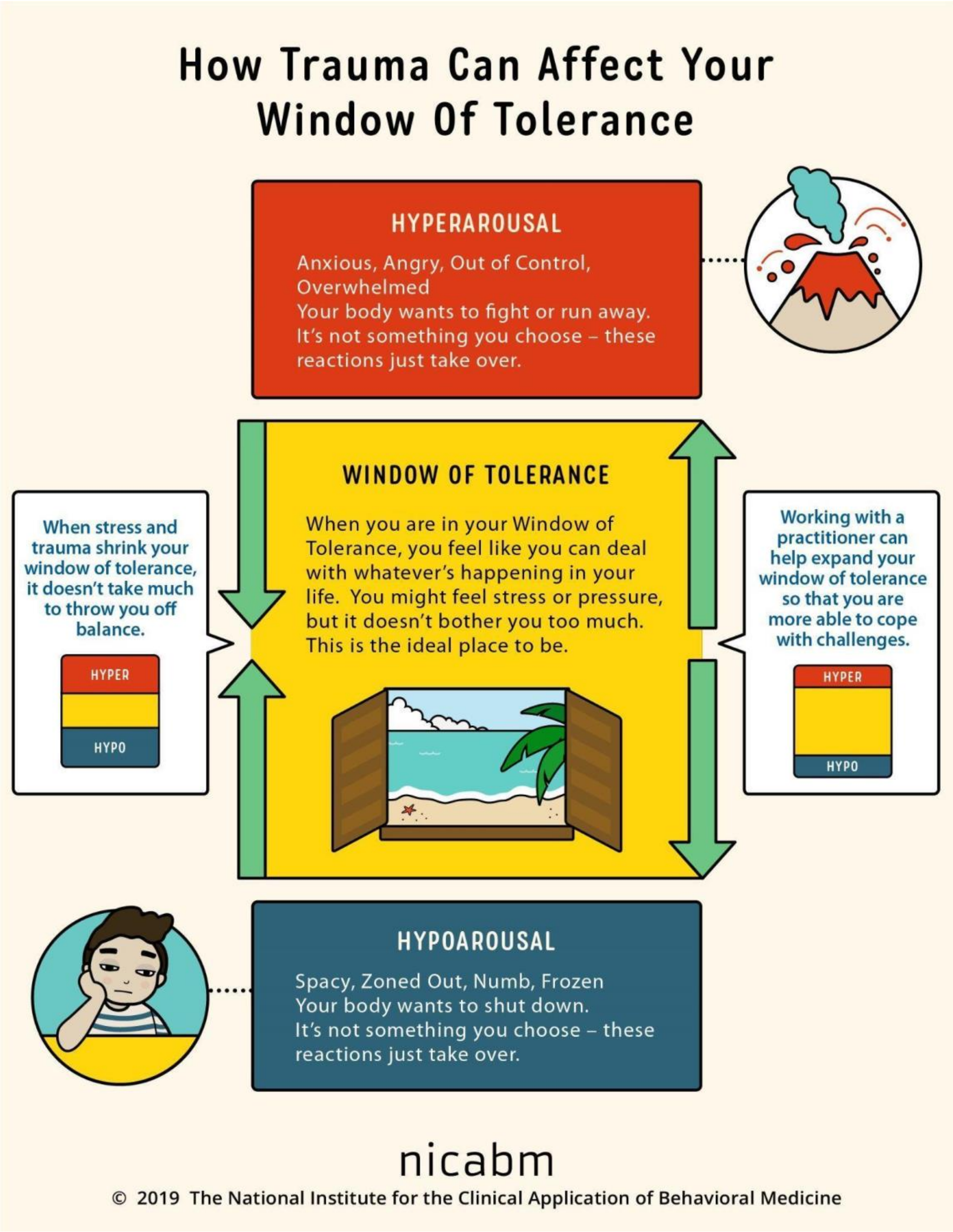

Figure 14: How trauma can affect your window of tolerance

To help manage the chaotic nature of liminal spaces, it's important to introduce a sense of predictable order. Adding structure can enhance a feeling of coherence during times of confusion. For example, if you're going through a period of uncertainty after a significant life change, you might establish a daily routine that includes activities like morning meditation, exercise, reading, and journaling. This consistent structure can provide a reliable anchor amidst the turbulence, helping you maintain a sense of stability and coherence.

Pause to Reflect: Recognizing Liminal Spaces

Reflect on a recent liminal space you encountered in your life. Did you notice any chaos or unpredictability during that time? How did you cope with the uncertainty? Consider how introducing a predictable routine or order could have helped you navigate that period with greater coherence and stability.

Reflections from a Fellow Traveller: "Liminal Space"
By Margaret Huml

I have crossed the threshold and even in moments when I think I want to go back I cannot. I am in a space between; a waiting space. Here I sit in this space – not the life I was living, left wondering when I will emerge on the 'other side' (whatever that means). I feel glimpses of anger, resentment, sadness. Sometimes they pull me in deep, and I want to go back to the discomfort of the way things were before, ironically, that felt more comfortable and somehow appealing.

When I look outside of myself, I see all that I think needs to change, and decisions that need to be made - a wave of overwhelm washes over me entirely. I have been trained that if I am not deciding, then I am indecisive, that if I am not DOING then I am not enough - so what happens now? In this space between, in this waiting, where nothing seems to be moving as quickly as I am used to.

I breathe.

I tap into my Being. And here I can see with new eyes. I can see that the healing timeline is a lie. There is no finish line, no decisions to be forced into existence. The path to healing is through - and how do I get through? - my noisy mind asks.

Sit and breathe my heart responds.

So I sit as best as I am able, for as long as I am able. Even when I want to run, I sit. I sit and breathe into the discomfort, into the ugliness, into the unraveling, into the stillness. I notice. And when I reach my maximum, I pause, I breathe again, I take a break (no shame) and come back to my BEing when I am able.

Maybe this liminal space, this space between, this waiting room? Maybe, just maybe, it is beautiful. Yes, it is wonderfully slow (according to societal standards) and it is unknown. I am grateful for the space of curiosity that is accessible here, so I tend to myself with gentleness, with compassion, with love. From a state of BEing.

Inevitably my noisy mind becomes restless and prodding and I come to a moment when I can no longer notice with curiosity, so I move with it. I wander in nature. I breathe. My chaotic mind tells me this walking is gluttonous, yet my body knows it is healing. Wandering in the woods, I am with *it* - the space between.

In this waiting space I search for anchors; anchors in nature, anchors in community, anchors in unconditional positive regard. So I show up here. Even when I feel messy. Even when I feel weary. Sad.

Ugly. I show up without the “right” words, without knowing the way. And I wait. There is beauty in the waiting. Even when it can feel excruciating.

Expansion and contraction are equally valuable. Though I prefer expansion – it ticks the box of “I’m doing it right” or “I am winning”.
And yet….
Contraction is the feeling in my body that represents a depth of meaning - can I meet myself where I am at, in this moment even when (especially when) I am not fond of this moment, this experience, this feeling? There is meaning here, so I wait. I cultivate self-compassion, because I need a load more of that.

I am grateful for this community where I am held in love. Where you hold me able to sit in uncomfortable spaces that I have not allowed myself to be before. And rather than greeting this space with tolerance (my typical grin & bear it approach), I am sitting, waiting to meet this space with compassion.

I remind myself sometimes the *significant thing* is to breathe. To sit. To wait. Healing is a process not a destination. This space we have created is a healing field – it is a sacred space that has been provided to me so that I may recognize what is whispering to be heard. To acknowledge it, to be with it, to tend to it and possibly to befriend it. This space is a gift, and although it feels turbulent at times, that’s ok. Here in this space I am not lost, I am found. I am connected with source. I am connected with my Being. I am connected with community.

When I can hear my heart it reminds me that a life wandering a healing path is a life well LIVED. And clearly it is a beautiful journey because it has gifted me each of you.

As I am in the middle of it and you are in the middle of it and the WORLD is in the middle of it, our healing ripples out to our community and throughout the world.

Soothing the Body

Soothing is a practice that describes how we work with our bodies to help them feel safe and nurtured. Because trauma often occurs when difficult events occur, without feelings of safety and nurturing, self-soothing (and specially allowing others to soothe us!) can prevent future trauma and to heal past trauma. It involves activities that help us feel confident in our safety, which in turn enables our bodies to relax, and emotions to be tended to.

Soothing practices can vary widely among individuals, as different activities resonate with different people. Common self-soothing techniques include deep breathing exercises, meditation, mindfulness, engaging in hobbies, taking a warm bath, listening to music, practicing gratitude, or spending time in nature. The choice of self-soothing activities often depends on personal preferences and what brings a sense of calm and reassurance to an individual.

The ability to soothe our bodies when they are feeling unsteady is essential skill in promoting mental and emotional well-being, empowering a sense of control as we move through challenging emotions with compassion and reducing the anxiety that results when we feel out of control.

Soothing in the Gap Between Awareness and Embodiment

Lasting change occurs when we undergo a spiritual reorientation—a shift in our entire frame of reference. As we spiritually evolve, old and unhelpful attachments naturally fall away over time. Yet, there's often a temporary gap between spiritual and behavioral shifts. Compassionate soothing serves as the bridge, offering grace in the struggle with outdated beliefs. Consciously navigating this transitional period is crucial to prevent the entrapment that arises when engulfed in shame. The more compassion we offer our bodies in this transition time, the quicker we will make it through the sticky spots that are inherent in the process.

Furthermore, successful self-soothing significantly reduces the likelihood of us grasping onto external substances to escape discomfort. It effectively de-escalates the nervous system, averting overwhelming reactions. By fostering a sense of inwardly directed unconditional positive regard, one's inner healing intelligence (the signal that emerges amid the noise) can guide what is called for in any given moment, which may be in the form of soothing words, breathing practices, connecting with loved ones who can remind us of our inherent worth and the resources available to us, connecting to nature, activities that spark a sense of desire and or fun, and physical touch.

Pause to Strengthen: Soothing with Breath

The breath is one soothing method that is always available to us, anytime, anywhere. Turning our attention to breathing is a quick way to ground – clearing out the "noise" so we can tune back into our "signal". Controlled breathing techniques that incorporate deep breathes, holding, yawning or sighing are especially helpful to alleviate stress, regulate, and relax the body (Corey et al., 2012; Vlemincx, Van Diest, & Van den Bergh, 2016).

Before you start, take note of how you are feeling.

While taking 5 deep breathes (inhaling through your nose and exhaling through your mouth), hold your breath for at least 2 seconds after you fully exhale. This promotes greater physical relaxation (Vlemincx et al., 2016). On the final exhale, let out a natural feeling sigh, which can help you more fully release areas of stuckness.

After the practice, spend a few moments to notice how your body responded. You can try different variations of this practice. For instance, the 4-7-8 breath (described earlier) is another excellent example of a regulating breathing technique. Those with a focus on the exhale are helpful to discharge excess energy from the body.

Soothing with Touch (Somatic resourcing)

When we feel physical pain, we instinctively soothe the injury by holding or rubbing the area. In a similar way, moving at the pace of trust and individual comfort, we can use touch to soothe emotional

pain. Soothing in this way improves the mind-body connection and regulates our physiology. As a result, interrupts the stress response, calms our cardiovascular system, reduces pain, improves our ability to concentrate and to absorb information, and strengthens our immune system (Lund et al., 2002; Uvnäs-Moberg & Petersson, 2010).

Touch is a tangible and effective tool to interrupt the stress response, calming the body and promoting a greater ability to step back from unhelpful thought patterns that continue to activate the nervous system. We can also use touch to tangibly secure our physical and energetic borders, reminding us where we stop, and others start. For instance, wrapping your arms around yourself in a protective self-hug, or imagining a protective bubble around us when we are taking on other's emotions as our own or when environmental stimuli feel overwhelming.

Clearing out the Sticky Bits

Stuckness, a term denoting a rigid attachment to emotional states, not only shapes but often dictates our perspective and subsequent behaviors. The process of **transmutation** facilitates a profound change in our state of being, allowing the adoption of new belief systems that naturally lead to transformed behaviors.

Recognizing that all emotions are integral to the human experience without inherent categorization as good or bad is crucial. However, dwelling in lower, dense emotions for extended periods diminishes one's sense of coherence, potentially leading to unease, dis-ease, and, ultimately, physical, mental-emotional, and spiritual ailments. Indigenous, Ayurvedic, and Chinese medicine emphasize that the roots of many diseases can be traced back to emotional, energetic, or ancestral factors. From a Western perspective, pioneers in quantum physics (McCraty, 2016; McCraty et al., 1995) have identified correlations between dense emotional vibrations, like anger, and adverse health outcomes, particularly cardiovascular issues. Simultaneously, they have observed that these vibrations can be transmuted by higher emotional energy frequencies, such as appreciation. Elevating the frequency of our vibration becomes a transformative practice, facilitating the transmutation of discordant (and at times destructive) emotions and energies that can get stuck within us.

Emotions such as anger, fear, hatred, grief, shame, resentment, guilt, and depression are classified as low or dense; they inherently carry a heavier and more discordant nature. On the other hand, curiosity, gratitude, forgiveness, and even carefully applied optimism and humor, represent significant transmuters with higher vibrational frequencies. These positive emotions serve as powerful tools for the process of transmutation, acting as 'significant things' that can effectively lighten the emotional load and enable the release of dense and burdensome feelings.

Transmuting 'Stuckness' with Gratitude

"When we practice gratitude for what we do have in our lives, this is a high frequency vibration that will help transform and transmute any discordant or destructive energies associated with a painful memory or experience. (Erika Gagnon, personal communication, November 5, 2023)"

Gratitude is one of the most powerful tools we can employ to shift our emotional trajectory. It happens when we focus on appreciating what we have rather than focussing on what we don't have. Our ability to feel grateful depends on our expectations and how deserving we feel. When we receive more than we think we deserve or when we receive more than we expected, we are going to feel more grateful. The more grateful we feel, the more contentment and joy (Watkins et al., 2018) we will experience. Added to general feelings of happiness and overall well-being (Wood & Maltby, 2009), when feeling grateful, we sleep better (Wood et al, 2009), have lower levels of stress (Solberg & Segerstrom, 2006), gain more satisfaction from our relationships, and are more spiritually tuned in (Bartlett & DeSteno, 2006).

It's not helpful to use gratitude to avoid difficult emotions that need to be felt. Feeling emotions is necessary to heal. If we don't feel emotions coming up, they will remain stuck in our body until we do.

To feel grateful, it requires us to pay attention to the positive aspects of life. We cultivate more gratitude by noticing the gifts we often take for granted. By immersing in gratitude, we can interrupt the powerlessness associated with when we get stuck in entitlement and victimhood. Gratitude is not about thinking positive thoughts, it's about immersing in an emotional state that enables us to shift our trajectory. Once we embody a more positive state of being, we interrupt the fear and powerlessness associated with the stress response, which enables us to keep things in a more optimistic perspective. From this empowered space, we can feel the emotions that need to be felt. By re-orienting our feeling state with gratitude, we interrupt the stress response and boost our confidence to navigate uncomfortable emotions and the associated life challenges (sense of coherence).

How Can I have Gratitude for Hardship?

Discovering healing, teaching, wisdom, or gifts from past experiences enables gratitude. Occasionally, lessons come through difficulty or pain, and it might be challenging to appreciate the process. By focusing on the ultimate gift gained, we learn to accept hardships as part of the journey toward the reward.

If understanding the teaching, wisdom, healing, or lesson is challenging, focus on a compassionate response to the part of yourself still entangled in the hardship. For example, acknowledging, "That was terrible. I will never allow that to happen again." This protective approach, essential in that moment, has now transformed into a source of wisdom that we can be grateful for. Therefore, no matter where you are on the path to forgiveness, the essence is that gratitude and forgiveness act as energies and emotions facilitating the transmutation of destructive memories and traumas. However, it's crucial to remember that it's easy to spiritually bypass if you are merely going through the motions at the level of the mind. Genuine healing involves including the whole self in the process. If you notice a block hindering your ability to go deeper, various healing approaches, such as talk therapy or somatic approaches, can assist in the process.

We are in no way condoning any negative experience, nor any actions done by someone else that has caused any harm, but remember we cannot change the past, and can only transform in the present moment. We are simply releasing ourselves from continuing to cause ourselves any further suffering, or self-destructive emotions, memories, thoughts and feelings.

"When one door of happiness closes, another opens; but often we look so long at the closed door that we do not see the one which has been opened for us" (Helen Keller, 1929).

Pause to Strengthen: Transmuting with Gratitude

When we engage in gratitude practices, our brain releases a pleasurable chemical (dopamine), which helps us develop habits that provide sparks of joy in the moment we are cultivating it. However, for this to be effective, it requires heartfelt engagement with the practice, not just going through cognitive motions.

Before you read through the list of practices that can cultivate gratitude, turn your focus to emotions and sensations in your body, rather than trying to evaluate them at the level of the mind. It is important that any new practices flow from inspiration, not obligation.

1. Recall one thing you are grateful for each day. Refer to it when challenges arise!
2. Recall one thing about yourself that you appreciate each day. Refer to it when you notice you are ruminating on self-critical thoughts.
3. When someone compliments you, say thank you rather than deflecting it.
4. When inadequacies seem to be especially apparent, remind yourself that we all have strengths and weaknesses, this is a normal part of being human. You are in good company!
5. Find creative ways to express gratitude, outside of the typical “thank you.” When we express gratitude more out of habit than out of a feeling state, it is far less effective. By getting creative, we are more likely to feel the gratitude infused in the message.
6. Focus more on what lies beneath the behaviour than the behaviour itself. While it can be more obvious to fixate on the symptoms of our pain (negative behaviours), practice focussing on how you can better tend to the wounds beneath the behaviours.
7. When you are longing for something you don’t have, practice re-orienting yourself by recalling how far you’ve come and how much you have right now.
8. Savor life’s pleasures. Savoring is that act of stretching out the moments that evoke positive feeling states. For instance, we can slow down and focus on the pleasures of a meal or we can melt into the pleasure of a long hug.
9. Much like gift giving, the giver of gratitude often receives even greater benefits than the receiver. Be creative and express gratitude in new ways, other than saying thank you. Coined phrases such as ‘thank you,” that are often offered habitually (subconsciously) are less effective than more specific and conscious offerings such as, “I am grateful for... ”. Additionally, spending the time to put a gratitude offering in writing can be even more effective for the giver and receiver.
10. Consider your mortality, doing your best to appreciate others as much as possible in the short time we are here.
11. Reflect on your connectedness. We are working within and contributing to a large and meaningful picture. From this perspective, even menial parts of our day can be meaningful. Viewing the world in this way, enables us to ask for help, as we are all working toward a common cause.
12. Tap into your inner joy, try subtly smiling as you go throughout your daily routine, notice the impact it has on your perspective and how others respond to you (mirroring effect).
13. Instead of saying sorry every time you make a mistake, consider if a sorry is the kindest response. For instance, perhaps you are running late; try focusing on thanking people for waiting, rather than berating yourself for being late.
14. When you are running behind on a task, instead of berating yourself for falling behind on a goal, try looking back at how much you have already accomplished.

15. Practice saying no to something you would typically grudgingly agree to. Celebrate the courage it took to say no (or stand up for yourself) like you would for a dear “other”.
16. Do something you desire as an act of appreciation!

What practices create a noticeable spark withing you?

What part of your day will you be most likely to make space for a regular gratitude practice?

Think of typical challenges in your day. What practice might be a good one to pull out when you most need it?

How will you remind yourself to engage in the practice?

Letting Go: *For*-Giving: What are you *Giving* your Freedom *For*?

“It is as though a destructive energy from the outside came into our lives the past when we were innocent, but as we continue to keep it alive in our memory, thoughts, feelings and stories we tell about ourselves, and in our mind and physical bodies, then the outside destructive energy, then becomes a self-destructive energy.” (Erika Gagnon, personal communication, November 6, 2023)

Forgiveness is an act of self-liberation that frees us from the cycle of dwelling on past hardships and allows us to release ongoing suffering. By accepting that the past is unchangeable, we can let go of the pain associated with holding on. Resentment activates our nervous system, creating chronic stress that affects our mental and physical health (Kelly, 2018). While forgiveness doesn’t alter the past, it changes how we respond to it. If we hold onto past wounds, we may subconsciously blame our suffering on current events and relationships.

Forgiveness is not forgetting. Instead, it provides a new way to remember past events, diffusing the negative emotions tied to them. By practicing forgiveness, we can rewrite our narratives. Although the details remain the same, our role shifts from victim to victor. This new perspective disrupts self-destructive thought patterns, allowing us to move forward without re-experiencing past violations. By recognizing the lessons within these experiences, we transform past suffering into present wisdom.

“Nothing ever happened in the past that can prevent you from being present now.” (Eckhart Tolle)

The journey of forgiveness takes time and effort. When feelings of anger or shame arise, we can approach them with compassion and curiosity, uncovering old wounds that need healing. Understanding that these emotions relate to past experiences rather than current events allows us to fully feel and release them. This process helps us rewrite unhelpful stories by learning from our experiences.

To navigate these emotions, it’s crucial to avoid interpreting them as threats, which can activate our nervous system. Instead, we can view them as opportunities. Cultivating non-attachment is essential;

stepping back allows us to reorient ourselves. Creating space between the sensation and our perception of it enables us to engage with our feelings without feeling overwhelmed.

To foster curiosity and non-attachment, try naming the emotion you're experiencing. This helps distinguish it as an "other," a third party that does not define you. Refer to the Appendices to explore the full spectrum of your emotions. If you struggle to see your feelings as separate, consider reaching out for support. Trusted individuals can help remind us of our inherent worth when we forget. Each time we treat our emotions as welcome "others," we take steps toward healing the wounds beneath them. With each encounter, as we lean into difficult emotions with compassion, we gradually forgive more until one day, the pain tied to past events dissipates entirely.

"I do not at all understand the mystery of grace—only that it meets us where we are but does not leave us where it found us." (Lamott, 1999)

Pause to Reflect: What are you giving your freedom for?

As adeptly asked by Elder and teacher Duncan Grady, what are you willing to give-for your freedom? (Personal communication, September, 2022).

Consider a past wound and related resentment you continue to hold onto. How does this wound continue to take up space in your day-to-day life (emotions, projections, behaviours)?

Pause to Strengthen: Transmuting with Forgiveness

"*Forgive yourself for not knowing what you didn't know before you learned it" (Maya Angelou).*

Releasing past wounds is a nuanced and intricate process, mirroring the complexity of the wounds themselves. Each act of letting go peels away a new layer, progressively lightening the burden and facilitating transmutation. To fully engage in this practice, we cannot simply go through the motions, we must engage the body by being present with sensations and emotions.

Feel free to use following practice whenever you sense resentment toward yourself or others. Adapt the words so they feel authentic to you. The practice can be brief, quiet, or out loud. Choose a space free from external distractions. This practice is one to repeat - often many times for emotions that continue to revisit, with words evolving or different memories surfacing. Some may find it helpful to turn this into a nightly ritual before sleep to loosen and clear any sticky emotions that are present at the end of the day.

The indication that a discordant emotion, like resentment, has been transmuted is when you no longer feel activated by it. However, be mindful that these feelings may resurface, signaling the existence of another layer that requires attention.

Please remember, this process is primarily for your benefit and is not contingent on receiving forgiveness from others, as that may not always happen. Your healing journey is independent of external factors. By transmuting the discordant energies within and taking steps toward repair, you can release attachment to outcomes, trusting that the positive effects will energetically extend beyond yourself in service to others.

PRACTICE READY?

When you feel ready (adequately resourced), start by envisioning another perspective. Think of someone you still hold resentment towards—someone who has wronged you, or perhaps, even yourself. Picture them sitting across from you in your mind, as you would with a friend you wish to connect with. Delve into your emotions, truly connecting to the moment and bringing their image to life.

As you envision the “other” sitting across from you, remind yourself that like you, they too experience pain and suffering. That from this place of overwhelming suffering, it naturally wells up to the point that it spills onto others. As a result, their suffering [subconsciously] spilled onto you, causing you to also suffer. Likewise, when your suffering is overwhelming, you too will [subconsciously] spill onto others. As you see them in this way, can you sense the weight of the suffering they carry, not so different from yours? Imagine they are taking stock of their past behaviours, sitting in the shame that naturally results. Take in the details of the expression on their face, their body position, and any other non-verbal forms of communication.

Embody through breath. When you have a felt sense of the experience, breathe in and out of your heart space. For those who are more visual, imagine your breath as a white light moving in and out with each inhale and exhale. For those resonate more with words, imagine breathing in mercy with each inhale and breathing out compassion with each exhale.

Embody the letting go. Coming back to the room, the sitting area, the quiet space between you and your guest, exhale and extend the white light or compassion toward them. Continue with this practice, paying attention to your felt sense, ensuring your body remains connected in the process. Continue until you notice a lightening in the body, a sense of peace in the experience. With this practice you are breathing in space between yourself and the resentment you have been holding. As you feel a sense of rest in the process, take the practice one step further, now paying attention to the spirit within them. Notice how their spirit, the “signal” [or essence] of who they are, is separate from their conditioning, their pain, and their resulting behaviours (“noise”). Continue to come back to your breath, sending them loving kindness, as you practiced earlier, with each exhale.

STOP OR CONTINUE?

You can stop here, or you can take this practice one step further by moving deeper into a general forgiveness practice, provided by Erika Gagnon (personal communication, November 5, 2023), who received the teaching from an Indigenous Andean Elder.

ASK for forgiveness. “I ask forgiveness if I have caused pain and suffering to anyone or anything, in anyway, whether conscious or unconscious, direct or indirect, visible or invisible... I ask forgiveness.”

OFFER forgiveness. "I offer forgiveness to anyone or anything else, if they ever caused me any pain & suffering in any way – Whether conscious or unconscious, directly or indirectly, visible or invisible... I offer forgiveness."

FORGIVE YOURSELF. "I forgive myself if I ever caused myself any pain & suffering in any way – whether I did it consciously or unconsciously, directly or indirectly, in the visible or invisible... I forgive myself.”

When you are ready, come back to the present moment, noticing how your body feels. End in gratitude by noticing what feels lighter within you, or whatever else you feel grateful for in this moment.

How did that go for you? How could you adapt the practice to sink deeper into the felt sense of it?

By engaging in forgiveness, empowered further through gratitude, you are severing the energetic cords that are stuck to discordant emotions, enabling the release of any lingering connections to others and past events. These ties, rooted in experiences that cannot be altered or erased, are being consciously cut, allowing for the liberation from the dense energies that when untended to, weigh us down and distract us from our highest purpose.

Significant Thing(s) and Optimistic Reframes

Significant things are intentional actions consciously taken to regain control during times of uncertainty. These actions fortify our sense of integrity, agency, and courage, guiding us and enabling us to live in alignment with our values and calling.

When confronted with challenging circumstances, it's akin to standing at a crossroads, offering us the opportunity to select our response. These responses may involve deciding on a specific course of action or intentionally refraining from action, allowing events to unfold naturally. Engaging in these deliberate actions empowers us to feel a sense of control amid uncertainty. This sense of control is pivotal in elevating our overall sense of coherence, as discussed earlier, and contributes to our well-being. Moreover, it creates an environment conducive to inspired living. It's crucial to emphasize that for the conscious and purposeful enactment of significant actions, the nervous system must feel secure enough to stand down (this is when self-soothing and regulation practices are imperative). Otherwise, we risk losing control to subconscious reactions, potentially derailing the possibility of an intentional response.

Order amid Chaos

Life often feels chaotic and unpredictable, particularly during challenging moments. However, by purposefully engaging in significant actions—deciding what to do or not to do—we bring an element of order into the situation. This order establishes a stable foundation, enabling us to navigate turbulence with greater clarity. Moreover, our choices in response to challenges not only shape our immediate reality but also influence our long-term perceptions and memories of the situation. Actively participating in significant actions allows us to mold our experiences, moving beyond mere reactions. This proactive stance doesn't just impact the present outcome; it cultivates resilience, reinforcing our capacity to bounce back from adversity. These intentional choices serve as reminders of our inner strength, fostering a mindset of growth and learning in the face of life's storms.

🧠 **Brain Science Behind Purposeful Action: Meet the Anterior Cingulate Cortex**

When we choose to take purposeful action—like calming ourselves before reacting, or planning our next step—we're using a part of the brain called the **anterior cingulate cortex (ACC)**. This region sits in the middle of the brain and plays a powerful role in helping us *notice* what's happening, *choose* how to respond, and *stay with it*—even when things are hard.

The ACC acts like a wise guide between our thoughts, emotions, and actions. It helps us:

- **Notice inner conflict** (like when part of us wants to yell, but another part wants to stay calm),
- **Stay connected to what matters most** (like our values),
- **Focus attention** even when we're overwhelmed,

- **Stick with important goals**, like healing, even when progress feels slow.

When we pause and choose a significant action—like taking a breath or asking for help—we're strengthening the ACC. Research shows that the *anterior mid-cingulate cortex*, a specific part of the ACC, plays a key role in helping us keep going when things get tough, supporting tenacity and effort toward meaningful goals (Touroutoglou et al., 2020). Over time, this strengthens our ability to stay congruent (aligned with our true self) and builds a stronger sense of coherence (feeling that life makes sense and that we can manage it).

🌱 **Why it matters:** Strengthening the ACC is like building a mental muscle that helps us find calm in chaos, courage in fear, and clarity when we feel stuck. When this part of our brain is active, we're more likely to feel that our actions are meaningful, that we have agency, and that we can grow through what we're going through.

Pause to Reflect: What does Purposeful Action Look Like?

Examples of significant things can vary widely depending on the situation. Consider recent events like the ones provided below. How might purposeful action help you navigate it more confidently?

<u>Conflict</u>
Scenario: You recently had a disagreement with a colleague at work, leading to heightened tension.
Action: Instead of reacting impulsively, you choose to pause, take a deep breath, and express your feelings calmly.
Result: This intentional choice defuses tension, fostering a more constructive conversation and promoting healthier communication.

<u>Overwhelm</u>
Scenario: Identify a situation where you felt overwhelmed by tasks.
Action: Imagine implementing a significant action, such as creating a prioritized to-do list to add a sense of order to the chaos of swirling tasks.
Result: Reflect on how this intentional planning might have provided a sense of order and control.

<u>Facing Fear</u>
Scenario: Think of a recent instance where you confronted a fear.
Action: Envision choosing small, gradual steps as significant actions, adding order to the chaos that presents with uncertainty, through an intentional step by step process.
Result: How might these intentional steps have helped you regain agency over your emotions?

Incorporating significant actions into our lives necessitates a conscious shift from reactivity to proactivity. Through intentional choices guided by our inner healing intelligence, we cultivate trust in our abilities, which enhances our sense of coherence. This trust becomes a cornerstone for navigating challenges with intention, resilience, and a renewed sense of control and confidence. This mindful practice gradually reshapes our interactions with the world, fostering continuous personal growth and empowerment.

Remember, the journey of healing unfolds one moment at a time, followed by one inspired action at a time. As trust with our inner healing intelligence grows, the next right action becomes more obvious with time.

The Optimistic Reframe

"If you don't like something, change it. If you can't change it, change your attitude" (Maya Angelou).

Those that tend toward optimism have significantly less stress than those who do not (Troy, 2015).
By re-orienting ourselves with a more optimistic lens, we bolster confidence in our ability to manage life's challenges, which improves sense of coherence. When we strategically use optimism to reframe our situation, we reduce stress levels and lower the risk of developing several chronic health conditions (Aldao, Nolen-Hoeksema, & Schweizer, 2010). In addition, people who are more optimistic tend to live 11 to 15% longer than those who aren't (Lee et al., 2019).

Strategic optimism is not helpful if it causes us to accept painful circumstances that are in our power to change or if we use it to avoid feeling difficult emotions. In fact, if we use optimism in this way, we may miss opportunities to tend to a wound that needs healing or to make changes that would benefit ourselves and others. As illustrated in the R.A.I.N.S, framework above, recognizing, allowing, investigating, and nurturing difficult emotions comes first. Recognizing our emotions and perception(s) of the situation allows us to gain awareness of our reaction. Allowance is the self-compassion to acknowledge our recognition. Part of investigating is determining what we can change and control. After tending to necessary emotions, it is then most helpful to take actions that reduce our suffering, thus nurturing ourselves where we are and where we intend to be. If we cannot make a change to reduce our suffering, this is the time to grieve the loss and then optimistically re-orient ourselves to the situation.

Optimism is *not* about merely thinking positive thoughts. For it to be effective, we must embody positive emotional state that enables us to shift our trajectory. Once we re-orient from this more optimistic place, we interrupt the fear and powerlessness associated with the stress response, which enables us to keep things in perspective. By expanding awareness in this way, we can recognize the opportunity in the challenge, improving our sense of coherence and reducing our stress in the process.

Working with Negative Bias

Most humans exhibit a natural inclination toward a negative bias, where negative events have a more profound and enduring impact on memory compared to positive ones (Gollan et al., 2016). While this bias can be advantageous in dangerous situations, it proves less helpful in day-to-day challenges. To counter this tendency, it is vital to consciously cultivate a more optimistic orientation. This involves a deliberate effort to recognize positive events and immerse ourselves in their felt sense. With time and dedication, we can retrain our brains to automatically notice positive occurrences, thereby balancing habitual negative bias.

It is important to note that leaning toward optimism is not the same as bypassing emotions that need to be felt to be released, even and especially the difficult ones. If optimism is applied to quickly, there is a

risk of bypassing difficult feelings by simply silver lining them, which is like applying a Band-Aid to a hemorrhaging wound.

Emotions are simply messengers that can only be heard through feeling. They are benevolent guests in our home, coming in a spirit of service. From this frame, it's imperative to notice, feel, and express all emotions without feeling the need to categorizing them as negative or positive. By refraining from such judgments, we reduce the risk of perceiving them as threats to be avoided, thereby activating the stress response.

The goal is not to evade feeling emotions but to choose how long we dwell in them. Neuroscientist Dr. Jill Taylor (2008) explains that emotions only last for about 90 seconds when we allow ourselves to be present with them. Each time we fully experience an emotion, we release another layer of density, lightening our load and making the process of transmutation possible. This transformative journey is further facilitated by developing gratitude and forgiveness, elevating our vibrational frequency to transmute elements that no longer serve our highest purpose.

"When one door of happiness closes, another opens; but often we look so long at the closed door that we do not see the one which has been opened for us" (Helen Keller, 1929).

Pause to Reflect: Transmuting with an Optimism (Empowering!) Reframe

The Serenity Prayer, a timeless reading often recited in 12-step groups, serves as a wise guide for amid life's challenges. Grounded in a philosophy of acceptance, optimistically reshaping elements within our control, and the sense of coherence necessary to enact the courage to carry it out, and the wisdom to distinguish between the changeable and the unchangeable.

"God, grant me the serenity to accept the things I cannot change;
Courage to change the things I can;
And wisdom to know the difference."
— Reinhold Niebuhr, 1892-1971

To apply this process, identify an unalterable challenge in your life. What aspects are within your power to change? Consider actions that grant you a sense of self-control unrelated to transforming the challenge itself. This could involve seeking additional resources, altering your role within the challenge, or adjusting your perspective and expectations.

For instance, take the scenario of a challenging family member or coworker who consistently seems to activate your nervous system. In many cases, complete removal may not be an option (identifying what cannot be reasonably changed) but what is in your control is the impact it has on you. As you cultivate some curiosity about what significant action could be possible, the potential for something new becomes possible (identifying what can be changed: the frame from which I view the threat).

On closer inspection, you may discover that their behavior echoes someone from your past evoking an old wound that this person is reminding you of (i.e. reminding you of the way a parent treated you, which led to feelings of inadequacy). While this person may have seemed like a threat in one frame.

From another, they are a teacher and an opportunity for healing. The past may have left you unequipped to address the wound at that time, but now that you are more resourced, this current day reminder of the past event is providing an opportunity to revisit it; to listen to its message; release the energy that's been waiting to be felt, and as a result, to lighten the load of stuck energies (trauma) you carry forward.

The Power of Humor in Coping and Resilience

Applying humor in a stressful situation has a remarkable ability to give us a fresh perspective on what can feel like a threatening challenge, enabling a loosening of our grip on rigid thought patterns. Because stress is deeply rooted in our perception of a situation, humor can help mitigate it, providing us with a less threatening frame (León-Pérez et al., 2021). All these elements improve our sense of control, which bolsters our sense of coherence, a core resilience factor in RTT.

Beyond its cognitive impact, the emotional aspects of humor, including joy and laughter, can have a positive psychophysiological effect too. It can enrich social connections, improve our ability to manage our emotions and can help us stay focused (Savage, Lujan, Thipparthi, et al., 2017).

While humor can be a valuable tool, it is also important to acknowledge that it can also function as a way of avoiding tension, or when used as an indirect form of communication, it can be hurtful, and can erode trust. Discernment is important here. You might ask yourself, am I using humor in this situation in a way that moves me toward connection or away from it?

Pause to Practice: A letter to myself

Sometimes our wiser selves need to remind our quivering selves of who we are when we forget. Love notes to ourselves enable us to break through the noise, making way for the signal within.

Take some time to write or record a message to your future struggling self, directly from your healthiest/highest self - the inner healing intelligence we introduced earlier.

This message/letter can serve as a reminder to you at a future moment in time of who you are, at your essence. It can be as simple as 3 or 4 post-it notes with some key messages that will remind your struggling self of who you truly are, it can be a full letter, or even a video to yourself. Whatever feels most congruent for you, lean into it and let your essence/pilot light shine on your struggling self!

We will be taking some time to speak to this integration practice in our COP next week, so it is important to create some space this week.

Generativity: Your Way Forward

Acknowledging our existing resources is important to bolster our confidence in our ability to navigate life’s challenges. This, in turn, improves our sense of coherence.

Use the roots of the tree in Figure 15 (below) to reflect on your inner resources—the qualities within you that keep you grounded and steady. These may include your values, strengths, passions, and the quiet confidence that helps you navigate life.

Then, look to the foliage of the tree to represent your outer resources—the people, practices, and environments that nourish you. These are the relationships and activities that help you remember who you are when you feel disconnected; the supports that help you return to your center, especially during moments of uncertainty or self-doubt.

Pause to Practice: Calling in my Resources

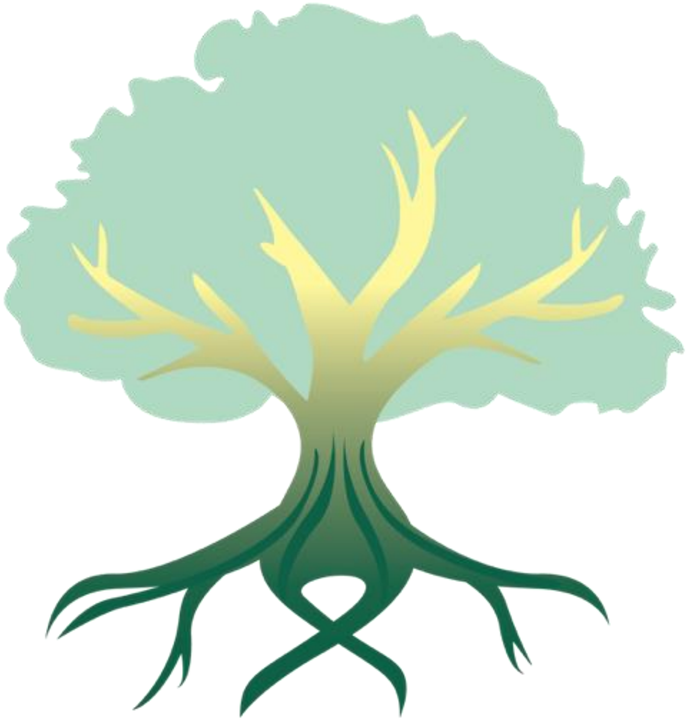

Figure 15: The roots represent your innate talents and inspirations. Your branches, foliage and fruits represent your material and relational resources. Use the tree to acknowledge your resources.

Sustaining Thriving Roots – A Way of Life
Wes Taylor

In the process of this journey, we've extended our roots, feeling into the nurturing soil that holds us. We need our roots to gather life energy from source – to help us remember the essence of who we are. These roots hold us steady in the storms. Strong winds carry the old away, clearing space to make way for something new.

How do we keep alive these profound experiences of unconditional positive regard, belonging in community, deep spiritual cleansing and awakening, and inspirations we've embodied along the way? This is the question of sustainability or generativity.

To do this, we make it a *Way of Life*. To live the essence of who I am as best I can. To do my best to use the various practices that I have learned to nourish my BEing. It is DOing to support BEing. Don't try to

fill your pockets in this last part of the journey. Don't look for those tools or practices that you have not yet picked up. Rather, review who you have become along the way. What have you come to know and experience as true about you? I'm not speaking of new positive beliefs, as beliefs are mental structures to which we cling to navigate in this world of form. Necessary, yes; but deeply insufficient for a fully lived life. Moving from a negative belief system into a positive belief system keeps our orientation on mental and abstract distractions from our essence. I'm speaking of the direct experience that happens before belief – the deep peace that you have touched, the truth that you *do* matter, you *do* belong, you *are* inextricably part of all that is… the truth that your essence is whole, undamaged, and pure. These are not concepts – but rather felt experiences that you have tasted, passed through, or simply now recognize. Return to these experienced, felt truths in whatever ways have worked for the past eleven weeks; those now familiar ways of placing your attention on your sensations, your breath, tapping, etc.

It is good to do a deep dive with our healing and growth periodically. You have just had one. And just like going to the dentist, having a deep cleaning once every 6 months (or 12 months in my case, as I really don't like going to the dentist) will not keep your teeth healthy if you don't tend to them daily. It must become a way of life – effortless, unquestioned, habit. What are those effortless habits of awareness, grounding, opening your heart in self-compassion, and alignment with your essence that you have already established? Keep it up. Those are your practices.

Your way of life is all about practice – Roots to Thrive has offered many. Neurons and neural networks that fire together, wire together - they get stronger and stronger. This is the neurobiology behind the value of practice. So please remember we are always practicing something – we are always strengthening some neural network or other. Choose wisely. As my friend Robert Gonzales wrote:

> "Many years ago, in a book I was reading by a popular spiritual teacher, a question was posed:
>
> '*What are you practicing? What are you meditating every moment?'* The implication of this question is that we are always focusing on something in our life. Our life *is* a meditation. The question is, "*What* are you meditating?" We have a choice. What is the nature of what we are meditating on? What are we conscious of, every day, in each moment of time? We can choose to have a conscious deliberate intention to focus our attention on presence and authenticity, or we can put our attention on the relatively unconscious and automatic mode of thinking and feeling and acting. If we do not have a deliberate spiritual practice that we use to focus our attention, then our attention feeds an automatic, unconscious repetition of patterns."

I certainly cannot talk about sustainability without mentioning the central role of community. You know this. You have lived and celebrated it week after week. Bottom line, we MUST have a supportive community to navigate this life with resilience. Trees do not survive on their own. Through their deep roots reaching out, intertwining, and with the support of the mycelial networks surrounding them, they pass nutrients to others when needed. They alert one another to approaching threats facilitating the enactment of protective mechanisms. They need each other. We need each other. The English poet, David Whyte spoke to the essential way we have created our community together:

> "…no matter the medicinal virtues of being a true friend or sustaining a long close relationship with another, the ultimate touchstone of friendship is not improvement, neither of the other nor of the self; the ultimate touchstone is *witness*, the privilege of having been seen by someone and

> the equal privilege of being granted the sight of the essence of another, to have walked with them and to have believed in them, and sometimes just to have accompanied them for however brief a span, on a journey impossible to accomplish alone." (Whyte, 2015)

So, double down with those communities or relationships where witnessing is happening. Ask yourself, what is one thing you can contribute to cultivate/deepen your community? And if you need to, find or create communities in which that quality of witness and unconditional positive regard is part of the DNA.

Now, here's the bad news about Roots to Thrive. It is a resilience program – no cures promised. For those of you who have had curative, healing experiences, you have been touched by grace and can trust that true healing does happen. At the same time, we are all going to get smacked in the face with the struggles and challenges that are part of life. Embrace the down times and know they will pass. They are unavoidable. They are not evidence of your failure or of the permanent return of your demons. They only confirm your humanness – and they are important messengers in this way.

At first, it takes a supported growth process to be able to tolerate the anguish and despair. Bit by bit, you have been learning to sit with uncomfortable emotions as your window of tolerance for suffering expands. As you continue healing, you can begin to even accept that these episodes happen and cease fighting when they make a visit. Then, over time, see if you can cultivate a sense of *welcome* – find that one bit of your essence that is undisturbed by pain, fear, shame, or despair – welcome the painful experience into your being and allow it to move through you and leave its wake. Allow yourself to be changed, reformed, reborn into each new moment. It is our clinging to calcified artifacts of identity (resistance to what is happening) that produces the deep suffering. As the great Sufi poet, Hafiz says:

Don't surrender your loneliness so quickly.
Let it cut more deep.
Let it ferment and season you
As few humans
Or even divine ingredients can.
Something missing in my heart tonight
Has made my eyes so soft,
My voice so tender,
My need for the divine
Absolutely clear.

When we embrace the moment of deep challenge and find a way to our tools, this is the most powerful type of practice**.** Utilize the tools when things are smooth, this is still valuable, and know if you can find your way to using these tools when you least want to, your resilience will be enriched the most. Lean into the hard times. Embrace them.

We cannot and will not stay in harmony, in perfect self-connection day in and day out. Success cannot be defined by always staying 'strong'. Our standard needs to be: how quickly can I notice that I am struggling and can then engage practices that fuel my pilot light? Or maybe find my pilot light again? Gentleness is a key and "good enough" is an enlightened way of being. The standard needs to be about the speed of recovery and presence of self-compassion.

Do not try to do it all! Pick 1-2 practices to focus on, test them, prove their value or eliminate them. Once you have established and integrated 1-2 valuable practices and they become part of your way of life, effortless, then see about adding others. The most important and effective tools are the ones that you actually USE.

Set and setting, while a huge focus of medicine journeys, are principles to which we need to attend in daily life. Cultivate your mindset with your mantras, calling statements, self-regulation practices, and open-hearted intentions. And pay close attention to your setting! What environments are you hanging out in? Do they nourish you? Are you walking directly on the earth, spending time touching moss, and smelling the forests? Are you surrounding yourself with relationships that are nurturing and nourishing? We will mirror those that we spend time with most – our deeply social brains – our communal neural networks have such influence on our daily experience. Ensure that your relational setting is designed intentionally rather than by default.

We are awash in the reminders to engage in deliberate gratitude these days. That is a powerful teaching. I want to align with that guidance not only with celebrating – consciously attending to/appreciating that which enriches life and dwelling in that experience for a while – but also with mourning. The painful aspects of life hold gifts within them. Be careful to not reactively push those experiences away using the new tools of tapping or 4-7-8 breathing, then call it self-connection. Feel the pain, loneliness, or grief – locate it in your body and breathe a bubble of space around it. As you hold it (yourself) with compassion and care, you can find the essence of the pain – it is something cherished, deeply valued that you have lost contact with. What a beautiful thing to hurt for. We only grieve for those qualities that are precious and held dear in our hearts. In this way, celebration and mourning are opposite sides of a single coin – a coin of incomparable value and meaning.

For me, this way of life is about living in surrender; surrendering to the flow of life. Discovering and gently removing those blocks and eddies in which I get stuck, in order to more fully re-enter the life-stream. Father Thomas Keating, Trappist Monk and teacher of Centering Prayer (the Christian version of meditation) said that we have but one action – one aspect of effort necessary for a spiritual way of life, and that is to “consent to the presence and activity of [the divine].” Just consent.

This way of life is expressed powerfully in a favourite poem from our fellow journeyer, Jalāl ad-Dīn Muhammad Rūmī. It is one that may unintentionally hint at the dissociated physical state of a transcendent Ketamine experience. His voice also speaks to me about this ordinary moment and the next, just as poignantly, in surrender and consent…

Zero circle

Be helpless, dumbfounded,
Unable to say yes or no.
Then a stretcher will come from grace
To gather us up.

We are too dull-eyed to see that beauty.
If we say we can, we’re lying.
If we say No, we don’t see it,

That No will behead us
And shut our window onto spirit.

So let us rather not be sure of anything,
Beside ourselves, and only that, so
Miraculous beings come running to help.
Crazed, lying in a zero circle, mute,
We shall be saying finally,
With tremendous eloquence, Lead us.
When we have totally surrendered to that beauty,
We shall be a mighty kindness.

Living Your Calling: The North Star Within

As fear begins to fade, it creates space within us. In this space, we start to feel the deeper longings of our heart—a calling that is no longer driven by fear but by a desire for meaning, purpose, and hope. While it's important to clear out the sticky belief systems and impulses that keep us focused on what's wrong, it’s just as vital to lift our gaze to the horizon, where a more profound calling takes hold. This is what we might call our inner North Star.

The North Star is unique because it remains fixed in the sky while other stars move around it, serving as a constant guide for explorers navigating the unknown. Often referred to as the "navel of the sky" or the "star of the sea," it has long symbolized the great spirit, guiding people on their life journey.
In the same way, we each have an internal North Star—a steady and reliable compass that anchors us in our deeper sense of purpose and meaning.

Pause to Practice: Calling Statement – A Bridge to your North Star

Set aside 10-15 minutes where you won’t be disturbed.

Ask yourself these questions, and jot down your thoughts without overthinking:

- What makes me feel alive and energized?
- When do I feel most connected to myself and others?
- What do I deeply care about or feel called to contribute to the world?
- If I could dedicate myself to one thing that brings purpose and meaning, what would it be?

Identify Common Themes: Look at your answers and highlight key words or phrases that repeat or stand out. These could be emotions, values, passions, or specific actions.

Draft Your Calling Statement: Using the themes you identified, write a short and simple statement that reflects your personal calling. Focus on clarity and heart. It could be something like:

"I am called to inspire creativity and connection in others."
"My purpose is to support healing and growth in my community."

"I strive to bring joy and understanding to those around me."
Refine and Reflect
Read your statement aloud. Does it feel true to you? If needed, tweak it until it resonates deeply.

Live with It: Keep your calling statement visible—on your desk, phone, or journal. Let it guide your decisions and remind you of what's important. Revisit it periodically and adjust as you grow.

Living in Harmony with Our Calling
Living in alignment with our calling is about more than simply pursuing our passions—it's about embodying a life guided by values, meaning, and inspiration. At the heart of this way of being is **integrity**: the state of being whole and undivided. Integrity calls us to stay true to our principles, even in the face of external pressure or uncertainty.

When we live with integrity, our thoughts, actions, and intentions become one—giving rise to authenticity and congruence. Within the context of our calling, integrity becomes a kind of inner compass, gently steering us toward choices that reflect our truest self.

You might imagine your calling as your heart's yearning—the spark that keeps your pilot light burning bright, the force that draws you forward with purpose. Integrity, then, is the bridge between that inner yearning and the actions you take in the world. Staying in sync with your values is what sustains that bridge, ensuring a consistent connection between who you are and how you live.

To follow your calling with integrity means making decisions not out of fear, approval-seeking, or trends, but from a grounded understanding of who you are and what truly matters to you. Your choices begin to flow from a sense of *good-enoughness*, rather than from a need to prove your worth. In this space, your actions and your essence begin to align.

Integrity also serves as a protective shield—guarding us from being pulled off course by the expectations of others. It empowers us to stay committed to our path, even when the road is unclear or difficult. Just as integrity reflects inner wholeness, living with integrity allows us to experience meaning, fulfillment, and peace—not only in reaching our goals, but in the way we walk the path itself.
At its core, honoring your calling through integrity is about respecting who you are, listening to your intuition, and honoring what you came here to do. When your aspirations and your actions are in harmony, you cultivate a life that feels true—and through that truth, a deep sense of purpose, significance, and contentment becomes possible.

"How we spend our days is how we spend our lives" Annie Dillard

A Final Blessing

As a final blessing on your journey, Marianne Williamson (1996) so eloquently reminds us of our inherent human worth and the power of radical authenticity on the path to individual and collective liberation,

Our Deepest Fear
By Marianne Williamsom

Our deepest fear is not that we are inadequate.
Our deepest fear is that we are powerful beyond measure.
It is our light, not our darkness
That most frightens us.

We ask ourselves
Who am I to be brilliant, gorgeous, talented, fabulous?
Actually, who are you not to be?
You are a child of God.

Your playing small
Does not serve the world.
There's nothing enlightened about shrinking
So that other people won't feel insecure around you.

We are all meant to shine,
As children do.
We were born to make manifest
The glory of God that is within us.

It's not just in some of us;
It's in everyone.

And as we let our own light shine,
We unconsciously give other people permission to do the same.
As we're liberated from our own fear,
Our presence automatically liberates others.

Appendix A: Emotional Descriptors

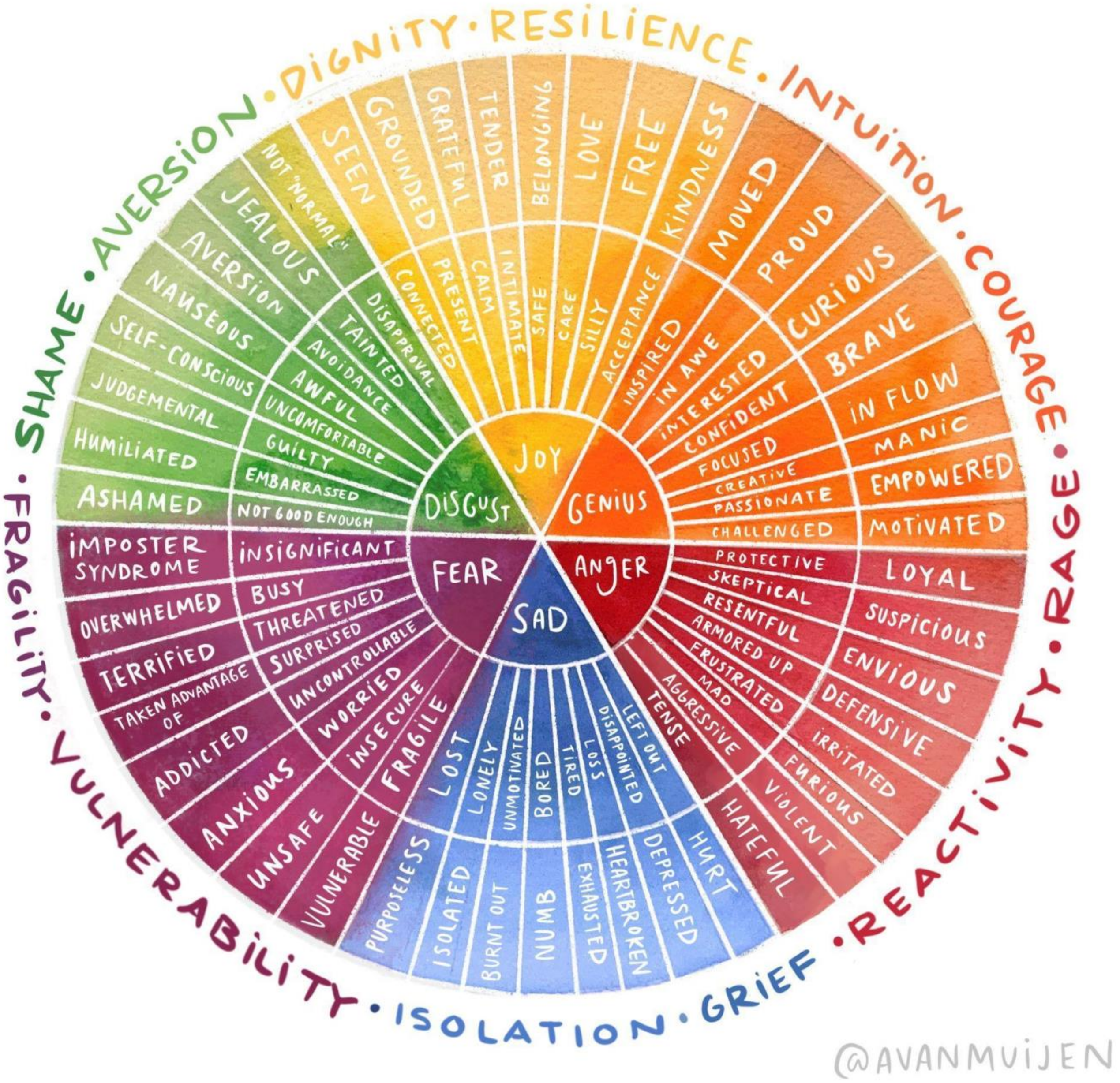

Appendix B: Psychedelic Medicines as Catalysts for Healing

In this section, we delve into the role of psychedelics as a catalyst for healing, with a specific focus on how they harmonize with the RTT framework of thriving. Different individuals find their unique pathways to well-being. Some might find resolution through therapy, self-regulation techniques, mindfulness, or medication, while others might require additional support to calm their nervous systems and access their inner realms. Seeking assistance to work with our biology is not a sign of weakness but rather a recognition of our individual needs, often influenced by ancestral traumas.

In western medicine, research is opening new doors to integrating psychedelics with psychotherapy. While still a subject of debate, emerging studies suggest their potential in promoting neuroplasticity, addressing trauma, and alleviating treatment-resistant depression and other mental health conditions (Ly et al., 2018). Medicinal adjuncts like psychedelics can take us to deeper levels of understanding, moving us beyond our ordinary ways of knowing and being. Through reconnecting with ourselves, we can release the shame and trauma that underlie our suffering.

While the allure of turning to psychedelic medicines as 'magic bullets' or 'last resorts' can be strong, we have come to firmly understand the importance of unconditional positive regard—it's the treasure awaiting us at the rainbow's end, undeniably THE essential element. Psychedelics, often referred to as mind-manifesting, possess the capacity to reconnect us with our core essence and nurture our human growth. However, it's crucial to recognize that they serve as a conduit rather than a direct source of healing.

These interventions that help us 'remember' are catalysts for healing, our healing allies, not the healers themselves.

Non-ordinary state of consciousness

The process involves moving from our ordinary state of consciousness to a **non-ordinary state of consciousness**, an expedition facilitated by the calming of the ego and its usual, well-worn thought patterns. By attaining this form of non-attachment (described earlier in the text), our defensive ego recedes, enabling us to objectively navigate past events and thought patterns. We start to see transgressions as the results of others' hurt and suffering, distancing ourselves from personalization. This shift grants us greater agency and objectivity, fostering a more positive, gracious view of ourselves and others.

We often use the term 'non-ordinary', because it marks a transition, the crossing of a threshold. As we navigate this transition, the veil of ordinary consciousness is momentarily lifted, allowing concealed energies to resurface—energies that might have been too intimidating to explore under normal circumstances. Thanks to the momentary disarming of the body's heightened threat alertness, we find ourselves more able to acknowledge and address past wounds from an objective and open hearted perspective. Viewing past pain and trauma through a more objective and loving lens enables us to transform shame and regrets with unconditional positive regard.

Furthermore, this shift often invites a flood of insights, unburdened by the influences of societal norms, offering us fresh perspectives on our lives, our interconnectedness, and the areas where we've felt stuck. When approached with healing intent, psychedelics can facilitate a benevolent connection with our inner selves, allowing us to practice self-compassion. For those who have lacked acceptance and

love, this newfound sense can empower them to embrace previously shunned aspects, cultivating self-compassion and congruence.

These medicines, through mechanisms like non-attachment and self-compassion (as explored earlier in the text), hold the potential to expedite the development of congruence and sense of coherence. Moreover, some psychedelics demonstrate neuroprotective and regenerative qualities, not only addressing past wounds that cause present-day stress, but also restoring brain regions linked to connection, insights, mindfulness, and cognitive performance (Ly et al., 2018).

With the realization that we are inherently whole and worthy, we begin to embody this truth. Psychedelics can lift the ego's veil, allowing us to perceive ourselves and others with love. This fosters unconditional positive regard and objectivity, enabling us to embrace and digest previously unacceptable parts of ourselves. As the ego recedes, we experience 'ah ha' moments, shifting our perspective and building trust in a bigger picture. This secure spiritual understanding empowers us to navigate external pressures with resilience.

Managing expectations

It is important to mention that returning to a balanced state of being can be challenging due to past wounds and biological imbalances, making inner exploration painful or difficult. They aid in our remembrance and healing, are allies in our journey, but they are not the sole healers. When using medicines and processes as catalysts for healing, it’s important to keep the focus on inner and outer connection as the primary mechanism of action, which reduces the potential harms that can result from fixating on the chemicals or processes that are facilitators of the connection required for our healing.

The Achilles’ heel of psychedelic therapy work is making it another DOing. Judging the experience. Feeling disappointment, even shame or anger that our experience is not enough. Forcing an intention or agenda. Attachment, judgment. *‘Are we there yet?’* Judging whether we are getting “better” quickly enough and in a straight line. The paradox, of course, is that we will never know what is unfolding outside our awareness in the body and the brain. Pathways of neurobiology being disrupted and remodeled.

We often say, ‘this is a time released process; results unfold over time.’ It can be a messy process and things may seem to get worse at the level of our awareness, and the benefits often don’t show up at the level of our awareness, especially in the short-term. Given that many of us have spent years immersed in belief systems and coping mechanisms we wish to transform, the process of developing new patterns of behavior requires time and patience. This underscores the importance of integrating within a supportive community, surrounded by a network of witnesses who can serve as reminders of our true essence, especially in moments when we lose sight of it.

Integration: Back to the ordinary

Integration, with the support of your Roots to Thrive community of practice, bridges the gap between memorized knowledge and embodied knowing. The intentional practice within a supportive community, empowers us to integrate insights into our daily lives, shifting our orientation and fostering profound change (Watts & Luoma, 2020). These intentional communities of practice are an important, if not imperative, component of integration.

Your Roots to Thrive community of practice is a vehicle that will help you integrate insights from your session, helping you to practice embodying these new insights as you interact with, and are lovingly witnessed by your peers. By practicing in this way, we gain the empowerment and courage necessary to continue to integrate it into our daily lives. Once we fully embody a new perspective, it changes our orientation to life, including how we view past events, and how we respond to future events.

Lastly, it's crucial to acknowledge that the process of change exists within a liminal space, as explained earlier in this text. This phase symbolizes the transitional space between our previous state and the destination we're moving towards. Venturing beyond our familiar ways of understanding and existing is akin to crossing a threshold into a more fluid and often less predictable realm. This journey can evoke feelings of chaos and instability at times.

To cultivate a sense of stability and agency amid this transition, it's essential to anchor ourselves in relationships that provide unconditional positive regard. These supportive connections act as a secure foundation as we navigate this transformative journey. Additionally, integrating practices, often referred to as rituals, can offer a sense of predictability and control. These practices serve as anchors that remind us we're not alone and that we have the capability to navigate the challenges ahead.

Throughout the Roots to Thrive program, you'll encounter a variety of practices. It's important to tune in to how your body responds and select the practices that genuinely resonate with your needs and preferences. There's no obligation to adopt practices that don't align with your journey, as the goal is to build a toolkit of supportive practices that resonate with your unique BEing.

Appendix C: Intention Setting and Identifying Your Pillars of Strength

Intention Setting and Identifying Pillars of Strength

Intentions are the conscious or subconscious thoughts and desires that shape the reality we experience. They serve as the guiding force behind our actions and decisions, influencing the path we take in life. Even when we're not fully aware of it, our intentions are continuously projected ahead of us, shaping the way our future unfolds.

Setting Intentions

Process > Outcomes

It is important to note that intentions should always be process oriented, as our process is all that is in our control. The 'how' things unfold is influenced by our process, but by and large, we cannot control the outcomes. If you notice that success hinges on an outcome-oriented intention, you might ask yourself questions like, "what quality do I need to acquire or manifest as I move toward a desired outcome?" (i.e. self-compassion, boundaries, more space in my life). The development of the quality then becomes your intention.

Intentions in the psychedelic environment vary, and can range from welcoming an unstructured, open experience - asking the medicine or your inner healing intelligence to guide you to what you need to learn - to wanting to grow a specific quality in your life such as contentment, self-compassion, or joy. You might begin by considering your original intentions coming into this program.

What brought you to this Roots to Thrive journey?

Who do you want to be at the end of the program? Be as specific as you can.

Now consider what qualities you need to lean into or expand to bring about this future self?

Here are some tips for intention creation. They tend to be most helpful if they are:

- personal - inspired by you not prescribed by others
- simple and specific - perhaps even a word or two
- what we want to move toward versus what we want to get rid of
  (e.g. find more calm vs get rid of this anxiety)

If a few come to mind, spend a moment or two trying to listen to your body. This can be simply bringing your awareness to your breath. With this gentle awareness, is there an intention that resonates for you physically?

Write the intention down; you can revisit it after the session or in the days and weeks to come.

Broad themes that you may want to refine for your intention might look like:

- Expanding awareness of your strengths
- Expanding awareness of what is at the root of a blind spot /life challenge
- Exploring an inspiration / desire that's calling you
- Inviting awareness of past trauma in order to begin healing
- Deepening self-compassion
- Deepening compassion and/or forgiving another
- Deepening your ability to be intimate with others
- Reducing attachment to a behaviour that is no longer serving you
- Expanding a sense of gratitude.

In setting your intentions, you can also ask for insights to be gentle, easy, and to inspire change that feel possible for you. We are happy to help you refine your intentions. Please reach out to your facilitators for this conversation.

Pillars of Strength

Choosing to enter into non-ordinary consciousness can be both exciting and anxiety provoking. Together we create a foundation of caring, connection, and trust in our small groups that carries into the medicine sessions. We also encourage you to take some time to identify the **pillars of strength** that you have within yourself that you can immediately reach for when stressed or activated. A pillar of strength is anything internally accessible that provides comfort, courage, or groundedness. It may include:

- a meaningful phrase, mantra, prayer
- tapping into and feeling your connection to certain people and animals in your life
- tapping into and feeling the beings who give you strength such as ancestors, angels, etc.
- a quality that you have, or are becoming: "I am strong", "I am becoming strong", "I am resilient", "I am regaining my power", etc.
- a cultural or spiritual tradition you can bring to mind/body in a moment of distress.
- a breathing practice
- attending to and tracking sensations of the body

Spend some time considering the following:

What can I be reminded of that would help me feel most secure and able to let go when I am fearful and challenged?

You may have an object or photo that symbolizes your pillars of strength. Bring these to your medicine session, or simply bring clarity of them in your mind and heart.

Appendix D: Ketamine-assisted Therapy

Updates by Dr. Pamela Kryskow

About Ketamine

Ketamine is a medication that was discovered in 1956 and patented in 1962. Its first use was as an anesthetic for operations in 1970. The antidepressant effects of ketamine were noted around this time as well, and in the late 1980's we learned of its usefulness for treating both alcohol and opiate use disorder.

Ketamine's psychedelic-like properties were also noted early on, but due to the growing stigma around psychedelic medicines at the time, it was labelled a "dissociative anesthetic" instead. Dissociative in this context refers to the way ketamine causes a sense of separation from one's mind and thoughts, and bodily sensations - even the sense of being in one's body. It is not the same as when people are said to dissociate during, or following, a traumatic event.

In the RTT-KAT program, the primary purpose of Ketamine Assisted Therapy (KAT) is to facilitate insights, resolve trauma and assist you in your healing journey.

Other Treatment Options

Ketamine is not the only treatment option for the challenges you are facing. Counselling, psychotherapy, other medications, ECT, Transcranial magnetic stimulation and lifestyle changes such as meditation, mindfulness, exercise, yoga, tai chi and Qi Gong can all assist individuals in their healing journey.

On and Off-Label Uses

Ketamine is used 'off-label' in this program.

'On-label' means that there have been Phase 1, 2, and 3 clinical trials of a medication at a specific dose, specific frequency and for a specific reason and it can be marketed and sold for that reason. For example, it could be advertised like this: ***You can take 'medication A' for depression at 30mg a day.***

'Off-label' is another allowed use of a medication when it has been found to be useful for other conditions, at other doses, dosing frequencies, or other routes of administration (IV, by mouth, topical, injection, etc.) that were NOT specifically in the clinical trials.

For example, ***'Medication A' can be helpful for pain at 15mg twice a day'***

'Off-label' use is acceptable if there is clinical evidence that it is safe and effective for that other reason and there are other medical professionals that are also in agreement that it is helpful when used in that way.

Ketamine currently has two 'on label' uses:

- as an anesthetic, administered by injection or intravenous (IV).
- for Treatment Resistant Depression, administered intranasally (nasal spray).

There is strong evidence to support using ketamine 'off- label' for depression, chronic pain, anxiety and acute suicidality.

There is growing evidence for the use of ketamine to treat eating disorders, end of life distress, substance use disorder, and PTSD. And for symptoms like grief.

Pain physicians are also using ketamine more and more frequently, instead of opioids, for acute pain treatment - such as after a knee operation.

In our program we use ketamine 'off label' for numerous mental health conditions because there is strong evidence to support its usefulness.

The Science Behind How Ketamine Works (feel free to skip ahead if this isn't of interest to you)

There are four main theories as to how ketamine works as a psychedelic-like substance and may help in your healing journey. They are covered briefly below.

1. Ketamine blocks NMDA Receptors. It causes gabanergic inhibition which gives a surge of glutamate release and mood improves quickly because signalling is restored.

2. Ketamine turns off your anti-reward centre, gives you a break from stress mode, facilitates emotional processing and reduces avoidance states. This is called the lateral habenula theory. The understanding here is that anxiety and depression are manifestations of an overuse injury, just like a cashier who gets carpal tunnel syndrome from constantly scanning food items. The brain can be overused and vulnerable to wear and tear, just like the rest of the body.

3. Ketamine disrupts the connection between the cortex and the limbic system giving you a time out from the ordinary mind, decreasing repetitive thoughts and increasing cognitive flexibility.

4. Finally, ketamine stimulates BDNF leading to new neural connections or neurogenesis, allowing you to make and strengthen new connections in the brain and learn new patterns of thinking and being.

Potential Benefits of Ketamine Over Time

Putting all the theories and clinical experience and research together, ketamine has the potential to:

- facilitate the processing of emotions and memories (good and bad)
- reduce the fear of painful emotions and memories
- reprocess memories
- release unprocessed or stuck emotions, assists with moving through layers of emotions
- help activate your inner healing intelligence
- mood improvement

- increase insight
- decrease anxiety
- release barriers and restrictions
- reduce emotional pain
- reduce physical pain

You may notice changes immediately and more over the coming days, weeks, months.

Potential Side Effects

Common temporary side effects of ketamine include:

Temporary elevation in blood pressure and heart rate
Poor balance (you can move and walk during the session if needed, with our assistance)
Dizziness
Blurred vision
Double vision
Tunnel vision
Slurred speech
Nausea*
Anticipatory anxiety
Agitation
Confusion
Pain at the injection site

*If you are someone that gets nauseated easily or experiences motion sickness, please let the RTT doctor know this during your appointment, so you can discuss the possible use of medication for nausea.

Uncommon temporary effects include:

Vomiting
Insomnia the night following treatment
Headache*
Irritable bladder
Loss of appetite
Muscle tremors

Migraines – some considerations:

Many people experience migraines as a health challenge. For some people participating in KAT migraines get better for others they stay the same. There is no way to know which way it goes.

To lessen migraines, we suggest adding to your daily supplements the following:
- Magnesium bisglycinate 200 – 400mg
- Vitamin B2 200-400mg

- Flushing Niacin 25mg slowly increasing to 500mg over weeks

If on the day of your ketamine session you feel a migraine starting, please take all your abortive medications including triptans, acetaminophen, ibuprofen or other medications that can usually help you. These will not interact negatively with ketamine.

Very rare side effects include:

Allergy to ketamine (treated with a drug called epinephrine)
Laryngospasm (a spasm in your 'voice box')- laryngospasm resolves on its own with respiratory support.

Our medical team is trained and prepared with appropriate medications and medical equipment for any emergencies that may arise.

Is Ketamine Addictive?

In RTT-KAT we use ketamine to assist with healing trauma. When we focus on healing the root of the problem, then misuse is not a concern. Ketamine used in this manner is not addictive. In fact, it can assist with healing the traumas that may have led to substance misuse. Ketamine used in the way we use it in Roots To Thrive can assist with reducing substance misuse.

Health conditions that make taking ketamine unsafe:

If you have any of the following health conditions you must be medically cleared by your primary care doctor and our RTT doctor to be in our program. Unstable conditions must be stabilized and determined safe prior to proceeding.

Unstable heart disease
Untreated or poorly controlled hypertension
Hyperthyroid
Psychosis
Raised intraocular pressure (case by case consideration)
Mania
Pregnancy
Liver disease (case by case consideration)
Renal disease (case by case consideration)
Intoxication on the day of the medicine session

You will be specifically screened for any of these conditions in your doctor's referral, and through the intake process. Please let us know immediately if you have, or develop during our program, one of these conditions, so that we may make a safe or alternative treatment plan.

Medications

Medications of concern include lamotrigine, benzodiazepines (lorazepam, clonazepam, diazepam, etc.), buprenorphine, high dose naltrexone, lithium, rifampin, St. John's Wort, ketoconazole, cimetidine, alcohol and kratom.

If you use any of these substances, it should have been listed on your referral form and reviewed during the intake process. If this was missed, please tell a RTT doctor immediately to make a safe treatment plan.

Consent for Treatment

Prior to your first ketamine session, you will receive instructions and information regarding the consent form that you will be required to sign to receive the ketamine therapy. You may withdraw your consent at any time prior to the injection of the medicine.

Preparing for your ketamine session

Days leading up to the session:

- If possible, have relaxing days in the days and week prior to your session.
- Get a good night's sleep prior to the session.
- It is helpful to prepare for physical and emotional challenges that may arise during the KAT session.
    - 4/7/8 breath
    - The daily energy routine
    - EFT/tapping
    - Butterfly Hug
    - RAIN
- Pre-session excitement or anxiety is normal and expected - this is an opportunity to try the strategies above.
- Make sure you arrange a ride home - you CANNOT drive after your session.

On the day of your session:

- Have minimal/light amount of food 4-6 hours prior
- Clear liquids ok to drink up to 2 hours prior
- Avoid grapefruit juice & caffeine
- Avoid stimulants (if possible)
- Wear comfortable clothing & bring layers

What to bring to your ketamine session

We provide mats, weighted blankets, eye coverings, and headphones for your use during the session.

Please bring your own pillow, socks or slippers, water, a sweater, and healthy snacks.

Consider also bringing additional blankets, items of support or significance such as photos, and a journal and pen. If you anticipate feeling uncomfortable lying down, please bring any extra pillows/bolsters that may help, or anything else that will make you feel more comfortable. Ketamine can give you the sensation of feeling cold so you may want a few extra layers.

Ketamine Session Information

Your three ketamine sessions will be with your small group. You will all experience the medicine together at the same time. The session will be led by your small group facilitators and will be supported by various members of our team. You will have met everyone in the room on-screen during our Community of Practice meetings.

On the day of the ketamine session, you will arrive at Snuneymuxw Traditional Medicines Wellness Centre for check-in. This involves having your blood pressure and heart rate checked. Your blood pressure needs to be in the normal range to have ketamine therapy. If you arrive and it is outside our treatment range, we will help you utilize various relaxation techniques to help you lower your blood pressure. If it is still high, we have the option of offering you either clonidine or captopril - two medications that are used to lower blood pressure and will not interfere with ketamine. If we cannot get your blood pressure in range, we will have to postpone your ketamine session.

You will see a RTT doctor for any final questions and to confirm your ketamine dose.

You then will settle into the ceremony room with your group. It will be a large room, with mats on the floor for each participant, and floor cushions for your facilitators and other team members.

Medication Dose and Administration

The amount of ketamine you receive is partly based on your weight and partly based on your intention for your medicine session. You will discuss your dose prior to your session with a RTT doctor during an online appointment. There, you will have an opportunity to ask any remaining questions, and together decide on a dose based on your priorities, intentions, previous experience, and comfort level. On the day of your session at the health centre you will have another opportunity to meet with your RTT doctor and confirm the dose.

*Please know that **all** doses are the right dose. No matter what you decide upon, the medicine will aid you in your healing journey.*

You will receive the ketamine in an intramuscular injection of ketamine in your gluteal muscle (buttock). When it is time to administer the medication, we will ask you to lie on your side, so a doctor or nurse can give the injection into your buttock. This will be done discreetly. You also have the option of a deltoid/shoulder injection - please discuss this with your RTT doctor in your appointment if you are interested in this option.

The injection will take about 20 seconds. You will then lay down and get comfortable with your eye shades, headphones and your blanket on. Music will be playing in the room, and in your headphones. A unique playlist is curated for each ketamine session. Within 2-4 minutes you will begin to feel the effects

of the ketamine. Usually, the first sensation you feel is relaxation. Often, the first sensation you feel is relaxation. You will feel the medicine most strongly 40-90 minutes after your injection.

You may experience an out- of-body sensation, formlessness, visual hallucinations, a sense of timelessness, a disruption or dissolution of negative feelings, relaxation from ordinary concerns, or a break from the constant stream of thoughts in your head. You may have visions, relive memories, you may experience darkness. All experiences are normal.

You may hear crying or laughter in the room. You may hear others speaking. There might be movement and sounds around you as we help people with their physical and emotional needs. Please be assured that the team is there for you and others, and allow yourself to stay with your own journey. You may be accustomed to caring for others in your day-to-day life, and it can be a powerful experience to release your role as a caregiver, and to just BE; trusting that others can navigate their own experience, and trusting the team will support whatever arises. Allow yourself to let go of any responsibility for others in the room. You don't have to DO anything; you can BE in your experience. This is your time.

The overall ketamine session is 4-5 hours; however, you will still feel effects of the ketamine for several hours after you leave. This is why you cannot drive following your session, operate equipment, or do anything that could be compromised by your being tired and/or uncoordinated. You must have someone pick you up from the health centre as you will be unsafe to drive yourself home. We can assist you in calling a taxi if needed.

Your Ketamine Experience

Everybody's experience is different. Whatever your experience is, it is what you need to take you to the next stages of YOUR healing journey.

The ketamine sessions are an opportunity to trust and invest in your own deep processes of growth and healing. You may be worried that the ketamine 'won't work'. This is a common concern. It may not look like what you expect, but trust that you will get the learnings or experience that you need. Trust your inner healing intelligence knows what you need to heal (see pages 52-53 for more about inner healing intelligence).

Think of hiking to a lake 5km away…you don't take one step and end up at the lake. Instead, the path winds, there are hills and valleys, and all along the way there are interesting things to see. Each step along the path is part of the journey. Ketamine will help your inner intelligence prioritize what needs to be attended to first; what steps are next along the path.

Some of you may have a very 'big' experience - your path might be filled with visuals, memories, profound insights. For others it may feel subtle; a quiet stroll.

If you find yourself in a challenging situation, first remember you are physically safe. Then we invite you to be curious about the challenging situation, what it can teach you, ask it questions, what you need to do to move through it and resolve it. If you need support to trust and let go, ask for help from the team or return to your pillars of strength.

As you emerge from your experience please remember *all* experiences are valuable and exactly what each person needs. Try to avoid comparing your experience to others.

We spend time setting intentions for the medicine session, which is reviewed below. Intentions can be helpful, but it is important to let go of the '*outcome'* of your ketamine session. Practice letting go of expectations, trusting that whatever arises is exactly right for you. In this place of surrender, you can *receive* what is offered to you.

Integration

The 36 to 48 hours following a medicine session experience is an important opportunity to engage in self-care, practice new patterns and begin to establish new neural pathways. You may hear us speaking of this time period as one of heightened **neuroplasticity**. Neuroplasticity is simply the brain's ability to modify, change, and adapt both structure and function.

Prepare a space at home (or wherever you are lodging) to gently land and allow for rest. We strongly encourage you to protect the following day with rest and gentleness. Previous participants have stated that having two days after their session off work was helpful. It may become important for you to flex your schedule and expectations of yourself to accommodate and care for yourself as new awareness and needs arise.

Integration happens when we embody new insights, which then leads to new behaviours. It happens when we simply settle into and practice new ways of being in the world, which can be further reinforced when compassionately witnessed by others. To encourage such settling, it's beneficial to continue going 'in and down' into your body, as often as possible. Listen to your body and engage in activities that feel inspiring.

The following are some suggestions for the days following your session:

- Try to create a calm & relaxing spot in your home
- Limit outside distractions as much as possible and consider taking a break from social media for a few days
- Eat nourishing, healthy foods
- Drink a lot of water or other refreshing fluids
- Journal
- Engage creatively - paint, draw, sculpt, make music, dance
- Light exercise - walking, stretching
- Daydream
- Meditate - if this is new to you, there are numerous guided meditations on YouTube.
- Magnesium supplements may be helpful for relaxation and sleep
- If you feel 'groggy' in the days following your session, a CBD tincture may help

To assist in integrating insights from the medicine sessions, we offer online integration sessions in the two days following your session. Days and times of Zoom integration sessions will be sent to you in an email from our team. These sessions are optional and we strongly encourage you to attend one or all of

them, even just to listen. Integration sessions are an opportunity to talk about your experience, be witnessed by others, and be a witness for the experience of others.

Once insights begin to drop more solidly into your being (finding the words that articulate your felt sense), try writing them down. This acts as a bridge between your insights, your bodily signals, and how they are then expressed in the world. To imbed these awarenesses further, discuss your discoveries with your buddy, a trusted friend, family member, or therapist. Sharing insights with others provides an opportunity for witnessing, which is also a key component of embodying more **congruence** (alignment between your real and ideal self) and **sense of coherence** (sense of meaning, understanding, and confidence to navigate challenges).

Appendix E: Psilocybin-assisted Therapy

Updates by Dr. Pamela Kryskow

Information About Psilocybin

Psilocybin is known as one of the ‘classic’ psychedelics and is derived from mushrooms of the genus Psilocybe (and others) and has a long history across the globe for cultural healing and spiritual practices. While it has been used for spiritual and medicinal purposes for many years in many cultures, here in Canada, it has not yet been approved for therapeutic purposes by Health Canada.

How Psilocybin Works

Psilocybin is a partial serotonin agonist that binds receptors in the brain that are responsible for mood and anxiety disorders, including the prefrontal cortex. While the molecular mechanisms are unclear, the resulting effects produce what is commonly known as a mystical-like non-ordinary effect which in turn facilitates antidepressant and anti-anxiety effects. Psilocybin can also bring about different states of conscious, including what might be described as spiritual or mystical awareness. These effects can support new insights; greater access to and / or a reorientation to memories and thought patterns; a different sense of self and reality. In a safe environment with therapeutic support, working with psilocybin can help individuals heal and transform, finding greater peace and compassion. Experiences tend to produce a positive change in outlook and character and relieve symptoms of depression, PTSD, anxiety, and other difficult states of mind and heart.

Essentially, the experience is a time-out from our ordinary state of mind, this period being of varying duration, usually four to eight hours. Characteristically, there is a relaxation from ordinary concerns while maintaining conscious awareness of the flow of mind under the influence of Psilocybin. This tends to lead to a disruption of negative feelings and obsessional preoccupations. This relief and the exploration and experience of other possible states of consciousness are uniquely impactful. The treatment team acts as facilitators of the experience, preparing you for it and facilitating your process.

How a PaT Session Works

During your PaT treatment sessions, you and your group will be accompanied by a medical clinician and a PaT trained therapeutic sitter. Once Psilocybin is taken, most effects will last between four to eight hours. Unlike other forms of therapy, PaT is largely an inner journey. You will not be talking through your experience while the medicine effects are unfolding. A clinician and therapist will be present and available to provide you with support at any time. There will be an opportunity to share and process your experience once the effects of the medicine have worn off and in the days following with the group & the facilitators. To support an inward focus and minimize external distraction, instrumental music will be played, and we encourage the use of eye shades.

Transformation can come while preparing for your psychedelic experience (see Appendix F for intention setting), during your experience, and following your experience during integration. We provide some thoughts and ideas about each of these phases. Hold them gently and run them through your own discernment process. Keep what works and set down what doesn’t.

Preparation

Minimize physical distractions as much as possible by ensuring your body's needs and comfort preferences are addressed leading up to your session.

- eat lightly before the session so your body can absorb the Psilocybin more easily.
- Take your regular medication unless you have been advised by one of the doctors to with-hold a particular medication. If you have specific medications you feel you will need, please bring them.
- Get a good night's sleep prior to the session.
- Prior to your session, it's helpful to prepare for physical and emotional challenges that may arise during the PAT session. The body may experience stress, this is an excellent opportunity to practice self-regulation strategies. The body may experience strong emotional sensations, this is a fantastic thing and an opportunity to allow an old wound to heal. *Because psilocybin provides non-attachment of emotions (a concept discussed in more detail in Chapter 9) , you have an opportunity to feel intense emotions without feeling so threatened by them.*
- During the session, you will find a position that allows you to rest completely without the distraction of physical effort (lying down is ideal).
- Make sure you arrange a ride home.

What To Bring

We provide mats, weighted blankets, eye shades and headphones. Please bring a water bottle, pillow, socks/slippers, and a snack for after the session (like cut up fruit, nuts, crackers, etc). Consider also bringing another blanket, a sweater, ice packs or heating pads if you use them in your standard care routine, a journal and pen, photographs and other touch stones you may want with you. If you have a favorite pair of eye shades bring those.

Some people bring a laundry basket (or whatever works for you) with all the things they need, but please remember, the less stuff in the room, the easier it is for team members to assist you.

Your Psilocybin Experience

In the psilocybin session, we are now invited to slow down, trust, and invest in our own deep processes of growth and healing. Let go as much as you can into whatever experience you are given. Although we spent time setting intentions for the journey, at this time it is important to let go of the **outcome.** As you go into your experience, notice if you find yourself grasping for your intention. Practice letting go of expectation, trusting that whatever arises is exactly right for you. In this place of surrender, you can **receive** what is offered to you.

You can break through anything that feels challenging by being curious and compassionate, which is the antidote to resisting the experience. You might even ask the presenting challenge a question, which can promote non-attachment and compassion for what is arising. If you need support to trust and let go, ask for help from the team or by returning to your pillars of strength.

We encourage you to go ‘down and in’ rather than ‘up and out.’ Avoid the urge to talk or connect with others about your experience while you are still in it. The deeper and more silent your experience, the more meaningful and transformative it can be.

Integration

Around 6 hours after you have taken the psilocybin, you will gradually come back into this time and place, bringing with you everything you experienced and learned. We will have a transition circle for sharing while we are still together.

You might find it difficult to speak about your experience during the closing portion of our session. You do not need to speak if you are not ready to do so. You might not yet have the clarity or words to describe your experience, or you may be impacted emotionally and not feel ready to talk. Or you might be excited and ready to share. Give yourself time and space to navigate this period gently and welcome any silence, or sharing, into which you are drawn. Trust yourself to know what you need during this time.

The 36 to 48 hours following a medicine session experience is an important opportunity to engage in self-care, practice new patterns and begin to establish new neural pathways. You may hear us speaking of this time period as one of heightened **neuroplasticity**. Neuroplasticity is simply the brain's ability to modify, change, and adapt both structure and function.

Prepare a space at home (or wherever you are lodging) to gently land and allow for rest for. We strongly encourage you to protect the following day with rest and gentleness. Previous participants have stated that having two days after their session off work was helpful. It may become important for you to flex your schedule and expectations of yourself to accommodate and care for yourself as new awareness and needs arise.

Integration happens when we embody new insights, which then leads to new behaviours. It happens when we simply settle into and practice new ways of being in the world, which can be further reinforced when compassionately witnessed by others. To encourage such settling, it’s beneficial to continue going ‘in and down’ into your body, as often as possible. Listen to your body and engage in activities that feel inspiring, rather than obligatory.

The following are some suggestions for the days following your session:

- Try to create a calm & relaxing spot in your home
- Limit outside distractions as much as possible and consider taking a break from social media
- Eat nourishing, healthy food
- Drink a lot of water or other refreshing fluids
- Journal
- Engage creatively - paint, draw, make music
- Light exercise - walking, stretching
- Daydream
- Listen to the playlist from your session, which will be provided to you following

- Meditation - if this is new to you, there are ample guided meditations via YouTube

Once insights begin to drop more solidly into your being, try writing them down. This acts as a bridge between your insights, your bodily signals, and how they are then expressed in the world. To imbed these awarenesses further, discuss your discoveries with your buddy, a trusted friend, family member, or therapist. Sharing insights with others provides an opportunity for witnessing, which is also a key component of embodying more congruence (alignment between your real and ideal self) and sense of coherence (sense of meaning, understanding, and confidence to navigate challenges).

Appendix F: Therapeutic Touch and Energy Medicine During Your Medicine Session

Therapeutic Touch and Energy Medicine Support

Two videos will be made available to all KAT/PAT participants to explain what therapeutic touch and energy medicine supports may be possible during the medicine session.

During the medicine sessions, energy practitioners and your facilitators are available to offer support.

While some participants lie still on the mat; headphones on and eyes covered for the duration of the session, others report benefit from therapeutic touch.

To connect with your inner healing intelligence, it is best to remain internally focussed; go ‘in and down’ you may hear us share from Elder Duncan Grady. We are careful to touch you only when deemed necessary, and only with your permission. Consent for energy work and touch are reviewed prior to administration of medicine, and communicated clearly to everyone who will be in the room. If there are immediate concerns for your physical safety, we may need to touch you to keep you, or others around you safe.

During the session, there may be an opportunity to create corrective and healing experiences in your body, helping your body remember it is not alone, that people are with you, keeping you safe. This physical care can help rewrite old stories of separation and isolation.

Asking for physical support can expand your window of tolerance, affirming that you are worthy of support. To create this sense of security in the body, you may ask for physical touch during your session.

Alternatively, it may be empowering to set a boundary, requesting no touch at all. That may be what you need to instill a feeling of security in the body. Know that we will honour your requests and that you can ask for physical support at any time during the session. Touch might include holding your hand, touching your shoulder or placing a hand on your forehead for calming assurance. Your ankles might be held to help you to settle.

The energy workers on our team might employ techniques including holding acupressure or neuro-lymphatic points on the head, hands or feet that are affiliated with emotions. This can help release suppressed emotions in the body. Holding the back or front of the chest can support the heart’s energy field. Beyond physical touch, energy work can be done in the field above the body. The same consent applies to any energy work.

Appendix G: Music as Medicine

By Phillip Dames

We all have stuck energies in our body. The medicine will show you where it is. The music will meet you there and assist in moving the energy. It's a tool. Meet the music at the energy, and your inner healing intelligence will take it from there.

What is music?

Music is medicine
Music is tangible
Music is a touch point in time
Music pulls you into active listening
Music is art
Music is connection
Music is vulnerable
Music is integration
Music is meant to be shared
Music is love
Music moves energy

Combined with intention and a secure set and setting, music helps metabolize strong emotions and sensations. It promotes heartfulness and with intention and regulation practices, it can also expand your window of tolerance. This can be especially helpful for those who continue to be immersed in trauma laden environments (Bensimon, 2020) and those working with post-traumatic stress (Beck et al., 2021; Macfarlane, Masthoff, & Hakvoort, 2019).

By making space for the various layers and sensations that music elicits in the body, we also learn to make space for the various layers and sensations that emotions elicit in the body. Music promotes active listening and pulls us into the present. In this presence we are more curious, increasing awareness within and releasing outside distractions. Sometimes we can even disengage from our thinking mind and our perception of time changes. Rather than trying to figure out what is coming next in a track or what track is coming next, we rest into the moment, allowing the music to take us there, meeting whatever we are meant to meet.

In these ways, music can be an integral part of one's healing, regardless of where we are on our journey. It creates a touch point in time that we can return to through our auditory senses. By using different types of music, we can create more precise touch points in time. Using tracks that create additional textures makes the music more tactile in nature with more layers to tune into. Adding these layers adds an additional sensory element. As a result, it magnifies the ability to reach back to that track when we need to tune back in. Our body has an incredible ability to recall these moments, even if our brain does not.

Types of Music and Set (intentions) and Setting (environment and tools):

- Go inside: close your eyes and ears to the outer world so you can focus on the inner world.
- Choose wisely: Find music that you like and that moves you – fluffing up sensations within, while simultaneously opening your heart space. Ideally you will choose music without words so you can listen for your inner voice. If you are listening to lyrics, make sure they align with your own values and intentions.
- Resonance: similar to music instruments, we all attune to one another. The resonance you feel with certain music has the capacity to pass onto others and form a connective pathway. As you create the set and setting for a musical journey, choose music you enjoy and that your body truly resonates with.
- Finding tracks that have a structure of tension and release allows you to move through different emotional processes. Having a higher level of Timbre helps in this process.
- Ambient music can be a good place to start. It often can provide a nice blend of overtone based music that is rich in timbre and can create different textures and spaciousness. Mixing in natural sounds like birds, rain or running water can increase spaciousness.
- Utilize different instruments and textures within the music to activate different parts of your body. Strong beats send vibrations into your centre, reminding you of the pilot light within. The sound of a bow on a stringed bass may be resonant in your heart or a violin may stimulate your mind.
- Working with Strong emotions/sensations: If strong sensations come up, this is an excellent time to use your regulation practices (softening tension with breath, EFT/tapping, stretching, physical activity, the butterfly hug, dance, sigh etc.). Also notice what song is playing, approaching it with curiosity. Think of each song as a scene in a movie. Ultimately each scene ends, as will each song.
- Use RAIN (see Chapter 4). RAIN is an excellent reminder to recognize, allow, investigate, and nurture what comes up for tending and releasing.
- Get outside and move! Combining music with nature and movement can be a profound mixture. Physical movement allows for energetic release, which allows space to come in. In that space, something like music can aide a unique process of unfolding.
- Notice specific tracks to mark a space in time during your journey. Come back to the tracks that move you: your mind may not remember, but your body will. If strong emotions arise during the track, take note and come back to it.
- Start gentle with music that provides more inner comfort than felt chaos. As you practice, you will be more able to allow strong sensations to come and go, trusting you have the inner and outer resources (security and regulating tools) to navigate whatever arises.
- Don't be afraid to play the music loud as it allows your body to feel the music more, becoming one with it.

Ambient Playlists for starting points:

Ambient Soundscapes (less beats, more chill)

Soundscapes for Gaming (more upbeat, including some words)

Playlists used in Ketamine Assisted Therapy Sessions

References

Aldao, A., Nolen-Hoeksema, S., & Schweizer, S. (2010). Emotion regulation strategies across psychopathology: A meta-analysis. *Clinical Psychology Review, 30*, 217–237. doi: 10.1016/j.cpr.2009.11.004

Anand, H. (2014). Effect of meditation ('OM' chanting) on alpha EEG and galvanic skin response: Measurement of an altered state of consciousness. Indian *Journal of Positive Psychology, 5*(3), 255.

Antonovsky, A. (1979). *Health, Stress and Coping*. San Francisco: Jossey-Bass.

Antonovsky, A. (1987). *Unraveling the Mystery of Health: How people manage stress and stay well*. San Francisco: Jossey-Bass.

Artigas, L., Jarero, I., Mauer, M., López Cano, T., & Alcalá, N. (2000, September). EMDR and Traumatic Stress after Natural Disasters: Integrative Treatment Protocol and the Butterfly Hug. Poster presented at the EMDRIA Conference, Toronto, Ontario, Canada.

Aspy, D. J., & Proeve, M. (2017). Mindfulness and loving-kindness meditation. *Psychological Reports, 120*(1), 102.

Bartlett, C., Marshall, M., Marshall, A. (2012). Two-eyed seeing and other lessons learned within a co-learning journey of bringing together indigenous and mainstream knowledges and ways of knowing. Journal of Environmental Studies and Sciences, 2, 331–340.

Bartlett, M.Y., DeSteno, D. (2006). Gratitude and prosocial behavior**.** *Psychology Science, 17*(4), 319-325. doi: https://doi-org.ezproxy.viu.ca/10.1111/j.1467-9280.2006.01705.x

Beck, B. D., Meyer, S. L., Simonsen, E., Søgaard, U., Petersen, I., Arnfred, S. M. H., Tellier, T., & Moe, T. (2021). Music therapy was noninferior to verbal standard treatment of traumatized refugees in mental health care: Results from a randomized clinical trial. *European Journal of Psychotraumatology, 12*(1)https://doi.org/10.1080/20008198.2021.1930960

Bensimon, M. (2020). Perceptions of music therapists regarding their work with children living under continuous war threat: Experiential reframing of trauma through songs. *Nordic Journal of Music Therapy, 29*(4), 300-316. https://doi.org/10.1080/08098131.2019.1703210

Bluth, K., Campo, R. A., Futch, W. S., & Gaylord, S. A. (2017). Age and gender differences in the associations of self-compassion and emotional well-being in a large adolescent sample. *Journal of Youth and Adolescence,* 46(4), 840-853. doi:10.1007/s10964-016-0567-2

Bly, R. *A Little Book on the Human Shadow*. New York: Harper & Row, 1989.

Bonini, L., Rotunno, C., Arcuri, E., & Gallese, V. (2022). Mirror neurons 30 years later: Implications and applications. *Trends in Cognitive Sciences, 26*(9), 767–781. https://doi.org/10.1016/j.tics.2022.06.003

Bowlby, J. (2012). *A secure base*. Hoboken: Taylor and Francis.

Brach, T. (2019). *Radical compassion: Learning to Love Yourself and Your World.* New York, Viking.

Brown, D. P., & Elliott, D. S. (2016). Attachment disturbances in adults: Treatment for comprehensive repair. W W Norton & Co.

Church, D., Stapleton, P., Vasudevan, A., & O'Keefe, T. (2022). Clinical EFT as an evidence-based practice for the treatment of psychological and physiological conditions: A systematic review. Frontiers in Psychology, 13, 951451- 9 51451. https://doi.org/10.3389/fpsyg.2022.951451

Corey, T. P., Shoup-Knox, M. L., Gordis, E. B., & Gallup, J., Gordon G. (2012). Changes in physiology before, during, and after yawning. *Frontiers in Evolutionary Neuroscience, 3*, 7. doi:10.3389/fnevo.2011.00007

Cruceanu, V.D. & Rotarescu, V.S. (2013). Alpha Brainwave Entrainment as Cognitive Performance Activator. Cognition, Brain, Behavior, 17(3), 249.

Dames, S. S. (2018). A Study of the Interplay between New Graduate Life Experience, Context, and the Experience of Stress in the Workplace: Exploring Factors towards Self-Actualizing as a Novice Nurse (Unpublished Doctoral Thesis). University of Calgary, Calgary, Ab.

Dames, S. (2022). Root Strength: A Health and Care Professionals' Guide to Minimizing Stress and Maximizing Thriving. Elsevier. Textbook. ISBN: 9780323778695

De Silva, P. (2017). Emotions and the body in Buddhist contemplative practice and mindfulness-based therapy: Pathways of somatic intelligence. Cham: Palgrave Macmillan US.

Fitts, P.M., Posner, M.,I. (1967). *Human Performance*. Brooks/Cole Pub. Co; Belmont, CA.

Gollan, J. K., Hoxha, D., Hunnicutt-Ferguson, K., Norris, C. J., Rosebrock, L., Sankin, L., & Cacioppo, J. (2016). Twice the negativity bias and half the positivity offset: Evaluative responses to emotional information in depression. *Journal of Behavior Therapy and Experimental Psychiatry, 52*, 166170. doi:10.1016/j.jbtep.2015.09.005

Gunnell, K. E., Mosewich, A. D., McEwen, C. E., Eklund, R. C., & Crocker, P. R. E. (2017). Don't be so hard on yourself! Changes in self-compassion during the first year of university are associated with changes in well being. *Personality and Individual Differences, 107*(2017), 43-48.

Hammarlund, R. A., Crapanzano, K. A., Luce, L., Mulligan, L. A., & Ward, K. M. (2018). Review of the effects of self-stigma and perceived social stigma on the treatment-seeking decisions of individuals with drug- and alcohol-use disorders. *Substance Abuse and Rehabilitation, 9*, 115-136. doi:10.2147/sar.s183256

Harmon-Jones, E., & Peterson, C. K. (2009). Supine body position reduces neural response to anger evocation. Psychological Science, 20(10), 1209-1210. doi:10.1111/j.1467-9280.2009.02416.x

Homan, K. J., & Sirois, F. M. (2017). Self-compassion and physical health: Exploring the roles of perceived stress and health-promoting behaviors. *Health Psychology Open, 4*(2), 2055102917729542. doi:10.1177/2055102917729542

Hutcherson, C. A., Seppala, E. M., & Gross, J. J. (2008). Loving-kindness meditation increases social connectedness. *Emotion, 8*(5), 720-724. doi:10.1037/a0013237

Hwang, S., Kim, G., Yang, J., & Yang, E. (2016). The moderating effects of age on the relationships of self-compassion, self-esteem, and mental health: Self-compassion and age. *Japanese Psychological Research*, 58(2), 194-205. doi:10.1111/jpr.12109

Ionescu, D. F., Felicione, J. M., Gosai, A., Cusin, C., Shin, P., Shapero, B. G., & Deckersbach, T. (2018). Ketamine-associated brain changes: A review of the neuroimaging literature. *Harvard Review of Psychiatry, 26*(6), 320-339. doi:10.1097/HRP.0000000000000179

Jakobsen, J. C., Gluud, C., & Kirsch, I. (2020). Should antidepressants be used for major depressive disorder?. *BMJ evidence-based medicine, 25*(4), 130. https://doi.org/10.1136/bmjebm-2019-111238

Jellal, R. & Barks, C. (1997). *The essential Rumi*.

Jellesma, F. C., Rieffe, C., Meerum Terwogt, M., & Westenberg, P. M. (2011). Children's sense of coherence and trait emotional intelligence: A longitudinal study exploring the development of somatic complaints. England: Routledge. doi:10.1080/08870440903411021

Jung, C.G. (1970). *Mysterium Coniunctionis*, Collected Works of C.G. Jung, Volume 14, Princeton, N.J.: Princeton University Press. ISBN 978-0-691-09766-4

Kearney, D. J., Malte, C. A., McManus, C., Martinez, M. E., Felleman, B., & Simpson, T. L. (2013). Loving-Kindness meditation for posttraumatic stress disorder: A pilot study. *Journal of Traumatic Stress, 26*(4), 426-434. doi:10.1002/jts.21832

Kelly, J. (2018). Forgiveness: A key resiliency builder. *Clinical Orthopaedics and Related Research, 476*(2), 203-204. doi:10.1007/s11999.0000000000000024

Kelly, A. C., Vimalakanthan, K., & Miller, K. E. (2014). Self-compassion moderates the relationship between body mass index and both eating disorder pathology and body image flexibility. *Body Image, 11*(4), 446-453. doi:10.1016/j.bodyim.2014.07.005

Lally, P, van Jaarsveld, C.H.M, Potts, H.H.W, Wardle, J. (2010). How are habits formed: modelling habit formation in the real world. European Journal of Social Psychology. 2010;40:998–1009.

Lee, L., James, P., Zevon, E., Kim, E., Trudel-Fitzgerald, C., Spiro, A., Grodstein, F., Kubzansky, L. (2019). Optimism is associated with exceptional longevity in 2 epidemiologic cohorts of men and women. Proceedings of the National Academy of Sciences. doi:10.1073/pnas.1900712116

Lee, Y., Kim, H., Cheon, E., Kim, K., Choi, J., Kim, J., . . . Koo, B. (2019). The analysis of electroencephalography changes before and after a single neurofeedback Alpha/Theta training session in university students. *Applied Psychophysiology and Biofeedback, 44*(3), 173-184. doi:10.1007/s10484-019-09432-4

León-Pérez, G., Martín-Albo, J., Notario-Pacheco, B., Gallego-Beuter, J. L., & Pérez-Yus, M. C. (2012). Spanish adaptation of the perceived stress scale (PSS-14): psychometric properties. Retrieved from [provide URL if available]

Li, X., Ma, R., Pang, L., Lv, W., Xie, Y., Chen, Y., . . . Zhang, X. (2017). Delta coherence in resting-state EEG predicts the reduction in cigarette craving after hypnotic aversion suggestions. *Scientific Reports, 7*(1), 2430-8. doi:10.1038/s41598-017-01373-4

Liu, Y. Z., Wang, Y. X., & Jiang, C. L. (2017). Inflammation: The Common Pathway of Stress-Related Diseases. Frontiers in human neuroscience, 11, 316. https://doi.org/10.3389/fnhum.2017.00316

Lund I., Ge Y., Yu L. C., Uvnäs-Moberg K., Wang J., Yu C., et al. (2002). Repeated massage-like stimulation induces long-term effects on nociception: contribution of oxytocinergic mechanisms. Eur. J. Neurosci. 16, 330–338. 10.1046/j.1460-9568.2002.02087.

Ly, C., Greb, A. C., Cameron, L. P., Wong, J. M., Barragan, E. V., Wilson, P. C., ... & Olson, D. E. (2018). Psychedelics promote

structural and functional neural plasticity. Cell Reports, 23(11), 3170-3182.

MacDonald, D. A., & Friedman, H. L. (2020). Growing up and waking up: A conversation with Ken Wilber about leaving transpersonal to form integral psychology. Journal of Humanistic Psychology, , 2216782090228. doi:10.1177/0022167820902287

McCraty, R., Atkinson, M., Tiller, W. A., Rein, G., & Watkins, A. D. (1995). The Effects of Emotions on Short-Term Power Spectrum Analysis of Heart Rate Variability. Journal of the American College of Cardiology, 78(14), 1091-1093.

McCraty, Rollin. (2016). Science of the Heart, Volume 2 Exploring the Role of the Heart in Human Performance An Overview of Research Conducted by the HeartMath Institute. 10.13140/RG.2.1.3873.5128.

Multidisciplinary Association for Psychedelic Studies (MAPS). (2015). A Manual for MDMA-Assisted Psycho-therapy in the Treatment of Posttraumatic Stress Disorder. Version 7.

Maslow, A. H. (1943). A theory of human motivation. *Psychological Review, 50*(4), 370-396. doi:10.1037/h0054346

Maslow, A. H. (1968). *Toward a psychology of being*. New York: D. Van Nostrand Company.

Maslow, A. H. (1971). *The farther reaches of human nature*. New York: Viking.

Macfarlane, C., Masthoff, E., & Hakvoort, L. (2019). Short-term music therapy attention and arousal regulation treatment (SMAART) for prisoners with posttraumatic stress disorder: A feasibility study. *Journal of Forensic Psychology Research and Practice, 19*(5), 376-392. https://doi.org/10.1080/24732850.2019.1670023

Morina, N., Schnyder, U., Schick, M., Nickerson, A., & Bryant, R. A. (2016). Attachment style and interpersonal trauma in refugees. *Australian & New Zealand Journal of Psychiatry, 50*(12), 1161-1168. doi:10.1177/0004867416631432

Nummenmaa, L., Hari, R., Hietanen, J. K., & Glerean, E. (2018). Maps of subjective feelings. Proceedings of the National Academy of Sciences of the United States of America, 115(37), 9198–9203. https://doi.org/10.1073/pnas.1807390115

Neff, K., Germer, C. (2018). The Mindful Self-Compassion Workbook: A proven way to accept yourself, build inner strength, and thrive. New York, London: The Guildford Press.

Neff, K. (2018). *Self-Compassion.* Center for Mindful Self-Compassion. Retrieved from http://self-compassion.org/the-three-elements-of-self-compassion-2/

Nepo, M. (1985). Unlearning Back to God: Essays on Inwardness, 1985-2005. Khanqahi Nimatullahi Publications.

Newson, J. J., & Thiagarajan, T. C. (2019). EEG frequency bands in psychiatric disorders: A review of resting state studies. Frontiers in Human *Neuroscience, 12*, 521. doi:10.3389/fnhum.2018.00521

Niebuhr, R. (1892-1971). Serenity Prayer (unpublished work).

Nummenmaa, L., Hari, R., Hietanen, J. K., & Glerean, E. (2018). Maps of subjective feelings. *Proceedings of the National Academy of Sciences of the United States of America, 115*(37), 9198–9203. https://doi.org/10.1073/pnas.1807390115

Oschman, James L, 2000, Energy Medicine, The Scientific Basis, Philadelphia,USA, p73-84.

Park B., J, Tsunetsugu Y, Kasetani T, Hirano H, Kagawa T, Sato M. (2007). Physiological effects of Shinrin-yoku (taking in the atmosphere of the forest) using salivary cortisol and cerebral activity as indicators. Journal of Physiological Anthropology, 26, 123–128.

Paris, J. (2015). The intelligent clinician's guide to DSM-5. Oxford, [England]; New York, New York;: Oxford University Press.

Porges, S. W. (2011). The polyvagal theory: Neurophysiological foundations of emotions, attachment, communication, and self-regulation (Norton series on inter- personal neurobiology). New York, NY: Norton.

Pratt, L. A., Brody, D.J., Gu, Q. (2017). Antidepressant use among persons aged 12 and over: United States, 2011–2014. NCHS data brief, no 283. Hyattsville,
MD: National Center for Health Statistics.

Rebadomia, F. M. L., Amparo, Jeremiah Seag Miguel G, Reyes, J. P., Cobar, A. G. C., Camarador, R. A., Ateneo de Manila University, Basic Education Unit, Quezon City, Philippines, & Polytechnic University of the Philippines, College of Human Kinetics, Manila, Philippines. (2019). Effect of music with brainwave synchronizer on the performance of collegiate throwing athletes. Sport Mont, 17(2), 17-22. doi:10.26773/smj.190603

Reinhold, M., Bürkner, P., & Holling, H. (2018). Effects of expressive writing on depressive symptoms—A meta-analysis. *Clinical Psychology:* Science and Practice, 25(1), e12224-n/a. doi:10.1111/cpsp.12224

Rogers, C. (1959). A theory of therapy, personality and interpersonal relationships as developed in the client-centered framework. In (ed.) S. Koch, Psychology: A study of a science. Social Context (Vol.

3, pp. 184-256). New York: McGraw Hill.

Rohr, Richard. (1999). Everything Belongs: The Gift of Contemplative Prayer. The Crossroad Publishing Company, pp. 155-156

Sabo Mordechay, D., Nir, B., & Eviatar, Z. (2019). Expressive writing - who is it good for? individual differences in the improvement of mental health resulting from expressive writing. *Complementary Therapies in Clinical Practice, 37, 115*-121. doi:10.1016/j.ctcp.2019.101064

Savage, B. M., Lujan, H. L., Thipparthi, R. R., & DiCarlo, S. E. (2017). Humor, laughter, learning, and health! A brief review. Advances in Physiology Education, 41(3), 341–347. https://doi.org/10.1152/advan.00008.2017

Seok, J., & Kim, J. U. (2024). The effectiveness of emotional freedom techniques for depressive symptoms: A meta-analysis. Journal of Clinical Medicine, 13(21), 6481. https://doi.org/10.3390/jcm13216481

Siegel, Dan. (1999). The Developing Mind: toward a Neurobiology of Interpersonal Experience. New York: Guilford Press

Simard, S.W., Asay, A.K., Beiler, K.J., Bingham, M.A., Deslippe, J.R., He, X., Philip, L.J., Song, Y., Teste, F.P. (2015). Resource transfer between plants through ectomycorrhizal networks – In: Mycorrhizal Networks. Edited by T. R. Horton. Springer, in press.

Sloan, D. M., & Marx, B. P. (2018). Maximizing outcomes associated with expressive writing. *Clinical Psychology: Science and Practice, 25*(1), e12231-n/a. doi:10.1111/cpsp.12231

Solberg Nes, L. S., & Segerstrom, S. C. (2006). Dispositional optimism and coping: A meta-analytic review. *Personality and Social Psychology Review, 10*, 235–251.

Some, S. (2010). The Seen and the Unseen: Sprituality among the Dagara people. Cultural Survival Quarterly Magazine. Retrieved from https://www.culturalsurvival.org/publications/cultural-survival-quarterly/seen-and-unseen-spirituality-among-dagara-people

Song, Y.Y., Simard, S.W., Caroll, A., Mohn, W.W., Zheng, R.(2015). Defoliation of interior Douglas fir elicits carbon transfer and defense signaling to ponderosa pine neighbors through ectomycorrhizal networks. Scientific Reports. 5(8495): 1-9.

Taylor, J. (2008). My Stroke of Insight: A Brain Scientist's Personal Journey. Viking.

Tolle, E. (2005). A new earth: Awakening to your life's purpose. New York: Penguin Group.

Tonarelli, A., Cosentino, C., Tomasoni, C., Nelli, L., Damiani, I., Goisis, S., Sarli, L., & Artioli, G. (2018). Expressive writing. A tool to help health workers of palliative care. *Acta bio-medica : Atenei Parmensis*, *89*(6-S), 35–42. https://doi.org/10.23750/abm.v89i6-S.7452

Touroutoglou, A., Andreano, J., Dickerson, B. C., & Barrett, L. F. (2020). *The tenacious brain: How the anterior mid-cingulate contributes to achieving goals*. Cortex, 123, 12–29. https://doi.org/10.1016/j.cortex.2019.09.001

Toussaint, L. L., & Worthington, E. L. (2015). Forgiveness and health: Scientific evidence and theories relating forgiveness to better health. D. R. Williams (Ed.), . Dordrecht: Springer.

Troy, A. S. (2015). Reappraisal and resilience to stress: Context must be considered. *The Behavioral and Brain Sciences, 38*, e123. doi:10.1017/S0140525X1400171X

Turner, E. H., Matthews, A. M., Linardatos, E., Tell, R. A., & Rosenthal, R. (2008). Selective publication of antidepressant trials and its influence on apparent efficacy. The New England journal of medicine, 358(3), 252–260.
https://doi.org/10.1056/NEJMsa065779

Uvnäs-Moberg K., Petersson M. (2010). “Role of oxytocin and oxytocin related effects in manual therapies,” in The Science and Clinical Application of Manual Therapy, eds King H. H., Jänig W., Patterson M. M., editors. (Amsterdam: Elsevier;).

Vickers, P. & Moyers, R. (2020). Healing Complex Trauma 1: A unity of minds, hearts, and culture Journal of Indigenous Well-being, (5)1. Retrieved from https://journalindigenouswellbeing.com/media/2020/05/126.139.Healing-Complex-Trauma-1-A-unity-of-minds-hearts-and-Culture.pdf

Vlemincx, E., Van Diest, I., & Van den Bergh, O. (2016). A sigh of relief or a sigh to relieve: The psychological and physiological relief effect of deep breaths. *Physiology & Behavior, 165*, 127-135. doi:10.1016/j.physbeh.2016.07.004

Wachholtz, A. B., Malone, C. D., & Pargament, K. I. (2017). Effect of different meditation types on migraine headache medication use. *Behavioral Medicine (Washington, D.C.), 43*(1), 1-8. doi:10.1080/08964289.2015.1024601

Watkins, P. C., Emmons, R. A., Greaves, M. R., & Bell, J. (2018). Joy is a distinct positive emotion: Assessment of joy and relationship to gratitude and well-being. The Journal of Positive Psychology, 13(5), 522-539. doi:10.1080/17439760.2017.1414298

Waldegrave, C. (1990). *Just Therapy*. Dulwich Centre Newsletter, 1(6), 46.

Watson, J. (2003). Love and caring. Ethics of face and hand--an invitation to return to the heart and soul of nursing and our deep humanity. *Nursing administration quarterly, 27.* 197-202.

Watts, R., & Luoma, J. B. (2020). The use of the psychological flexibility model to support psychedelic assisted therapy. *Journal of Contextual Behavioral Science, 15*, 92-102. doi:10.1016/j.jcbs.2019.12.004

Wei, M., Russell, D. W., Mallinckrodt, B., & Vogel, D. L. (2007). The experiences in Close Relationship Scale (ECR)-Short Form: Reliability, validity, and factor structure. *Journal of Personality Assessment, 88,* 187-204.

Weingarten, Kaethe (2003) Compassionate Witnessing and the Transformation of Societal Violence: How Individuals Can Make a Difference. Retrieved from https://www.humiliationstudies.org/documents/WeingartenCompassionateWitnessing.pdf

Wenger-Trayner, E., Wenger-Trayner, B. (2015). Communities of practice a brief introduction. Retrieved from https://wenger-trayner.com/wp-content/uploads/2015/04/07-Brief-introduction-to-communities-of-practice.pdf

Whitaker, R. & Cosgrove, L. (2015). *Psychiatry Under the Influence: Institutional Corruption, Social Injury, and Prescriptions for Reform*. Palgrave MacMillan: New York, NY.

Whyte, D. (2015). Consolations: The Solace, Nourishment and Underlying Meaning of Everyday Words. Many Rivers Press.

Williamson, M. (1996). *A return to love.* HarperCollins.

Wood, A. M., Joseph, S., Lloyd, J., & Atkins, S. (2009). Gratitude Influences Sleep through the Mechanism of Pre-sleep Cognitions, *Journal of Psychosomatic Research 66*(1), 43–48.

Wood, A. M., Maltby, J. J. (2009). Gratitude predicts psychological well-being above the Big Five facets. *Personality and Individual Differences, 46*(4), 443-447 https://doi-org.ezproxy.viu.ca/10.1016/J.PAID.2008.11.012

Yip, J. A., Stein, D. H., Côté, S., & Carney, D. R. (2020). Follow your gut? emotional intelligence moderates the association between physiologically measured somatic markers and risk-taking. United States: American Psychological Association. doi:10.1037/emo0000561

Zanos, P., & Gould, T. D. (2018). Mechanisms of ketamine action as an antidepressant. *Molecular Psychiatry, 23*(4), 801-811. doi:10.1038/mp.2017.255
