# Supplementary material for: A clinical protocol for group-based ketamine-assisted therapy in a community of practice: the Roots To Thrive model
Source: Front Psychiatry. 2025 Sep 22;16:1568017. doi: 10.3389/fpsyt.2025.1568017 (PMC12498912; doi:10.3389/fpsyt.2025.1568017)
Supplement: Supplementary file 6 [file DataSheet6.pdf]

Roots to Thrive Ketamine Assisted Therapy (RTT-KAT) Intake Packet

Roots to Thrive Society for Psychedelic Therapy

Thank you for your interest in participating in RTT-KAT!

Included in this packet are the following documents:

1. RTT-KAT Self-reflection questions
2. RTT-KAT Payment Policy
3. RTT-KAT Onboarding Agreements
4. RTT-KAT Referral Form (requires primary care provider's signature)
5. RTT-KAT Consent form (for your review, signature not required yet)

Please follow the instructions below to process your paperwork. All steps must be completed in the following order to begin your intake process:

1. Register your interest in RTT-KAT via our online portal at https://oab.owlpractice.ca/rootstothrive and verify the account registration email sent to you by Owl Practice.

2. Review the RTT-KAT onboarding agreements (page 4). *Signature will be requested via Owl forms.

3. Complete the physician referral form with your physician (pages 5-7). ***Signature by primary care provider required.** Fax to Roots to Thrive at 250-244-8426 or email to navigator@rootstothrive.com

If you have any questions, please email navigator@rootstothrive.com

Roots to Thrive with Ketamine assisted therapy - Self-Reflection Questions

Roots to Thrive with ketamine assisted therapy is a 12-week virtual program that offers resiliency knowledge, skills, and practice in a supportive community (Community of Practice). It is not group therapy. RTT-KAT includes three in-person ketamine assisted therapy sessions during the 12-week program

Here are some questions to help you decide if this program is a good fit for you:

	Yes	No
Do I have a diagnosis of PTSD, depression, anxiety, and/or substance use disorder that has not significantly improved, despite medication, psychotherapy, and/or other approaches?		
Are you comfortable participating in small groups online? Is it relatively easy for you to listen and speak in small groups?		
Do you have the time, energy, and interest to attend weekly meetings from 6-8:15pm for 12 weeks?		
If needed, do you have childcare support for the virtual weekly meetings and for the three in-person ketamine sessions?		
If you self-medicate with alcohol and/or drugs, your substance use will not detract from your ability to be open-hearted and fully present at meetings?		
Do you have sufficient emotional stability to absorb and integrate new learnings?		
As you are unable to drive until the following day, have you confirmed transportation arrangements to and from the three Ketamine sessions?		

Please note, we recognize the importance of consistent, specialized, and ongoing care for those with complex psychiatric histories. As such, we are not always able to accommodate complex psychiatric histories in this program.

Roots to Thrive Payment Policy

Payments:

As a non-profit society, Roots to Thrive relies on extensive volunteer hours and participant program fees to keep our program operational. We must financially break even, or we cannot continue to offer our programming thus, we thank you for your participation. Further, it is important that payments are received timely for the viability of the program.

The cost of our intake process is $500 and serves as a deposit for participation. When your referral is submitted, this $500 non-refundable deposit is required. Should our intake team determine that the program is not a good fit for you at this time, your $500 deposit will be refunded. The intake team will provide recommendations for next steps to support your program readiness for future cohorts.

The balance of your invoice is due by the program start date. Additional payments may be made toward your balance any time between your initial intake, and the program start date, to allow for spacing of your payments if necessary. Full payments must be received by the start date, or participation will not be permitted.

Payment Options:

Payment Options:

1. **E-Transfer (preferred)**: Please send e-transfers to finance@rootstothrive.com with "roots" as the password.
2. **Cheque**: Please make cheques payable to 'Roots to Thrive Society for Psychedelic Therapy' and mailed to PO Box #41007, RPO Woodgrove, Nanaimo, BC V9T 6M7

We are unable to accept credit card payments at this time.

Refund Policy:

Refunds are reviewed on an individual basis.

Refund requested before start of Community of Practice #1: Full refund, minus the $500 deposit.

Refund requested before start of Community of Practice #2: 50% refund, minus the $500 deposit.

No refunds are given after Community of Practice #2

Roots to Thrive KAT – Onboarding Agreements

INTAKE PROCESS:
Once you have completed the steps above, you will be ready to take part in the intake process with our intake clinicians. All participants in RTT-KAT are required to be medically cleared by our clinical team to participate. This process involves up to three intake appointments with our intake team.

ALL intake appointments are virtual (online) and conducted within the Owl online portal.

CORE INTENTIONS
Throughout the intake process to assess suitability, please be aware of the following core intentions of our program:

- To co-create a community that models unconditional positive regard for ourselves and for each other.
- To practice expressing ourselves authentically.
- To gently step beyond our comfort zones.
- To care for and support others.

INTAKE PROCESS AND COMMUNITY OF PRACTICE AGREEMENTS:

- I acknowledge that I have read and agree to the payment policy.
- I agree to pay the required deposit when my referral is submitted.
- If, for any reason, I am not accepted into the program my deposit will be reimbursed in full.
- I agree to be on time for my intake appointments.
- I agree that if I need to change any of my intake appointments, I will give more than 24 hours' notice.
- I acknowledge that I am available for a 12-week virtual program, held Tuesday evenings 6:00-8:15 pm Pacific Time.
- I agree to have pre-arranged transportation and to not drive until I have had a night's sleep after each of my ketamine medicine sessions.
- I acknowledge that session dates and times may changes and, in this event, the RTT Team will provide as much notice as possible.
- I acknowledge that the RTT program cannot safely support escalating mental health crises. If I experience a crisis (eg: extreme ongoing emotional distress, and/or self-harm ideation), I will cooperate with the team to coordinate transition to appropriate community supports.

These agreements will be sent to you for signature from the Owl online portal.

ROOTS TO THRIVE
Phone: 778-744-0789 | Fax: 250 244 8426 | www.rootstothrive.com

RTT-KAT Referral Form

To Whom It May Concern:

Please accept this request from your patient to participate in the Roots to Thrive with ketamine assisted therapy program.

Roots to Thrive consists of 12 weekly virtual facilitated meetings (referred to as a Community of Practice) that offer resiliency knowledge, skills and practice in a supportive community for individuals experiencing mental distress. It is not group therapy. Over 20 medical professionals are collaborating to run this program. This includes a holistic intake assessment overseen by our lead psychiatrist.

The following article, Published in Frontiers of Psychiatry Journal, provides further information on program design and outcomes to date.
https://www.frontiersin.org/articles/10.3389/fpsyt.2021.803279/full?utm_source=FNTF&utm_medium=EMLX&utm_campaign=PRD_FEOPS_20170000_ARTICLE

PLEASE NOTE: If the patient has a mental health diagnosis, refer based on that diagnosis (if it aligns with the eligibility criteria as listed below). If the patient has not been assigned a diagnosis, please describe the patient's specific challenge(s), which will then be assessed by our intake team prior to entering the program. Medical care and monitoring will be provided by the Roots to Thrive medical team during any planned ketamine medicine sessions. Other medical issues that arise for the participant during the 12-week program will be attended to by the participants primary care provider.

If you have any questions, please email us at navigator@rootstothrive.com with 3 specific days and times you are available for one of our clinicians to follow up. We will email you in response to let you know which day and time.

When the referral form below is completed, please fax to 250-244-8426 (confidential) or email to navigator@rootstothrive.com INCOMPLETE REFERRALS WILL BE DECLINED.

Referral Form for Roots to Thrive Psychedelic Assisted Therapy (for primary care provider to fill and sign)

Name: ____________________________________ Preferred Name: __________________________________
Gender: (most identify with)____________________________ Pronouns: _______________________________
DOB (mm-dd-yyyy): ______________________________ PHN: ____
Primary Phone: _______________________________ Email (mandatory): ______________________________
Alternate Contact: _______________________________ Phone:______________________________________
Address: ____

Provisional Diagnoses: (mark all that apply):

- ❑ Treatment Resistant Depression
- ❑ Generalized Anxiety Disorder
- ❑ Adjustment Disorder
- ❑ Post-Traumatic Stress Disorder
- ❑ Substance Use Disorder
- ❑ Disordered Eating
- ❑ Obsessive Compulsive Disorder

Concurrent Challenges: (separate from formal/ongoing mental health diagnosis (mark all):

- ❑ Unresolved Grief
- ❑ Suicidal Ideation
- ❑ Sleep Disorders
- ❑ Chronic Pain
- ❑ Compulsive Behaviours (gambling, sex, others)
- ❑ Personality Disorder (specify):_________

Exclusion Criteria for Use of Psychedelic Medications (Ketamine, Psilocybin, MDMA): (mark all that apply):

- ❑ Presence of active psychotic symptoms
- ❑ Diagnosis of dementia or delirium
- ❑ Suicide risk
- ❑ Extreme emotional instability
- ❑ Pregnant or Breastfeeding
- ❑ Lack of sufficient understanding or capacity to make or communicate responsible decisions concerning themselves by reason of mental illness, mental deficiency, physical illness or disability, advanced age or other cause (incapacitated persons)

Relative Contraindications to Use of Ketamine

- ❑ Uncontrolled hypertension (above 150/90)
- ❑ Severe liver disease
- ❑ Severe kidney disease
- ❑ Severe cardiac or vascular disease
- ❑ Allergy to ketamine

Exclusion Criteria for Use of Psilocybin

- ❑ Currently receiving chemotherapy, hormonal therapy, radiation therapy, biologic therapies
- ❑ If patient has cancer: cancer has central nervous system involvement
- ❑ If patient is palliative (Est life span is less than 6 months), palliative performance scale score <50%
- ❑ Severe hepatic dysfunction (LFTs > 3x normal)
- ❑ Severe renal insufficiency (GFR < 60)
- ❑ Paraneoplastic syndrome or a tumor with ectopic hormone production which may place the patient at risk for hypercalcemia, Cushing's syndrome, or SIADH secretion
- ❑ Uncontrolled cardiovascular condition or vascular disease
- ❑ Uncontrolled epilepsy
- ❑ Unstable insulin dependent diabetes
- ❑ Allergy to psilocybin

Exclusion Criteria for Use of MDMA

- ❑ History of current of primary psychotic disorder or bipolar affective disorder type 1
- ❑ Severe anorexia or who are actively purging
- ❑ Severely immunocompromised
- ❑ Neurological disease, including seizure disorder
- ❑ Uncontrolled hypertension
- ❑ Unstable peripheral vascular disease
- ❑ Unstable hepatic disease (with or without abnormal liver enzymes)
- ❑ History of hyponatremia
- ❑ History of hyperthermia
- ❑ Allergy to MDMA

Has this person ever been admitted to hospital or emergency for a mental health reason? (**please specify with dates**): ____

____

Current or recent out-patient psychiatric care (**please specify**):____

Any history of self-harm behavior (**please specify**): ____

Other: ____

List all mental health medications tried and dates if known:

____

____

List all mental health therapies (i.e. counselling) tried and dates if known:

____

Has this person ever had a substance use challenge (none; alcohol; tobacco; cannabis; opioids/meth; other):

____

Family Mental Health (who and what mental health challenge):

____

Family Substance Use History (who and what substances):

____

Allergies:____

Is this person able to tolerate group work? Yes/No

Current Medications (noted but not limited to):

- ❑ Lithium________________________________
- ❑ Tramadol_______________________________
- ❑ Benzodiazepines_____________________
- ❑ Dextromethorphan___________________
- ❑ Lamotrigine_________________________
- ❑ Buprenorphine__________________________
- ❑ Psychostimulants (including ADHD medications) __________________
- ❑ MAOIs (Phenelzine, Selegiline) __________________
- ❑ Ozempic (semaglutide)________________________
- ❑ Antidepressants (venlafaxine, bupropion, desipramine) _______________
- ❑ Calcineurin inhibitors (cyclosporine, tacrolimus) _________________
- ❑ Corticosteroids _______________
- ❑ Estrogens_______________
- ❑ NSAIDS________________________
- ❑ Testosterone: _____________
- ❑ Triptans: ______________
- ❑ Other **(please list all other medications):** ____

Please list all current supplements (even if intermittent):

____

Relevant Abnormal Lab/ECG Results:

____

Accessibility:

- ❑ Ambulatory aids
- ❑ Ostomy Bags
- ❑ Disabilities
- ❑ Other (please specify): _____________________________________

Mandatory Vital Signs

Date:_______ BP:__________ Respiratory Rate: ____________ Pulse:_______ Weight (kg please): ___________

Is there any reason that this patient past or present could not sustain a 4-5 hours of increased blood pressure?

- ❑ No
- ❑ Yes, Please explain:____

Referring Health Care Provider: ____
Phone: ___________________________________ Fax: ____

HCP Signature: ______________________________ HCP's Billing #: ___________________ Date: ______________

Participant Information for Review

Roots to Thrive Society for Psychedelic Therapy

ALL RTT-KAT PARTICIPANTS MUST REVIEW PRIOR TO RTT AND SIGN PRIOR TO KAT

CLINICAL TREATMENT PROGRAM CONSENT FORM

Roots to Thrive (RTT) & Ketamine Assisted Therapy (KAT)

You have expressed interest in Ketamine Assisted Therapy (KAT), a new and evidence-informed option that uses the medicine Ketamine in partnership with therapeutic activities and relationships. The following information is to help you decide if KAT is right for you, describing how KAT works, the potential benefits and risks of participation, and how you will be supported throughout the program. If you choose to participate, you can decide to withdraw at any time during the program (please refer to the payment policy). In this case, please let both the RTT team and the physician who referred you know.

Please ensure all your questions have been answered by the RTT team before signing this document. Signing this consent is required to enter the RTT-KAT program, but it does not guarantee your entry. A fulsome intake process (medical and psychiatric) to ensure your safety and suitability for the program is required prior to participating in RTT-KAT. In the RTT-KAT psychiatric intake process, you will be screened for suicide risk. This is done through iterative conversation with the intake team. For example, by asking: 1. Do you feel that life is worth living? 2. Do you wish you were dead? 3. Do you have thoughts of self-harm? 4. Do you have a plan?" If suicidality is a concern, the RTT-KAT intake team will determine the best course of action, based on the unique situation.

RTT Community of Practice (CoP) Weekly Meetings

Weekly CoP meetings serve as the container for unconditional positive regard, a core therapeutic tool utilized in RTT. Small groups meet virtually every week for two hours during the 3-month program. These CoPs aim to mirror unconditional positive regard and to cultivate greater resiliency individually and collectively. The RTT CoPs have shown improvements in cognitive control and many other personal and social benefits (gaining a sense of belonging in community and ultimately, resiliency).

Information About Ketamine

Ketamine has been used since the 1970's as an anesthetic and more recently for pain control and frequently in the ER for trauma & procedural sedation. There are now clinical trials supporting its use for Treatment Resistant Depression, Suicidality, PTSD and more. In RTT-KAT, generic ketamine is used "off-label" to treat mental health conditions. This means that ketamine is being used for a purpose that is not listed on the official label (for what it was initially approved to be used for). At higher doses, ketamine's mode of action mimics a psychedelic, which can work very quickly to significantly reduce anxiety and depression and create a sense of space or detachment from emotions and sensations. It can also bring about different states of consciousness, including what might be described as spiritual or mystical awareness. These effects can

support new insights; greater access to and/or a reorientation to memories and thought patterns; a different sense of self and reality. In a safe environment with therapeutic support, working with ketamine can help individuals heal and transform, finding greater peace and compassion.

How Ketamine Works

One of the current theories of ketamine's mode of action is as an NMDA antagonist working through the glutamate neurotransmitter system. This is a different pathway than that of other psychiatric drugs such as the SSRIs, SNRIS, lamotrigine, anti-psychotics, benzodiazepines, etc.

Ketamine is classified as a dissociative anesthetic, dissociation meaning a sense of disconnection from one's ordinary reality and usual self. At the much lower dosage level administered in RTT-KAT, one will most likely experience mild anesthetic, anxiolytic, antidepressant and, potentially, psychedelic effects.

No matter the route of administration, ketamine tends to produce dissociative effects in a broad range—almost none occurring in a session to strongly present– depending on dosage and one's susceptibility. The evidence supports that the 'dissociative' experiences may well be instrumental in providing a more robust effect. In the trance state, these effects are deliberately reduced in intensity to enable therapy and direct communication. At higher but still sub-anesthetic doses, the dissociative effect predominates for a time, usually about a half hour to an hour.

Ketamine treatment provides essential time-outs from our ordinary states of mind, this period being of varying duration, usually 30 to 90 minutes, though it will seem timeless. Characteristically, there is a relaxation from ordinary concerns and usual mind, while maintaining conscious awareness of the flow of mind under the influence of ketamine. This tends to lead to a disruption of negative feelings and obsessional preoccupations. This relief, and the exploration and experience of other possible states of consciousness, are uniquely impactful. As your Roots to Thrive Team, we act as guides to the experience, preparing you for it and facilitating your process. For most people, KAT is singularly beneficial.

How a KAT Session Works

The Ketamine Assisted Therapy (KAT) component of the program consists of a series of three sessions. During your KAT treatment sessions, you and your small group will be accompanied by a medical clinician and a KAT trained therapeutic sitter. Once ketamine is taken, the majority of effects will last between one to two hours.

Unlike other forms of therapy, KAT is largely an inner journey. You will not be talking through your experience while the medicine effects are unfolding. The therapy team will be present and available to provide you with support at any time. There will be an opportunity to share and process your experience with your small group and the therapy team once the effects of the medicine have worn off and, in the days following.

To support an inward focus and minimize external distraction, instrumental music will be played, and we encourage the use of eye shades. Before, during, and after your session, we will monitor your physical vital signs, and will be available to answer any questions you have.

As the medicine begins to wear off, we will encourage you to continue exploring your inner world, and to work with stress regulation and loving-kindness practices that resonate with you, as you transition back to your ordinary state of consciousness.

Part of your KAT preparation work (with your therapist and/or community of practice) will be to explore such regulation and loving-kindness practices, for use at any time. To continue assessing your needs and progress, and to improve the quality of our program, we will also ask you to complete feedback surveys at various points as you move through the RTT-KAT program. Quality improvement results that speak to program impact will be shared with others, but no personal information will be shared.

Roots to Thrive (RTT) Group KAT Sessions

The Roots to Thrive (RTT) community of practice (CoP) program is the primary support and development vehicle in the RTT-KAT program. You will meet virtually every week for two hours during the 3-month RTT-KAT program. Within your 3-month treatment period, you will also participate in three group KAT sessions with the same small group you are meeting with weekly.

Please acknowledge your understanding and consent to the statements below:

You will receive ketamine in a group format, with participants who are also part of your community of practice.

- Mirroring unconditional positive regard is a primary principle in the group format. As such, people may come into the program who have a history where they may have acted out in ways that resulted in harm to themselves or others. These acts may be of a physical, emotional, or sexual nature. As a program, we understand that other people's past transgressions may result in trauma being activated in others. We see these events as opportunities for growth, as long as well supported and managed, in a safe relational environment. Please reach out to a team member if you need more support or accommodations. We, as a team, are committed to ensuring that all participants can relax into a co-created environment that feels physically, emotionally, and spiritually safe for all. As your team members, we will do our best to minimize and manage any risks. We are in this together!
- All participants are required to keep other's experience and sharing confidential within the group, and to protect group agreements for creating and maintaining a safe and supportive environment.
- Within the group, participants must agree to open sharing: no one will ask another to hold a secret.
- If a group member shares privately with you a concern for their health or wellbeing, this must be reported to a facilitator right away, for everyone's safety, trust and comfort.
- You will remain onsite for the duration of your KAT treatment (4-5 hours) to enable adequate time to transition home. Prior to leaving, you will participate in a group debrief, have a set of vital signs taken, walk to the bathroom, tidy your space, and when you feel ready, you will be walked to your ride home.

Please initial here that you understand and consent to the above statements: ______.

Risks

Ketamine is a Health Canada-approved drug that we are using "off-label" for ketamine-assisted therapy. It has an extensive record of safety and has been used at much higher doses for surgical anesthesia, without respiratory depression. As with any other medication, there are also some potential risks and side effects to be informed of and consider.

The most common physical side effect is a short-term rise in blood pressure or heart rate, which may be a risk to those with any condition that high blood pressure may be an issue. Other common side effects include dizziness/light-headedness, sedation, impaired balance, excitability, relaxation, headache, anxiety, nausea, and vomiting. These effects are transient and resolve as the active phase of the medication ends (generally within 2-4 hours). Some people may feel the effects for a few days thus we suggest that you leave the days following your ketamine sessions unscheduled.

Repeated, high dose, chronic use of ketamine has caused urinary tract symptoms and even permanent bladder dysfunction or cystitis. These adverse effects are unlikely in medically supervised ketamine treatment populations, but might include more frequent, painful, or difficult urination. Please inform your providers immediately if you notice any of these side effects.

In terms of psychological risk, ketamine has been shown to worsen certain psychotic symptoms in people who suffer from schizophrenia or other serious mental disorders. It may also worsen underlying psychological problems in people with severe personality disorders and dissociative disorders.

To mitigate potential risks, the following conditions will make you ineligible to receive ketamine-assisted therapy.

- *Hypersensitivity to ketamine,*
- *Presence of active psychotic symptoms,*
- *Diagnosis of dementia/delirium, high risk for coronary artery disease, uncontrolled cardiopulmonary disease/ cardiovascular disease/hypertension, aneurysm, history of intracerebral hemorrhage, hepatic cirrhosis, hepatorenal disease,*
- *Pregnancy or if breastfeeding, must agree to refrain within 11 hours of ketamine administration.*

Additionally, you should plan for a 4–5-hour window of time to be on site during KAT session days. This allows time for a multi-step check-in and assessment process. The medical portion of the KAT session will unfold within a 3-hour period. You will then have transition time before transitioning home.

Crisis Services

Roots to Thrive is unable to provide mental health crisis response services. In the event that these services are required during program duration, our team will promptly connect you with community-based mental health supports.

Benefits

The benefits are described in previous sections of this document. It must be recognized that you may or may not receive any benefit from your participation.

Participation

Your participation is voluntary. You can choose not to participate, or you may withdraw at any time (please refer to payment policy).

Managing Dual Roles

When practitioners investigate their own professional practices for quality improvement or research purposes, and there are patients for which they have a previously established relationship (I.e. as a teacher, colleague, employee, spouse, dependent), it's called a dual role. Dual roles can also exist between team members, between a team member and a patient, or between two patients. While dual roles are not necessarily problematic, they do need to be articulated and managed, especially when involving positions of authority (power over) and when patient confidentiality/privacy is a concern. To manage dual roles, all patients and team members will be asked to disclose dual roles. If such roles exist the team will work with you to determine how best to manage them, mitigating any potential risks.

Cost

The RTT-KAT program is based on a cost-recovery model. These costs are regularly assessed based on program needs. Expenses may be covered through a mix of private pay and philanthropic funds to support scholarships for those with limited resources (as available).

Questions

If you suffer any concerning effects from your treatment or if you have any general questions, please report to the clinician overseeing your care.

Confidentiality

RTT clinical data is stored within a secure online, electronic medical records system.

All medical and other personal information and records are collected and used under the authority of the section 26(c) of the B.C. *Freedom of Information and Protection of Privacy Act*, and will be kept confidential to ensure your privacy, as per the *Health Professions Act* and the regulations of each of the professional governing bodies for the attending professionals and applicable law. They will be maintained with the same precautions as all medical records. Your personal information will only be shared with third party health professionals for the purpose of conducting and monitoring your treatment (i.e. sending a discharge note to your referring physician).

Please note, legal and professional ethical responsibilities do require disclosure of confidential personal information in very limited circumstances, such as where you threaten the bodily harm or death of yourself (i.e. suicidality) or others, or you report abuse regarding a child or vulnerable adult.

If you have questions or concerns regarding the collection and use of your personal information, please email Julia@rootstothrive.com.

You have the legal right to access your personal information and, if need be, an opportunity to correct any errors in this information.

___ I understand all information stated in the above statements regarding confidentiality.

___ I understand that the email address I provide to the program will be used to receive and send communications and health and wellness measures between myself and the RTT team.

Data Collection and Secondary use of Data for Research Purposes

To better understand the impact of this program and to continue improving the quality of our program, we may ask you to complete various mental health and feedback questionnaires throughout the program. If you consent to the use of your de-identified treatment data for research purposes, the pooled data may be published in a peer-reviewed journal. These results will not include personal or otherwise identifiable information. No information or record that discloses your identity will be removed or released without your consent unless required by law. If you choose not to include your personal data in the program of research, you can continue in the program without ramifications.

Do you permit the use of your de-identified treatment data to be used for research purposes?
Please indicate "Yes" or "No" below.

Yes _____ No _____

If you have any questions or concerns about the use of your data for research purposes, please contact Shannon Dames at Shannon.dames@viu.ca , who oversees the research and development of the Roots to Thrive program.

Medical Informed Consent:

*Please initial each statement, which signifies that you agree with each of the statements below:

This document will be sent to you electronically during the intake process. Please read through it and make sure you agree to the statements, no initials are required at this point.

___ I have read and understood the verbal and written patient information provided to me. This includes information on how ketamine works, indications for use, contraindications, side effects, dosing, route of administration and how I can prepare for ketamine assisted therapy sessions.

___ I am aware that ketamine for this protocol is being used "off label," i.e. it has not been approved by Health Canada for this purpose. I have tried other treatments which I have not tolerated or have not been effective and have considered other alternatives prior to ketamine assisted therapy.

_____I am aware that while research suggests there are potential benefits of this treatment, it is still being studied.

___ I understand the medical risks and benefits, and I freely give my consent to participate in ketamine assisted therapy as outlined in this form, and under the conditions indicated in it.

___ I have had the opportunity to raise questions and have received satisfactory answers. Before I make my decision about participating in ketamine assisted therapy, I have the right to ask and will be encouraged to ask any questions I may have about the process.

___ My decision to undertake ketamine assisted therapy is completely voluntary.

___ No oral or written statements have been made to pressure me to undertake ketamine assisted therapy.

___ I understand that I may withdraw from ketamine assisted therapy at any time, up until the actual injection has been given.

___ I fully understand that the ketamine assisted therapy session(s) can result in a profound change in mental state and may result in unusual psychological and physiological effects.

___ I understand that I must have someone drive me home from the sessions, and not operate heavy machinery or engage in any driving or hazardous activity for at least 6 hours or more, depending on the continued presence of effects after my session has concluded. The RTT-KAT team may release me to alternative forms of transportation (i.e. Local transit or taxi) based on their assessment of my condition after the session has concluded.

___ I understand that I am to have no food or drink 4 hours prior to my ketamine assisted therapy session.

___ I understand that if I show up intoxicated or under the influence of other substances, I will not be able to undertake ketamine assisted therapy.

___ I understand that I may be offered lorazepam if deemed necessary for anxiety, ondansetron for nausea, and captopril or clonidine for high blood pressure.

___ I understand that I can keep a copy of this RTT-KAT Consent form (please prompt the team for a signed version if you wish to receive one).

By signing this consent, you indicate that you understand the information provided and that you give your consent to participate in ketamine assisted therapy, and the collection of your personal data and program improvement suggestions. In addition, you have had the opportunity to discuss our therapeutic methods and medical procedures and your questions have been answered to your satisfaction. By signing below, you are consenting to take part in this treatment with the understanding that you may withdraw at any time without affecting the availability of continued medical care (outside of this therapy). Upon request, you will be provided with a signed copy of this consent form.

A copy of this document will be sent to you electronically from Owl during the intake process. No initials or signature are required now.
