# Supplementary material for: A clinical protocol for group-based ketamine-assisted therapy in a community of practice: the Roots To Thrive model
Source: Front Psychiatry. 2025 Sep 22;16:1568017. doi: 10.3389/fpsyt.2025.1568017 (PMC12498912; doi:10.3389/fpsyt.2025.1568017)
Supplement: Supplementary file 7 [file DataSheet7.pdf]

Post Ketamine Safety Check list:

- ✓ No driving or operating potentially dangerous equipment for the rest of the day.
- ✓ Keep the rest of the day low key. Rest. Journal. Listen to music.
- ✓ Arrange a ride home after your session.
- ✓ Hydrate frequently and eat healthy food.
- ✓ If you feel nauseated, eat bland food, try ginger or peppermint tea.
- ✓ If you feel dizzy or unsteady, it will be short lived. Relax, sit or lay down until it passes.
- ✓ Both fatigue and bursts of energy after a session are normal. If you are fatigued, please rest. If you have bursts of energy, please do not overly exert yourself.
- ✓ Continue your regular medications. If you feel the urge to stop any, please discuss it first with your primary care provider.
- ✓ If you have a headache, take an over the counter medication that you are familiar with (ibuprofen, acetaminophen)
- ✓ If you feel more emotional, anxious or depressed following your session please contact any of your clinical team listed below or your buddy in the community of practice.

Post Ketamine Session Team:
(we are available to you 24 hours a day if needed in emergency situation following your KAT session)

Provider 1: …
Provider 2: …
Provider 3: …
