# Supplementary material for: A clinical protocol for group-based ketamine-assisted therapy in a community of practice: the Roots To Thrive model
Source: Front Psychiatry. 2025 Sep 22;16:1568017. doi: 10.3389/fpsyt.2025.1568017 (PMC12498912; doi:10.3389/fpsyt.2025.1568017)
Supplement: Supplementary file 8 [file DataSheet8.pdf]

Roots to Thrive Community of Practice (CoP)
Intentions and Agreements

Each group should adapt these intentions and agreements to ensure collective buy-in and co-create a space that feels safe, inclusive, and supportive for all. This document will be developed during the first 2-3 meetings and reviewed mid-way through the program.

Intentions of Our Roots to Thrive Circle:

1. To co-create a community that practices compassion, caring and unconditional positive regard for ourselves and each other.
2. To practice expressing ourselves authentically and honestly, rather than saying what we think others want to hear.
3. To develop our ability to comfort and calm ourselves in moments of suffering (stress).
4. To contribute to feelings of safety by honouring vulnerability.
5. To gently step beyond our comfort zones.
6. To experience authentic connection to ourselves and to one another.

Agreements for participation in our Roots to Thrive Circle: Each member of our Circle is asked to commit to these agreements.

1. I agree to protect the privacy (confidentiality and anonymity) of group members. Outside of this circle I will only speak about my own experience, and not that of others. When possible, I will use a private space for the group Zoom meetings; if not I'll use headphones, and ensure my screen isn't visible to others, so that only I see and hear the group.
2. I agree to co-create a safe 'container,' where we practice giving and receiving unconditional positive regard and honour diversity.
3. I agree to practice listening compassionately, and to share from a place of "I want to hear" rather than "I want to fix".
4. I agree to refrain from analyzing or redirecting focus from someone's share or healing process to myself, ensuring the space remains centered, supportive, and respectful of the speaker's experience.
5. I agree to not interrupt or cross-talk, so everyone feels free to share without judgment.
6. I agree to be on time for meetings. If I need to be late, I will text or call one person to let them know approximately when I will arrive (no need to explain why).
7. I agree to attend the first 4 meetings, and at least 3 out of every 4 meetings thereafter.
8. If unable to attend a meeting, I agree to email the group with a check in as to how my week has been, just as I would during a meeting (e.g. practicing vulnerability, responding to that week's reflection questions).

9. If I use substances, I agree to not use an amount or at a time that would interfere with my ability to participate in group.
10. If I am aware of being at risk of harming myself or others, I will seek help.
11. I agree that if I have a safety concern for a group member or a group member shares a concern for their own safety or the safety of another with me, I will bring this information to a facilitator or team member for help. This is necessary to enable each of us to remain focused on our healing, to prevent moral distress, and for those of us in the helping professions, to satisfy any legal reporting requirements.
12. I understand that this community of practice is intended as a support but is not a replacement for any other therapy or healing work I will continue doing.
13. Because feelings of intimacy, warmth and affection can happen in these groups, I agree to keep all relationships within the group platonic and not pursue any romantic feelings. This is important to protect the sense of safety for everyone in the group.
14. I understand that I am not to ask other members of the group to hold any secrets.
15. If I leave the group, I will attend one final meeting to let everyone know that I will be leaving, allowing for good-byes and closure.
