# Supplementary material for: A clinical protocol for group-based ketamine-assisted therapy in a community of practice: the Roots To Thrive model
Source: Front Psychiatry. 2025 Sep 22;16:1568017. doi: 10.3389/fpsyt.2025.1568017 (PMC12498912; doi:10.3389/fpsyt.2025.1568017)
Supplement: Supplementary file 9 [file DataSheet9.pdf]

TOUCH PROTOCOL

Roots to Thrive Society

Considerations

Participants in psychedelic-assisted therapy are in a very vulnerable space. It is imperative that facilitators and team members maintain a high degree of professionalism and ethical integrity. A felt sense of safety allows participants to fully engage with their own inner healing intelligence, retreating from external distractions.

It is important that facilitators do their own healing work to avoid affecting participants with their own wounds. Ultimately, as we are invited to move into the vulnerable spaces of another, the role of a psychedelic therapist is sacred. The responsibility we carry as practitioners who move beyond the 'ordinary' [per western culture] physical veil is immense. As individuals, let's hold intention for integrity and compassion to drive our choices. As professionals, let's remain in the scope of the regulatory board requirements and/or agreements that we are accountable to. As a team, let's help each other rise to this sacred calling.

Development of self-awareness on the part of facilitators and team members must be ongoing and thorough. Facilitators need to be vigilant for transference/countertransference. Practices of self-care, self-examination and self-growth are important. Sexual boundary violations occur due to insufficient healing work on the part of the guide/sitter/therapist and pose an ongoing problem in all therapies, including psychedelic therapy.

Facilitator's state of Consciousness

The facilitator's own state of consciousness is fundamental in creating a safe environment for participants. Daily mindful practices using breath or visualization are extremely effective at cultivating a still, inner space. Supporting participants in psychedelic-assisted therapy while experiencing one's own inner stillness, is known as 'holding space'. This maintains the 'healing field' and is of primary importance.

Informed Consent

Informed consent reinforces the participant's autonomy and a felt sense of safety. Participants are informed that all physical touch is optional and choose whether they would like supportive touch. Touch may be useful when participants have difficulty settling (usually at the beginning of a session) or when difficult emotions arise during the session. The purpose of touch is not to suppress challenging emotions but to assist the participant remaining within the temporarily expanded window of tolerance during the experience.

It is important that participants know that consent to touch does not mean that touch will occur. Spending maximum time 'in and down' in connection with the inner healer is of prime importance. To keep distractions to a minimum, any intervention will only occur if it is deemed necessary.

The participants' manual and facilitator script (read prior to administering the medicine) clearly describes what touch may be expected. Examples include:

-holding a hand
-placing a hand on the forehead
-holding the ankles or the bottoms of the feet
-placing a hand on the shoulder
-placing a hand on the back

In addition, a short video is available to model this compassionate touch to participants.

The energy workers on the team have been trained in using additional techniques, including holding points on the head, hands or feet that are affiliated with emotions. This supports a shift in the perception of suppressed emotions. Holding the back or front of the chest can regulate the heart's energy field. Beyond physical touch, energy work can be done in the field above the body.

These techniques are explained in the participants' manual, in the script and in the video.

Participants give consent to touch prior to the administration of medicine. All requests are honoured. Physical support can be requested at any time during the session. Participants are invited to signal requests for support by raising a hand. If a participant requests no touch prior to a session, then asks for touch while under the medicine, no touch is given, unless required for physical safety.

Priority is given to the decision made while not under the influence of medicine.

Further safety measures include always having more than one team member in the session rooms. Participants are asked if they have any gender boundaries regarding touch. If a participant needs help inside the washroom, a person of the same gender will assist.

Compassionate Touch vs, Energy work

Given that our physical body is continuous with our energy body, *all* touch is 'energy work' and there is no need to separate 'compassionate touch' from 'energy work'. The word *touch* encompasses both elements: above the body, or in contact with the surface of the body. Separating the components creates confusion.

Alternatives for Touch

As an alternative to touch, participants may be offered the use of a weighted blanket (stored at the foot of the pad), placed over the full body, just on the feet or torso or as a 'swaddle'.

Participants can be invited/encouraged to hold their own hand, or place a hand over their heart, or elsewhere. Participants can be reminded to bring their Pillars of Strength to mind during any challenging portion of the session.

These are powerful self-help techniques for participants.

A Gift and a Responsibility

Appropriate use of touch is an age-old catalyst for healing. It is both a gift and a responsibility to be able to participate in these healing practices (MAPS Code of Ethics: January 2021).

Links to Touch Video and Grounding Exercises

How Grounded Am I

https://www.pruneharris.com/post/reinforcing-the-auric-membrane

RTT Energy Work Info Video - YouTube

RTT Touch Informational Video - YouTube

Contemplative Questions:

It is very important to recognize that **all people** in this work have the possibility of transgressing or causing harm. It is by acknowledging this regularly that one is less likely to. (Andrea Celenza's book is a great resource for this discussion).

It is when we are most challenged in our lives that we are most at risk – a divorce, recent challenge at work, death of a loved one, financial challenges, etc. These may be times to step away from this work until we are feeling solid on our foundation and is a great time to harness the support of a colleague / professional accountability group.

PSYCHEDELIC ASSISTED THERAPY SESSION SUPPORT RECORD

Date:	Stream:	Cohort:	Session #:
Facilitator:	Co-Facilitator:	Nurse:	MD:

Printed name of person filling out form: ______________________ Signature _______________________

Support Team

Name:	Role:	Things to note:

Participant requests for Support and Boundaries

Name:<br><br>Consent to touch<br>❑ Yes<br>❑ No	Support:	Boundaries:

Name:<br><br>Consent to touch<br>❑ Yes<br>❑ No	Support:	Boundaries:

Name:<br><br>Consent to touch<br>❑ Yes<br>❑ No	Support:	Boundaries:

Name:<br><br>Consent to touch<br>❑ Yes<br>❑ No	Support:	Boundaries:

Name:<br><br>Consent to touch<br>❑ Yes<br>❑ No	Support:	Boundaries:

Name:<br><br>Consent to touch<br>❑ Yes<br>❑ No	Support:	Boundaries:

Name:<br><br>Consent to touch<br>❑ Yes<br>❑ No	Support:	Boundaries:

Name:<br><br>Consent to touch<br>❑ Yes<br>❑ No	Support:	Boundaries:
