# Supplementary material for: A clinical protocol for group-based ketamine-assisted therapy in a community of practice: the Roots To Thrive model
Source: Front Psychiatry. 2025 Sep 22;16:1568017. doi: 10.3389/fpsyt.2025.1568017 (PMC12498912; doi:10.3389/fpsyt.2025.1568017)
Supplement: Supplementary file 10 [file DataSheet10.pdf]

Ketamine Assisted Therapy Session Record (Rev. 02/21/25)

Allergies: ____________________________Gluten sensitivity: Yes___ No___

Date: ______________Arrival Time: _________ Session #_____ Alum_____ Exp._____ Ind. ______
If 3rd RTT-KaT Session, unisex T-shirt size ________

Driver Name______________________ Driver phone#________________
Provided with Post Ketamine Safety Sheet ______ Signed Consent in OWL: Yes____ No____

Baseline VS: **Time: _____** BP: ____ /___ HR: ____ Weight: _____
See MD Rationale Form for With-Holding or Administering Ketamine When BP Elevated______
Mental State On Arrival: Calm___ Anxious___ Other______________Nurse/MD initials: _____

Ketamine Order: Dose: _____ mg IM Needle Size: 1" ___ 1.5" ___
Top-up Dose: ________mg. IM
_____Give only if /asks for top-up before 20 minutes post dose.
_____Offer post dose at: 5 min___ 10 min___ 15 min___ 20 min___

Signature of Dr.: ___________________________________
Dr. P. Kryskow (#63029) ____ Dr. B. Fehlau (#24285) ____

Ketamine Draws: *(If Total Dose > 2.5 ml consider dividing dose into 2 syringes)*
Ketamine draw from vial to syringe (50mg/1ml) ______mg/_____ml
Signature: ______________________ Designation: MD: ___ RN: ___RPN: ___NP: ___
Double Check of Dose(s): ____ (clinician initial)

Top-up: Ketamine draw from vial to syringe (50 mg/1ml) ______mg/_____ml
Signature: ______________________ Designation: MD: ___ RN: ___RPN: ___NP: ___
Double Check of Top-up Dose(s): ____ (clinician initial)

Ketamine Administration:
Administration **Time**: ________ **Dose**: _______ mg. IM DG: Rt.___ Lt.____
Signature: ______________________ Designation: MD: ___ RN: ___RPN: ___NP: ___

Top-up **Time: _______ Dose: ______**mg. IM Deltoid Rt.___ Lt.____
Signature: ______________________ Designation: MD: ___ RN: ___RPN: ___NP: ___

Date: ________________________

PRN Medications: Standing Orders.

Time:	Medication:	Dosing Range:	Dose Given:	Route:	Signature
	Ondansetron	4-16mg.		SL/PO	
	Dimenhydrinate	50 mg		PO	
	Epinephrine (1mg/1ml)	0.5mg x 3 q 5 min		IM	
	Ibuprofen	200-400 mg		PO	
	Acetaminophen	325-650 mg.		PO	
	Ativan	0.5mg		SL	
	Clonidine (repeat x 1 with MD order)	0.1-0.2mg		PO	
	Captopril (with MD order)	12.5 – 25 mg		PO	
	Nicoderm Patch Step 3	7 mg.		Dermal	

Session/Post Session Notes *(Charting is by exception only)*

Time	Notes

Discharge Vital Signs

Time: ______ BP*:* ___/____ HR: _________
Mental State Post Session: Calm__ Anxious__ Other________________ Provider Initials_____

Post session Notes: *(Nurse completes if any of the following were experienced by participant post session)*

	X	Interventions	Effectiveness
Nausea			
Vomiting			
Headache			
Dizziness			
Hypertension (>150/90)			
Altered state > 120 minutes			
Other (see notes)			

**Final Discharge** – completed upon discharge *(Initial which is true):* Escorted to driver____ Front door____ Door and left on foot____ Signature:____________________	**Time:**
